# Supplementary material for: Growth, physical, and cognitive function in children who are born HIV-free: School-age follow-up of a cluster-randomised trial in rural Zimbabwe
Source: PLoS Med. 2024 Oct 11;21(10):e1004347. doi: 10.1371/journal.pmed.1004347 (PMC11498706; doi:10.1371/journal.pmed.1004347)
Supplement: S1 Text — Table A. Baseline and early-life characteristics of HIV–positive women and their infants enrolled and not enrolled in the follow-up cohort. n: number, SD: standard deviation, IQR: Inter-quartile range, LAZ: Length-for-age Z-score, WAZ: Weight-for-age Z-score, WHZ: Weight-for-height Z-score, HCZ: Head circumference for Z-score, MUACZ: Mid-upper arm circumference Z-score, Hb: Haemoglobin, Chi-Sq: Chi -Square from logistic regression and adjusted for clustering. GEE (robust) Generalised estimating equations with robust variance estimation adjusted for clustering, Somers’ D comparison of medians using t-distribution adjusted for clustering. *Of the 267 caregivers, data were not available for 2 women at baseline entry to the trial, and for 7 of the not included group. Applying a Bonferroni correction for multiple comparisons, a P-value <0.001 is considered significant. Table B. Baseline and early-life characteristics of HIV–negative women and their infants enrolled and not enrolled in the follow-up cohort. n: number, SD: standard deviation, IQR: Inter-quartile range, LAZ: Length-for-age Z-score, WAZ: Weight-for-age Z-score, WHZ: Weight-for-height Z-score, HCZ: Head circumference for Z-score, MUACZ: Mid-upper arm circumference Z-score, Hb: Haemoglobin, Chi-Sq: Chi -Square from logistic regression and adjusted for clustering. GEE (robust) Generalised estimating equations with robust variance estimation adjusted for clustering, Somers’ D comparison of medians using t-distribution adjusted for clustering. There were less mothers than children due to some households having twins. *Note that of the 988 HIV–negative mothers recruited into SHINE follow-up, data were not available for 66 mothers at the baseline visit and similarly for the 2,949 HIV–negative mothers not recruited, data were not available for 182 mothers at the baseline visit. **Note that 1,002 children included 2 children who were HIV–positive as their mothers seroconverted during breastfeeding and the children be [file pmed.1004347.s001.docx]

# S1 Supporting information for Growth, physical and cognitive function in children who are born HIV-free: school-age follow-up of a cluster-randomized trial in rural Zimbabwe

## Further description of scales used in baseline questionnaire

The socioeconomic status was recorded using a locally validated wealth index based on the household’s type of buildings and ownership of assets such as a wheelbarrow or chickens[1], with a higher score indicating greater household wealth. The coping strategies index is a validated measure of food insecurity, with a higher score showing greater food insecurity[2]. Caregiver depression was measured using the Edinburgh Postnatal Depression Scale, a 10-item self-report questionnaire which has previously been validated as a screening tool for depression in Zimbabwe[3], where a higher score represents more severe depressive symptoms. A higher score in gender norm attitudes reflects more equitable gender norms in the caregiver[4]. Similarly, an increase in the social support score reflects the caregiver’s perception of improved support from neighbours and family, particularly in sharing problems[4]. Both gender norms and social support scores have previously been associated with improved child growth at 18 months [5]. Maternal schooling exposure was measured by number of years of education.

## Further description of scales used in contemporary caregiver questionnaire

Child schooling exposure was measured in number of years of education combined with months of school attended in the current year. Adversity was measured using a range of questions exploring recent adversities in the child, caregiver or household, based on previous work in India[6] and used in a pilot study[7], with a higher score reflecting greater adversity. Nurturing was measured using the Child-Parent Relationship scale, where a higher score represents a more positive relationship of the child with the caregiver[8]. The MICS Child Discipline questionnaire asks a series of questions on methods of discipline used for the child, with a higher score reflecting more harsh methods of discipline[9, 10]. The Household Food Insecurity Assessment Scale (HFIAS) reflects food coping behaviours in the previous 28 days, with a higher score indicating greater food insecurity[11]. The Household Dietary Diversity Scale (HDDS) represents the range of foods eaten in the past week, with a higher score representing greater diversity[12]. Both HFIAS and HDDS have been previously used in Zimbabwe together to describe food security[13]. The Household Water Insecurity Experiences Scale (HWISE) also reflects water scarcity coping behaviours within the past 28 days, with a higher score representing more water insecurity[14].

### Description of secondary outcomes

The Kaufman Assessment Battery for Children 2^nd^ edition (KABC-II) assesses cognitive processing using Luria’s model by focusing on tests that children have not been previously exposed to in schools. Hence the test results may be less dependent on overall schooling, which is suitable in circumstances where similar-aged children may have very different schooling exposure due to socioeconomic and environmental factors. The KABC-II can be condensed to eight subtests which are scaled based on the participant’s age, so that younger children have a higher scaled score for each test. The scaled results from two of the eight subtests are added together to create the four Luria domains of cognitive processing. The Sequential memory domain focuses on short-term sequential memory using the Number Recall and Word Order subtests. The Planning domain represents pattern recognition and problem-solving using the Story Completion and Pattern Reasoning subtests (this included the adaptations for Zimbabwe previously published[15]). Short- and long-term memory provide the Learning domain, measured using the Atlantis and Atlantis Delayed subtests. Finally, the Simultaneous domain measures logical and spatial problem solving using the Rover and Triangles subtests.

The school achievement test (SAT) was developed previously in a pilot study cohort[7] and measured numeracy, reading and writing skills. Briefly, numeracy was the total from counting, visual and figure-based questions based on previous studies[16] [10]. Reading was the total from timed questions on reading letters, words and a local story, with participants given a choice of local languages (Shona, Ndebele or English) [16]. Writing was the total from the participant writing letters, words and their name in local languages[17].

The *PLUS-EF* is a tablet-based executive function tool, that has been used in school-aged children[18]. The score from multi-source interference (MSIT) was the total that reflected accuracy from the child picking the correct number from different sequences. Stars and Flowers and the Fish Flanker scores was the total in accuracy for the child picking the correct side for different shapes or arrows in different sequences.

Fine motor (FM) testing was calculated as the number of seconds to complete six repetitions of finger-tapping in sequence from the thumb to the 5^th^ finger, divided into dominant and non-dominant hand, with a higher value indicating a longer time, and therefore slower fine motor coordination.

The Strengths and Difficulties Questionnaire (SDQ) consists of 4 subscales with questions asking about emotional, behavioural, attention and relationship problems, with a higher score indicating more difficulties. These four subscales provided the SDQ total. A fifth prosocial subscale asks about positive behaviours[19]. The child socioemotional (CSE) subscale removed the one question on food security, and retained five questions on how the child felt they were supported at home.

For physical function, grip strength (GS) was subdivided into dominant and non-dominant hands, and a standardised score calculated. Broad jump (BJ) and shuttle run (Run) also had a standardised score calculated, so that an overall standardised physical function score was calculated. Blood pressure (BP) was measured at rest, where the pulse pressure was the difference between systolic and diastolic blood pressures. Blood pressure was also measured sequentially 5 times after the shuttle run test. All units were in mm Hg. Finally, raw units of reactance and resistance at 50 kHz were recorded for bioimpedance analysis (BIA).

### Table A. Baseline characteristics of HIV-positive women and their infants enrolled or not enrolled in the follow-up cohort

| **Supplementary table 1** | **Included in follow-up** | **Not included in follow-up** | **p-value** | **Statistical test used** |
| --- | --- | --- | --- | --- |
| **Caregiver assessed, n*** | 267 | 459 |  |  |
| **Children assessed, n** | 273 | 465 |  |  |
| **Household characteristics** |  |  |  |  |
| **Median number of occupants [IQR]** | 4.0 (3.0, 6.0) | 4.0 (3.0, 6.0) | 0.03 | Somers’ D |
| **Wealth quintile, n (%)** |  |  |  |  |
| **Lowest** | 64/258 (24.8%) | 129/453 (28.5%) | 0.49 | Chi-Sq |
| **Second** | 55/258 (21.3%) | 108/453 (23.8%) |  |  |
| **Middle** | 57/258 (22.1%) | 83/453 (18.3%) |  |  |
| **Fourth** | 41/258 (15.9%) | 63/453 (13.9%) |  |  |
| **Highest** | 41/258 (15.9%) | 70/453 (15.5%) |  |  |
| **Electricity** |  |  |  |  |
| **Electricity in home, n (%)** | 6/260 (2.3%) | 13/452 (2.9%) | 0.65 | Chi-Sq |
| **Other electric power, n (%)** |  |  |  |  |
| **Generator** | 9/260 (3.5%) | 9/453 (2.0%) | 0.10 | Chi-Sq |
| **Solar panel** | 171/260 (65.8%) | 269/453 (59.4%) |  |  |
| **Inverter** | 5/260 (1.9%) | 8/453 (1.8%) |  |  |
| **No other type** | 75/260 (28.9%) | 167/453 (36.9%) |  |  |
| **Sanitation** |  |  |  |  |
| **Any latrine at household, n (%)** | 92/255 (36.1%) | 142/447 (31.8%) | 0.28 | Chi-Sq |
| **Improved latrine at household, n (%)** | 79/255 (31.0%) | 124/446 (27.8%) | 0.42 | Chi-Sq |
| **Water** |  |  |  |  |
| **Main source of household drinking water improved, n (%)** | 159/255 (62.4%) | 263/446 (59.0%) | 0.46 | Chi-Sq |
| **Treat drinking water to make it safer, n (%)** | 28/255 (11.0%) | 56/438 (12.8%) | 0.50 | Chi-Sq |
| **One-way walk time to fetch drinking water (min) , median (IQR)** | 10.0 (5.0, 25.0) | 10.0 (5.0, 20.0) | 0.01 | Somers’ D |
| **Per capita water volume collected past 24 hr, median (IQR)** | 6.7 (4.0, 10.0) | 8.6 (5.0, 13.3) | <0.001 | Somers’ D |
| **Hygiene** |  |  |  |  |
| **Handwashing station at household, n (%)** | 35/246 (14.2%) | 33/412 (8.0%) | 0.02 | Chi-Sq |
| **Improved floor, n (%)** | 115/258 (44.6%) | 222/444 (50.0%) | 0.20 | Chi-Sq |
| **Number of chickens, median (IQR)** | 5.0 (2.0, 10.0) | 4.0 (0.0, 8.0) | 0.01 | Somers’ D |
| **Livestock observed inside the house, n (%)** | 103/258 (39.9%) | 140/453 (30.0%) | 0.02 | Chi-Sq |
| **Faeces observed in the yard, n (%)** | 86/256 (33.6%) | 123/450 (27.3%) | 0.09 | Chi-Sq |
| **Diet quality and food security** |  |  |  |  |
| **Household meets minimum dietary diversity score, n (%)** | 85/243 (35.0%) | 151/369 (40.9%) | 0.15 | Chi-Sq |
| **Coping strategies index, median (IQR)** | 3.0 (0.0, 9.0) | 2.0 (0.0, 12.0) | 0.12 | Somers’ D |
| **Maternal characteristics** |  |  |  |  |
| **Mean age (SD), years** | 30.2 (6.1) | 28.6 (6.3) | <0.001 | GEE (robust) |
| **Mean height (SD), cm** | 160.2 (6.2) | 160.2 (6.2) | 0.88 | GEE (robust) |
| **Mean mid-upper-arm circumference (SD), cm** | 26.5 (2.8) | 26.1 (3.00 | 0.01 | GEE (robust) |
| **Mean maternal Hb (SD), g/dL** | 11.5 (1.4) | 11.1 (1.8) | 0.01 | GEE (robust) |
| **Mother meets minimum dietary diversity score, n (%)** | 90/250 (36.0%) | 182/439 (41.5%) | 0.16 | Chi-Sq |
| **Mean years of schooling completed (SD)** | 9.2 (1.9) | 9.1 (2.2) | 0.54 | GEE (robust) |
| **Median parity (IQR)** | 2.0 (1.0, 3.0) | 2.0 (1.0, 3.0) | 0.01 | Somers’ D |
| **Married, n (%)** | 231/248 (93.2%) | 412/434 (94.9%) | 0.71 | Chi-Sq |
| **Employed, n (%)** | 17/260 (6.5%) | 50/450 (11.1%) | 0.04 | Chi-Sq |
| **Religion, n (%)** |  |  |  |  |
| **Apostolic** | 118/252 (46.8%) | 212/437 (48.5%) | 0.85 | Chi-Sq |
| **Other Christian** | 106/252 (42.1%) | 182/437 (41.7%) |  |  |
| **Other religion** | 28/252 (11.1%) | 43/437 (9.8%) |  |  |
| **Maternal capabilities** | | | | |
| **Mean Gender norms attitudes (SD)** | 2.2 (0.8) | 2.2 (0.8) | 0.34 | GEE (robust) |
| **Mean Perceived social support (SD)** | 3.4 (0.7) | 3.5 (0.7) | 0.08 | GEE (robust) |
| **Mean EPDS depression scale (SD)** | 4.7 (5.5) | 3.6 (4.9) | 0.02 | GEE (robust) |
| **Infant characteristics** | | | | |
| **Female, n (%)** | 137/273 (50.2%) | 230/460 (50.0%) | 0.96 | Chi-Sq |
| **Mean birth weight (SD), kg** | 3.0 (0.5) | 3.0 (0.5) | 0.01 | GEE (robust) |
| **Low birthweight, n (%)** | 26/254 (10.2%) | 58/397 (14.6%) | 0.10 | Chi-Sq |
| **Institutional delivery, n (%)** | 216/247 (87.5%) | 328/402 (81.6%) | 0.06 | Chi-Sq |
| **Vaginal delivery, n (%)** | 240/258 (93.0%) | 369/401 (92.0%) | 0.73 | Chi-Sq |
| **Child characteristics at age 18 months** | | | | |
| **Mean LAZ at 18 months, (SD)** | -1.9 (1.1) | -1.9 (1.2) | 0.92 | GEE (robust) |
| **Mean WAZ at 18 months, (SD)** | -1.0 (1.1) | -0.9 (1.1) | 0.51 | GEE (robust) |
| **Mean WHZ at 18 months, (SD)** | -0.1 (1.1) | -0.02 (1.2) | 0.31 | GEE (robust) |
| **Mean HCZ score at 18 months, (SD)** | -0.5 (1.1) | -0.5 (1.2) | 0.90 | GEE (robust) |
| **Mean MUACZ score at 18 months, cm (SD)** | -0.1 (0.9) | -0.2 (0.9) | 0.01 | GEE (robust) |
| **Mean Hb at 18 months, g/dL (SD)** | 11.8 (1.2) | 11.8 (1.2) | 0.79 | GEE (robust) |

**Table A**: **Baseline and early-life characteristics of HIV-positive women and their infants enrolled and not enrolled in the follow-up cohort** n: number, SD: standard deviation, IQR: Inter-quartile range, LAZ: Length-for-age Z-score, WAZ: Weight-for-age Z-score, WHZ: Weight-for-height Z-score, HCZ : Head circumference for Z-score, MUACZ: Mid-upper arm circumference Z-score, Hb: Haemoglobin, Chi-Sq: Chi -Square from logistic regression and adjusted for clustering . GEE (robust) Generalised estimating equations with robust variance estimation adjusted for clustering, Somers’ D comparison of medians using t-distribution adjusted for clustering.

*Of the 267 caregivers, data were not available for 2 women at baseline entry to the trial, and for 7 of the not included group.

Applying a Bonferroni correction for multiple comparisons (40), a P value <0.001 is considered significant.

### Table B. Baseline characteristics of HIV-negative women and their infants enrolled or not enrolled in the follow-up cohort

| **Supplementary table 2** | **CHU included in follow-up** | **CHU not included in follow-up** | **p-value** | **Statistical test used** |
| --- | --- | --- | --- | --- |
| **Caregiver assessed, N*** | 988 | 2949 |  |  |
| **Children assessed, N** | 1002** | 2987 |  |  |
| **Household characteristics** | | | | |
| **Median number of occupants [IQR]** | 5.0 (4.0, 6.0) | 5.5 (3.0, 6.0) | 0.09 | Somers’ D |
| **Wealth quintile, n (%)** |  |  |  |  |
| **Lowest** | 164/914 (17.9%) | 511/2744 (18.6%) | 0.84 | Chi-Sq |
| **Second** | 168/914 (18.4%) | 544/2744 (19.8%) |  |  |
| **Middle** | 186/914 (20.4%) | 553/2744 (20.2%) |  |  |
| **Fourth** | 200/914 (21.9%) | 576/2744 (21.0%) |  |  |
| **Highest** | 196/914 (21.4%) | 560/2744 (20.4%) |  |  |
| **Electricity** |  |  |  |  |
| **Electricity in home, n (%)** | 31/914 (3.4%) | 69/2738 (2.5%) | 0.18 | Chi-Sq |
| **Other electric power, n (%)** |  |  |  |  |
| **Generator** | 32/914 (3.5%) | 86/2743 (3.1%) | 0.21 | Chi-Sq |
| **Solar panel** | 631/914 (69.0%) | 1795/2743 (65.4%) |  |  |
| **Inverter** | 13/914 (1.4%) | 44/ 2743 (1.6%) |  |  |
| **No other type** | 238/914 (26.0%) | 818/2743 (29.8%) |  |  |
| **Sanitation** | | | | |
| **Any latrine at household, n (%)** | 343/899 (38.2%) | 981/2707 (36.2%) | 0.42 | Chi-Sq |
| **Improved latrine at household, n (%)** | 290/898 (32.3%) | 867/2703 (32.1%) | 0.92 | Chi-Sq |
| **Water** |  |  |  |  |
| **Main source of household drinking water improved, n (%)** | 618/900 (68.7%) | 1675/2725 (61.5%) | 0.01 | Chi-Sq |
| **Treat drinking water to make it safer, n (%)** | 127/895 (14.2%) | 322/2673 (12.1%) | 0.12 | Chi-Sq |
| **One-way walk time to fetch drinking water (min) , median (IQR)** | 10.0 (5.0, 20.0) | 10.0 (5.0, 20.0) | 0.30 | Somers’ D |
| **Per capita water volume collected past 24 hr, median (IQR)** | 6.7 (4.4, 10.0) | 7.5 (5.0, 12.0) | <0.001 | Somers’ D |
| **Hygiene** | | | | |
| **Handwashing station at household, n (%)** | 101/876 (11.5%) | 207/2557 (8.1%) | 0.01 | Chi-Sq |
| **Improved floor, n (%)** | 484/900 (53.8%) | 1512/2707 (55.9%) | 0.44 | Chi-Sq |
| **Number of chickens, median (IQR)** | 6.0 (2.0, 10.0) | 6.0 (2.0, 10.0) | 0.12 | Somers’ D |
| **Livestock observed inside the house, n (%)** | 390/963 (40.5%) | 1039/2886 (36.0%) | 0.02 | Chi-Sq |
| **Faeces observed in the yard, n (%)** | 322/958 (33.6%) | 877/2879 (30.5%) | 0.15 | Chi-Sq |
| **Diet quality and food security** |  |  |  |  |
| **Household meets minimum dietary diversity score, n (%)** | 328/880 (37.3%) | 965/2344 (41.2%) | 0.10 | Chi-Sq |
| **Coping strategies index, median (IQR)** | 0.0 (0.0, 6.0) | 1.0 (0.0, 7.0) | 0.01 | Somers’ D |
| **Maternal characteristics** | | | | |
| **Mean age (SD), years** | 25.7 (6.3) | 25.6 (6.7) | 0.15 | GEE (robust) |
| **Mean height (SD), cm** | 159.9 (6.0) | 160.2 (5.6) | 0.08 | GEE (robust) |
| **Mean mid-upper-arm circumference (SD), cm** | 26.6 (3.3) | 26.3 (3.0) | 0.05 | GEE (robust) |
| **Maternal Hb, g/dL (SD)** | 12.2 (1.4) | 12.1 (1.5) | 0.21 | GEE (robust) |
| **Mother meets minimum dietary diversity score, n (%)** | 330/901 (36.6%) | 1081/2675 (40.4%) | 0.10 | Chi-Sq |
| **Mean years of schooling completed (SD)** | 9.7 (1.7) | 9.6 (1.8) | 0.10 | GEE (robust) |
| **Median parity (IQR)** | 2.0 (1.0, 3.0) | 2.0 (1.0, 3.0) | 0.03 | Somers’ D |
| **Married, n (%)** | 889/935 (95.1%) | 2657/2782 (95.5%) | 0.29 | Chi-Sq |
| **Employed, n (%)** | 69/916 (7.5%) | 242/2739 (8.8%) | 0.23 | Chi-Sq |
| **Religion, n (%)** |  |  |  |  |
| **Apostolic** | 455/941 (48.4%) | 1308/2804 (46.7%) | 0.097 | Chi-Sq |
| **Other Christian** | 395/941 (42.0%) | 1289/2804 (46.0%) |  |  |
| **Other religion** | 91/941 (9.7%) | 207/2804 (7.4%) |  |  |
| **Maternal capabilities** |  |  |  |  |
| **Mean Gender norms & attitudes (SD)** | 2.3 (0.8) | 2.2 (0.8) | <0.001 | GEE (robust) |
| **Mean Perceived social support (SD)** | 3.5 (0.6) | 3.6 (0.6) | 0.17 | GEE (robust) |
| **Mean EPDS depression scale (SD)** | 2.8 (4.0) | 2.9 (4.2) | 0.01 | GEE (robust) |
| **Infant characteristics** |  |  |  |  |
| **Female, n (%)** | 511/1002 (51.0%) | 1451/2972 (48.8%) | 0.28 | Chi-Sq |
| **Mean birth weight (SD), kg** | 3.1 (0.5) | 3.1 (0.5) | 0.43 | GEE (robust) |
| **Low birthweight, n (%)** | 90/957 (9.4%) | 235/2616 (9.0%) | 0.74 | Chi-Sq |
| **Institutional delivery, n (%)** | 855/935 (91.4%) | 2353/2669 (88.2%) | 0.01 | Chi-Sq |
| **Vaginal delivery, n (%)** | 909/965 (94.2%) | 2482/2699 (92.0%) | 0.10 | Chi-Sq |
| **18 month endpoint characteristics** | | | | |
| **Mean LAZ at 18 months, (SD)** | -1.5 (1.0) | -1.5 (1.1) | 0.18 | GEE (robust) |
| **Mean WAZ at 18 months, (SD)** | -0.7 (1.0) | -0.7 (1.0) | 0.06 | GEE (robust) |
| **Mean WHZ at 18 months, (SD)** | -0.04 (1.0) | 0.1 (1.1) | 0.001 | GEE (robust) |
| **Mean Head circumference Z score at 18 months, (SD)** | -0.2 (1.1) | -0.2 (1.5) | 0.48 | GEE (robust) |
| **Mean MUAC Z scoe at 18 months (SD)** | 0.1 (0.9) | 0.02 (0.9) | 0.03 | GEE (robust) |
| **Mean Hb at 18 months, g/dL (SD)** | 11.8 (1.1) | 11.7 (1.2) | 0.12 | GEE (robust) |

**Table B** n: number, SD: standard deviation, IQR: Inter-quartile range, LAZ: Length-for-age Z-score, WAZ: Weight-for-age Z-score, WHZ: Weight-for-height Z-score, HCZ : Head circumference for Z-score, MUACZ: Mid-upper arm circumference Z-score, Hb: Haemoglobin, Chi-Sq: Chi -Square from logistic regression and adjusted for clustering . GEE (robust) Generalised estimating equations with robust variance estimation adjusted for clustering, Somers’ D comparison of medians using t-distribution adjusted for clustering. There were less mothers than children due to some households having twins.

*Note that of the 988 HIV negative mothers recruited into SHINE follow-up, data were not available for 66 mothers at the baseline visit and similarly for the 2949 HIV negative mothers not recruited, data were not available for 182 mothers at the baseline visit.

**Note that 1002 children included two children who were HIV positive as their mothers seroconverted during breastfeeding and the children became HIV positive. These two children were excluded from all analyses.

Applying a Bonferroni correction for multiple comparisons (40), a P value <0.001 is considered significant.

### Table C: Baseline characteristics of children born HIV-free (CBHF) and children HIV-unexposed (CHU)

|  | **CBHF** | **CHU** | **p-value** | **Statistical test used** |
| --- | --- | --- | --- | --- |
| **Maternal characteristics in pregnancy** | | | | |
| **Mean age (SD), years** | 30.3 (6.0) | 25.7 (6.3) | <0.001 | GEE (Robust) |
| **Mean height (SD), cm** | 160.4 (6.1) | 160.0 (6.0) | 0.35 | GEE (Robust) |
| **Mean MUAC (SD), cm** | 26.6 (2.8) | 26.6 (3.2) | 0.99 | GEE (Robust) |
| **Mean haemoglobin (SD), g/dl** | 11.5 (1.4) | 12.2 (1.4) | <0.001 | GEE (Robust) |
| **Mean years of schooling (SD)** | 9.1 (1.9) | 9.7 (1.7) | <0.001 | GEE (Robust) |
| **Median parity (IQR)** | 2 (1, 3) | 2 (1, 3) | <0.001 | GEE (Robust) |
| **Married, % [n]** | 93.5% [229/245] | 95.2% [893/938] | 0.45 | Chi-Sq |
| **Employed, % [n]** | 6.6% [17/256] | 7.5% [69/919] | 0.64 | Chi-Sq |
| **Religion, % [n]** |  |  |  |  |
| **Apostolic, % [n]** | 45.6% [113] | 48.1% [454] | 0.60 | Chi-Sq |
| **Other Christian** | 42.7% [106] | 42.0% [398] |  |  |
| **Other non-Christian** | 11.7% [29] | 9.8% [92] |  |  |
| **Maternal capabilities** |  |  |  |  |
| **Mean Gender norms (SD)** | 2.2 (0.8) | 2.3 (0.8) | 0.16 | GEE (Robust) |
| **Mean perceived social support (SD)** | 3.4 (0.7) | 3.5 (0.6) | 0.03 | GEE (Robust) |
| **Mean Edinburgh Postnatal Depression Score (SD)** | 4.6 (5.6) | 2.8 (4.0) | <0.001 | GEE (Robust) |
| **Maternal HIV disease severity and treatment** | | | | |
| **Mean CD4 count in pregnancy, cells/uL (SD)** | 474.3 (SD 214.6)  [n=210] | N/A |  |  |
| **Documented antiretroviral therapy during pregnancy** | 82.8% [226/273] | N/A |  |  |
| **Tenofovir disoproxil fumarate-based ART regimen** | 69.9% [158/226] | N/A |  |  |
| **Zidovudine based ART regimen** | 18.6% [42/226] | N/A |  |  |
| **Other/unknown ART regimen** | 11.5% [26/226] | N/A |  |  |
| **Documented co-trimoxazole prophylaxis during pregnancy** | 66.7% [182/273] | N/A |  |  |
|  |  |  |  |  |
| **Household characteristics in pregnancy** | | | | |
| **Median household size (IQR)** | 4 (3, 6) | 5 (4, 6) | 0.08 | Somers’ D |
| **Median Coping Strategies Index (IQR)** | 3 (0, 9) | 0 (0, 9) | <0.001 | Somers’ D |
| **Baseline wealth quintile, % [n]** |  |  |  |  |
| **lowest** | 25.2% [64 /254] | 17.7% [162 / 917] | 0.007 | Chi-Sq |
| **second** | 21.7% [55 / 254] | 18.4% [169 / 917] |  |  |
| **middle** | 21.7% [55 / 254] | 20.5% [188 / 917] |  |  |
| **fourth** | 15.0% [38 / 254] | 21.7% [199 / 917] |  |  |
| **highest** | 16.5% [42 / 254] | 21.7% [199 / 917] |  |  |
| **Electricity in house, % [n]** | 2.3% [6 / 256] | 3.4% [31 / 917] | 0.40 | Chi-Sq |
| **Any latrine, % [n]** | 36.3% [91 / 251] | 38.0% [343 / 902] | 0.61 | Chi-Sq |
| **Improved latrine, % [n]** | 30.7% [77 / 251] | 32.1% [289 / 901] | 0.67 | Chi-Sq |
| **Improved water source, % [n]** | 61.8% [155 / 251] | 68.3% [617 / 903] | 0.05 | Chi-Sq |
| **Treat drinking water in any way, % [n]** | 11.2% [28 / 251] | 14.5% [130 / 898] | 0.18 | Chi-Sq |
| **HH meets min dietary diversity, % [n]** | 36.4% [87 / 239] | 37.7% [333 / 884] | 0.72 | Chi-Sq |
| **Women meet min dietary diversity score, % [n]** | 37.4% [92 / 246] | 36.8% [333/ 904] | 0.87 | Chi-Sq |
| **Child Variables** | | | |  |
|  | CBHF | CHU | p-value |  |
| **Mean Birthweight, Kg (SD)** | 3.03 (0.46) | 3.10 (0.47) | 0.06 | GEE (Robust) |
| **Proportion Low Birthweight, % [n]** | 10.2% [25 / 246] | 9.2% s[87 / 945] | 0.65 | Chi-Sq |
| **Proportion Institutional delivery, % [n]** | 87.0% [207 / 238] | 91.4% [844 / 923] | 0.04 | Chi-Sq |
| **Proportion vaginal delivery, % [n]** | 93.2% [232 / 249] | 94.4% [900 / 953] | 0.45 | Chi-Sq |
| **Mean breastfeeding duration, months (SD)** | 17.7 (4.5) | 19.1 (3.7) | <0.001 | GEE (Robust) |
| **Mean 18-month LAZ (SD)** | -1.83 (1.06) | -1.50 (1.02) | <0.001 | GEE (Robust) |
| **Stunted at 18 months, % [n]** | 45.2% [118 / 261] | 29.6% [290 / 981] | <0.001 | Chi-Sq |
| **Mean 18-month WAZ (SD)** | -0.96 (1.05) | -0.74 (0.98) | 0.003 | GEE (Robust) |
| **Underweight at 18 months, % [n]** | 16.9% [44 / 261] | 8.8% [86 / 981] | <0.001 | Chi-Sq |
| **Mean 18-month WHZ (SD)** | -0.11 (1.06) | -0.03 (1.00) | 0.28 | GEE (Robust) |
| **Mean 18-month HCZ (SD)** | -0.46 (1.07) | -0.21 (0.98) | 0.001 | GEE (Robust) |
| **Mean 18-month MUACZ (SD)** | -0.06 (0.87) | 0.10 (0.87) | 0.01 | GEE (Robust) |
| **Mean 18-month Hb, g/dL (SD)** | 11.8 (1.1) | 11.8 (1.1) | 0.93 | GEE (Robust) |

**Table C** Baseline household, maternal and child characteristics between children born HIV-free (CBHF) compared with children who were HIV-unexposed (CHU). Note that household and maternal characteristics were all measured in pregnancy at the baseline visit. Child characteristics were measured at birth for birth outcomes, and when the child was 18 months old for the growth outcomes specified.

**Table D Interaction analysis by sex of children**

| **Variable** | **P-value of interaction of HIV-exposure with sex pf children** | **GEE Coefficient for Girls (95% CI)** | **GEE coefficient for Boys (95% CI)** |
| --- | --- | --- | --- |
| **Mental Processing Index** | 0.20 | N/A | N/A |
| **School Achievement Test** | 0.99 | N/A | N/A |
| **Plus EF test score** | 0.87 | N/A | N/A |
| **Fine motor, seconds** | 0.58 | N/A | N/A |
| **Strengths and Difficulties Questionnaire** | 0.032 | 0.3 (-0.6, 1.2) | -1.2 (-0.2, -2.3) |
| **Child socioemotional score** | 0.050 | 0.1 (-0.0, 0.2) | -0.1 (-0.2, 0.0) |
| **Mean Grip Strength, Kg** | 0.47 | N/A | N/A |
| **Mean Broad jump, m** | 0.61 | N/A | N/A |
| **VO2max, ml kg^-1^ min^-1^** | 0.092 | 0.4 (0.0, 0.8) | 1.1 (0.4, 1.7) |
| **Diastolic BP, mm Hg** | 0.89 | N/A | N/A |
| **Systolic BP, mm Hg** | 0.90 | N/A | N/A |
| **Height-for-age Z-score** | 0.37 | N/A | N/A |
| **Weight-for-age Z-score** | 0.99 | N/A | N/A |
| **BMI Z-score** | 0.30 | N/A | N/A |
| **Knee-heel length** | 0.63 | N/A | N/A |
| **Head circ, cm** | 0.72 | N/A | N/A |
| **MUAC, cm** | 0.24 | N/A | N/A |
| **Waist circ, cm** | 0.97 | N/A | N/A |
| **Hip circ, cm** | 0.37 | N/A | N/A |
| **Calf circ, cm** | 0.052 | 0.3 (-0.0, 0.6) | -0.2 (-0.5, 0.2) |
| **Lean mass index** | 0.41 | N/A | N/A |
| **Impedance Index** | 0.84 | N/A | N/A |
| **Phase angle** | 0.45 | N/A | N/A |
| **Total skinfold thicknesses, mm** | 0.19 | N/A | N/A |
| **Peripheral skinfold thickness, mm** | 0.21 | N/A | N/A |
| **Central skinfold thickness, mm** | 0.29 | N/A | N/A |
| **Hb, g dl^-1^** | 0.30 | N/A | N/A |

**Table D: Results of subgroup analysis exploring interaction of sex of children with HIV-exposure for SAHARAN toolbox outcomes**. If the p-value was greater than 0.1, the interaction was not considered significant, hence N/A (not applicable) was completed for the difference between boys and girls.

### Table E: Cognition subtests & secondary physical function outcomes compared between Children born HIV-free (CBHF) and Children HIV-unexposed (CHU)

|  | **Outcome** | **CBHF** | | **CHU** | | **GEE Mean difference (95% CI)** | | | |
| --- | --- | --- | --- | --- | --- | --- | --- | --- | --- |
|  | **Cognitive subtests** | **N** | **Mean (SD)** | **N** | **Mean (SD)** | **Unadjusted** | **Adjusted difference**  **Model 1 (Trial factors)** | **Adjusted difference**  **Model 2 (Trial factors & contemporary covariates)** | **Adjusted difference**  **Model 3 (Trial factors & baseline covariates)** |
| **KABC-II domain & subtest** | **Atlantis** | 264 | 6 (2) | 990 | 6 (2) | 0 (0, 1) | 0 (0, 1) | 0 (0, 1) | 0 (0, 1) |
|  | **Story completion** | 264 | 4 (2) | 990 | 5 (2) | 0 (0, 0) | 0 (0, 0) | 0 (0, 0) | 0 (0, 0) |
|  | **Number recall** | 264 | 7 (2) | 990 | 7 (2) | 0 (0, 1) | 0 (0, 1) | 0 (0, 0) | 0 (0, 1) |
|  | **Atlantis delayed** | 264 | 7 (2) | 990 | 7 (2) | 0 (0, 1) | 0 (0, 1) | 0 (0, 1) | 0 (0, 1) |
|  | **Rover** | 264 | 7 (2) | 990 | 7 (2) | 0 (0, 1) | 0 (0, 1) | 0 (0, 1) | 0 (0, 1) |
|  | **Triangles** | 264 | 4 (2) | 990 | 4 (2) | 0 (0, 1) | 0 (0, 1) | 0 (0, 1) | 0 (0, 1) |
|  | **Word Order** | 264 | 5 (2) | 990 | 6 (2) | 0 (0, 1) | 0 (0, 1) | 0 (0, 1) | 0 (0, 1) |
|  | **Pattern reasoning** | 264 | 5 (2) | 990 | 6 (3) | 1 (0, 1) | 1 (0, 1) | 0 (0, 1) | 0 (0, 1) |
|  | **Learning (domain)** | 264 | 13 (3) | 990 | 13 (4) | 1 (0, 1) | 1 (0, 1) | 1 (0, 1) | 1 (0, 1) |
|  | **Planning (domain)** | 264 | 10 (3) | 990 | 11 (4) | 1 (0, 1) | 1 (0, 1) | 0 (0, 1) | 1 (0, 1) |
|  | **Simultaneous (domain)** | 264 | 10 (3) | 990 | 11 (4) | 1 (0, 1) | 1 (0, 1) | 1 (0, 1) | 1 (0, 1) |
|  | **Sequential (domain)** | 264 | 12 (4) | 990 | 13 (4) | 1 (0, 1) | 1 (0, 1) | 1 (0, 1) | 1 (0, 1) |
| **SAT** | **Numeracy** | 264 | 17 (6) | 990 | 18 (6) | 2 (1, 2) | 2 (1, 2) | 1 (0, 2) | 1 (0, 2) |
|  | **Reading** | 264 | 10 (12) | 990 | 14 (13) | 3 (2, 5) | 3 (1, 5) | 2 (1, 4) | 3 (1, 4) |
|  | **Writing** | 264 | 12 (9) | 990 | 14 (10) | 2 (1, 3) | 2 (1, 3) | 2 (0, 3) | 2 (0, 3) |
| **Plus-EF** | **Multi-source interference test (MSIT)** | 251 | 22 (11) | 978 | 24 (11) | 2 (1, 4) | 2 (1, 3) | 2 (0, 3) | 2 (0, 3) |
|  | **Stars and Flowers** | 251 | 41 (10) | 978 | 43 (9) | 2 (0, 3) | 2 (0, 3) | 1 (0, 3) | 2 (0, 3) |
|  | **Fish flanker** | 251 | 46 (12) | 978 | 47 (12) | 1 (0, 3) | 1 (-1, 3) | 1 (-1, 3) | 1 (-1, 3) |
| **FM** | **Finger tapping (dominant), sec** | 262 | 24 (7) | 986 | 23 (7) | -1 (-1.8, -0.2) | -1.1 (-1.9, -0.2) | -0.6 (-1.4, 0.3) | -0.8 (-1.6, 0.0) |
|  | **Finger tapping (non-dominant), sec** | 262 | 26 (7) | 986 | 25 (7) | -0.9 (-1.9, 0) | -1 (-2, 0) | -0.5 (-1.5, 0.5) | -0.7 (-1.7, 0.4) |
| **SDQ** | **Emotional** | 263 | 2 (2) | 989 | 2 (2) | 0 (0, 0) | 0 (0, 0) | 0 (0, 0) | 0 (0, 0) |
|  | **Conduct** | 263 | 2 (2) | 989 | 2 (2) | 0 (0, 0) | 0 (0, 0) | 0 (0, 0) | 0 (0, 0) |
|  | **Hyperactivity / inattention** | 263 | 4 (2) | 989 | 4 (2) | 0 (0, 0) | 0 (0, 0) | 0 (0, 0) | 0 (0, 0) |
|  | **Peer relationships** | 263 | 1 (1) | 989 | 1 (1) | 0 (0, 0) | 0 (0, 0) | 0 (0, 0) | 0 (0, 0) |
|  | **Prosocial** | 263 | 8 (2) | 989 | 8 (2) | 0 (0, 0) | 0 (0, 0) | 0 (0, 0) | 0 (0, 0) |
| **CSE** | **Child socioemotional (removing food security question)** | 256 | 4 (1) | 973 | 4 (1) | 0 (0, 0) | 0 (0, 0) | 0 (0, 0) | 0 (0, 0) |
|  | **Physical function** |  |  |  |  |  |  |  |  |
| **Grip** | **Grip strength dominant hand, Kg** | 262 | 10.6 (2.1) | 990 | 10.8 (2.0) | 0.2 (-0.1, 0.5) | 0.3 (0.0, 0.6) | 0.3 (0.0, 0.6) | 0.1 (-0.2, 0.4) |
|  | **Grip strength non-dominant hand, Kg** | 262 | 10.4 (1.9) | 990 | 10.6 (2.1) | 0.2 (-0.1, 0.5) | 0.2 (0.0, 0.5) | 0.2 (-0.1, 0.5) | 0.0 (-0.3, 0.3) |
|  | **Standardised Grip strength (a)** | 262 | -0.1 (1) | 990 | 0.0 (1.0) | 0.1 (0.0, 0.2) | 0.1 (0.0, 0.2) | 0.1 (0.0, 0.3) | 0.0 (-0.1, 0.2) |
| **BJ** | **Standardised Broad jump (b)** | 259 | -0.1 (1.1) | 987 | 0.0 (1.0) | 0.1 (0.0, 0.3) | 0.1 (0.0, 0.3) | 0.1 (0.0, 0.3) | 0.1 (-0.1, 0.2) |
| **Run** | **Standardised VO2max (c)*** | 255 | -0.2 (1) | 986 | 0.1 (1.0) | 0.3 (0.1, 0.4) | 0.2 (0.0, 0.3) | 0.2 (0.0, 0.3) | 0.1 (0.0, 0.3) |
| **Tot** | **Physical function (a)+(b)+(c)** | 254 | -0.3 (2) | 984 | 0.1 (2.1) | 0.4 (0.1, 0.7) | 0.4 (0.1, 0.6) | 0.4 (0.1, 0.6) | 0.2 (-0.1, 0.4) |
| **Blood pressure** | **Resting pulse pressure, mm Hg** | 264 | 34 (7) | 988 | 35 (7) | 1 (0, 2) | 1 (0, 2) | 0 (0, 1) | 0 (0, 1) |
|  | **Systolic BP 1 min after SRT, mm Hg** | 256 | 125 (11) | 976 | 127 (11) | 1 (0, 3) | 1 (-1, 3) | 1 (-1, 2) | 1 (-1, 2) |
|  | **Diastolic BP 1 min after SRT, mm Hg** | 256 | 85 (12) | 985 | 86 (11) | 1 (-1, 3) | 1 (-1, 3) | 1 (-1, 2) | 1 (-1, 2) |
|  | **Pulse pressure 1 min after SRT, mm HG** | 256 | 39 (9) | 985 | 40 (9) | 1 (0, 2) | 1 (-1, 3) | 0 (-1, 1) | 0 (-1, 1) |
|  | **Difference between 1^st^ & 5^th^ systolic BP measurements, mm Hg** | 256 | 22 (9) | 975 | 22 (8) | 0 (-1, 1) | 0 (-1, 1) | 0 (-1, 1) | 0 (-1, 1) |
|  | **Difference between 1^st^ & 5^th^ diastolic BP measurements, mm Hg** | 256 | 19 (9) | 984 | 19 (8) | 0 (-1, 1) | 0 (-1, 1) | 0 (-1, 1) | 0 (-2, 1) |
|  | **Growth** |  |  |  |  |  |  |  |  |
| **BIA** | **Reactance at 50 kHz, Ohms** | 261 | 73.5 (10.3) | 981 | 71.7 (10.5) | -1.7 (-3.4, 0) | -1.3 (-2.7, 0.1) | -1.4 (-2.8, 0.0) | -1.3 (-2.7, 0.2) |
|  | **Resistance at 50 kHz, Ohms** | 262 | 833 (95) | 986 | 830 (94) | -2.9 (-18.3, 12.5) | -3.1 (-16.1, 9.9) | -3.5 (-16.0, 9.1) | 0 (-14.8, 14.7) |

**Table E**. **Secondary outcomes comparing Children born HIV-free (CBHF) and Children unexposed to HIV (CHU).** Cognitive function included the Kaufman Assessment Battery for Children (KABC-II) with its 8 subtests Atlantis, Story completion, Number recall, Delayed Atlantis, Rover, Triangles, Word Order and Pattern reasoning. 2 from each of these 8 subtests were added together to form the 4 cognitive domains of Sequential, Planning, Learning and Simultaneous domains. The School Achievement Test (SAT) was formed of numeracy, reading and writing sections. The Plus-EF total was formed of 3 subtests Multi-Source Interference Test (MSIT), Stars and Flowers and Fish Flanker. The Fine motor (FM) test was measured by sequential finger tapping for both dominant and non-dominant hands, using seconds as a unit and hence a higher number represented slower fine motor coordination. The Strength and Difficulties Questionnaire (SDQ) total was measured using 4 subscales of emotional, conduct, hyperactivity and inattention with higher scores representing more difficulties. In addition the prosocial subscale was separately measured for positive behaviour. The child socioemotional subscore was the total with one question removed on food security. Grip strength (GS) was measured with the highest value for both dominant and non-dominant hands. Standardised scores were included for broad jump (BJ) and the shuttle run test (Run). The total of the standardised scores provided the physical function score. Blood pressure included pulse pressure as the difference between systolic and diastolic, and included values measured after the shuttle run test. Bioimpedance (BIA) measured raw values of reactance and resistance in Ohms.

Model 1 is adjusted for trial factors (arm, study nurse, exact child age, calendar month recruited, temperature, sex). Model 2 is adjusted for trial factors from Model 1 and contemporary factors (socioeconomic status, caregiver depression score (EPDS), household food insecurity (HFIAS), household religion, caregiver social support, caregiver gender norms, caregiver age, caregiver education, adversity score, children’s books at home). Model 3 is adjusted for trial factors from Model 1 and early-life factors (length for age Z-score (LAZ) at 18mo, birthweight, maternal baseline depression score (EPDS), household diet, maternal haemoglobin, socioeconomic status, facility birth, gender norms, and maternal years of schooling).

*Note that standardised VO_2_max score was used as this represented the distribution of shuttle run better by adjusting for differences between the number of sub-levels for each stage of the shuttle run test

### Table F Summary of findings

| **Overall findings following Bonferroni correction for children born HIV-free (CBHF) compared to those unexposed to HIV (p<0.002)** | | | | | | |
| --- | --- | --- | --- | --- | --- | --- |
|  | **Variable** | **Effect size (SD)**  **Unadjusted (p)** | **Adjusted for contemporary covariates (p)** | **Adjusted for baseline covariates (p)** | **Subtests & child sex** | **Interpretation** |
| **Cognition** | Mental processing index (MPI) | CBHF 0.28 SD lower, p<0.001 | <0.001 | <0.001 | CBHF scored lower in all subtests for both sexes | Evidence that CBHF have globally reduced cognitive processing across all subtests, even when adjusting for contemporary or baseline covariates |
|  | School Achievement Test (SAT) | CBHF 0.24 SD lower, p<0.001 | 0.002 | 0.001 | CBHF scored lower in all subtests for both sexes | Evidence that CBHF have globally reduced academic function across literacy and numeracy, even when adjusting for contemporary or baseline covariates |
|  | Plus EF Executive function test (Plus EF) | CBHF 0.21 SD lower, p=0.001 | 0.004 | 0.002 | CBHF scored lower except in Flanker test, for both sexes | Some evidence that CBHF have reduced executive function (significant in unadjusted models only) |
|  | Fine motor | CBHF 0.14 SD slower, p=0.022 | 0.076 | 0.11 | No overall evidence of difference | No overall evidence of difference in fine motor function following Bonferroni correction |
|  | No evidence of difference for socioemotional function measured by Strengths and difficulties Questionnaire or child socioemotional score overall. | | | | Weak evidence that CBHF boys may have worse SDQ score | No overall evidence of difference for socioemotional function. Subgroup analysis provides weak evidence that CBHF boys may score worse for SDQ. |
| **Physical** | Maximal Oxygen consumption (VO_x_max) from shuttle run test | CBHF 0.26 SD lower, p<0.001 | 0.009 | 0.015 | Weak evidence that CBHF boys may have lower VO2max | Some evidence that CBHF have reduced cardiovascular fitness (significant in unadjusted models only) |
|  | No evidence of difference for grip strength, broad jump or blood pressure | | | | | |
| **Growth** | Head circumference | CBHF 0.20 SD smaller, p=0.009 | 0.003 | 0.002 | Both sexes affected | Weak evidence that CBHF may have reduced head circumference in unadjusted models only |
|  | No evidence of difference for any other measure of growth or body composition | | | |  |  |

**Table F: summary of findings for Children born HIV-free (CBHF) compared with Children HIV-unexposed (CHU) at 7 years.** This table summarises the associations observed, SD: Standard deviation

**Supporting information references**

1. Chasekwa B, Maluccio JA, Ntozini R, Moulton LH, Wu F, Smith LE, et al. Measuring wealth in rural communities: Lessons from the Sanitation, Hygiene, Infant Nutrition Efficacy (SHINE) trial. PLoS One. 2018;13(6):e0199393. doi: 10.1371/journal.pone.0199393.

2. Maxwell D, Caldwell R. The coping strategies index: field methods manual. Cooperative for Assistance and Relief Everywhere Inc. USA. 2008.

3. Chibanda D, Mangezi W, Tshimanga M, Woelk G, Rusakaniko P, Stranix-Chibanda L, et al. Validation of the Edinburgh Postnatal Depression Scale among women in a high HIV prevalence area in urban Zimbabwe. Arch Womens Ment Health. 2010;13(3):201-6. Epub 2009/09/18. doi: 10.1007/s00737-009-0073-6. PubMed PMID: 19760051.

4. Matare CR, Mbuya MNN, Pelto G, Dickin KL, Stoltzfus RJ, Sanitation Hygiene Infant Nutrition Efficacy Trial T. Assessing Maternal Capabilities in the SHINE Trial: Highlighting a Hidden Link in the Causal Pathway to Child Health. Clin. Infect. Dis. 2015;61 Suppl 7(Suppl 7):S745-S51. Epub 11/11. doi: 10.1093/cid/civ851. PubMed PMID: 26602303.

5. Tome J, Mbuya MNN, Makasi RR, Ntozini R, Prendergast AJ, Dickin KL, et al. Maternal caregiving capabilities are associated with child linear growth in rural Zimbabwe. Matern. Child Nutr. 2021;17(2):e13122. doi: https://doi.org/10.1111/mcn.13122.

6. Bhopal S, Roy R, Verma D, Kumar D, Avan B, Khan B, et al. Impact of adversity on early childhood growth & development in rural India: Findings from the early life stress sub-study of the SPRING cluster randomised controlled trial (SPRING-ELS). PLoS One. 2019;14(1):e0209122. doi: 10.1371/journal.pone.0209122.

7. Piper JD, Mazhanga C, Mapako G, Mapurisa I, Mashedze T, Munyama E, et al. Characterising school-age health and function in rural Zimbabwe using the SAHARAN toolbox. PLoS One. 2023;18(5):e0285570. doi: 10.1371/journal.pone.0285570.

8. Driscoll KS, Pianta RC. Mothers’ and fathers’ perceptions of conflict and closeness in parent-child relationships during early childhood. Psychology. 2011.

9. Straus MA, Hamby SL, Finkelhor D, Moore DW, Runyan D. Identification of child maltreatment with the Parent-Child Conflict Tactics Scales: development and psychometric data for a national sample of American parents. Child Abuse Negl. 1998;22(4):249-70. Epub 1998/05/20. doi: 10.1016/s0145-2134(97)00174-9. PubMed PMID: 9589178.

10. ZIMSTAT, UNICEF. Zimbabwe Multi-Indicator Cluster Survey 2019; Snapshots of Key Findings. https://www.unicef.org/zimbabwe/: [Accessed 2019 9/9/2023]. Report No.

11. Salvador Castell G, Pérez Rodrigo C, Ngo de la Cruz J, Aranceta Bartrina J. Household food insecurity access scale (HFIAS). Nutr Hosp. 2015;31 Suppl 3:272-8. Epub 20150226. doi: 10.3305/nh.2015.31.sup3.8775. PubMed PMID: 25719795.

12. Gandure S, Drimie S, Faber M. Food Security Indicators after Humanitarian Interventions Including Food Aid in Zimbabwe. Food Nutr. Bull. 2010;31(4):513-23. doi: 10.1177/156482651003100405.

13. Bhalla G, Handa S, Angeles G, Seidenfeld D. The effect of cash transfers and household vulnerability on food security in Zimbabwe. Food Policy. 2018;74:82-99. doi: 10.1016/j.foodpol.2017.11.007.

14. Young SL, Boateng GO, Jamaluddine Z, Miller JD, Frongillo EA, Neilands TB, et al. The Household Water InSecurity Experiences (HWISE) Scale: development and validation of a household water insecurity measure for low-income and middle-income countries. BMJ Glob Health. 2019;4(5):e001750. Epub 2019/10/23. doi: 10.1136/bmjgh-2019-001750. PubMed PMID: 31637027; PubMed Central PMCID: PMCPMC6768340.

15. Adaptation of the Kaufman Assessment Battery for Children—2nd edition (KABC-II) to assess school-age neurodevelopment in rural Zimbabwe [Internet]. 2022. Available from: osf.io/ybct4

16. Gove A, Brunette T, Bulat J, Carrol B, Henny C, Macon W, et al. Assessing the Impact of Early Learning Programs in Africa. New directions for child and adolescent development. 2017;2017(158):25-41. doi: 10.1002/cad.20224. PubMed PMID: 29243385.

17. Puranik CS, Lonigan CJ. Name-writing proficiency, not length of name, is associated with preschool children's emergent literacy skills. Early Child Res Q. 2012;27(2):284-94. Epub 2011/09/21. doi: 10.1016/j.ecresq.2011.09.003. PubMed PMID: 22523450.

18. Obradović J, Sulik MJ, Finch JE, Tirado-Strayer N. Assessing students' executive functions in the classroom: Validating a scalable group-based procedure. J. Appl. Dev. Psychol. 2018;55:4-13. doi: 10.1016/j.appdev.2017.03.003.

19. Hoosen N, Davids EL, de Vries PJ, Shung-King M. The Strengths and Difficulties Questionnaire (SDQ) in Africa: a scoping review of its application and validation. Child Adolesc Psychiatry Ment Health. 2018;12. doi: 10.1186/s13034-017-0212-1.
