# Supplementary figures and images for: Growth, physical, and cognitive function in children who are born HIV-free: School-age follow-up of a cluster-randomised trial in rural Zimbabwe
Source: PLoS Med. 2024 Oct 11;21(10):e1004347. doi: 10.1371/journal.pmed.1004347 (PMC11498706; doi:10.1371/journal.pmed.1004347)

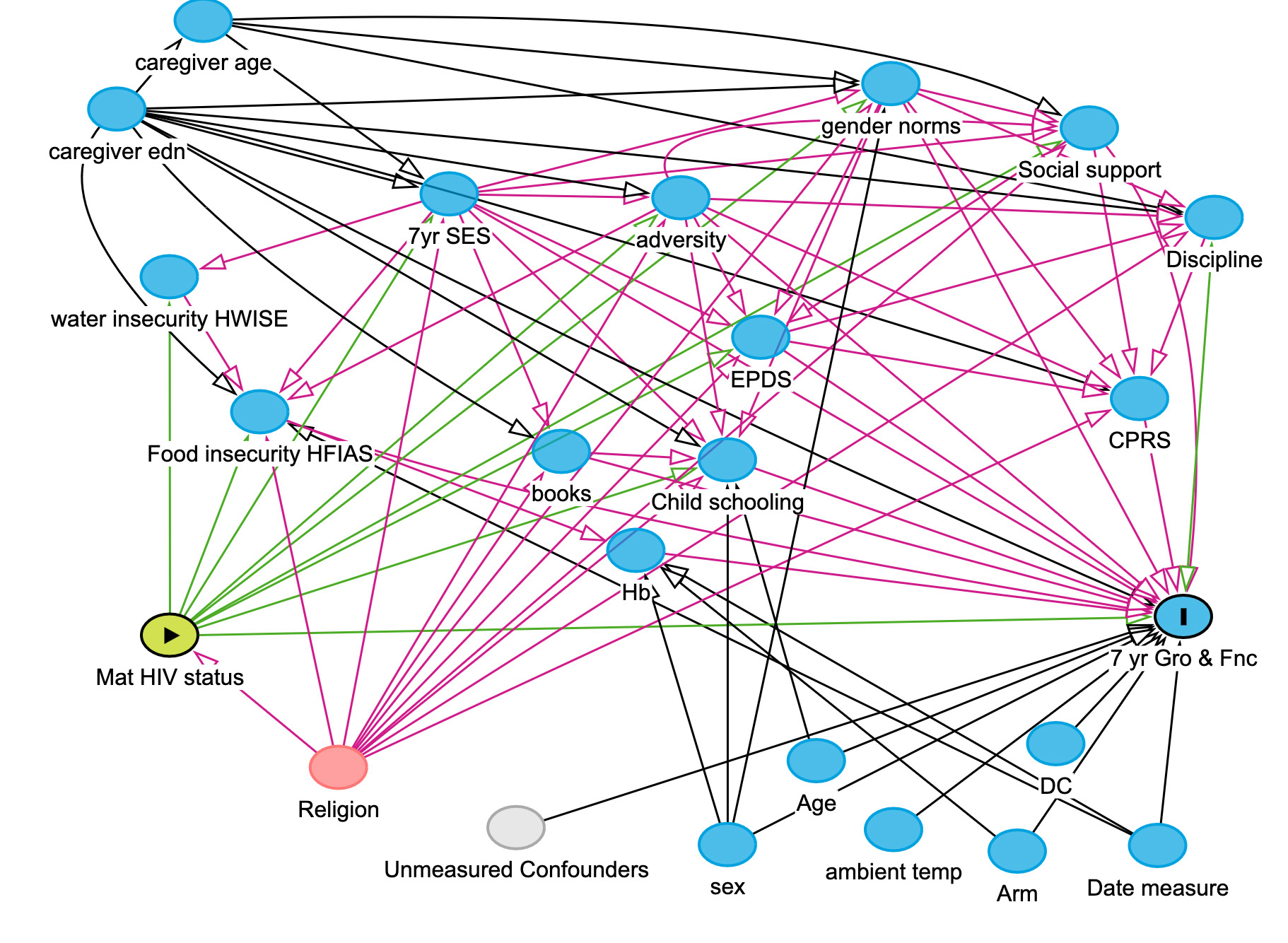

Supplement: S1 Fig — Variables listed from top to bottom: Caregiver edn: Caregiver schooling in number of years, Caregiver age: age of primary caregiver at 7 year visit, 7yr SES: contemporary socioeconomic status (wealth index), adversity: contemporary adversity score, EPDS: contemporary caregiver Edinburgh Postnatal Depression Score, Gender norms: contemporary caregiver gender norm scale, Social support: contemporary caregiver social support scale, Discipline: child discipline scale, Water insecurity (HWISE): Household water insecurity experiences scale, Food insecurity (HFIAS): Household food insecurity experiences scale, Books: number of children’s books at home, Child schooling: Total child schooling in years and months, Hb: child contemporary haemoglobin measured during visit, CPRS: Child parent relationship scale (measure of nurturing), Religion: household religion, mat HIV: Maternal HIV status during pregnancy (the exposure), 7yr Gro & fnc: child growth, cognitive and physical function at 7 years (outcome), DC: Data collector, sex: Child sex, Arm: SHINE trial intervention arm, Date measure: calendar quarter when measurement performed, Age: exact age of child, Ambient temp: Ambient temperature: average temperature during SAHARAN toolbox measurements, Unmeasured Confounders: unmeasured confounders. Adjustment variables for model 2 were arm, Data Collector, age of child, calendar age recruited, temperature, sex, Socioeconomic status, Caregiver depression measure (EPDS), Household food insecurity (HFIAS), Household religion, Caregiver social support, Caregiver gender norms, Caregiver age, Caregiver education, Adversity score, Children’s books at home. Colour coding: Green circle: exposure variable, green arrow: causal path, blue circle with I: outcome variable, blue circles: ancestor of outcome variables, red circle: ancestor of exposure and outcome variables, red arrows: biasing path, black arrows: postulated interactions not on the biasing path, grey circle: unobserved (latent) var [file pmed.1004347.s002.tiff]

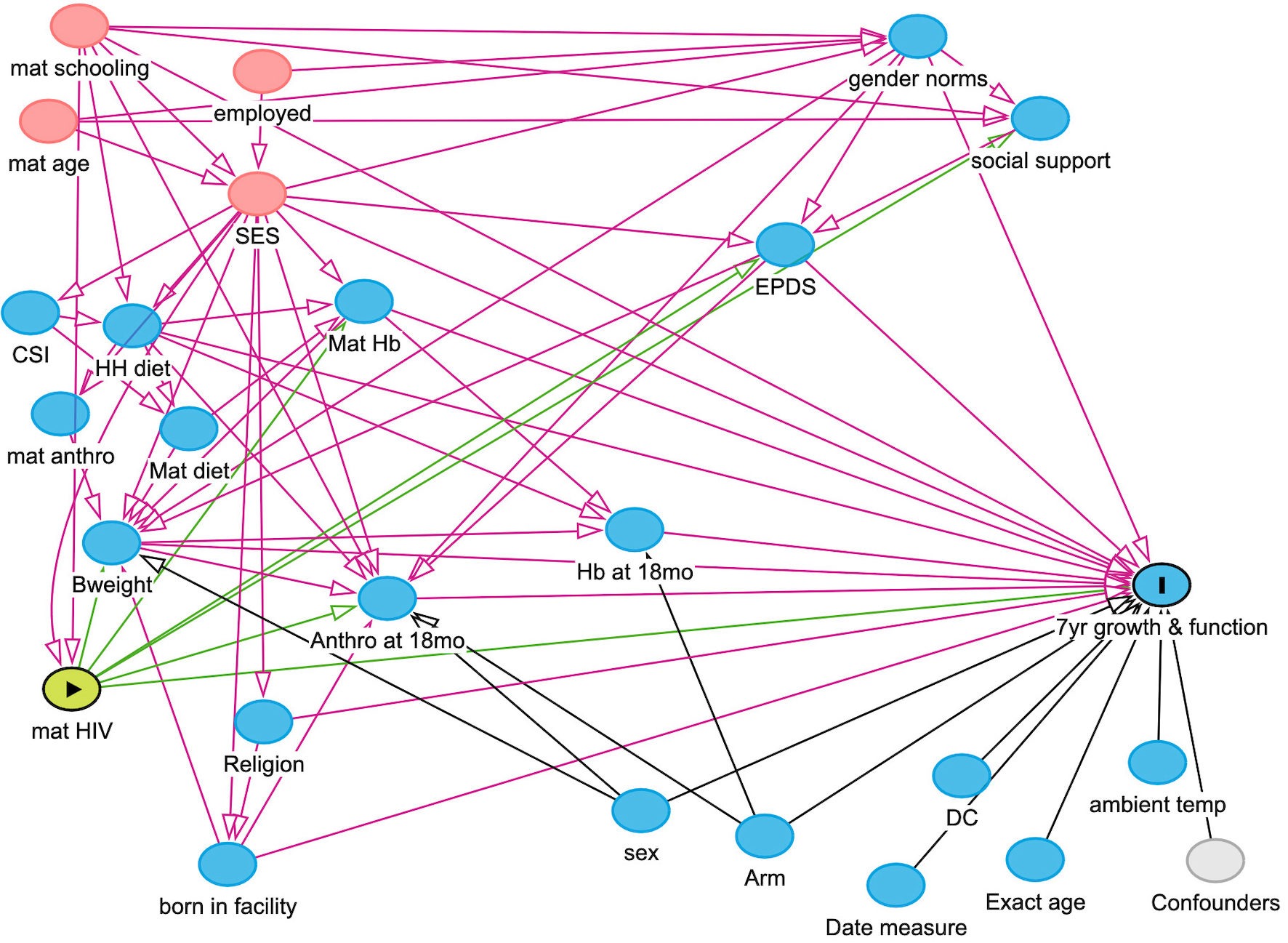

Supplement: S2 Fig — Variables listed from top to bottom: Mat schooling: Maternal schooling in number of years, mat age: maternal age, SES: baseline socioeconomic status (wealth index), employed: whether mother was employed or not, EPDS: Baseline maternal Edinburgh postnatal depression score, Gender norms: baseline maternal gender norm scale, social support: baseline maternal social support scale, CSI: Coping strategies index (measure of food insecurity), HH diet: household dietary score, Mat Hb: Maternal haemoglobin in pregnancy, Mat diet: maternal diet score, Mat anthro: Maternal anthropometry (note height was used in model), Bweight: child birthweight, mat HIV: Maternal HIV status during pregnancy (exposure), 7yr Gro & fnc: child growth, cognitive and physical function at 7 years (outcome), Anthro at 18 months: child anthropometry at 18 months (length-for-age-z-score used in model), religion: household religion, Hb at 18 months: child haemoglobin at 18 months of age. DC: Data collector, sex: Child sex, Arm: SHINE trial intervention arm, Date measure: calendar quarter when measurement performed, Age: exact age of child, Ambient temp: Ambient temperature: average temperature during SAHARAN toolbox measurements, Confounders: unmeasured confounders. Adjustment variables were: Arm, data collector, age of child, calendar age recruited, temperature, Anthropometry at 18mo, Birthweight, maternal depression score (EPDS), household dietary score, maternal haemoglobin in pregnancy, baseline socioeconomic scale, born in facility, gender norms, maternal years of schooling. DC: Data collector, sex: Child sex, Arm: SHINE trial intervention arm, Date measure: calendar quarter when measurement performed, Exact age: exact age of child, ambient temperature: average temperature during SAHARAN toolbox measurements, Confounders: unmeasured confounders, Colour coding: Green circle: exposure variable, green arrow: causal path, blue circle with I: outcome variable, blue circles: ancestor of outcome variables, [file pmed.1004347.s003.tiff]
