# Supplementary material for: Growth, physical, and cognitive function in children who are born HIV-free: School-age follow-up of a cluster-randomised trial in rural Zimbabwe
Source: PLoS Med. 2024 Oct 11;21(10):e1004347. doi: 10.1371/journal.pmed.1004347 (PMC11498706; doi:10.1371/journal.pmed.1004347)
Supplement: S1 SHINE Protocol — (DOCX) [file pmed.1004347.s004.docx]

**SHINE**

**Sanitation Hygiene Infant Nutrition Efficacy Project**

ClinicalTrials.gov identifier NCT01824940

Protocol version 28

October 11th, 2021

__________
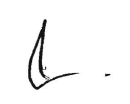
_____________

Professor Andrew Prendergast, PI

*October 11th, 2021*

Date:_____________________

**Summary of changes from version 27**

**Minor changes**

- Change of form version **SHINE_Form_99_v2_7yr VHW_monitoring**
- Additional form for HIV positive mothers, to confirm details from pregnancy and early life using **SHINE_Form_100_v1_7yr_early_life_questionnaire**
- Change from CMV DNA PCR quantification in a subgroup of mothers and infants to CMV DNA PCR quantification in all mothers and infants with stored samples available (plasma, urine, saliva) at any visit date.

**Summary of changes from version 26**

**Minor changes**

- Change of form versions **SHINE_Form_96_v2_7yr _Caregiver_Questionnaire** and **SHINE_Form_97_v2_7yr_Child_Data_Collection**

**Summary of changes from version 25**

**Major changes:**

- Addition of three research questions (page 24; questions 21-23) to evaluate the long-term outcomes of children enrolled in SHINE, by assessing i) the differences in health, growth, physical function and cognition by randomized trial arm; ii) differences in health, growth, physical function and cognition between HIV-exposed uninfected and HIV unexposed children; and iii) which early-life exposures influence school-age health, growth, physical function and cognition.
- A follow-up visit will be conducted to address these research questions in 1300 children at 7 years of age, during which a battery of tests will be performed that directly measure cognitive function, physical function, growth and body composition (all defined in Table 3). We will also measure further aspects of the child’s development, behavior, emotions and schooling, together with updated household sociodemographic data, using a detailed caregiver questionnaire. We will offer HIV testing to the caregiver as part of this visit, and offer HIV testing to the children of HIV-positive and HIV-unknown mothers.
- This study will use **SHINE_Form_94_v1_7yr_Caregiver_Consent, SHINE_Form_95_v1_7yr_Child_Assent, SHINE_Form_96_v1_7yr _Caregiver_Questionnaire** and **SHINE_Form_97_v1_7yr_Child_Data_Collection** (Pages 72-73), **SHINE_Form_98_v1_Community_sensitisation form**
- A shortened battery of tests, derived from the 7-year-old assessment, will be conducted annually between ages 8-12 years to assess the trajectory of growth, cognitive and physical function during mid-childhood.
- All 1300 children in the long-term follow-up study will have monthly data collected by Village Health Workers on acute illnesses using **SHINE_Form_99_v1_VHW_monitoring form** to determine whether there are differences in illness episodes between HIV-exposed and HIV-unexposed children. We have updated event reporting processes during long-term follow-up (page 105-6).
- Change of PI from Prof Jean Humphrey to Prof Andrew Prendergast

**Minor changes**

- In a group of 10 HIV-negative SHINE mothers who had stool samples collected in pregnancy, we are requesting permission to explore the role of microbial biofilm formation at Johns Hopkins University (page 64).

**Summary of changes from version 24**

**Minor changes:**

- Addition of further questions within the pilot study to validate tools to assess growth, physical and cognitive function of 7-year old children. Within the child questionnaire, we are piloting the Foundational Learning Module recently used in the MICS UNICEF study in Zimbabwe. Within the pilot caregiver questionnaires, we are asking additional questions on child schooling affected by COVID-19 precautions, homework, household food and water insecurity and the child discipline module used within the MICS 2019 study. Therefore the pilot study will use the following data collection forms: **SHINE_Form_91_v2_7yr_Pilot_Caregiver_Questionnaire** and **SHINE_Form_92_v2_7yr_Pilot_Child_Data_Collection.** We are also piloting a community sensitization form: **SHINE_Form_93_v1_Community_sensitisation form.**
- We are piloting a further measure of executive function: the NEPSY Animal card-sorting test. We are also piloting the OMNI and Visual Analogue scales of effort during the physical function tests.
- We have made minor adjustments to the consent and assent forms so we will use these versions: **SHINE_Form_89_v2_7yr_Pilot_Caregiver_Consent** and **SHINE_Form_90_v2_7yr_Pilot_Child_Assent**

**Summary of changes from version 23**

**Major changes:**

- Addition of two research questions, to assess the impact of the SHINE interventions on school-aged child growth, cognitive and physical function. The new Secondary Aims (Aims 19, 20) are stated on page 22-23.
- Addition of a pilot study to first validate the tools to assess growth, physical and cognitive function of 7-year old children. This pilot study will enroll up to 80 children who live in rural Shurugwi but were not recruited to SHINE, to test a battery of tests that examine cognitive function, physical function, growth and body composition (Table 3). We will also pilot caregiver questionnaires on the child’s development, behavior, emotions and schooling; an adversity index; and questionnaires on food security, dietary diversity and socioeconomic status. This pilot study will use **SHINE_Form_89_v1_7yr_Pilot_Caregiver_Consent, SHINE_Form_90_v1_7yr_Pilot_Child_Assent, SHINE_Form_91_v1_7yr _Pilot_Caregiver_Questionnaire** and **SHINE_Form_92_v1_7yr_Pilot_Child_Data_Collection** (Pages 72-73).

**Minor changes:**

- Addition of cytomegalovirus (CMV) DNA PCR on 150 HIV-negative mothers at baseline (page 77).
- Addition of Jonathan Broad as a student investigator (Page 100).

**Summary of changes from version 22**

**Major changes:**

- Addition of formative research about improving the IYCF intervention using locally available foods in 24 mothers with children under 18 months who live in areas that had been randomized to IYCF during SHINE that the Village Health Worker is experienced in IYCF issues to evaluate the feasibility and acceptability of adding dehydrated egg, moringa leaf powder and sugar bean powder to infant complementary feeding interventions (Page 63-64).This pilot study will use **SHINE Form 86 Recipe TIPS Trial** and **SHINE Form 87 Recipe Trial Focus Group** for written informed consent, and **SHINE Form 88 Recipe Trial Focus Group Discussion Guide** as an interview guide.

**Minor Changes:**

- Addition of urinary polycyclic aromatic carbons measured by GC-MS in maternal and infant urines to test a biomarker approach for estimating indoor air pollution. We will send urine samples to Dr. Laura Smith at University at Buffalo to undergo measurement (pages 40, 56, 68)
- Measurement of HIV and CMV viral loads on all HIV-positive mothers at baseline and 32 weeks (rather than just a sub-group at baseline), pages 68-69
- Addition of CMV PCR on stored saliva in all HIV-exposed children (1 month through 18 months of age), page 69
- Addition of IL-6 and lipopolysaccharide binding protein (LBP) by plasma ELISA in a sub-group of 300 mothers and children at baseline and 1 month of age, respectively; page 68
- In a subgroup of 200 SHINE mother infant pairs, microbial DNA from stool specimens will be sequenced at a deeper level in the laboratory of Dr Nicola Segata in Trento, Italy (page 69)
- Addition of Nadia Koyratty as a student investigator (pages 89-90) from University at Buffalo and confirmation that University at Buffalo has entered into an authorization agreement with the JHBSPH IRB (page 90).
- Dr. James Church, Miss Jaya Chandna (student investigators) and Mr Goldberg Mangwadu as Co- Principal Investigator’s work has been completed therefore we will not be renewing their research registration certificates but however we will keep their names on SHINE's write-ups and manuscripts (pages 89-91).

**Summary of changes from version 21**

**Minor Changes:**

- We have rephrased the In-depth Interview Questions SHINE_Form 82_v2_Friendship Bench Maternal Indepth Interview Guide and the Focus Group Discussion Guides, SHINE_Form 83_v2_Friendship Bench Maternal Focus Group Discussion Guide and SHINE_Form 85_v2_Friendship Bench VHW Focus Group Discussion Guide for purposes of clarity to participants being interviewed. The version changes on the documents have been effected in the highlighted protocol_v21 dated September 18, 2018 from page 69-70.

**Summary of changes from version 20**

**Major changes:**

- Addition of a pilot intervention for maternal depression in 30 SHINE mothers to evaluate the feasibility of delivering the Friendship Bench through Village Health Workers. Depression is associated with child stunting and finding ways to tackle depression is therefore an important goal of SHINE.
- We have added a community brochure (**Friendship Bench Community Sensitisation Brochure v1**) for distribution in the study area before recruitment.
- We have designed an **Introductory Script** to explain to the mother our reason for visiting.
- We will gain written informed consent using **SHINE_ Form 80_v1 Friendship Bench Consent Form.**
- A Data Collector will undertake a home visit to collect data using **SHINE_ Form 81_v1 Friendship Bench Data Collection Form**.
- At the end of the intervention, a Data Collector will visit the mother at home and repeat the assessment using **SHINE_ Form 81_v1 Friendship Bench Data Collection Form**.
- In addition, we will conduct 10 in-depth interviews with women, using **SHINE_ Form 82_v1 Friendship Bench Maternal In-Depth Interview Guide**, to evaluate their understanding of depression and the acceptability and feasibility of the Friendship Bench in a rural setting. We will also conduct 2 focus group discussions with women (each comprising 6-8 participants) using **SHINE_ Form 83_v1 Friendship Bench Maternal Focus Group Discussion Guide**. The interview will be audio recorded if the mother provides permission.
- A subgroup of 6-8 Village Health Workers will be asked to provide written informed consent to take part in a focus group discussion using **SHINE_ Form 84_v1 Friendship Bench VHW Focus Group Discussion Consent Form.** The interview will explore their experience of the Friendship Bench and will be conducted using the **SHINE_ Form 85_v1 Friendship Bench VHW Focus Group Discussion Guide**.

**Minor Changes:**

- Update of SGA/preterm substudy to include low birth weight (<2.5kg) and early stunting (LAZ<-2 at 1 month of age) as additional case definitions (pages 23 and 28)
- Update of methodology for microbiota analysis to address mechanistic questions using metagenomics, metabolomics, culture and animal model investigations (pages 57, 67and 68)
- Addition of monocyte immunophenotyping and lymphocyte proliferation to flow cytometry panels undertaken in mothers and infants (pages 56,66)
- Addition of multiplex cytokines, sCD163, HIV and CMV viral load measurement in 150 HIV-exposed and 150 HIV-unexposed mother-infant pairs to evaluate differences in immune activation between groups (page 66)
- Addition of Dr Christie Noble as a student investigator

**Summary of changes from version 19**

**Minor changes:**

- Addition of Dr Ceri Evans as a student investigator
- Change in role for Jaya Chandna, who is now a student investigator

**Summary of Changes from version 18**

**Major changes:**

- Addition of urinary and faecal metabolomics in a subgroup of infants and their mothers. In a group of 1000 infants (500 with ‘good growth’, defined as LAZ>-0.5 at 18 months; and 500 with ‘poor growth’, defined as LAZ<-2 at 18 months) we will send urine samples from the 12 month visit to Dr Jon Swann at Imperial College London to undertake untargeted metabolomics by nuclear magnetic resonance spectroscopy and/or mass spectrometry. From a group of 150 mother-infant pairs (longitudinal samples from 32 weeks and 1 month postpartum for mothers, and 1, 3, 6, 12 and 18 months for infants) we will send stool samples to the University of British Columbia, Vancouver, to undertake untargeted metabolomics by nuclear magnetic resonance spectroscopy. Metabolomics is a powerful technology for assessing metabolic pathways without a priori hypotheses, which will provide unique insights into the pathogenesis of stunting beyond the EED pathway that we are already testing, but methodology is not available in Zimbabwe for this work.
- Addition of a qualitative study of mothers' experiences using the SHINE
  interventions in which we will purposively sample up to 80 mothers (20/arm) to conduct an in-depth interview. Consent will be obtained using **SHINE_ Form 76_­ v1_ Mother’s Experience with Interventions Consent Form** and the in-depth interview using **SHINE_ Form 77_v1_ Mother’s Experience with SHINE interventions Questionnaire**.
- Addition of observation study to be conducted once on 180 mother-infant pairs. These households will be selected by matching the gender and age of the youngest 90 infants in the WASH arms to 90 infants in the non-WASH arms. The purpose of these visits is to check compliance with WASH and IYCF behaviors, and therefore provide an additional measure of intervention uptake. In addition, a trained observer will conduct a 6-hour structured observation. The researcher will visit the mother and obtain consent using **SHINE Form 78**. After obtaining consent the researcher will observe the household as well as the normal daily routines of the mother using **SHINE form 79** to record specific baby activities and household observations.
- Removal of unscheduled visits. We have removed microbiology testing and **SHINE_ Form 42_v1_Unscheduled Visit Form**. .
- Request to change recipient of aflatoxin-lysine analyte of extracted serum samples for measurement of aflatoxin-lysine by mass spectrometry from John Hopkins University USA to University of Maryland College Park,USA.

**Minor Changes:**

- Correction to research questions 13, 14 and 15 in the Secondary Specific Aims; these questions will explore relationships between pregnancy exposures and mean birth length, birth weight and gestational age
- Update of the preterm birth substudy, which now includes ‘small for gestational age’ as an outcome together with preterm birth. This enables us to explore the overlap between SGA and preterm in infants.
- We have updated the methodology regarding the adverse birth outcome substudies to clarify the approach that will be taken for each, particularly regarding selection of cases and controls.
- Addition of infant scores to the OMCI test on **SHINE_Form 63_v5_ 24 Month Visit Data Collection Form**
- Clarification that 24 month visits will be conducted even if the infant has moved from the primary SHINE home
- **SHINE_Form 71_v3_Body Composition Data Collection Form** has been updated. Mid-thigh circumference has been changed to mid-calf circumference.
- We have updated the current panel of biomarkers being used to assess maternal and infant EED throughout the proposal, and added FUT2 measurement in infant saliva
- We have changed the location for sample export to analyze plasma for measurement of the tryptophan metabolism pathway and citrulline, from Oregon Analytics, USA to Imperial College London, UK.
- We clarified sample sizes for mycotoxin measurement. In summary, afm1 will be assessed on all enrolled women at baseline and 32-34 weeks gestation and 1600 EED infants at 3, 6, 12 and 18 months. Aflatoxin-lysine and multiple urinary mycotoxins will be assessed in women enrolled in the miscarriage, stillbirth and preterm/SGA substudies. and 1600 EED infants at 6, 12 and 18 months. Clarifications appear on pages 37, 51, 52 and 61.
- We have updated **SHINE_ Form 55_v2__Birth Facility Information** to include parity.
- Addition of Dadirai Fundira as a student investigator
- We have also removed Dr Mduduzi Mbuya (**PhD**) who also left the organization in September 2016 from Supervision roles and responsibilities and added him as an External Investigator, as he continues to contribute towards SHINE study page 84.

**Summary of Changes from version 17**

**Minor Changes:**

- Guardian consent will be obtained where a biological mother is not able to consent at the 24 month visit using **SHINE_Form 72_v1_Guardian Neurodevelopment at 24 months Visit Consent.**
- Addition of **SHINE_Form_73_v1_Specimen Storage and Shipment at 24 months** **Visit Consent** to obtain consent to store and ship samples collected at the 24 month visit, or **SHINE_Form_74_v1_Guardian Specimen Storage and Shipment at 24 months Visit Consent** when obtaining consent from a guardian.
- Clarification that body composition at 24 months will be assessed in the subgroup of children turning 24 months of age from September 2016. Addition of mid-thigh circumference measurement to this visit.
- Addition of **SHINE_Form 71_v2_Body Composition Data Collection Form** to collect data on body composition at 24 months.
- Addition of collagen X as a biomarker of bone growth, measured by ELISA in infants recruited to the EED substudy (page 61).
- AFM1 will now be measured on all enrolled women at baseline and 32 weeks, instead of the original subgroup of 3200 women (page 36), to provide more complete data on short-term aflatoxin exposure during pregnancy.
- Addition of a new information sheet, SHINE_Form 75_v1_Diarrhea Sample Collection Information Sheet to improve specimen recovery rates in the diarrhea substudy.
- Request for shipment of samples to perform rotavirus IgA assays at the University of Vermont, USA, because methodology cannot be established in country.

**Summary of Changes from version 16**

**Major Changes:**

- Addition of bioimpedance analysis, skinfold thicknesses and leg length measurement to the 24 month visit, to more fully assess the impact of the randomized interventions on infant body composition, with consent obtained using **SHINE _Form 70_v1 Neurodevelopment and Body Composition at 24 months visit Consent Form.** (Table 9, and page 57). Details of the devices are listed in section 11b.

**Minor Changes:**

- Sample size for EED substudy updated on page 24 to explain the detectable difference in biomarkers between groups.
- We have updated the list of assays to be conducted on maternal and infant biospecimens to reflect current panels of markers that best define EED and oral vaccine responses. We have therefore removed AGP, IL-6, IL-1b and IGFBP3 from the assay list, and added kynurenine:tryptophan ratio (plus metabolites along tryptophan/IDO1 pathway, AhR agonists and serotonin) and tetanus IgG. Additionally, to gain insights into functional immune responses, we have added whole blood stimulation using pathogen-associated molecular patterns at the 18 and 24 month visits (Table 9).
- In line with the changes in biomarkers above, we are requesting permission to ship plasma samples for all mothers and infants requiring citrulline, kynurenine:tryptophan ratio (plus metabolites along tryptophan/IDO1 pathway, AhR agonists and serotonin) and essential amino acids to Oregon Analytics in USA, where they will be measured by mass spectrometry.
- We have added analysis of enteropathogens by Taqman Array Card on stool samples of infants in the EED substudy. These will be analyzed in conjunction with diarrhoeal specimens using the same technique (at University of Virginia, USA), which will allow us to calculate the attributable fraction of diarrhea caused by each pathogen, accounting for carriage of enteropathogens in EED ‘control’ specimens.
- Addition of FUT2 as a salivary analyte in women to evaluate maternal capacity to fucosylate human milk oligosaccharides, which is linked to infant growth.
- Clarification in Table 9 that HIV DNA PCR will be conducted on all infants whose mothers are known to be HIV-positive at the 18 and 24 month visit, as stated in the consent form, with results returned to the caregiver.
- We have reduced salivary collection at the 24 month visit from 3 infant saliva samples to one single sample (Table 9).
- We have simplified the assessment of early child development by removing the assessment of behavior and mood using the frustration task (Table 3).
- Addition of **SHINE_FORM 67_ v1_ Parent Information sheet** listed in the protocol as the information sheet provided to caregivers of children with developmental delay.
- Addition of responsibilities to Research Administration and Compliance Manager page 82
- Addition of Dr Joseph Piper as a student investigator page 82

**Summary of changes from version 15**

**Major Changes:**

- Recruitment figures have been updated to 5282 women total, to reflect the 10% over-recruitment that was conducted to ensure sufficient power to conduct sensitivity analyses. This was particularly important in the WASH arms because of the failure of the original subcontractor of latrine construction (Oxfam UK) to ensure timely installation of latrines per protocol for the first 730 enrollees.

**Minor Changes:**

- **Addition of urinary aflatoxin M1 as a measure of aflatoxin exposure in up to 1000 infants at 12 and 18 months of age.**
- End of allowable window for 24 month visit extended from 110 weeks to 112 weeks
- Update of method used to assess infant behavior and mood following piloting of tool (Table 3)
- Updated data collection form **SHINE_Form 63 v2_24 month visit data collection form** to reflect final tests to be administered following piloting.
- Addition of a third infant saliva sample at the 24 month visit in order to monitor stress recovery in children, and removal of maternal saliva sample from the 24 month visit (Table 9). This is reflected in amended consent form **SHINE_Form 65 _v2 Neurodevelopment at 24 months Consent Form.**
- Submission of a new information sheet (**SHINE_Form 67_v1 Parent Information Sheet 24 Month Visit)** that can be given to caregivers of children following Early Child Development assessments.
- Removal of the intensive 24 hour dietary recall for infants in the EED substudy at the 18 month visit (Table 10)
- Updated data collection form **SHINE Form 43v2 24Hr Dietary Recall Form** to address two minor issues observed in 3 questions: a lack of clarity observed in initial interviews and separation of a two part question (Page 58).
- Addition of a two-weekly reminder phone call to mothers of infants in the diarrhea substudy (page 58). This is to improve collection of specimens for infants who develop diarrhea.
- Change to specimen collection schedule at 18 months if mother-infant pairs are visited at long distance, out of the study districts. Where it is not possible to get specimens processed in the hub laboratory the same day, infants will instead have a dried blood spot card stored, and mothers will not have a blood sample collected (Tables 9 and 10).
- Provision of an information sheet (**SHINE_Form 68_v1_ Specimen and Testing non EED Information Sheet)** for mothers of non-EED babies at the 18 month postnatal visit, and an information sheet (**SHINE_Form 69_v1_Specimen and Testing EED Information Sheet**) for mothers of EED babies to explain the reasons for specimen collection and haemoglobin testing.
- Local anaesthetic cream will be available for infants to reduce the pain associated with venipuncture if the mother wishes (Tables 9, 10).
- Addition of Jaya Chandna as a collaborator (Page 79).

**Summary of changes from version 14**

Minor Changes:

- Addition of 70 non SHINE mothers to step 2 of piloting the McArthur Bates language assessment test
- Reduction from 70 to 20 SHINE mothers to step 3 (piloting ALL cognitive tests)

**Summary of changes from version 13**

**Major Changes**

- Addition of three research questions, to assess the impact of the SHINE interventions on early child development. This requires a new visit at 24 mo of age, with additional informed consent taken for this additional visit, which will enable us to more fully evaluate the impact of the randomized interventions on health and growth, by assessing domains of child development that are not captured in current outcome measures. This additional visit allows us to evaluate more completely whether SHINE interventions helped children to ‘thrive’ and harness their full human potential, beyond the measures of illness and linear growth that were already addressed. The new Secondary Aims are stated on page 16 (Aims 16, 17, 18), and the description of the new visit is outlined on page16.
- We are requesting permission to ship samples for all mothers and infants requiring lactulose-mannitol measurement in urine samples to Oregon Analytics in USA.
- Pages 73-77: on advice of the international IRB (Johns Hopkins) and local IRB (MRCZ), we have altered the reporting process for Adverse Events (AE) and Serious Adverse Events (SAE). We will only report AE and SAE within the expedited time-frame if they are judged to be uncertainly, probably or definitely related to the study interventions or procedures. Events that are unlikely related to study interventions or procedures will be reported annually in tables, split by AE/SAE type.

**Minor Changes**

- Addition of measurement of alpha-1-antitrypsin and neopterin in maternal serum. Protocol already included measurement in stool. This change was made to fully characterize these biomarkers in women which represent inflammation and gut damage. (pg 50 and Table 3)
- Revision of the section “Developmental Evaluation of the protective play space intervention component” to include participants of the non-WASH (SOC and IYCF) arms of the study. This change was made in order to understand the positive and intentional impact of the WASH intervention as well as any unintended consequences that might result; in addition to the original intent of strengthening the playspace component of the intervention. Accordingly, the exploration has been renamed in the protocol to “Qualitative study to assess the potential effectiveness of the SHINE WASH intervention” on page 45-6.
- Continue recruitment to the Environmental Enteric Dysfunction substudy beyond the original sample size of 1000 HIV-negative mothers and their infants. We plan to enroll all mothers who joined SHINE from 1^st^ May 2014 to the end of recruitment. This will ensure at least 150 children per trial arm have longitudinal assessments of EED (allowing for missed samples and loss to follow-up) to allow the substudy to be adequately powered.
- Secondary Aim 8 has been amended to remove “between birth and 6 months of age”
- Table 7 footnote - If women request an HIV test at other study visits to learn their status, we will undertake a rapid test in the home where feasible.
- Table 9 footnote - Anthropometry data and hemoglobin result at 18 months (primary trial endpoints) will be checked carefully by Research Nurse Supervisors at the SHINE hub; in cases where primary endpoints are either missing or outside the stipulated plausibility ranges, a home visit will be conducted to repeat the measurement(s).
- Table 11 footnote – if an infant HIV DNA PCR is indeterminate we will conduct a home visit to give the result to the caregiver, but will request a repeat sample so that we can repeat the test and return the result to the mother to clarify the infant’s HIV status.
- Page 59: If maternal HIV status was not confirmed using samples collected at baseline and/or 32 weeks, HIV testing will be repeated in EED substudy mothers using the maternal sample collected at 1mo postnatal, to ensure final maternal HIV status has been determined, as stated in the main trial consent form.
- Clarification of consent procedure for illiterate women (section 7c).
- Page 78: Updated role of Assistant Director, Field Operations.

**Summary of changes from version 12**

**Major Changes**

- Addition of the measurement of T-2 toxin, zearalenone and ochratoxin A in mothers in the preterm birth substudy and 200 infants.
- Request to ship urine from mothers and infants to University of Muenster, Germany for measurement of fumonisin, deoxynivalenol, T-2 toxin, zearalenone and ochratoxin A (page 32).
- Addition of an exclusion criterion to the EED substudy: infants with a major non-fatal

abnormality that is likely to directly affect gut health/function or stature (e.g. neural

tube defects, cerebral palsy, Down syndrome). This is because additional

procedures may cause undue discomfort or pose additional risk to these infants, and

will yield data that are difficult to interpret in view of their underlying medical

condition.

**Minor Changes**

- Addition of measurement of multiple mycotoxins at 6 months and 18 months of age in 200 infants and removal of measurement at 3 months.
- Change of measurement of multiple mycotoxins in the mothers enrolled in the preterm birth substudy instead of 200 mothers in the EED substudy.
- Change to request to ship aflatoxin-lysine analyte to Johns Hopkins University, USA and/or University of Muenster, Germany
- Amendment to one secondary aim (number 14), to include the question of whether adverse effects of maternal schistosomiasis are mediated through inflammation.
- Addition of a laboratory assay to measure poliovirus IgA in infants enrolled to the EED substudy as a measure of oral vaccine immunogenicity. This will allow us to address the question of whether a WASH intervention improves immune responses to polio vaccine as well as rotavirus vaccine (EED substudy aims 13 and 14 updated)
- Extend outer window of 18 month visit to 130 weeks

**Summary of changes from version 11**

**Major Changes**

- Request to ship aflatoxin-lysine analyte from extracted maternal and infant plasma samples to Johns Hopkins University, USA, for measurement of aflatoxin-lysine by mass spectrometry.
- Addition of one research question to the EED substudy to assess the relationship between EED and aflatoxin exposure using urine and plasma samples.

**Minor Changes**

- Measurement of urinary aflatoxin M1 in 3200 women instead of 2000 women as an additional measure of aflatoxin exposure that will inform seasonality of exposure in the SHINE districts (page 30).
- We have added a new community brochure (Community Brochure Post Recruitment V1) for distribution in the study area after recruitment had ended.
- We have removed stool collection from main trial infants at 18 months of age (Table 9), in line with our decision not to test for soil-transmitted helminthes at 18 months of age. Infants enrolled to the EED substudy will continue to have stool collected at 18 months of age (Table 10).
- We have delayed enrollment into the diarrhea sample collection substudy until 1^st^ June 2015, to provide more time to prepare for this substudy in the field. We have also changed the timing of consent from the 1 month postnatal visit to the 3 month postnatal visit, which is a less busy visit and provides more time to undertake the consenting process.
- We have clarified the HIV testing procedure for women and infants in the main trial at 18 months postnatal (Table 9 footnotes): if the mother is found to be HIV-positive, she will have a CD4 count measured and the infant blood will be used to test for HIV by DNA PCR and to measure CD4 count; results will be returned to mothers by home visit. For HIV-exposed infants in the EED substudy, results of the HIV DNA PCR will be returned to the mother by home visit (Table 10, footnote).

**Summary of changes from version 10**

Minor Change

- On page 52 we have amended Diarrhoea Sub-study Visit form to version 3 (SHINE_Form 51v3 Diarrhoea Sub-study Visit). We have updated this form to allow for more than one episode of diarrhoea specimen collection, altered the coding of diarrhoeal specimen labelling and separated out presentation with blood and mucus in the stool.
- On Page 53 we have corrected the word Welfare to Care in line with the new name of the Ministry which changed from Ministry of Health and Child Welfare to Ministry of Health and Child Care.

**Summary of changes from version 9**

**Major changes**

- We have removed testing of infant stool for soil-transmitted helminths (STH) at 18 months of age. This is because the 2010 National Helminthiasis Survey showed very low prevalence of STH in Shurugwi and Chirumanzu districts (1.5% and 0% among school-age children, respectively), and we would expect the prevalence to be even lower in 18 month old children. Because the laboratory methods for helminth detection are not highly sensitive, we have decided not to test stool samples routinely at 18 months. Stools are being stored at -80C and could later be used to diagnose STH by PCR if this was necessary for later analyses. We have therefore removed details of the Kato Katz test from the Diagnostic Tests section. We have added an explanation of how we will inform the study participants: that we will not test their children for helminthes because a large survey conducted by the Ministry of Health has demonstrated this is not a problem among children in Chirumanzu and Shurugwi.

**Minor changes**

- We have changed the sample type collected at 18 months for infants in the main trial, from one EDTA and one serum gel clot tube, to two EDTA tubes. This is to simplify procedures in the field and the laboratory, because all analytes can be measured on plasma. The volume of blood collected has not changed, only the blood tube.
- We have updated enrolment procedures for the EED substudy so that women who miss the 32 week gestation visit can provide consent to join the EED substudy at the earliest opportunity thereafter.
- We have clarified in Tables 9 and 10 that infants enrolled in the EED substudy should have specimens collected according to the EED substudy schedule (Table 10) and not the main trial specimen collection schedule (Table 9).
- We have clarified that diarrhoeal specimens will only be collected from infants who are enrolled to the EED substudy from March 2015 onwards, and that we will ask mothers to inform us if their infant has blood or mucus in the stool, in addition to watery diarrhoea. Data will be collected at diarrhoeal specimen collection visits using **SHINE_Form 51v2 Diarrhoea Sub-Study Visit.**
- We have updated Tables 7, 9 and 10 to state in the Footnote that missing biological specimens will be collected as soon as possible after a missed visit or failed collection attempt, and that this may occur outside the window of the original visit. This is to minimize missing data in the trial.
- We have updated the turnaround time of HIV testing for infants. The result will be provided to the mother within 4 weeks of collecting the specimen, because of the logistics of transporting dried blood spot specimens from the field to Harare, undertaking HIV DNA PCR testing and returning the result to the mother.
- We are requesting permission to provide women enrolled into the SOC and IYCF arms a certificate at the 18 month visit which pledges that she will have a latrine constructed at the end of the trial. This is to avoid any doubt on the part of the mother that a latrine will be constructed by SHINE.
- We have added an additional section on Adverse Event reporting to the Johns Hopkins University IRB in line with their reporting guidelines.

**Summary of changes from version 8**

**Major changes**

- Addition of two research questions to the EED substudy to assess the relationship between EED and rotavirus vaccine immunogenicity (aim 13), and the impact of WASH interventions on rotavirus immunogenicity (aim 14), using plasma samples already being collected. This aim has been added to leverage the roll-out of rotavirus vaccination within the EPI programme in Zimbabwe. Immunogenicity is defined in Table 3 and added to assays to be undertaken on infant plasma (page 48).
- Addition of microbiological sampling to the unscheduled visit for 800 women (page 45) to assess the impact of WASH interventions on environmental contamination.
- Addition of diarrhoeal stool specimen collection for infants enrolled in the EED substudy (Table 10, and page 48), to assess the pathogens causing diarrhoea in this trial by TaqMan array card PCR, with consent obtained using SHINE_Form 50v1 Diarrhoea Sample Collection Consent Form and data collected using SHINE_Form 51v1Diarrhoea Data Collection Form.
- Request to analyze 50 maternal plasma samples for aflatoxin-lysine by mass spectrometry at Johns Hopkins University, USA.
- Addition of a Qualitative Study of Intervention Nurse evaluation of VHW performance Consent form using SHINE Form_52v1 and SHINE Form_53v1, Intervention Nurse’s perception of VHW performance Interview Guide.
- Addition of SHINE_Forms 57, 58, 59, 60_v1_Reconsent Form for participants who have withdrawn but wish to resume their participation in the study.

**Minor changes**

- Update of Secondary specific aim no 9, to remove anemia at 30-34 weeks gestation (since hemoglobin is not being assessed at this timepoint) but to add mycotoxin status.
- Addition of SHINE_ Form 54v1_End of Pregnancy Catch up Form to Table 5.
- Addition of SHINE_Form 55 Birth Facility Information to collect data on newborn babies from health facility registers (this was previously included in the End of Pregnancy form, but has been taken out to create a new, updated form).
- Addition of SHINE_ Form 56v1_Baseline Catch up Form to Table 5.
- Change of consent procedure for unscheduled observations; we have eliminated SHINE Form _41v1_ Unscheduled Consent Form; instead, consent for unscheduled observations and microbiological sampling will be obtained at trial enrolment, or using SHINE_Form 46-49v1_Consent Form Addendum.
- Update of exit procedures from SHINE trial, using updated SHINE_ Form 24_v3_ Exit form
- Updating of HIV testing procedures for women in Table 7. In addition to the rapid test conducted in the woman’s home, the same sample will be tested for HIV using a rapid test algorithm in the laboratory, to confirm the result obtained in the home.
- Correction of Table 9 to include paternal height, since this measurement will be taken once at any point during the trial, including during the postnatal period.
- Change to the selection criteria for EED substudy; instead of selecting a subgroup of maternal-infant dyads to join the study, all mothers will be invited to join at the 32 week gestation visit until 1000 HIV-negative mother-infant dyads and all HIV-positive mother-infant dyads have been recruited.
- Addition of HIV DNA PCR testing for infants born to HIV-positive mothers at 1mo of age to enable timely diagnosis of HIV infection; CD4 counts will be measured in all HIV-exposed infants. Dried blood spot cards will be made in the laboratory, and not in the field.
- Addition to pre-LM test single urine sample collection from infants in EED substudy (Table 10), as per SOP, prior to ingestion of lactulose-mannitol solution.
- Addition of aflatoxin M1 (AFM1) and creatinine measurement to urine samples from the first 2000 women in the trial, as an additional measure of aflatoxin exposure that will inform seasonality of exposure in the SHINE districts (Table 3, and page 28).
- Update of Loss to Follow Up section to state that intervention delivery will resume if women return to their primary home after moving away.
- Updates to Supervision Roles and Responsibilities (section 15).

**Summary of Changes from version 7**

**Major Changes**

- Change in inclusion/exclusion criteria, as below:

*Inclusion:* Pregnant women residing in randomized clusters within the study districts, whose pregnancy is confirmed by a urine pregnancy test.

*Exclusion*:

- Women residing in the study districts who become pregnant during the enrollment period but do not consent to join the trial
- Women who reside in urban areas of these two districts
- Women who are resident as employees at a homestead or who are living on rented premises

**Summary of changes from version 6**

**Major changes**

- Addition of new substudy, ‘Risk Factors for Neonatal Death Substudy’
- Change of sample collection protocol in Environmental Enteric Dysfunction substudy, substituting PAXGene RNA Blood Tubes for serum tubes. Tubes will be frozen at -80C for subsequent analysis of gene expression profiles in stunted infants.
- Addition of DNA collection from all mothers and infants, on advice of the DSMB, for future studies of genetic determinants of infant growth and health. The Specimen Storage and Shipment Consent Form 9v4) has been updated to request consent to extract DNA from existing samples from women. Addition of cell pellet storage from all infants at 18 month blood draw to enable DNA extraction if consent is provided. The section on the biospecimen repository (section 17) has been updated to provide detail about DNA and RNA storage.
- Addition of consent form addenda (SHINE_Form 46-49v1_Consent Form Addendum, April 1, 2014) for already enrolled participants that covers the addition of, and informed consent for, DNA collection from mother and baby (above), a request for birth notification, clarification on the implications of moving on study participation, and provision of a small incentive gift to the baby in line with cultural norms (Makorokoto).

**Minor Changes**

- Change of terminology from Environmental Enteropathy (EE) to Environmental Enteric Dysfunction (EED)
- Change from ‘maternal HIV status’ to ‘maternal/infant HIV status’ in all analyses of secondary specific aims
- Addition of a small non-monetary gift for all postnatal visits
- Addition of a solar charger for women enrolling into the EED substudy so that they can charge the cellphone provided to improve communication regarding the morbidity diary.
- Change to windows for each study visit; extending the allowable range of data collection to the start of the next scheduled visit to minimize missed visits, and correcting the weeks of intervention delivery (computed using a conversion factor of 4.33) in line with the calendar months of child’s age (Table 5).
- Addition of a qualitative exploration of mothers' experiences in receiving the SHINE
  interventions.
- Addition of structured observations and interviews designed to strengthen the delivery and uptake of the “Protection of a Developing Child” module (W3) among households in the two WASH intervention arms; with separate consent form (SHINE Form­­ 36v1 developmental evaluation consent form) and data collection forms (SHINE Form 37 v1 play space developmental evaluation Visit 1x) and (SHINE form 38 v1 play space developmental evaluation Visit 2).
- Addition of paternal height to data collection activities (Table 7) with separate consent form (SHINE Form 40_v1 Parental Height Consent form).
- Addition of aflatoxin M1 measurement in urine collected from 1500 mothers at baseline visit.
- Addition of unscheduled data collection visits for 800 women on one occasion, to provide an additional measure of the uptake of interventions. A separate consent form will be used for these unscheduled visits (SHINE Form _41v1_ Unscheduled Consent Form) and data collected using SHINE Form 42v1_Unscheduled Visit Form
- Extension of enrolment period for HIV-exposed infants to the EED Substudy to include those who have been born prior to launch of the EED substudy to ensure HIV status can be ascertained on these infants.
- Addition of intensive 24 hr dietary recall (SHINE Form 43v 1 Dietary Recall Form) for 50 randomly selected infants per arm in the EED substudy (Table 10)
- Additional details provided about the infant morbidity diary for infants in the EED substudy. Details provided of a pilot evaluation for the first 40 infants in the EED substudy to determine the best means of support for mothers in diary completion, comparing phone calls, SMS and home visits.
- Addition of two new forms to collect Verbal Autopsy data for mothers or infants who die beyond the End of Pregnancy visit (SHINE Form 35 v1 Maternal Verbal Autopsy and SHINE Form 45v1 Infant Verbal Autopsy, respectively).
- Clarification regarding interventions provided to women who move from their original homes (Loss to Follow Up section) and change from making only 2 attempts at each research visit to enabling more visits to be made, in line with the extended windows for each visit.
- Addition of plasma citrulline as an additional biomarker of EED (Table 3).
- Change of SHINE Community Brochure v4 on contact numbers and inclusion of LMS given to mothers.
- Use of a new **SHINE EED Community Brochure v1** to sensitize study participants and the community about the EED substudy.
- Addition of a definition of ‘guardian’ in situations where the mother dies.
- Addition of more detail about the HemoCue 301 haemoglobinometer specifications.
- Addition of a Monitoring Plan for SHINE (see section 12d)
- Updating of definitions of malnutrition for infants (Table 14)
- Addition of plans to minimize group harm within the SHINE trial, to sections 8 (e) and 17(h).
- Clarification of procedures for consent and for destruction of audiotape recordings in the ‘Qualitative study of mothers' experiences in receiving the SHINE interventions’ and ‘Developmental Evaluation of the protective play space intervention component’ interviews.

**Summary of changes from Version 5**

**Major Changes**

- Increase recruitment by identifying eligible women at Antenatal Clinics and increasing gestational age inclusion criteria from <18 weeks to <24 weeks gestation using SHINE Form 27 v1 ANC Surveillance Form.

**Minor Changes**

- Minor changes to forms to be compliant with ANC recruitment:
  - Addition of SHINE Form 27_v1_ANC Surveillance Form
  - Change to timing of baseline visit from 10 - 20 weeks, to 10- 26 weeks using SHINE Form13_v4_Baseline Survey Form
  - Change to Consent forms 2,3,4,5 to reflect increase in gestational age eligibility
- To correct research questions for EE Substudy to include both gestational time points.
- Add Module BV to Postnatal Forms SHINE 20_v2_ 3 Month Visit which was mistakenly omitted in _v1.
- To add a consent form for the guardian to use in the case that a mother dies and the baby survives using SHINE Form 28v1 Guardian Consent
- Changed Ministry of Health and Child Welfare (MoHCW) to Ministry of Health and Child Care (MOHCC), their recently altered name.
- To reduce transportation costs in collecting morbidity data from EE substudy mothers by providing these mothers with a cellphone and airtime

**Summary of changes from Version 4**

**Major changes**

- Addition of new substudy (Risk Factors for Miscarriage)
- Change to inclusion criteria: gestational age eligibility increased from <14 weeks to < 18 weeks
- Removal of exclusion criterion: infants with congenital malformations
- Change to timing of baseline visit from 10-16 weeks, to 10-20 weeks
- Addition of one research question to the environmental enteropathy substudy (specific aim 8)
- Addition of 4 new standard of care modules (S1-S4) to the intervention schedule (Table 6)
- Addition of lactulose mannitol test for infants in the environmental enteropathy substudy at 3 months of age
- Change to adverse event and serious adverse event reporting processes

**Minor changes**

- Changes to the sample size calculation for the EE sub-study
- Updated definition of depression in mothers and environmental enteropathy in women and infants (Table 3)
- Updated timepoints in Table 5
- Addition of SHINE_Form25_v1 for collection of home birth data
- Addition of CD4 counts for HIV positive mothers at baseline and at 30-34 weeks gestation (Table 7) and for HIV-infected infants at 3, 6, 12, and 18 months gestation (Table 10)
- Addition of cytokine measurement on saliva samples.
- Table 9: Updated postnatal data collection, addition of birth visit for home births, and stool collection from all infants at 18 months
- Change in timing of informed consent process for the EE sub-study to 32 week visit
- Reduction in number of morbidity diary visits for women in the EE substudy and updated total number of visits for mother-child pairs in the study
- Clarification of loss to follow-up definitions
- Change in Data Safety and Monitoring Board membership from Prof James Hakim to Dr. Tariro Makadzange
- Updated title of Mr Robert Ntozini to Associate Director IT/Data/Statistics.

**Summary of changes from Version 3**

**Major changes**

- Addition of two research questions (specific aims 10 and 11) in the environmental enteropathy substudy
- Clarification of inclusion criteria: women must be within first 14 gestational weeks, live within randomized clusters and be permanent residents in the study districts
- Change in timing of lactulose mannitol test from 5hr to 2hr urine collection
- Increase in blood volume collected from women, from 12ml to 15ml; reduction in blood volume collected from infants, from 5.9ml to 5.4ml
- Addition of 1 month postnatal collection of blood and stool from mother-infant pairs enrolled in the environmental enteropathy substudy
- Removal of 9 month and 15 month postnatal visits
- Change to adverse event and serious adverse event reporting processes

**Minor changes**

- Updated map of clusters
- Additional study outcome definitions added to Table in section 4 (Measurements and Definitions)
- Update to table of visits and table of intervention modules and addition of allowable windows of timing around each visit.
- 7 month antenatal visit renamed 32-week gestation visit
- The Table on pages 26-28 has been updated to reflect a few minor changes to the data collection schedule.
- Minor updates to Research and Implementation table
- Primary care nurses renamed throughout protocol as SHINE Intervention Nurses (INs) or SHINE Data Collectors (DCs); senior nurses renamed as Research Nurse Supervisors, and data mangers as Field Data Supervisors.
- Updated sample sizes for mycotoxin analyses
- Details of methodology for microbiota analysis added
- Addition of hepatitis A serology on infant 18 month blood samples
- Addition of REG-1 ELISA assay to be undertaken on stool samples
- Addition of postnatal forms to protocol: SHINE_ Form 20_v1_3 months visits; SHINE_ Form 21_v1_6 months visits; SHINE_ Form 22_v1_12 months visits; SHINE_ Form 23_v1_18 months visits
- Clarification of definitions of loss to follow-up, and addition of an exit form (SHINE_ Form 24_v1_ Exit form)
- Addition of membership of the DSMB.
- Change from use of cell stabilized tubes to collection of whole lysed blood samples for analysis of PBMCs
- Reference list updated.

**Summary of changes from Version 2**

**Minor changes**

- Addition of justification for HIV testing
- Addition of maternal blood collection to table of antenatal visits
- Change in title from “SHINE Nurse” to “SHINE Nurse Data Collector (DC)”

**Summary of changes from Version 1**

**Major changes**

- Addition of infant and maternal stool collection and infant blood collection at 1 month postnatal visit for mother-infant pairs in environmental enteropathy substudy
- Addition of adverse event and serious adverse event reporting system

**Minor changes**

- Addition of blood volumes to be collected
- Addition of statement that there are no plans for genetic testing on samples
- Addition of intervention-specific brochures to be distributed by Village Health Workers
- Clarification in table of interventions that latrine will be provided at the end of the study to women in the standard care arm
- Addition of insulin-like growth factor binding protein 3 (IGFBP3) measurement on blood samples, and intestinal fatty acid binding protein on urine
- Mycotoxin exposure defined and method of assessment added
- Provision of a calendar to enrolled women to record important dates
- Change in system of notification of eligible women by Village Health Workers from paper form to cellphone SMS
- Addition of mycotoxin exposure assessment at 7 month gestation visit and change in sample size and timepoints for mycotoxin assessment
- Change in screening procedure for women who report vaginal bleeding: consent visit delayed by 2 weeks and women referred to clinic
- Change in stool collection procedure to delay collection for one week after ingestion of lactulose mannitol solution
- Collection of pre-lactulose:mannitol urine sample for urinalysis and microscopy
- Change in clinic referral criteria for anemic women from <7 g/dL to <10 g/dL
- Addition of assessment of end of pregnancy outcome at 1 month postnatal visit
- Change of location of manufacture of lactulose-mannitol solution from local pharmaceutical firm to Zvitambo clean room
- Addition of detail regarding flow cytometry assessments being undertaken
- Addition of questions on maternal health and wellbeing on follow up data collection forms
- Volume of infant blood draw added to EE substudy data collection table
- Definition of loss to follow-up added
- Clarification of HIV testing algorithm and addition of HIV DNA PCR for discordant HIV rapid test results
- Addition of postdoctoral fellows in the investigator list

PI: Jean H Humphrey

**Study Title: SHINE –Sanitation Hygiene Infant Nutrition Efficacy Project**

**IRB#4205**

**PI Version Number/Date: Version 28, October 11^th^ 2021**

Table of Contents

[1. Research Questions 22](#_Toc463944690)

[Main Trial 22](#_Toc463944691)

[Risk Factors for Preterm Birth and Small for Gestational Age (SGA) Sub-study 26](#_Toc463944692)

[Risk Factors for Stillbirth Sub-study 27](#_Toc463944693)

[Risk Factors for Miscarriage Sub-study 27](#_Toc463944694)

[Risk Factors for Neonatal Death Sub-study 28](#_Toc463944695)

[2. Background and Rationale 28](#_Toc463944696)

[3. Participants 29](#_Toc463944697)

[Main Trial 29](#_Toc463944698)

[Environmental Enteric Dysfunction Sub-study 30](#_Toc463944699)

[Risk Factors for Preterm Birth and Small for Gestational Age Sub-study 32](#_Toc463944700)

[Risk Factors for Stillbirth Sub-study 33](#_Toc463944701)

[Risk Factors for Miscarriage Sub-study 33](#_Toc463944702)

[Risk Factors for Neonatal Death Sub-study 34](#_Toc463944703)

[4. Study Procedures and Design 34](#_Toc463944704)

[General Study Design and Methods 34](#_Toc463944705)

[Cluster Construction and Randomization 34](#_Toc463944706)

[Intervention Arms 37](#_Toc463944707)

[Health System Strengthening Prior to Randomized Trial 46](#_Toc463944708)

[Study Procedures in Sequential Order for Women Referred to Study 50](#_Toc463944709)

[Study Procedures in Sequential Order for Village Health Workers 93](#_Toc463944710)

[5. Data Security and Protection of Subject Confidentiality 94](#_Toc463944711)

[a. Applying for a Certificate of Confidentiality? 94](#_Toc463944712)

[b. Data security plan 94](#_Toc463944713)

[c. Description of use of participants’ personal identifiers and plans for disposing of identifiers. 95](#_Toc463944714)

[d. Description of plans for destroying data 95](#_Toc463944715)

[6. Recruitment Process 95](#_Toc463944716)

[7. Consent process and documentation 95](#_Toc463944717)

[a. Countries where the research will take place and the languages into which each consent document will be translated 95](#_Toc463944718)

[The entire project will be conducted in Zimbabwe. 95](#_Toc463944719)

[b. Who will obtain informed consent from participants, how, when and where 95](#_Toc463944720)

[**c.** **Vulnerable population enrolled?** 97](#_Toc463944721)

[d. Waiver of consent requested? 97](#_Toc463944722)

[8. Risks 97](#_Toc463944723)

[a. Description of risks 97](#_Toc463944724)

[b. Frequency and severity of risks 97](#_Toc463944725)

[c. Description of measures to minimize risks 97](#_Toc463944726)

[d. Description of level of research burden 97](#_Toc463944727)

[e. Description of how participant privacy will be protected 98](#_Toc463944728)

[9. Benefits 98](#_Toc463944729)

[a. Direct Benefits 98](#_Toc463944730)

[b. Societal Benefits 99](#_Toc463944731)

[10. Payment 99](#_Toc463944732)

[11. Drug Products, Vitamins, Dietary Supplements and Devices 99](#_Toc463944733)

[a. Drug, Vitamin, and Dietary Supplement products 99](#_Toc463944734)

[b. Medical Devices 101](#_Toc463944735)

[12. Safety monitoring 102](#_Toc463944736)

[a. Participant safety monitoring 102](#_Toc463944737)

[b. Data Safety Monitoring Board (DSMB) 102](#_Toc463944738)

[c. Plans for interim analysis and stopping rules 102](#_Toc463944739)

[13. Plan for reporting unanticipated problems and adverse events. 103](#_Toc463944740)

[14. Other IRBs/Ethics Review Boards 108](#_Toc463944741)

[15. Supervision Roles and Responsibilities 108](#_Toc463944742)

[16. External Investigator Roles and Responsibilities 108](#_Toc463944743)

[17. Oversight plan for studies conducted at non-JHSPH sites 109](#_Toc463944744)

[18. Creation of a biospecimen repository 111](#_Toc463944745)

[a. Source and purpose of the biospecimens 111](#_Toc463944746)

[b. Biospecimen storage 111](#_Toc463944747)

[c. Sharing of biospecimens 111](#_Toc463944748)

[d. Future specimen derivation and processing (cell lines, DNA/RNA, etc.) 111](#_Toc463944749)

[e. Specimen coding and identification 112](#_Toc463944750)

[f. Certificate of Confidentiality protections 112](#_Toc463944751)

[g. Ability of participant to withdraw consent 112](#_Toc463944752)

[h. Data and/or specimen use agreements 112](#_Toc463944753)

[19. Data Coordinating Center 113](#_Toc463944754)

[20. References 113](#_Toc463944755)

**Phase IV. Clinical Trial Research Plan**

We propose to conduct a 2x2 factorial, cluster-randomized, community-based trial in two rural districts of Zimbabwe where HIV prevalence in pregnant women is approximately 15%. Nested within this large trial are four sub-studies each of which will address research questions that are complementary to the main trial. We have organized the sections of the Research Plan by study (main trial and sub-studies). The sub-studies titles are:

- Environmental Enteric Dysfunction (EED) Sub-Study.
- Risk Factors for Pre-Term Birth Sub-Study.
- Risk Factors for Stillbirth Sub-Study.
- Risk Factors for Miscarriage Sub-Study

**NOTE: This trial is Phase 4 of a multi-phased project. During Phases 1, 2, and 3 we conducted surveys to determine baseline water and sanitation coverage in the study area, surveys and formative research to develop and pilot test the interventions, and implemented blanket interventions across the entire study area [strengthening of the Village Health Worker system and implementation of the WHO 2010 Prevention of Mother-to-Child Transmission (PMTCT) Guidance]. All previous phases were reviewed and approved under MRCZ/A/1534.**

## Research Questions

Main Trial

#### Primary Specific Aims:

1. What are the independent and combined effects of a package of interventions to improve household water, sanitation, and hygiene (WASH) and a package of interventions to improve infant feeding practices (IYCF – Infant and Young Child Feeding), when these interventions are initiated during early pregnancy among **HIV negative** women and continued through 18 months post partum, on **stunting** of rural Zimbabwean infants at 18 months of age?
2. What are the independent and combined effects of a package of interventions to improve household water, sanitation, and hygiene (WASH) and a package of interventions to improve infant feeding practices (IYCF), when these interventions are initiated in early pregnancy among **HIV negative** women and continued through 18 months post partum, on **anemia** of rural Zimbabwean infants at 18 months of age?

#### Secondary Specific Aims:

1. What are the independent and combined effects of a package of interventions to improve household water, sanitation, and hygiene (WASH) and a package of interventions to improve infant feeding practices (IYCF), when these interventions are initiated in early pregnancy among **HIV positive** women and continued through 18 months post partum, on **stunting** of rural Zimbabwean infants at 18 months of age?
2. What are the independent and combined effects of a package of interventions to improve household water, sanitation, and hygiene (WASH) and a package of interventions to improve infant feeding practices (IYCF), when these interventions are initiated in early pregnancy among **HIV positive** women and continued through 18 months post partum, on **anemia** of rural Zimbabwean infants at 18 months of age?
3. What is the effect of the IYCF intervention on uptake of improved infant feeding practices by maternal/infant HIV status, specifically:
   1. Infant diet quality as assessed by WHO IYCF indicators (WHO 2010)
   2. Infant nutrient intake from complementary foods assessed by 24 hour dietary recall
   3. Appropriate use of Nutributter from 6 to 18 months
4. What is the effect of the WASH intervention on the 5 key behaviors it promotes by maternal/infant HIV status?
5. Proper disposal of animal and human feces
6. Handwashing with soap (HWWS) after fecal contact
7. Point-of-use chlorination of drinking water
8. Protecting children from ingestion of dirt and feces
9. Feeding baby freshly prepared foods, or reheating leftover food
10. For each randomized intervention (IYCF and WASH), is the impact on **stunting** of children at 18 months of age modified by the following factors in addition to maternal/infant HIV status?:
11. Household and community socioeconomic and demographic characteristics. For example, maternal age, size of the household, socioeconomic status, etc.
12. Village Health Worker (VHW) performance assessed by degree of transfer of correct knowledge to participating mothers and the quality of VHW interpersonal approach
13. Maternal capacities (1) social support for mothering or the mother’s social networks, (2) access to and control of resources, (3) depression and stress, (4) roles, priorities and time, (5) perceived physical health, and (6) mothering self-efficacy
14. For each randomized intervention (IYCF and WASH), is the impact on **anemia** at 18 months of age modified by the following factors in addition to maternal/infant HIV status?
15. Household and community socioeconomic and demographic characteristics
16. Village Health Worker (VHW) performance assessed by degree of transfer of correct knowledge to participating mothers and the quality of VHW interpersonal approach
17. Maternal capacities (1) social support for mothering or the mother’s social networks, (2) access to and control of resources, (3) depression and stress (4) roles, priorities and time, (5) perceived physical health, and (6) mothering self-efficacy
18. What is the effect of the WASH intervention on diarrhea prevalence among infants 0-18 months of age? Is this different among children born to HIV positive and negative mothers?
19. What is the prevalence of exclusive breastfeeding among all infants enrolled in the trial by maternal/infant HIV status?
20. What is the prevalence of anemia among pregnant women at 10-26 weeks by HIV, mycotoxin and schistosomiasis status?
21. What is the prevalence of schistosomiasis among pregnant women living in areas of high prevalence among school-aged children?
22. What is the prevalence of mycotoxin exposure among pregnant women?
23. What is the prevalence and severity of EED among pregnant women at 10-26 weeks and 30-34 weeks gestation by maternal HIV and mycotoxin status, as assessed by:
    1. Maternal gut inflammation

Indicators of Environmental Enteric Dysfunction pathway

- 1. Maternal gut permeability
  2. Maternal gut microbial translocation
  3. Maternal systemic immune activation
  4. Maternal growth hormone axis

1. What is the association between maternal EED and infant birth outcomes (mean birth weight, mean birth length, mean gestational age at delivery by maternal/infant HIV status)?
2. What is the association between maternal schistosomiasis and infant birth outcomes (mean birth weight, mean birth length, mean gestational age at delivery by maternal/infant HIV status), and are these associations mediated through inflammation?
3. What is the association between maternal mycotoxin exposure and infant birth outcomes (mean birth weight, mean birth length, mean gestational age at delivery by maternal/infant HIV status)?
4. What is the association between EED and early child development at 24 months of age, stratified by maternal HIV status?
5. What are the independent and combined effects of a package of interventions to improve household water, sanitation, and hygiene (WASH) and a package of interventions to improve infant feeding practices (IYCF – Infant and Young Child Feeding), when these interventions are initiated during early pregnancy among **HIV negative** women and continued through 18 months post partum, on **early child development** of rural Zimbabwean infants at 24 months of age?
6. What are the independent and combined effects of a package of interventions to improve household water, sanitation, and hygiene (WASH) and a package of interventions to improve infant feeding practices (IYCF – Infant and Young Child Feeding), when these interventions are initiated during early pregnancy among **HIV positive** women and continued through 18 months post partum, on **early child development** of rural Zimbabwean infants at 24 months of age?
7. What are the independent and combined effects of a package of interventions to improve household water, sanitation, and hygiene (WASH) and a package of interventions to improve infant feeding practices (IYCF – Infant and Young Child Feeding), when these interventions are initiated during early pregnancy among **HIV negative** women and continued through 18 months post partum, on **school-aged child growth and function** of rural Zimbabwean infants at 7 years of age?
8. What are the independent and combined effects of a package of interventions to improve household water, sanitation, and hygiene (WASH) and a package of interventions to improve infant feeding practices (IYCF – Infant and Young Child Feeding), when these interventions are initiated during early pregnancy among **HIV positive** women and continued through 18 months post partum, on **school-aged child growth and function** of rural Zimbabwean infants at 7 years of age?
9. What are the differences in growth, health, physical function and cognition by randomized trial arm at 7 years of age?
10. What are the differences in growth, health, physical function and cognition between HIV-exposed uninfected and HIV-unexposed children at 7 years of age?
11. Which early-life exposures influence school-age growth, physical function and cognition?

**Environmental Enteric Dysfunction (EED) Sub-study**

The purpose of the EED sub-study is to examine hypothesized causal pathways of each intervention, stratified by maternal/infant HIV status:

#### Primary Specific Aims:

1. What are the effects of the WASH intervention on each of the following domains of the hypothesized biomedical causal pathway between poor sanitation/hygiene and child **stunting** at 1, 3, 6, 12, and 18 months of age?
   1. Infant gut inflammation

Indicators of Environmental Enteric Dysfunction pathway

- 1. Infant gut permeability
  2. Infant gut microbial translocation
  3. Infant systemic immune activation
  4. Infant growth hormone axis

1. For the domains listed in #1, what is the association between the variability in each upstream domain with that of the downstream domains (upstream=a and downstream=e along a continuum from a to e) among all children and stratified by intervention group?
2. What is the relative importance of EED vs. diarrhea in mediating the effects of the WASH intervention on stunting?
3. What are the effects of the WASH intervention on each of the following domains of the hypothesized biomedical causal pathway between poor sanitation/hygiene and child **anemia** at 1, 3, 6, 12, and 18 months of age?
   1. Infant gut inflammation

Indicators of Environmental Enteric Dysfunction pathway

- 1. Infant gut permeability
  2. Infant gut microbial translocation
  3. Infant systemic immune activation
  4. Infant hepcidin production as assessed by plasma hepcidin levels

1. Are the associations observed in specific aims 1-4 above modified by HIV status of the mother/infant?
2. What is the effect of the WASH intervention on diarrhea incidence and duration among infants 0-18 months of age, stratified by maternal/infant HIV status?
3. What are the associations between inflammatory markers (serum TNF-α, CRP, sCD14 and AGP), iron status and anemia among pregnant women at 10-26 weeks and 30-34 weeks gestation according to their HIV status?
4. What are the associations between maternal and infant EED, and between maternal EED and infant postnatal stunting?
5. Is EED more severe among infants who are mixed fed compared to infants who are exclusively or predominantly breast fed, stratified by maternal/infant HIV status?
6. What is the prevalence of mycotoxin exposure among infants between birth and 18 months, and what is the association between mycotoxin exposure, EED and stunting between birth and 18 months?
7. What is the impact of the maternal microbiota, infant breastfeeding practices and the WASH intervention on early (0-6 months) intestinal microbiota development and on infant growth between 0 to 6 months?
8. What is the impact of the early microbiota, the IYCF intervention and the WASH intervention on late intestinal microbiota development and on infant growth between 6-18 months?
9. What is the association between infant EED and infant rotavirus vaccine immunogenicity?
10. What are the effects of the WASH intervention on infant rotavirus vaccine immunogenicity?
11. What are the associations between aflatoxin exposure and maternal EED among pregnant women at 10-26 weeks gestation according to their HIV status?

### Risk Factors for Preterm Birth, Small for Gestational Age (SGA) Sub-study, Low Birth Weight and Early Stunting

#### Primary Specific Aims:

1. What is the association between maternal EED and preterm/SGA,* low birth weight and stunting at 1 month of age,stratified by maternal/infant HIV status?
2. What is the association between maternal anemia measured at 10-26 weeks and 30-34 weeks gestation and preterm/SGA low birth weight and stunting at 1 month of age,* stratified by maternal/infant HIV status?
3. What is the association between maternal schistosomiasis measured at 10-26 weeks and 30-34 weeks gestation and preterm/SGA,* low birth weight and stunting at 1 month of age,stratified by maternal/infant HIV status?
4. What is the association between maternal mycotoxin exposure measured at 10-26 weeks and 30-34 weeks gestation and preterm/SGA,* low birth weight and stunting at 1 month of age,stratified by maternal/infant HIV status?

*Four infant groups will be defined based on gestational age and weight (categorized as small-for-gestational age (SGA) or appropriate-for-gestational age (AGA)): preterm/AGA; preterm/SGA; term/SGA and term/AGA (reference group). We will also explore associations between each exposure and stunted-for-gestational age (birth length <10^th^ centile) to evaluate impact on birth length as well as birth weight. We will also use low birth weight (<2.5kg) to define cases, since this captures both preterm and SGA, and enables inclusion of infants with inaccurate gestational ages at birth (due to incorrect last menstrual period dates); we will also use birth weight as a continuous variable. Finally, stunting (LAZ<-2) at 1 month of age will be used instead of birth length as a case definition to evaluate the impact of each exposure on early stunting.

### Risk Factors for Stillbirth Sub-study

#### Primary Specific Aims:

1. What is the association between maternal EED and stillbirth stratified by HIV status?
2. What is the association between maternal anemia measured at 10-26 weeks and 30-34 weeks gestation and stillbirth stratified by maternal HIV status?
3. What is the association between maternal schistosomiasis measured at 10-26 weeks and 30-34 weeks gestation and stillbirth stratified by maternal HIV status?
4. What is the association between maternal mycotoxin exposure measured at 10-26 weeks and 30-34 weeks gestation and stillbirth stratified by maternal HIV status?

### Risk Factors for Miscarriage Sub-study

#### Primary Specific Aims:

1. What is the incidence of miscarriage, and what are the epidemiological exposures associated with miscarriage in rural Zimbabwean women?
2. What is the association between maternal EED and miscarriage stratified by HIV status?
3. What is the association between maternal anemia measured at 10-26 weeks and 30-34 weeks gestation and miscarriage stratified by maternal HIV status?
4. What is the association between maternal schistosomiasis measured at 10-26 weeks and 30-34 weeks gestation and miscarriage stratified by maternal HIV status?
5. What is the association between maternal mycotoxin exposure measured at 10-26 weeks and 30-34 weeks gestation and miscarriage stratified by maternal HIV status?

### Risk Factors for Neonatal Death Sub-study

#### Primary Specific Aims:

1. What is the association between maternal EED and neonatal death stratified by HIV status?
2. What is the association between maternal anemia measured at 10-26 weeks and 30-34 weeks gestation and neonatal death stratified by maternal HIV status?
3. What is the association between maternal schistosomiasis measured at 10-26 weeks and 30-34 weeks gestation and neonatal death stratified by maternal HIV status?
4. What is the association between maternal mycotoxin exposure measured at 10-26 weeks and 30-34 weeks gestation and neonatal death stratified by maternal HIV status?

## Background and Rationale

Child undernutrition remains a large problem in developing countries with substantial adverse sequelae (Black 2008, Victora 2008). It develops during the first two years of life, when mean weight-for-age and length-for-age Z-scores (WAZ and LAZ, respectively) of children living in Africa and Asia plunge to about –2.0 followed by little or no recovery (Victora 2010).

Under the plausible assumption that children grow poorly because they don’t eat enough of the right foods, enormous research effort has focused on identifying dietary solutions. Numerous studies have tested a myriad of nutrient-dense foods and supplements, nutrition education interventions, and infant feeding behavioral-change strategies. A recent review of 38 of the best of these studies revealed that most (but not all) achieved statistically significant growth effects: compared to controls, intervention children gained 0–760 g more weight (0.0 – 0.76 WAZ) and grew 0–1.7 cm taller (0.0 – 0.64 LAZ) by 12 to 24 months (Dewey 2008). Enthusiasm is tempered, however, by the realization that the growth effect of even the most successful of these studies (~+0.7 Z) is equivalent to about one-third of the average deficit of Asian and African children (~–2.0 Z).

Prevalent diarrhea has also been implicated. In a pooled analysis of nine studies that together collected diarrhea and growth data on 1393 children, the odds of stunting at 24 mo increased multiplicatively by 2.5% per episode of diarrhea, and 25% of all stunting among 24-mo-old-children was attributable to having five or more episodes of diarrhea in the first two years of life (Checkley 2008). However, other authors have contended that the effect of diarrhea on permanent stunting is small because children grow at “catch-up” velocities between illness episodes (Briend 1990). Hence, the relative contribution of diarrhea to undernutrition, and consequently, the potential impact that diarrhea control programs (i.e., sanitation/hygiene interventions) could have on growth has been unresolved. The recent *Lancet* Undernutrition Series estimated that sanitation/hygiene interventions implemented with 99% coverage would reduce diarrhea incidence by 30%, which would in turn reduce prevalent stunting by a modest 2.4% (Bhutta 2008).

This study will test the hypothesis that a major cause of child undernutrition is Tropical or Environmental Enteropathy, more recently renamed Environmental Enteric Dysfunction (Keusch 2013) a ***subclinical*** condition of the small intestine caused by poor environmental sanitation and characterized by villous atrophy, crypt hyperplasia, increased permeability, inflammatory cell infiltrate, and modest malabsorption.^8^ Furthermore, the study will test the hypothesis that the primary pathway from sanitation/hygiene to child undernutrition is via EED rather than diarrhea. If this is true, previous analyses may have substantially underestimated the effect of sanitation/hygiene on growth because the effect was modeled entirely through diarrhea, and sanitation/hygiene interventions may have been undervalued because they have been appraised primarily for their impact on diarrhea.

## Participants

### Main Trial

#### Study Population

Study participants will be women who are rural residents of Chirumanzu or Shurugwi districts in Zimbabwe and who become pregnant during the enrollment period of the trial and are identified and consent to participation before --24 weeks of gestation, and their live born infants. A total of 4800 women will be enrolled.

All Village Health Workers working in the two districts will be approached to provide their informed consent for assessment of their work capacity, performance and perceptions about the motivational and supervisory characteristics of their work. A total of 430 VHWs will be enrolled.

#### Inclusion/Exclusion Criteria

*Inclusion:* Pregnant women residing in randomized clusters within the study districts, whose pregnancy is confirmed by a urine pregnancy test.

*Exclusion*:

- Women residing in the study districts who become pregnant during the enrollment period but do not consent to join the trial.
- Women who reside in urban areas of these two districts
- Women who are resident as employees at a homestead or who are living on rented premises

#### Sample Size

A total sample size of 4800 pregnant women will be enrolled, 1200 in each of 4 treatment arms. The primary outcomes are growth and anemia of children at 18 months of age, stratified by maternal HIV status. The main trial inference will be based on outcomes of infants of HIV-negative women, of whom we expect at least 4080 (more likely, 4200) based on a 15% HIV-positive rate among pregnant women. Allowing for 20% loss of evaluable infants at 18 months (this includes pregnancy losses, infant mortality and loss to follow up), we expect about 816 measurements in each of the four study arms. With Type I error of 5%, and power of 90%, and control group stunting (height-for-age Z score <-2) of 30%, and the assumption that the effects of the two interventions will be additive, we will be able to detect a reduction of about 8 percentage points, i.e. from 30% to 22.0%, for either of the interventions(Hayes, 1999).Among the expected 720 pregnancies in HIV positive women, we expect a minimum of 25% loss through fetal loss, etc. resulting in 540 total children at 18 months of age, or 135 per treatment group.

This is based on an assumed coefficient of variation of the true proportions of 0.43, and an effective loss of 33% of sample size due to cluster size variability, which corresponds to a design effect of 2.5. Note that the largest within-unit correlation reported by Katz for stunting in several countries was only 2.6 (Katz, 1995). Using the design effect of 2.5, we can detect a shift of 0.2 weight-for-age and length-for-age Z-scores, assuming a within-randomized-cluster variance of 1.25.

*Hemoglobin:* Assuming a SD at 18 months of 12.8 g/L, we will be able to detect a shift of 2.6 g/L.

***Sample size update:*** The final sample size will be 5282 enrolled women, reflecting 10% over-recruitment to provide sufficient power to conduct sensitivity analyses. This is particularly important for the WASH arms, because the initial subcontractor (Oxfam UK) failed to ensure timely installation of latrines and Tippy Taps for the first 730 women enrolled in WASH arms.

***Identifiers*** Names, addresses, and antenatal booking number will be collected on all participants at baseline and also recorded on all subsequent study instruments to confirm identity. Household GPS location will be collected at one visit. Each mother-infant pair will be given a unique study ID which will be used to identify all data collected from the pair and will also be recorded on all study instruments. When the data are transferred to the study office in Harare, all personal identifiers except the Study ID will be stripped from the files before adding the data to the database. A list matching personal identifiers and unique identifiers will be kept in a password-protected file on a computer in a locked office which will only be accessible by research staff.

### Environmental Enteric Dysfunction Sub-study

#### Study Population

The study population for this sub-study will consist of two groups:

1. HIV-negative mothers and their infants.

*Inclusion:* women enrolled in the trial from May 1st 2014 onwards who test HIV-negative at their last HIV test done during antenatal care and who consent into the sub-study at the 32 week gestation visit, or as soon as possible thereafter.

*Exclusion*: 1. HIV-negative women enrolled in the trial prior to May 1st 2014, and women who refuse to learn their HIV test result. 2. HIV-negative women enrolled in

the trial after May 1, 2014 whose child has a major non-fatal abnormality that

is likely to directly affect gut health/function or stature (e.g. neural tube defects,

cerebral palsy, Down syndrome)

1. All enrolled HIV-positive mothers (~600) and their infants.

*Inclusion:* women enrolled in the trial who test HIV-positive at any time

*Exclusion: 1.* HIV-negative women, and women who refuse to learn their HIV test result. 2. HIV-positive women enrolled in the trial after May 1, 2014 whose child has

a major non-fatal abnormality that is likely to directly affect gut health/function or

stature (excluding HIV infection) (e.g. neural tube defects, cerebral palsy, Down

syndrome).

#### Sample Size

This original sample size was decided based on calculations of lactulose:mannitol ratio, endotoxin core antibody and total IgG. We used conservative estimates of standard deviations of 0.3 for the lactulose:mannitol, 101 MUx10^3^/L for endotoxin core antibody, 2.78 g/L for total IgG, and assumed β=0.20, α=0.05, and two-sided tests. With 150 infants per group we will be able to detect effect sizes ranging from 35% to 40% of one standard deviation for each of these indicators when comparing each of the 3 treatment arms to the control group (i.e. without utilizing the factorial design). With the factorial design, if the effects of the two factors are additive, we will be able to detect effect sizes on the order of 25% of one standard deviation. These calculations account for a design effect of 1.25; we expect this to be relatively small, given the small numbers of women sampled per cluster. These effect sizes are small compared to the observed differences between infants reared in more vs. less sanitary environments in observational studies, which were 50-100% of one standard deviation.

The sample size was subsequently increased by allowing all HIV-negative women and their infants who joined the trial from May 1^st^ 2014 onwards to enrol, to ensure at least 150 children per trial arm have longitudinal assessments of EED, allowing for missed samples and loss to follow-up. Assuming an average of 2-3 infants per cluster, and a CV of 0.25, this would provide >80% power to detect a shift of 0.25 SD or greater for any individual biomarker between WASH and non-WASH arms, and 0.33 SD between any two trial arms.

#### Identifiers

As per Main Trial.

### Risk Factors for Preterm Birth, Small for Gestational Age, Low Birth Weight and Early Stunting Sub-study

### *Study Population*

### The study population for this sub-study will consist of pregnancies in women who are enrolled in the trial that are delivered alive either i) at less than 37 completed weeks of gestation (preterm) but appropriate for gestational age (AGA; ie weight >10^th^ centile); ii) at term (>37 weeks) but small-for-gestational age (SGA; ie weight <10^th^ centile); iii) preterm and SGA or iv) term and AGA (reference group). We will use incidence density sampling to select cases and controls, using the same reference group (term/AGA) for each of the three cases by undertaking group matching, based on maternal HIV status and gestational age at the time of maternal baseline specimen collection. We will also use low birth weight (<2.5kg) to define cases in a subsequent analysis, since this captures both preterm and SGA, and enables inclusion of infants with inaccurate gestational ages at birth (due to incorrect last menstrual period dates); we will also repeat analyses using birth weight as a continuous variable outside of the case-control design. Finally, stunting (LAZ<-2) at 1 month of age will be used (instead of birth length, which is not available for most infants) to define cases, to evaluate the impact of each exposure on early stunting.

### *Inclusion/Exclusion Criteria*

### *Inclusion:* women enrolled in the trial whose infants are SGA/term, SGA/preterm, AGA/preterm (all case groups) compared to women whose infants are AGA/term (control group). For low birth weight analysis, inclusion of all women whose infants were low birth weight (<2.5kg; cases) compared to women whose infants were not low birth weight (>2.5kg; controls). For early stunting analysis, inclusion of all women whose infants had LAZ<-2 at 1 month of age (cases) compared to women whose infants had LAZ>-2 (controls) at 1 month of age.

### *Exclusion*: women whose infants are AGA/term (or, depending on substudy, weight >2.5kg, or LAZ at 1 month >-2) but not selected as controls.

Sample Size

~480-960 women and their infants (240-480 mothers of SGA and preterm infants in the combination groups shown above and a group of control mothers of AGA/term infants). Since we are comparing all case groups with the same control group, we will select a higher number of controls (typically SQRT(k) x no of cases, where k = number of case groups). Taking the conservative number of 240, matched 1:1, and assuming a correlation coefficient for exposure between matched cases and controls of 0.2, Types I and II error of 0.05 and 0.80, and a prevalence of 0.25 of a given risk factor among controls, then we will be able to detect an odds ratio of 1.9 or larger (for prevalence of 0.10, detectable OR is 2.4)(Dupont,1990).

#### Identifiers

As per the Main Trial.

### Risk Factors for Stillbirth Sub-study

#### Study Population

This is a 1:2 case control study that will use incidence density sampling. The study population for this sub-study will consist of outcomes of pregnancies in women who are enrolled in the trial that are delivered stillborn at 28 completed weeks of gestation or later (cases), each matched to 2 pregnancies in women enrolled in the trial that are delivered alive at 28 completed weeks of gestation or later (controls), matched on infant gender, maternal HIV status, and intervention arm. Stillbirth rates are estimated to be 3-4%; thus we will enroll 140-190 cases and 280-380 controls.

#### Inclusion/Exclusion Criteria

*Inclusion:* women enrolled in the trial who deliver a stillborn infant, each matched to 2 control women who did not deliver a stillborn infant.

*Exclusion*: women who deliver at <28 weeks gestation.

Sample Size

140-190 stillborn infants and 280-380 matched controls. Taking the conservative number of 140, matched 1:2, and assuming a correlation coefficient for exposure between matched cases and controls of 0.2, Types I and II error of 0.05 and 0.80, and a prevalence of 0.25 of a given risk factor among controls, then we will be able to detect an odds ratio of 2.3 or larger (for prevalence of 0.10, detectable OR is 3.0) (Dupont, 1990).

#### Identifiers

As per the Main Trial.

### Risk Factors for Miscarriage Sub-study

#### Study Population

This is a 1:2 case control study that will use incidence density sampling. The study population for this sub-study will consist of outcomes of pregnancies in women who are enrolled in the trial and experience spontaneous miscarriage prior to 28 completed weeks of gestation (cases), each matched to 2 pregnancies in women enrolled in the trial who do not experience spontaneous miscarriage prior to 28 completed weeks of gestation (controls), matched on maternal HIV status, and intervention arm. Miscarriage rates are estimated to be 5-10%; thus we will enroll 240-480 cases and 280-960 controls.

#### Inclusion/Exclusion Criteria

*Inclusion:* women enrolled in the trial who experience spontaneous miscarriage each matched to 2 control women.

*Exclusion*: women who undergo a traditional or medical procedure to induce abortion

Sample Size

240-480 women experiencing miscarriage and 480-960 matched controls. Taking the conservative number of 240, matched 1:2, and assuming a correlation coefficient for exposure between matched cases and controls of 0.2, Types I and II error of 0.05 and 0.80, and a prevalence of 0.25 of a given risk factor among controls, then we will be able to detect an odds ratio of 1.9 or larger (for prevalence of 0.10, detectable OR is 2.4)(Dupont,1990).

#### Identifiers

As per the Main Trial.

### Risk Factors for Neonatal Death Sub-study

#### Study Population

The study population for this sub-study will consist of the mothers of infants who are delivered alive but die within the first 28 days of life from any cause, stratified as early and late neonatal deaths, each matched to 2 women enrolled in the trial whose babies are delivered alive and survive the first 28 days of life, matched on infant gender and gestational age, maternal HIV status, and intervention arm. The neonatal death rate is estimated to be around 3%; thus we will enroll approximately 140 cases and approximately 280 controls.

#### Inclusion/Exclusion Criteria

*Inclusion:* women enrolled in the trial who deliver a liveborn infant who dies within 28 days of life, each matched to 2 control women on infant gender and gestational age, maternal HIV status, and intervention arm.

*Exclusion*: Infants with life-threatening congenital abnormalities.

Sample Size

Approximately 140 neonatal deaths and 280 matched controls. Taking the conservative number of 140, matched 1:2, and assuming a correlation coefficient for exposure between matched cases and controls of 0.2, Types I and II error of 0.05 and 0.80, and a prevalence of 0.25 of a given risk factor among controls, then we will be able to detect an odds ratio of 2.3 or larger (for prevalence of 0.10, detectable OR is 3.0) (Dupont, 1990).

#### Identifiers

As per the Main Trial.

## Study Procedures and Design

### General Study Design and Methods

The project will be conducted in two rural districts of Zimbabwe (Chirumanzu and Shurugwi); it will be a community-based, 2X2 factorial cluster-randomized trial among 4800 households in which a woman becomes pregnant during the recruitment period. Our overall approach is to implement a multi-sector project in which randomized interventions are delivered by Village Health Workers who are supervised by Ministry of Health and Child Care (MOHCC) nurses in delivering their regular duties and by research staff in delivering the additional tasks required by the trial. Research staff will measure the quality and extent of delivery of randomized and non-randomized services, behavioral responses to and uptake of the interventions, and trial outcomes. To maintain neutrality of the research staff to the interventions, we will strictly separate the functions and supervision of the Village Health Workers implementing the interventions from the research staff measuring study outcomes.

### Cluster Construction and Randomization

#### Cluster Definition

The study area has been divided into 212 clusters, each equivalent to the catchment area of 1 to 3 Village Health Workers. Digital maps of administrative boundaries of the study area were obtained from the Central Statistics Office and using Google Earth, the positions of all homesteads and key landmarks (roads, clinics, schools, rivers) for the 2 districts were plotted. Large scale maps were created including all details and Zvitambo staff met with each of the 320 Village Health Workers and asked them to identify the borders of their catchment areas on the maps. Data were also gathered from the Village Health Worker registers regarding the number of households and reproductive-aged women in their catchment, number of currently pregnant women, and number of <2 year old children. Cluster boundaries were finalized by grouping together Village Health Workers who work together or whose catchment areas include homesteads that are close to the catchment boundaries into the same cluster and putting VHWs that worked alone into one cluster while also attempting to achieve about the same number of households, pregnant women, and young children in each cluster. The resulting map is shown below (Figure 1); where the coloured lines represent ward boundaries and black lines represent VHW boundaries and each different fill color represents a cluster.

**Figure 1 – SHINE clusters**


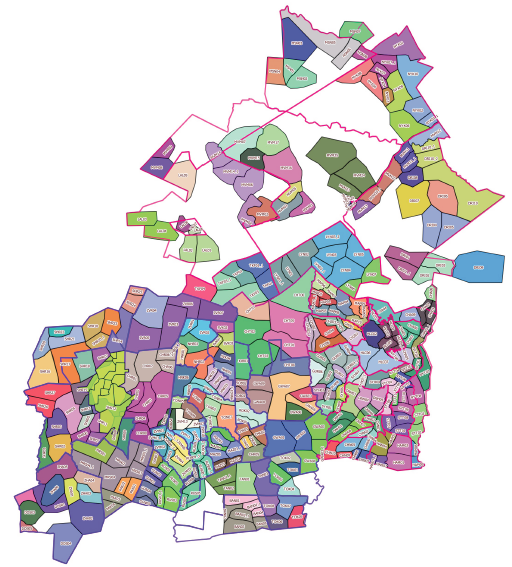


#### Randomization

Clusters will be randomized using a highly restricted randomization scheme. Balance will be achieved for key variables to within 5-10 percentage points with balance for each pair of trial arms (6 combinations) and each of the marginal intervention comparison arms (arms 1+2 vs. 3+4 and 1+3 vs. 2+4). The key variables are:

1. Predominant settlement type (traditional communal; resettled)
2. Number of women aged 15-49 years
3. Sanitation coverage (% of households with at least one latrine; % with a latrine that is half or less full; % with a handwashing facility)
4. Water access coverage (% of households with a perennial, functioning water source <500m away; and % with such a source >=1500m)
5. Mean distance to nearest health facility
6. Geography: balance on mean and variance of latitude and longitude; guarantee each Ward has at least two of the four trial arms represented.
7. District: there will be the same number (within one) of each type of treatment arm in each district (there are 111 units in one district, 101 units in the other, so exact balance within each district is not possible).
8. Village Health Worker characteristics: balance on time-in-post (the VHWs in post received their initial training in either 2001, 2011 or 2012) and number of VHWs in the cluster

Randomization will be conducted by Professor L Moulton using a published method (Moulton, 2004). A computer program will generate millions of random permutations, enumerating those that meet specified balance criteria; a check will be made for bias and validity of the scheme on 5000 such acceptable allocations. Then, from the acceptable allocations that also meet bias and validity specifications, 10 allocations will be randomly selected. Each randomization scheme will divide the 212 randomization units into 4 groups of 53 units. These 10 schemes will be numbered 1-10. Each scheme will be printed on a separate piece of paper with each of the 4 groups of 53 clusters printed in columns labeled A, B, C, and D. The final assignment of the four treatment conditions to the 212 randomization units will be chosen during a forum with community leadership. In their presence, 10 ping pong balls (numbered 1-10) will be placed in an opaque bucket. A representative will select one ball from the bucket held above his/her head. The draw will identify which of the 10 numbered allocations will be used. Then, four balls (labeled A, B, C, and D) will be placed in one bucket, and four balls (labeled with the 4 interventions – see Table below) will be placed in a second bucket. Representatives will draw a ball from the first bucket and a ball from the second bucket, pairing a group of clusters with one of the four intervention arms. This will be repeated twice more to pair the next two groups of clusters with two more intervention arms. The remaining balls in the two buckets will form the final pairing. This second stage is included to provide an additional impression of impartiality/randomness and a further opportunity for participation of the community leadership.

### Intervention Arms

The two active interventions to be studied in this 2X2 Factorial trial are:

- *Intervention 1*: a package of interventions to improve household sanitation and hygiene (WASH)
- *Intervention 2*: a package of interventions to improve infant feeding (IYCF).

The Standard Care interventions are the blanket interventions that were developed and implemented during Phase II of this project and have been approved previously by the IRB. The components of the four intervention arms are detailed in Table 1.

| **Table 1: Randomized Arms** | |
| --- | --- |
| **Standard Care: (Ball labeled “Latrine Later”)**   - Exclusive breastfeeding promotion for all infants, birth to 6 months - Strengthened PMTCT services - Strengthened Village Health Worker system - Latrine provided at end of study | **WASH: (Ball labeled “Latrine Now”)**   - Standard care interventions - Provide household ventilated pit latrine, water treatment solution (Waterguard), monthly liquid soap, play mat and play yard. - Provide interpersonal communication interventions promoting feces disposal in a latrine, HWWS, drinking water treatment, hygienic weaning food preparation, and preventing babies from putting dirt and animal feces in their mouths. |
| **IYCF: (Ball labeled “Latrine Later and Nutributter”)**   - Standard care interventions - Provide 20 g/d Nutributter from 6-18 months - Provide interpersonal communication interventions promoting optimal use of locally available foods for complementary feeding after 6 months, continued breastfeeding and feeding during illness. | **Sanitation/Hygiene AND Nutrition: (Ball labeled “Latine Now and Nutributter”)**   - Standard care interventions - All WASH interventions - All IYCF interventions |

Interventions will include both commodities (i.e., “hardware”) and behavior change communication (i.e., “software”). These components are described for each type of intervention in Table 2 below:

**Table 2: Components of SHINE interventions**

|  | IYCF | WASH |
| --- | --- | --- |
| Intervention Objective | To achieve dietary intake that meets all nutrient requirements by all children in IYCF clusters everyday between birth and 18 months of life. | To prevent all fecal ingestion by all children in WASH clusters everyday between birth and 18 months of life. |
| Commodities | Nutributter, 20 g/day from 6-18 months of age | - Ventilated pit latrine, or upgrades to existing latrine to meet MOHCC standard - 2 Tippy Tap handwashing facilities - Liquid soap for Tippy Taps - Water Guard water treatment solution - Play mat - Play yard (fence/gate) |
| Core Messages | - - - 1. Feed 20 g Nutributter to your baby every day       2. Prepare and feed enriched thick porridge to your baby       3. Feed a variety of foods to your baby including animal foods, bean powder, fruits, and vegetables       4. Your baby can eat all foods that an adult can eat if it is processed so baby can chew and swallow it       5. Continue feeding your baby during and after illness | 1. Dispose of all human and animal feces in the latrine 2. Wash hands with soap after fecal contact and before preparing, eating or feeding food 3. Treat all drinking water with Water Guard 4. Put your baby in a clean protected play area where he cannot access dirt and feces when he is playing or eating 5. Feed your baby freshly prepared food, or boil leftover food before feeding |

These modules and interactive tools are based on formative research and were piloted during Phase III of this project.

**Measurements and Definitions**

A number of parameters will be assessed in this trial that could be measured or defined in a number of ways. Table 3 shows a listing of definitions that will be used in the study. Some of these may be subject to change if more accurate or less costly approaches to measurement become available during the study period.

**Table 3: Definitions used in SHINE**

| **Parameter** | | **Definition/Assessment Method** | |
| --- | --- | --- | --- |
| Environmental Enteric Dysfunction in Pregnant Women | | Assessed through five domains of the hypothesized causal pathway, as outlined on page 5. | |
| Environmental Enteric Dysfunction in Infants | | Assessed through five domains of the hypothesized causal pathway, as outlined on page 5. | |
| Gut Inflammation in women and infants | | Increased concentrations of fecal neopterin, alpha-1 antitrypsin and myeloperoxidase. | |
| Gut microbial translocation in women and infants | | Increased concentrations of plasma Endotoxin Core Antibody, lipopolysaccharide, and soluble CD14 | |
| Gut damage/absorptive capacity and permeability in women and infants | | Assessed by intestinal fatty acid binding protein (I-FABP), plasma citrulline and lactulose:mannitol ratio | |
| Systemic immune activation in women and infants | | Elevated serum levels of ferritin, CRP, AGP, kynurenine tryptophan ratio, and T-cell activation | |
| Infant growth hormone axis | | Depressed plasma levels of insulin like growth factor-1 (IGF-1) and collagen X (a marker of growth plate activity) | |
| Aflatoxin exposure in women and infants | | Detectable AFB1-lysine in plasma and detectable AFM1 in urine | |
| Fumonisin exposure in women and infants | | Detectable FB1 in urine | |
| Deoxynivalenol exposure in women and infants | | Detectable DON in urine | |
| Zearalenone exposure in women and infants | | Detectable zearalenone in urine | |
| Ochratoxin A exposure in women and infants | | Detectable ochratoxin A in urine | |
| T-2 exposure in women and infants | | Detectable T-2 in urine | |
| Maternal and infant intestinal microbiota | | 16S rRNA and whole genome sequencing of DNA and RNA from stool to define the composition and function of the microbial community that inhabits the human intestine. | |
| Maternal anemia | | Hemoglobin < 120 g/L as assessed using the Hemocue hemoglobinometer | |
| Maternal severe anemia | | Hemoglobin <70 g/L as assessed using the Hemocue hemoglobinometer | |
| Maternal iron status | | Plasma ferritin level adjusted for CRP | |
| Infant anemia | | Hemoglobin < 105 g/L as assessed using the Hemocue hemoglobinometer | |
| Infant severe anemia | | Hemoglobin < 70 g/L as assessed using the Hemocue hemoglobinometer | |
| Infant rotavirus immunogenicity | | Measurement of rotavirus IgA titre in plasma | |
| Infant polio vaccine immunogenicity | | Measurement of polio virus IgA titre in plasma | |
| Low birth weight | | Infant born weighing <2500 g within 72 hours following delivery | |
| Preterm birth | | Infant born prior to completing 37 weeks gestation | |
| Stunted for gestational age | | Infant with length at birth less than the 10th percentile of WHO standard for term infants (WHO 2006) and a new fetal growth standard which will be released by the end of our trial (Villar 2010) | |
| Small for gestational age | | Infant with weight at birth less than the 10th percentile of WHO standard for term infants (WHO 2006) and a new fetal growth standard which will be released by the end of our trial (Villar 2010) | |
| IYCF indicators | | A set of 7 indicators published by WHO (WHO 2010 ) | |
| WASH indicators | | Structured observations of key behaviors, and hardware/environment spot-checks to assess WASH behavior uptake: latrine use (presence of fecal matter in the homestead, a well trodden path to the latrine), handwashing (presence of water in the tippy-tap), water treatment (test for residual chlorine in water) and presence of a protected play area | |
| Nutributter | | This is a peanut based spread that is fortified with micronutrients and other essential nutrients. See section 11 for complete formulation | |
| Play mat | | 3 X 2.8 polythene mat | |
| Play yard | | A colorful and durable plastic commercially available play yard product. The specific product being distributed is the NorthStates Superyard (Colorplay). | |
| Play space | | Play mat + Play yard. The two components are deigned to provide a protective play area for the child that provides protection from environmental contamination while allowing for exploratory play. | |
| Social support for mothering | | Assessed using questions adapted from published instruments (Cohen 1985; Sherbourne 1991) and pilot-tested in Shona, comprising material, informational, and emotional support. | |
| Maternal access/control of resources | | Questions adapted from Gates-Funded Alive & Thrive Project survey, and pilot-tested in Shona | |
| Maternal depression | | Assessed with the Edinburgh Postnatal Depression Scale, whereby 10 questions are each scored between 0-3. A score of 9-11 indicates mild to moderate depression; a score of 12 or above indicates major depression. | |
| Maternal stress | | Elevated salivary cortisol concentration | |
| Maternal roles, priorities and time | | Novel set of questions developed and pilot-tested in Shona by our team | |
| Maternal perceived physical health | | Assessed using an adaption of the SF-36 | |
| Mothering self-efficacy | | Assessed using questions adapted from published instruments (Dumka 1996; Gilmore 2009) and pilot-tested in Shona | |
| Fidelity of implementation (FOI) of interventions | | Extent to which study interventions are delivered as designed/intended. (Timely delivery/receipt of intervention messages and inputs; adherence to intervention module structure and content; quality of provider-client interaction/message delivery)  Assessed through review of supervision and VHW records to establish timing of VHW visits and adherence to implementation protocols. | |
| Village Health Worker capacity to deliver randomized interventions | | VHWs’ motivational and supervisory characteristics assessed using questions adapted from IFPRI-World Vision instruments, published instruments and our formative research. Knowledge acquisition and the extent of knowledge sharing with mothers (transfer of knowledge) assessed using knowledge tests administered to mothers and VHWs. | |
| Gross motor, fine motor, language and social development | | Assessed using the Malawi Developmental Assessment Tool (MDAT), which measures child development in 4 categories: 1) Motor coordination; 2) Fine motor coordination; 3) Language; 4) Social. | |
| Language development^1^ | | Assessed using the McArthur Bates CDI: a specific assessment of child language. The interview has three sections: 1) Vocabulary checklist; 2) Communicative gestures; and 3) Grammar checklist. | |
| Object permanence/cognition | | Assessed using the A not B test. This task requires the child to watch as a treat is hidden in one of two possible locations; after a brief delay, the infant is allowed to reach for the hidden treat. After two successful retrievals the object is hidden in the alternate location. The exercise is repeated ten times. | |
| Impulsivity | | Assessed using the Delayed inhibition test, in which the child is required to watch as a treat is promised to them, but they have to wait to take it. | |
|  | |  | |
| Maternal-child interaction | | Assessed using the Observation of Maternal Child Interaction (OMCI) in which the mother is given a picture book and asked to show her child the book and play with her child normally. The mother is observed and data recorded using a checklist including items on the mother’s interaction, mood, verbal statements, language stimulation, the infant’s mood, focus, communication and mutual enjoyment. | |
| Home environment and nurturing | | Assessed using the Family Care Indicator inventory, which assesses the home environment on child development. The questionnaire/inventory covers: objects children play with in the home; adult-child interactions; the number of books, magazines and newspapers in the home. | |
| School-aged cognitive function | | Assessed using the Kaufmann Assessment Battery for Children, 2^nd^ Edition (KABC-2) which measures general cognition, memory, spatial ability and reasoning. Note the following 8 KABC2 subtests will be in the main study, and are highlighted in bold below: Number Recall, Word Order, Rover, Triangles, Pattern Reasoning, Story Completion, Atlantis, Atlantis delayed   \| **Scale** \| **Subtest** \| **Description** \| \| --- \| --- \| --- \| \| Sequential Processing \| **Number Recall** \| Child repeats a series of numbers in the same sequence the examiner said them. \| \|  \| **Word Order** \| The child touches a series of silhouettes of common objects in the same order as the examiner said the names of the objects. \| \| Hand Movements \| The child copies the examiner’s precise sequence of taps on the table with the fist, palm or side of the hand. \| \| Simultaneous Processing \| Face Recognition \| The child is presented with a photograph of a human face for 5 seconds. A photograph is then presented with multiple individuals and the child is asked to point to the person with the face previously shown. \| \| **Rover** \| The child moves a toy dog to a bone on a checkerboard-like grid that contains obstacles (rocks and weeds) and tries to find the quickest path i.e. the one that takes the fewest moves. \| \| **Triangles** \| For most items, the child assembles several identical foam triangles (blue on one side, yellow on the other) to match a picture of an abstract design. For easier items, the child assembles a set of colourful plastic shapes to match a model either constructed by the examiner or shown on the easel. \| \| Block Counting \| The child counts the number of blocks in a picture of a stack of blocks, some of the blocks are partially hidden. \| \| Gestalt Closure \| The child mentally ‘fills the gaps’ in partially completed drawings and names or describes the object or action depicted by the drawing. *Items representing French fries, fire hydrant, windmill, electric cord, map of the USA, egg carton, and ice cream will be removed.* \| \| Planning \| Conceptual thinking \| The child is shown a series of four stimuli whereby one of them does not belong with the other three. The child identifies the item that does not belong. \| \|  \| **Pattern Reasoning** \| The child is shown a series of stimuli that form a logical, linear pattern, but one stimulus is missing. The child completes the pattern by selecting the correct stimulus from 4 to 6 options at the bottom of the page. \| \|  \| **Story completion** \| The child is shown a row of pictures that tell a story, but some of the pictures are missing. The child is given a set of pictures, selects only the ones that are needed to complete the story and places the missing pictures in their correct locations. This test may also be adapted with more culturally relevant stories. \| \| Learning and Delayed Recall \| **Atlantis** \| The examiner teaches the child nonsense names for fanciful pictures of fish, plants and shells. The child demonstrates learning by pointing to each picture when it is named. \| \|  \| **Atlantis delayed** \| The child demonstrates delayed recall of paired associations learned about 15-25 minutes earlier during Atlantis by pointing to the picture of the fish, plant or shell that is named by the examiner. \| \|  \| Rebus \| The examiner teaches the child the word or concept associated with each particular rebus (drawing) and the child then ’reads’ aloud phrases and sentences composed of these rebuses. \| \|  \| Rebus delayed \| The child demonstrates delayed recall of paired associations learned about 15-25 minutes earlier during Rebus by “reading” phrases and sentences composed of those same rebuses. \| \|  \|  \|  \|   NIH toolbox  The pilot will assess the suitability of the iPad based NIH toolbox tasks(Fernald, Prado et al. 2017), to see if this is feasible. This is not in the main study   \| Measure \| Test \| Description \| \| --- \| --- \| --- \| \| Attention & executive function \| Flanker Inhibitory Control and Attention \| The child has to select an object flanked by diversionary objects: These are either in the same direction as the target (*congruent* flankers), or in the opposite response (*incongruent* flankers), or to neither (*neutral* flankers). \| \| Episodic Memory \| Picture sequence memory test \| The child has to recall a series of illustrated objects that are presented in a particular order on the computer screen. \| \| Working Memory \| List sorting memory task \| The child is presented with a series of pictures. The child has to remember the order and reorder them from smallest to largest. \| \| Language \| Picture and oral reading recognition \| Firstly, the child selects the picture resembling a word. Secondly, the child is asked to read and pronounce letters and words. \| \| Executive function \| Dimensional Change card sort test \| The child sorts cards with 2 objects on them (e.g., red trucks and blue trucks) one way (e.g., by color) and then is instructed to switch and sort the same cards a new way (e.g., by shape). \| \| Processing speed \| Pattern comparison speed test \| The child identifies whether two visual patterns are the “same” or “not the same” (responses were made by pressing a “yes” or “no” button) in a short period of time. \|   PLUS EF Pilot  The pilot will assess the suitability of the android based PLUS- EF tool(Obradović, Sulik et al. 2018) for executive function tasks, to see if this is feasible. Note the success of this in the pilot means the PLUS-EF will be incorporated into the main study   \| Measure \| Test \| Description \| \| --- \| --- \| --- \| \| Executive function \| Hearts and Flowers \| The child is presented with an image of heart or flower on one side of the screen and is told to focus on either the heart or the flower. The child is asked to press the button either on the same (congruent test) or opposite side (incongruent test) as the selected object. \| \|  \| Multi-Source Interference Test \| The child is presented with a sequence of three digits. For each trial, two of these digits (the distractors) are the same and one (the target) differs from the distractors (e.g., “2 2 1”). The child is instructed to press a button for the numeric value of the target. For example, the correct response to the sequence “2 2 1” would be “1”. \| \|  \| Digit Span Backward \| A series of digits are presented sequentially on the tablet screen. The child is instructed to enter those numbers backwards onto a numeric keypad after the last digit was presented. \|   NEPSY Animal Card-sorting task  The card-sorting task also assesses executive function. Animal Sorting requires the child to sort cards into two groups of four cards each using various self-initiated sorting criteria. It assesses the child’s ability to formulate basic concepts, to transfer those concepts into action (i.e. sort into categories), and to shift from one concept to another within 6 minutes of total time. This is only in the pilot. | |
|  | |  | |
| School-aged literacy and language | | Assessed using the Early Grade Reading Assessment (EGRA). The EGRA gauges early literacy skills through a 15-minute individual oral assessment of five fundamental reading skills.  In addition, the Foundational Learning Module from the MICS 2019 survey will assess literacy using different tasks. Note for the main study, the literacy test has been simplified in reading. In addition, some writing tasks have been added based on the Word Reading Arithmetic Test (WRAT) | |
| School-aged numeracy | | Some questions from the Foundation Learning Module from the MICS 2019 survey will also assess basic numeracy, as well as from the early maths assessment and from the Word Reading Arithmetic Test (WRAT) for the main study . | |
| School-aged fine motor function | | Assessed using rapid continuous sequential movements (RCSM) of the hands. This task uses timed sequential finger tapping and timed hand pronation and supination, which was previously shown to be slower in children with stunting(Chang, Walker et al. 2010). This is also in the main study. | |
| School-aged growth and body composition | | Growth assessed by anthropometry including height, weight, head circumference, abdominal, upper arm, hip and calf circumferences. Body composition assessed by knee-heel length, skinfold thickness measurements and bioimpedance analysis (BIA). Knee-heel length measures from the knee to the bottom of the ankle as a sensitive measure of growth. Skinfold thickness measures the subcutaneous fat layer around the body. BIA measures the proportion of lean mass using an imperceptible electrical signal between the hand and foot. All these measurements are also in the main study. | |
| School-aged physical function | | Assessed by measuring handgrip strength, how far the children can jump in the broad jump and also how far and fast they can run in the beep test. During the beep test, their heart rate and blood pressure will also be measured and compared to resting values. All these measurements are also in the main study. | |
| School-aged overall child function | | Assessed using the UNICEF/Washington Group Child Functioning module, which highlights disability. This is also in the main study. | |
| School-aged socio-emotional development | | Assessed using the Child Behavior Checklist (CBCL) and Strength and Difficulties Questionnaire (SDQ). These are caregiver questionnaires that screen for socio-emotional symptoms in children by performing a detailed behavior assessment. For the main study, the SDQ will be used. | |
| Schooling and homework | | Assessed by a caregiver questionnaire including enrolment, and periods of absence, available books for the child and help with homework. Further questions will also assess the impact of COVID-19 precautions on school enrolment and schooling support for children at home. All these questions are also in the main study. | |
| School-aged adversities index | | Assessed by a caregiver questionnaire that asks for major life adversities associated with reduced child development since birth, including health. This includes the Child Parent Relationship scale (CPRS) which measures the caregiver’s view of their relationship with their child. All these questions are also in the main study. | |
| School-aged food and water insecurity | | Assessed using the Household Food Insecurity Access Scale (HFIAS) and Household Dietary diversity score (HDDS). Further food insecurity questions will be asked using the Holistic Food insecurity scale (H-FOOD) and Food Consumption score (FCS). For water insecurity, the Household Water Insecurity Experience Scale (HWISE) will be used and also the Holistic water insecurity scale (H-WATER). All these questions are also in the main study. | |
| School-aged water access | | Assessed by questionnaire on type of water point and time to walk for both drinking and household uses. All these questions are also in the main study. | |
| School-aged child discipline | | Assessed by a caregiver questionnaire that asks on use of discipline. This was previously used in 2019 across Zimbabwe in the UNICEF Multi-Indicator Cluster Survey (MICS6)(ZIMSTAT and UNICEF 2019). All these questions are also in the main study. | |
| School-aged illness | | Assessed by monthly screening questionnaire from village health workers, combined with standardized data questionnaire from data collectors. | |
| School-aged illness exposures | | Assessed by examining the handheld health card, previous clinic visits and hospital records (where appropriate) | |

^1^ The McArthur Bates test is an assessment of language skills, which requires several standardized steps to adapt it for use in a local language. Because there are a very small proportion of Ndebele-speaking families in the study area, the test will be adapted for use in Shona and not Ndebele. Instead, children from Ndebele speaking households will be assessed using the Malawi Developmental Assessment Tool.

### Health System Strengthening Prior to Randomized Trial

The following activities have been conducted under separate phases of this project as preparation for this randomized trial phase. These include:

1. Strengthening of the PMTCT program in the study area.
2. Strengthening of the Village Health Worker program of the Ministry of Health and Child Care.

#### Strengthened Pregnancy Surveillance

In addition to the above mentioned health systems strengthening activities, prior to beginning trial recruitment, Early Pregnancy Identification (EPregI) by Village Health Workers will be strengthened in the two study districts, in collaboration with the Ministry of Health and Child Care (MOHCC). Currently, MOHCC Village Health Workers are expected to visit every household in their catchment area every 3 months to identify pregnancies, document newly identified pregnancies in the pregnant women’s section of the Village Health Worker Register, and refer pregnant women to the nearest health facility for antenatal care by their 4^th^ month of gestation (16-24 weeks). Village Health Workers also provide health education to pregnant women and their partners, and discuss the role of men in maternal and newborn health.

However, reliable and timely identification of pregnancies by women and health workers is critical for optimal antenatal care coverage and early pregnancy identification is particularly essential for effective implementation of the 2010 PMTCT Guidance which requires drug initiation by 14 weeks gestation for HIV-positive women. Given the high HIV prevalence in this setting, the MOHCC has agreed to work in collaboration with our team to strengthen pregnancy surveillance by systematizing the record keeping by VHWs and by training them, as a routine part of their work, to enquire about a woman’s last menstrual period (LMP) and to offer urine dipstick pregnancy tests to mothers in the community who have missed a period. To assist women in recalling their LMP date, VHWs will provide all reproductive-aged women with a calendar, which can be used to record her menstruation dates. In addition, women will be given a generic information sheet which provides an overview of the SHINE trial. Women who have a positive pregnancy test will be given a referral slip to the health center, encouraged to book as soon as possible, and encouraged to be HIV-tested at the first ANC visit. For women who have a negative pregnancy test followed by a positive pregnancy test, or who can provide a reliable LMP date, the VHW will also request the woman’s assent be visited by a SHINE Data Collector (DC) to learn about SHINE, following the script on the Village Health Worker Weekly Form (**SHINE_Form1_v3_VHW Weekly Form and Script to Assent for Referral to SHINE**). If the mother assents, the Village Health Worker will tell the mother that he/she will return within a few weeks with a SHINE Data Collector. The SHINE field hub will receive an SMS from the VHW once s/he has identified a newly pregnant woman. S/he will include in her SMS, following the woman’s verbal assent, the date of her LMP so that gestational age can be calculated. Women identified as pregnant will be eligible to join SHINE. The SHINE Field Data Supervisor will schedule an appointment date for the VHW and a SHINE Data Collector (DC) to go together to the woman’s house. The VHW will leave **an Intervention-specific brochure (SHINE_16_v2 WASH Brochure; SHINE_17_v2 WASH/IYCF Brochure; SHINE_18_v2 IYCF Brochure; SHINE_19_v2 SOC Brochure)** with the woman, which provides further information about the SHINE intervention the mother would receive if she joined, based on the cluster where she lives. At the screening visit, the DC will explain the SHINE trial to the mother if she is eligible (ie confirmed pregnant by a urine pregnancy test)

**Antenatal Clinic Screening**

We will also screen women attending antenatal clinics to identify eligible women who may have been missed by E Preg I. A SHINE data collector will visit high volume ANC sites. After introduction by the Sister in Charge, the DC will ask the women attending for antenatal care whether they know about SHINE, provide them with the community brochure describing the trial, and complete the SHINE-Form 27_v1 Surveillance Form to identify eligible women who are interested in joining SHINE. For women who are interested in the trial, the DC will make an appointment with her for a consent visit at her home by calling the Field Data Supervisor at the SHINE hub.

The Field Data Supervisor will also contact the woman’s VHW and request the VHW to meet the Data Collector for the consent visit. Thereafter, consent into SHINE will proceed in the usual way as described above.

**Screening and referral for postnatal treatment of schistosomiasis**

In mid-August 2011, the findings of a national helminthiasis and schistosomiasis survey, which had been conducted at the district level in late 2010 by the Zimbabwean National Institutes of Health Research, were released. (Ministry of Health and Child Care of Zimbabwe, 2011). In brief, 10,222 school-age children were tested for soil transmitted helminths (hookworm+ ascaris+ trichiuris) and schistosomiasis [S. hematobium (urinary) and S. mansoni (intestinal)]. Prevalence for our study districts are presented in Table 4.

**Table 4: Prevalence of schistosomiasis and soil-transmitted heminths in school-age children in Shurugwi and Chirumanzu.**

| Study district | Schistosomiasis | Soil transmitted helminths |
| --- | --- | --- |
| Shurugwi | 55.9% | 1.5% |
| Chirumanzu | 23.5% | 0% |

Shurugwi had one of the highest prevalence of schistosomiasis in the country; nearly all of the infections were urinary (only 0.8% intestinal). Based on these survey findings, the WHO recommendations are annual and biannual mass drug administration for Shurugwi and Chirumanzu, respectively. The first mass drug administration is due to be delivered by MOHCC in June 2012, which will coincide with the start of SHINE. All members of the community will be treated presumptively, except for women who are identified as being pregnant based on a history of amennorhea. Although a WHO informal consultation (WHO, 2002) recommended that pregnant and lactating women should benefit from treatment with praziquantel as much as any other group, this guidance has not been adopted by MOHCC in Zimbabwe because of concerns regarding abortion following administration of praziquantel.

Reproductive aged women have been identified as a high-risk group by WHO, because of the morbidity caused by female genital schistosomiasis and anemia, together with the potential for adverse birth outcomes among infected women.

While schistosomiasis prevalence is likely to be lower in adults than primary school children, these survey findings suggest schistosomiasis may be both substantial and an important cause of anemia and immune activation in pregnant women enrolled in our trial. Since both maternal anemia and immune activation are likely to mediate adverse birth outcomes, maternal schistosomiasis is a potentially important factor in this current proposal. The majority of women recruited to the trial will have received praziquantel during the mass drug administration, therefore we anticipate the prevalence of schistosomiasis will be substantially reduced. However, treatment failure and re-infection occurs commonly in endemic areas. We will therefore screen all women recruited to the trial for schistosomiasis by urinary filtration and light microscopy for egg detection, to determine the prevalence of the infection among women and to enable us to adjust for schistosomiasis infection in our analyses. Any women found to be infected will be informed and advised to seek treatment from their local clinic after delivery.

**Screening for mycotoxin exposure among antenatal women**

Mycotoxins are fungal metabolites that can contaminate foods such as maize, wheat and groundnuts. Three mycotoxins that are particularly prevalent in food, and which have been linked to poor growth in humans and/or animals, are aflatoxin, fumonisin and deoxynivalenol (DON). Additionally, recent method development allows the assessment of other potentially important mycotoxins in urine including: T-2-toxin (T-2) (and its metabolites HT-2-toxin (HT-2), HT-2-toxin-4-glucuronide (HT-2-4-GlcA)), zearalenone (ZEA) (and its metabolites zearalanone (ZAN), α-zearalanol (α-ZEL), and β-zearalanol (β-ZEL) and corresponding 14-O-glucuronic acid conjugates (ZEA-14-GlcA, ZAN-14-GlcA, α/β–ZEL-14-GlcA)) and ochratoxin A (OTA and ochratoxin alpha (OTα)). One of the pathological mechanisms underlying mycotoxin exposure is intestinal damage, which is histologically similar to EED. Several studies have shown associations between maternal aflatoxin exposure and low birth weight, and between infant aflatoxin exposure and stunting in early childhood. Little is known about the impact of fumonisin or DON on adverse birth outcomes or stunting, but it is biologically plausible that these mycotoxins may mediate similar effects to aflatoxin. Assessment of T-2 toxin, zearalenone, and ochratoxin A will allow us to estimate the total mycotoxin exposure burden in SHINE participants. The prevalence of mycotoxin exposure in Shurugwi and Chirumanzu is not known, but since the staple foods in these districts include maize and ground nuts, there may be high levels of exposure among antenatal women and their infants. To determine the levels of exposure, all 4800 mothers will be screened for aflatoxin M1 at the baseline and 30-34 weeks gestation visits, and the 1600 infants enrolled in the EED substudy will be assessed for aflatoxin M1 at 3, 6, 12 and 18 mo and aflaotinx-lysine, fumonisin, DON ,T-2 toxin, zearalenone and ochratoxin A at 6, 12 and 18 mo of age. Mothers in the preterm birth/SGA substudy will be assessed for aflatoxin-lysine, fumonisin , DON, T-2 toxin, zearalenone and ochratoxin A.

Methods development for the aflatoxin-albumin ELISA and mass spectrometry to measure exposure in SHINE trial participants in Zimbabwe have been ongoing, but there is a global lack of standard materials (i.e. samples with known concentrations of aflatoxin-albumin). After 2 years of working on these methods, we have failed to develop a sufficiently sensitive analytical method with the equipment available in Zimbabwe and must ship samples for analysis to University of Maryland College Park, USA and/or University of Muenster, Germany which have the technical mass spectrometry capacity to measure aflatoxin-lysine. Aflatoxin-lysine will be extracted from maternal and infant plasma samples using solid-phase extraction columns in the Zvitambo laboratory, and the extracted aflatoxin-lysine analyte will be shipped to University of Maryland College Park, USA and/or University of Muenster, Germany to measure aflatoxin-lysine using mass spectrometry.

The equipment in Zimbabwe will subsequently not be suitable for measurement of urinary excretion of mycotoxins and their metabolites. Recently, a lab at the University of Muenster has developed a highly sensitive method to measure multiple mycotoxin exposure. Urine samples will be shipped to the University of Muenster, Germany to measure fumonisin, DON, T-2 toxin, zearalenone and ochratoxin A.

***Screening for PAH exposure in Pregnancy and Infancy***

Indoor air pollution for pregnant women and children may be high in rural Zimbabwe due to reliance on wood and other organic compounds for cooking and heating. Polycyclic Aromatic Hydrocarbons are released during wood burning and may be associated with inflammation, child growth and poor cognitive outcomes. Urinary PAH metabolites could be an effective biomonitoring approach for estimating exposure to indoor air pollution in young children. We propose to conduct a pilot study on 600 mother and infant urine samples to screen for PAH exposure using GC-MS. Dr. Olson’s lab at The University at Buffalo specializes in this assay. We propose to send 600 urine samples to The University at Buffalo for analysis.

### Study Procedures in Sequential Order for Women Referred to Study

#### Informed Consent Visit

The DC will ask the mother whether she has received an **intervention-arm specific brochure (ie, SHINE forms 16, 17, 18, or 19)** and give her one if she does not have it. The DC will review the brochure with the mother, allowing her to ask questions.

Note that due to the clustered nature of the randomization and that many of the interventions will be delivered through the Village Health Worker System, the consent form will be customized for women in each intervention cluster [(**See Consent Forms specific to each intervention arm: SHINE_Form 2_v10_SOC Consent Form; SHINE_Form 3_v10_IYCF Consent Form; SHINE_Form 4_v10_WASH Consent Form; SHINE_Form 5_v10_WASH+IYCF Consent Form**].

The DC will tell the woman that only women who are pregnant are eligible for enrollment into SHINE. Following the intervention-specific consent form, the DC will obtain the mother’s written permission to transcribe the date of her LMP from the Village Health Worker’s register, and to confirm her pregnancy with a second urine pregnancy test. The DC will also enquire about any episodes of vaginal bleeding since the VHW undertook a pregnancy test. If the pregnancy test is negative the nurse will provide counseling regarding causes of early pregnancy loss or amenorrhea not associated with pregnancy, and tell the mother that if she becomes pregnant again during the recruitment period of the trial, she would then be eligible for the trial. If the woman reports a recent episode of vaginal bleeding, the nurse will explain that this could be a sign of early pregnancy loss and that the pregnancy test can remain positive for several weeks after a miscarriage. The DC will not enroll the woman at that visit, but will refer her to the local clinic for assessment. She will reschedule an appointment 2 weeks later, when she will enquire about the medical assessment of the mother’s bleeding. If early pregnancy loss was confirmed by the clinic, she will be counseled but not enrolled into the trial; the DC will tell the mother that if she becomes pregnant again during the recruitment period of the trial, she would then be eligible for the trial. If early pregnancy loss was not confirmed by the clinic, she will be offered another pregnancy test. If the test is positive and she has had no further episodes of vaginal bleeding since her clinic appointment, and she is <24 gestational weeks by calculation from the LMP, the DC will describe the study following the intervention-specific consent form. If the mother wants more time to decide (i.e., wishes to discuss the study with other family members or consider it further) the DC will leave the intervention-specific consent form with the mother and make an appointment for a return visit. If the mother does not consent, the DC requests the mother’s assent to record her age, place of residence (Study cluster number), and reason for not joining the study on a Declining Participant Information Sheet **(SHINE_Form 6_v4_Declining Participant Information Sheet)**

If the mother consents to join the study, the DC will proceed to the obtain consent for specimen archival, using the **(SHINE_Form 9_v5_Specimen Storage and Shipment Consent Form).**The DC will then schedule a baseline visit with the mother and give her a labeled stool collection package and ask her to collect a sample of her stool on the morning of the baseline visit if possible.

At each postnatal research visit a small, non-monetary gift will be offered to the mother to thank her and her infant for their time in participating in data collection. The total value of all gifts over the course of the study will not exceed $10.

#### Baseline Enrollment Visit

On the appointed day, the DC will arrive at the mother’s house in the morning and proceed as follows:

- - If the mother has produced a stool sample that morning, the DC collects it and stores it in a cool box. If she has not provided a stool sample, the nurse will ask her to try to provide a stool sample before ingesting the lactulose-mannitol solution. If she is not able, the DC leaves the specimen container with the mother and will schedule a time to come and collect the sample, if feasible.
  - The DC asks the mother to provide a urine specimen before ingestion of lactulose-mannitol solution, and undertakes urinalysis using a Multistix test strip. She also stores a sample of this urine in the cool box for subsequent microscopy and storage.
  - The DC asks the mother to drink a solution of Lactulose and Mannitol (sugar water) and records the time of consumption on the Baseline Survey Form **(SHINE_13_v5_Baseline Survey Form).**
  - For 2 hours, the nurse collects and measures all maternal urine, storing the specimen in a pre-labeled bottle in the cool box.
  - During the 2-hour urine collection period, the nurse:
    - - Administers the Baseline Survey Form; see Research Data Collection section for details).
      - Weighs the mother and measures her height and mid-upper arm circumference (MUAC)
      - Weighs the mother’s youngest child <24 months of age and measures his/her recumbent length and MUAC
      - Addresses any questions about the study which the mother or a member of her household has
      - Collects maternal blood samples by venous puncture (5 ml in clot tube, 2 x 5 ml in EDTA tubes)
      - Tests the mother’s blood for HIV using a rapid test algorithm approved for use in Zimbabwe and provides appropriate counseling (referral for PMTCT care if positive, how to stay negative if negative). If the mother prefers not to have the HIV test in her home, the DC discusses the benefits of learning her HIV status during early pregnancy so that if she is positive she can receive medication to help prevent her baby from getting infected, and will strongly encourage her to be tested during her first visit to antenatal clinic. In all Zimbabwean public antenatal clinics, HIV testing is done routinely for all pregnant women at first booking (unless the woman specifically “opts out”) and HIV test results are recorded on the woman’s handheld antenatal card. For women who opt out of HIV testing, the DC will explain that HIV testing will be undertaken on her blood sample in the Zvitambo laboratory, because it is important for the trial to know her HIV status; however, she can choose not to receive the result if she prefers.
      - Tests the mother’s blood for hemoglobin using the Hemacue rapid test. If the mother’s hemoglobin is <10g/dL the DC will refer the mother for work-up and care at the local clinic. Regardless of the hemoglobin result, the DC will explain the advantages of iron and folic acid (IFA) supplementation during pregnancy and advise early booking for antenatal care to access IFA.
      - Measures the mother’s blood pressure
      - Collects saliva sample
      - Makes an appointment with the mother for the 32 weeks gestation follow up visit. The target timing of the 32 week gestation visit is 30-34 weeks; if the baseline visit occurs >30 weeks, the 32 week gestation visit will be omitted.

#### Intervention and Research Data Collection Activities Timeline

Following the baseline visit, two sets of activities will be initiated; 1) the interventions based on the randomization and 2) continued research data collection. The staff who implement the interventions are purposely separate from the staff who will conduct research data collection. All the education and behavior change components of the intervention modules will be implemented through the MOHCC Village Health Worker system with the support of SHINE Intervention Nurses (INs). The construction of latrines and installation of hand washing stations (Tippy Taps) in the WASH arms will be done through a collaboration between Zvitambo and the Ministry of Health and Child Care.

The table below illustrates how these two sets of activities are integrated temporally. In the following sections we specify the components of each of the behavior change modules referred to in Table 5 (all of which were developed and pilot tested during previous phases of this project following IRB approval) and the content of each data collection visit.

**Table 5 – Timetable of research and implementation visits in SHINE**

| **RESEARCH** | | | **IMPLEMENTATION** | | | | | | | | | |  |
| --- | --- | --- | --- | --- | --- | --- | --- | --- | --- | --- | --- | --- | --- |
| **Target date (optimal dates) [allowable dates]§** | **Data collection activity** | | **Timing of intervention visit**  **Intervention Implementation Activities**  Black – MOHCC intervention delivered by Village Health Worker  **Blue – Study behavior change intervention delivered by Village Health Worker**  Red – Study hardware intervention delivered by MOHCC/study staff  Green – Study consumable delivery by Village Health Worker | | | | | | | | | |  |
|  |  |  | **Target date* (optimal dates) [allowable dates]** | | **Standard Care** | | **IYCF** | | **WASH** | | **IYCF+WASH** | |  |
| 2-<24 weeks gestation | Village Health Worker referral to trial | |  | | Village Health Worker referral for ANC booking | | Village Health Worker referral for ANC booking | | Village Health Worker referral for ANC booking | | Village Health Worker referral for ANC booking | |  |
| 9 weeks (8-12 weeks) [8-wks gestation < parturition] | Informed consent and enrolment | |  | |  | |  | |  | |  | |  |
| 11 weeks (10-12 weeks) [10wks gestation<parturition] | Baseline visit **SHINE_Form 13_ v5 _Baseline form** | |  | |  | |  | |  | |  | |  |
|  |  | | <24 wks gestation (before delivery) | |  | |  | | - Latrine construction - 2 Tippy Taps delivered to household | | - Latrine construction - 2 Tippy Taps delivered to household | |  |
|  |  | | 25 wks gestation  (25-<29 wks)  [Before delivery] | | - **Module S1** | | - **Module S1** | | - **Module S1** - **Module W1** | | - **Module S1** - **Module W1** | |  |
|  |  | | 29 wks gestation  (29-<33 wks) | | - **Module S1:** | | - **Module S1** | | - **Module W2** - Give soap. - **Module S1** | | - **Module W2** - Give soap. - **Module S1** | |  |
| 32 wks gestation (30-34 weeks)  [30 weeks gestation<parturitionl] | Pregnancy Follow-up Visit **SHINE_Form 15_ v2 _32 weeks Gestation form** | | 33 wks gestation (33-37 wks)  [Before delivery] | | - **EBF Module** - **Module S1** | | - **EBF Module** - **Module S1** | | - **EBF Module** - Give soap - **Module S1** | | - **EBF Module** - Give soap - **Module S1** | |  |
| BIRTH  (0-1 week) [0-4 weeks] | Birth visit**  **SHINE Form**  25 _v1_Birth Visit_ | | BIRTH  Day 3 | | - **EBF Module** - **Module S2** | | - **EBF Module** - **Module S2** | | - **EBF Module** - **Module S2** | | - **EBF Module** - **Module S2** | |  |
| 5 weeks (4-6 weeks)  [4-12 weeks] | End of Pregnancy **SHINE_Form 14_v5 End of Pregnancy form**  / 1 month visit*** | | 3 weeks  (3-<8 weeks)  /1 month visit | | - **EBF Module** - **Module S3** | | - **EBF Module** - **Module S3** | | - **EBF Module** - **Module S3** - Give soap | | - **EBF Module** - **Module S3** - Give soap | |  |
|  |  | | 8 weeks  (8-<12 weeks)  /2 month visit | | - **Module S3** | | - **Module S3** | | - **Module W3** - **Module S3** - Give soap | | - **Module W3** - **Module S3** - Give soap | |  |
| 14 weeks (12-16 weeks)  [12-25 weeks] | 3 month visit  **SHINE_Form 20_v4_3mo visit** | | 12 weeks  (12-<16 weeks)  /3 month visit | | - **EBF Module** - **Module S3** | | - **EBF Module** - **Module S3** | | - **EBF Module** - **Module S3** - Give soap | | - **EBF Module** - **Module S3** - Give soap | |  |
|  |  | | 16 weeks  (16-<20 weeks)  /4 month visit | | - **Module S4** | | - **Module S4** | | - **Module W4** - Give Water Guard and soap - **Module S4** | | - **Module W4** - Give Water Guard and soap - **Module S4** | |  |
|  |  | | 21 weeks  (21-<25 weeks)  /5 month visit | | - **Module S4** | | - **Module N1** - **Module S4** | | - **Module W5** - Give Water Guard and soap - **Module S4** | | - **Module N1** - **Module W5** - Give Water Guard and soap - **Module S4** | |  |
|  |  | | 21 weeks  (21-<33 weeks) | |  | |  | | - Play yard delivered to household | | - Play yard delivered to household | |  |
| 27 weeks (25-29 weeks)  [25-51 weeks] | 6 month visit  **SHINE_Form 21_v3_6mo visit** | | 25 weeks  (25-<29 weeks)  /6 month visit | | - **Module S4** | | - **Module N2** - Give Nutributter - **Module S4** | | - Give Water Guard and soap - **Module S4** | | - **Module N2.***Infant’s first solid foods* - Give Nutributter - Give Water Guard and soap - **Module S4** | |  |
|  |  | | 29 weeks  (29-33 weeks)  /7 month visit | | - **Module S4** | | - **Module N3** - **Module S4** - Give Nutributter | | - **Module S4** - Give Water Guard and soap | | - **Module N3** - **Module S4** - Give Nutributter - Give Water Guard and soap | |  |
|  |  | | 34 weeks  (34-<38 weeks)  /8 month visit | | - **Module S3** | | - **Module N4** - **Module S3** - Give Nutributter | | - **Module S3** - Give Water Guard and soap | | - **Module N4** - **Module S3** - Give Nutributter - Give Water Guard and soap | |  |
|  |  | | 42 weeks  (42-46 weeks)  /10 month visit | | - **Module S3** | | - **Module N5** - **Module S3** - Give Nutributter | | - **Module S3** - Give Water Guard and soap | | - **Module N5** - **Module S3** - Give Nutributter - Give Water Guard and soap | |  |
|  |  | | 47 weeks  (47-<51 weeks)  /11 month visit | | - **Module S4** | | - **Module S4** - Give Nutributter | | - **Module W4** - **Module S4** - Give Water Guard and soap | | - **Module W4** - **Module S4** - Give Nutributter - Give Water Guard and soap | |  |
| 53 weeks (51-55 weeks)  [51-76 weeks] | 12 month visit  **SHINE_Form 22_v2_12mo visit** | | 51 weeks  (51-55 weeks)  /12 month visit | | Review S4 | | Review  Review S4   - Give Nutributter | | Review  Review S4   - Give Water Guard and soap | | Review  Review S4   - Give Nutributter - Give Water Guard and soap | |  |
|  |  | | 13-17 monthly visits | |  | | - Give Nutributter | | - Give Water Guard and soap | | - Give Nutributter - Give Water Guard and soap | |  |
| 78 weeks (76-80 weeks) [76-104 weeks] | 18 month visit  **SHINE_Form 23_v2_18mo visit** | | 77 weeks  (77-<81 weeks)  /18 month visit | | - **Module S3** | | - **Module S3** | | - **Module S3** | | - **Module S3** | |  |
|  |  | |  | |  | |  | |  | |  | |  |
| 104 weeks  (102-106 weeks) [102-112 weeks] | | 24 month visit  **SHINE_Form 63 v5_24 month visit form**. | |  | |  | |  | |  | |  | |

*Date stated is the target date. VHW visits should ideally be made within a week of the target date, but can be made within a 4-week window of this date, as shown in brackets. Missed VHW module delivery visits may be summarized during subsequent contacts. Timing of VHW visits will be monitored to establish the degree of intervention fidelity. The target for latrine construction and tippy tap distribution is <24 weeks gestation, but in all cases the WASH hardware must be in place before the baby is born. The target for play yard distribution is 21 weeks of child’s age, but in all cases the play yard must be at the household before the child is 33 weeks of age.

**Birth visit is for home deliveries only, to undertake anthropometry on the infant using SHINE_Form 25_v1_Birth Visit. In the case of institutional deliveries, anthropometry will be undertaken in the health facility and the birth home visit will not be made.

***The 1 month postnatal visit will usually coincide with the end of pregnancy visit. In cases of miscarriage, premature delivery, stillbirth, neonatal death or maternal death, an end of pregnancy visit will be scheduled as soon as feasible after the Hub is informed and in the event the baby was delivered at an institution, further birth information will be collected using SHINE_Form 55_v2_Birth Facility Information.

§ If the participant is away for a long period of time and is not able to be visited within the allowable window, then a decision may be made by the Hub to obtain data for that visit at a subsequent time-point to avoid missing data, using SHINE_Form 54v1 End of Pregnancy Catch Up Form and SHINE_ Form 56v1_Baseline Catch up Form for EOP and baseline data respectively.

#### Behavior Change Intervention Module Description

All behavior change interventions will be delivered by the Village Health Worker. The components of each module are identified in Table 6.

**Table 6 – Behavior change intervention modules**

| **Module Number** | **Components** |
| --- | --- |
| **W1** | **Stool Disposal**   - Emphasizes extent of fecal contamination (human and animal) in the household - Discuss the importance of maintaining a clean environment for baby’s health - Promoting consistent latrine use by all household members - Discuss importance of proper stool disposal in relation to baby’s health |
| **W2** | **Handwashing with soap (HWWS)**   - Initiate hand washing with soap discussion   - HWWS after handling fecal material   - HWWS before handling food   - HWWS of the infant’s hands - Emphasis on disrupting the transmission of germs to the infant - Discuss how to use the tippy tap |
| **W3** | **Protection of the Developing Child**   - Discuss what the ‘traffic of dirt’ mean in terms of the amount of fecal material in the soil - Using a Play mat and play yard to prevent children from eating soil or chicken feces |
| **W4** | **Water treatment**   - Discussion of the importance of water treatment - Discuss water treatment |
| **W5** | **Hygienic Handling Preparation and Feeding of Complementary Food**   - Discuss hygienic preparation of infant food,   - clean utensils,   - HWWS before serving food   - hygienic storage   - reheating of food that was safely stored |
| **EBF** | **Exclusive breastfeeding**   - All infants should be fed only breastmilk from birth to 6 months - This means no water, no cooking oil, no traditional medicines – nothing but breastmilk (emphasis on the management of fontanelle, colic) - EBF is important for all infants whether mother is HIV positive or HIV negative. |
| **N1** | **Nutrition for your baby**   - Emphasizes the importance of continuing to exclusively breastfeed infants from 5 to 6 months of age - Discuss the various roles that food helps the body perform: brain development, fight infection, health growth - Responsive Feeding |
| **N2** | **Your baby’s first solid foods**   - Introduction of solid foods: Nutributter and thick porridge - Why does the infant now need foods in addition to breast milk - How much food can be fed to the infant - Demonstrate how to prepare thick porridge - Demonstrate how to feed infant |

| **N3** | **Introducing more foods to your baby**   - Emphasize breast milk as the foundation of infant’s diet - Importance of continuing to feed 1 sachet of Nutributter each day - Importance of adding a variety of other foods to the infant’s diet - Demonstration of food processing - Review and support of responsive feeding |
| --- | --- |
| **N4** | **Maintenance of breastfeeding and feeding during illness**   - Discussion of what mother feeds infant during illness - Explanation of what infant needs during illness to recover - How to feed infant during illness - How to address diarrhea |
| **N5** | **Dietary Diversity**   - Importance of varying infant’s diet now that it is older - Discussion of 4 main food groups: porridge/sadza (carbohydrates), meat (protein), vegetable (vitamins/minerals), cooking oil/sugar (extra energy) - Discussion of foods mother prepares for the family - Review of responsive feeding |
| **S1** | **Encouraging Targeted ANC visit**   - Introduction to the antenatal care clinic - Discussion of what to expect at a visit - Importance of Iron Folate supplements - Importance of Tetanus Injection |
| **S2** | **Early Neonatal Home Visit**   - Introduces and promotes skin to skin contact - Promotes hygienic umbilical cord care - Provides support for low birth weight and sick newborns and infants born to HIV infected mothers |
| **S3** | **EPI Reminders**   - Emphasizes the importance of vaccinations - Encourages timely vaccinations according to the national EPI schedule |
| **S4** | **Family Planning**   - Introduces Family Planning - Timed discussion of various Family Planning methods |

**Qualitative study of mothers' experiences in receiving the SHINE
interventions.**

At least one year after the recruitment of mothers, we will purposively sample up to 80 mothers (20/arm) who have been enrolled in SHINE, to conduct an in-depth interview.
The purpose of the interview is to learn about what mothers think about the
interventions in SHINE and the performance of VHWs i.e. the different ways the VHWs deliver intervention messages.
This exercise may lead to improvements in the interventions or their delivery.  A SHINE researcher will visit the mother and obtain her consent for the interview using SHINE Form­­ 33 v1 Mother’s Intervention Delivery Perception Consent Form.   The interview (using SHINE Form 34v1 Mother’s Intervention Delivery Perception Questionnaire) will last up to 2 hours and will be audiotape recorded if the mother provides permission.  If information has been missed in the interview, the mother may have one follow-up to gather that information missed in the initial interview. All audiotapes will be destroyed after transcription.

**Qualitative study to assess the potential effectiveness of the SHINE WASH intervention**

It is important to understand not only the positive and intentional impact of the intervention but also to understand what unintended consequences might result. To elucidate this, and in

order to strengthen the delivery of the module on “Protection of the Developing Child” (W3), we will conduct structured interviews and observations we will conduct a developmental evaluation among a purposive sample of up to 80 women enrolled into the WASH and WASH + IYCF arms and 40 women enrolled in the IYCF and SOC arms of the study and with a baby between 5-18months. Structured interviews and observations will enable us to:

- Confirm the feasibility and acceptability of the play space in this context;
- Learn how play spaces are being used and where, the frequency and duration of their use, and their cleaning and storage; and
- Assess if the intervention is introducing unintended benefits or risks;
- Ascertain and address any safety concerns or misunderstandings;
- Assess if the interventions modify the care that babies receive; and
- Assess if mother’s perspectives about child care are influenced by the interventions they are receiving

Two rounds of data collection visits will be conducted: the first round will target women with babies 5-8 months and who would have recently received play yards, while the second round will target women with babies 12-18 months (at least 6 months after the delivery of the play space).

The first round of data collection will comprise two visits conducted among up to 40 women.

***Visit 1*:** 1-3 weeks after the delivery of the play yard SHINE research staff will obtain written informed consent using **SHINE Form­­ 36v1 developmental evaluation consent form.** The researcher will ask the mother questions related to, and make observations around the use of the SHINE play space components (play mat and play yard) using **SHINE Form 37 v1 play space developmental evaluation Visit 1.** Based on their observations, the researcher will also counsel the mother on safe use of the play space and potential problems and suggested solutions using SHINE form 38 v1 play space counselling guide. The visit will last up to 2 hours and the participant interviews will be audio/tape recorded if the mother provides permission. All audiotapes will be destroyed after transcription.

***Visit 2:*** 2 weeks after visit 1, SHINE research staff will conduct a follow-up visit to ask further questions and make observations around the continued use of the play space using **SHINE form 39 v1 play space developmental evaluation Visit 2**. The visit will last up to 2 hours and the participant interviews will be audio/tape recorded if the mother provides permission.

For the second round of data collection (to be done at least 6 months after the first round) we will select another 80 women (who may include round one participants) and visit them once. During this visit, SHINE research staff will obtain written informed consent using **SHINE Form­­ 36 v1 developmental evaluation consent form** (for new, non-round-one, participants) and collect questionnaire and observation data using **SHINE form 37 v1 play space developmental evaluation Visit 1**. The observation will last up to 6 hours and the interview up to an hour. The interview will be audio/tape recorded if the mother provides permission.

**Qualitative Study of Intervention Nurse evaluation of VHW performance**

In order to strengthen the ongoing supportive supervision of VHWs, we will conduct in-depth interviews among a purposive sample of up to 10 intervention nurses. The interviews will provide insight into the intervention nurses’ (IN) perspective of VHW performance and identify target areas to more effectively support VHWs in their SHINE activities. A SHINE researcher will visit the IN and obtain consent using **SHINE form 52v1 Intervention Nurse’s perception of VHW performance**. The interview will last up to 1 hour. All interviews will be audiotape recorded if the IN provides permission. All audiotapes will be destroyed after transcription.

**Qualitative study of mothers' experiences using the SHINE interventions.**

We will purposively sample up to 80 mothers (20/arm) who have been enrolled in SHINE, to conduct an in-depth interview. The purpose of the interview is to learn about mothers’ experiences using the SHINE interventions and get their insights on challenges and opportunities for using these interventions. This exercise will explore how the SHINE interventions fit into the existing child care environment and how they influenced the mother’s child care decisions. A SHINE researcher will visit the mother and obtain her consent for the interview using **SHINE_ Form 76­_ v1_ Mother’s Experience with Interventions Consent Form.**   Following consent the researcher will conduct an in-depth interview using **SHINE_ Form 77_v1_ Mother’s Experience with SHINE interventions Questionnaire**. The interview will last up to 2 hours and will be audiotape recorded if the mother provides permission.  If information has been missed in the interview, the mother may have one follow-up to gather that information missed in the initial interview. All audiotapes will be destroyed after transcription.

#### Research Data Collection

All data collection is conducted by DCs during household visits, except institutional birth data which is transcribed from health registers: a) for deliveries that occur in one of the 43 health institutions in the study area, birth data will be transcribed by the DC from the Ministry of Health and Child Care delivery register; b) for home deliveries, birth data will be collected by DC using **SHINE_Form 25_v1 Birth visit**. Where notification of home birth is delayed (>7days) birth data will be transcribed by the DC from the Village Health Worker register. Table 7 outlines the specific types of data collected at each study visit. All interview data will be collected electronically and all biospecimens will be transported to a field laboratory for initial processing before being sent from the field to the study laboratory in Harare.

**Table 7 – Data and specimen collection from antenatal mothers**

| ***Antenatal Period*** | | | |
| --- | --- | --- | --- |
|  | **Study Visit** | | |
| **All Enrolled Mothers** | **Baseline**  **SHINE_ Form 13v5_ Baseline form** | **32 weeks Gestation**  **SHINE_Form 15_v2_32 weeks Gestation** | **End of pregnancy**  **SHINE_Form 14_v5_ End of Pregnancy form** |
| Woman’s vital status | x | x | x |
| Household composition | x |  |  |
| Education, employment, child care | x |  |  |
| IYCF practices for youngest child | x |  |  |
| IYCF knowledge | x |  |  |
| WASH knowledge | x | x | x |
| Exposure to intervention messages | x | x |  |
| Use of health care | x |  |  |
| Anthropometry of youngest child | x |  |  |
| Woman's autonomy and social support | x |  |  |
| Woman's physical and mental health | x |  | x |
| Mothering self-efficacy, time allocation and stress | x |  |  |
| Household sanitation and hygiene reported practices and observations | x | x | x |
| Mother’s chores and employment, household economic status, income and aid | x |  |  |
| Tippy tap, soap, latrine, Water Guard knowledge and water chlorination test | x | x |  |
| Socioeconomic status, aid, assets | x |  |  |
| Post-harvest storage practices | x | x |  |
| Household food security and dietary diversity | x |  |  |
| Economic shocks | x |  |  |
| Maternal height | x |  |  |
| Maternal weight | x | x | x |
| Maternal mid-upper arm circumference | x | x | x |
| Maternal blood pressure | x | x | x |
| Mother’s relationship to VHW | x | x |  |
| **Pregnancy Assessment** |  |  |  |
| Antenatal care of pregnancy | x | x | x |
| Past medical history | x |  |  |
| 7 day maternal health recall | x | x | x |
| Maternal 3 month symptom history | x | x | x |
| Pregnancy exposures (medication, alcohol, smoking) | x | x | x |
| Mother’s reproductive health and previous pregnancy |  | x |  |
| Delivery details |  |  | x |
| PMTCT history^1^ |  |  | x |
| Maternal postnatal morbidity |  |  | x |
| Maternal clinic visit and hospitalization history |  |  | x |
| Infant birth details |  |  | x |
| Facility birth information |  |  | x |
| Infant 7 day symptom recall |  |  | x |
| Infant postnatal morbidity |  |  | x |
| Infant clinic visit and hospitalization history |  |  | x |
| Infant immunizations and vitamin A |  |  | x |
| Infant practices (fontanelle and cord care, malaria prevention) |  |  | x |
| Infant PMTCT (HIV-exposed infants only) |  |  | x |
| Miscarriage risk factors and post-miscarriage health^2^ |  |  | x |
| Stillbirth verbal autopsy^3^ |  |  | x |
| Infant verbal autopsy^4^ |  |  | x |
| Maternal verbal autopsy^5^ |  |  | x |
| **Maternal Biologic Specimen collection^a^::** |  |  |  |
| L:M 2-hour urine collection | x |  |  |
| Single sample urine collection | x | x |  |
| Blood sample (for serum, plasma, cell storage) | x | x |  |
| Saliva | x |  |  |
| Stool | x |  |  |
| Maternal HIV rapid test | x^6^ | x^6^ |  |
| Maternal CD4 count | x^7^ | x^7^ |  |
| Maternal Hemoglobin (Hemocue) | x |  |  |
| Maternal urinary microscopy for schistosomiasis | x | x |  |
| Maternal urinalysis | x | x |  |
|  |  |  |  |
| **Paternal data collection** |  |  |  |
| Paternal height^8^ | (x) | (x) | (x) |
|  |  |  |  |
| Infant date of birth |  |  | x |
| Infant gender |  |  | x |
| Infant vital status |  |  | x |
| Infant birth weight |  |  | x |
| Infant birth length |  |  | x |
| IYCF practices for index child |  |  | x |
| Anthropometry of index child |  |  | x |
| Neonatal examination |  |  | x |
| Neonatal temperature and respiratory rate |  |  | x |

^1^For HIV-positive mothers only

^2^Only in women who have miscarriage (fetal loss before 28 gestational weeks)

^3^Only in women who have stillbirth (delivery of a dead fetus >28 gestational weeks)

^4^Only in women whose infant dies after a live birth

^5^Only in cases of maternal death: consent for a verbal autopsy will be obtained from a close relative

^6^ Women will be offered rapid testing in their home at baseline and again at 32 weeks (or those who were negative at baseline). HIV rapid testing will also be repeated in the laboratory using the same sample, to confirm the result obtained during the home visit. Women who prefer not to be tested in their home will be counseled on the benefits of learning their status during pregnancy and encouraged to be tested in the antenatal clinic. Blood samples collected from women who prefer not to be tested at home will be tested for HIV in the laboratory, but these results will be used only during analysis after personal identifiers are stripped from the HIV result data to interpret primary outcomes by maternal HIV status. If an indeterminate result is obtained in the laboratory, or if the result is different to the result obtained in the woman’s home, women may be approached to provide a repeat blood sample so that definitive HIV status can be determined. If women request an HIV test at other study visits to learn their status, we will undertake a rapid test in the home where feasible.

^7^In HIV-positive women only

^8^Paternal height will be measured once during the course of the trial, whenever the father is available. He will be asked to provide consent to measure and record his height, using **SHINE_Form 40v2_Paternal Height Consent form**. This information will be used to calculate the mid-parental height, which is an important genetic determinant of growth potential for the infant.

^a^Where specimens are not collected at a planned visit, either because the visit was missed or because it was not possible for another reason, those specimens will be collected as soon as possible thereafter, which may be outside the window of the original visit.

The mother’s biospecimens will be used for the following assays:

Urine:

Lactulose:mannitol test: A lactulose and mannitol sugar solution will be ingested by women and urine collected for 2 hours at the 10-<26 weeks gestation baseline visit. A sample of urine will then be taken from the bottle, preservative added and the sample frozen for subsequent analysis of L:M ratio by mass spectrometry at Oregon Analytics, USA, and for long-term storage (see section 17).

Lactulose mannitol (L:M) solution will be made up in a clean bench-top hood in a dedicated preparation room at Zvitambo in Harare. Supplies will either be autoclaved prior to use (stir bars, spatula, beakers), or will be sterile (water, 50mL conical tubes).

|  | **20ml** | **200ml** | **1000ml** |
| --- | --- | --- | --- |
| Sterile water (mL) | 12.5 | 125 | 625 |
| Mannitol (g) | 1.0 | 10 | 50 |
| Lactulose solution (mL) [conc. = 10g/15mL] | 7.5 | 75 | 375 |

20ml aliquots of L:M solution will be produced, using mannitol powder USP (Spect Chemicals and Laboratory Products, Gardena, CA, USA), lactulose solution USP (10g/15ml; Coghlan Group, USA) and sterile water, according to Table 8, below.

**Table 8 – Preparation of lactulose-mannitol solution**

20ml aliquots will be labeled with lot number, production date and expiry date (with 3 month shelf life) and stored in a dedicated refrigerator in Harare at 2-8C. A 5mL aliquot from every

batch of L:M solution produced will be cryopreserved for any future QC testing.

A strict cold chain (2-8C) will be maintained while transporting aliquots of the L:M solution to the 4 field hubs, where they will be kept in a dedicated L:M refrigerator with 24hr back-up generator. A log of refrigerated aliquots will be maintained by the laboratory technician at each site. Once a home visit requiring L:M administration is scheduled, the laboratory technician will label a vial of L:M solution to be used with a barcoded label that contains the study participant ID and date of visit.

Research Nurse Supervisors will dispense the L:M solution on the morning of the home visit, by matching the study subject ID and date with the barcoded vial, and DCs will transport it to the mother’s house in a dedicated cold box with ice packs.

Upon arrival at the mother’s home, the DC asks the mother to drink the whole 20ml dose. For infants, the DC will weigh the child, calculate the dose to be given (2ml/kg to a maximum of 20ml), measure the correct dose and give the solution with an oral syringe. The dose given will be recorded on the study form for that visit.

Single urine collection: A fresh sample of urine will also be collected from the pregnant women at both baseline and 32 weeks visits for urinary filtration and microscopy to detect ova of *Schistosoma haematobium.* Cryopreserved urine collected at the baseline visit from all enrolled women will be used to measure aflatoxin M1, which provides a measure of aflatoxin exposure over the prior 24-48 hrs, and will provide representative data on seasonality. Urinary creatinine will also be measured on these same women to correct for urinary dilution. Cryopreserved urine from both visits will be used to measure fumonisin (FB1) deoxynivalenol (DON and DON-G), T-2-toxin (T-2) (and its metabolites HT-2-toxin (HT-2), HT-2-toxin-4-glucuronide (HT-2-4-GlcA)), zearalenone (ZEA) (and its metabolites zearalanone (ZAN), α-zearalanol (α-ZEL), and β-zearalanol (β-ZEL) and corresponding 14-O-glucuronic acid conjugates (ZEA-14-GlcA, ZAN-14-GlcA, α/β–ZEL-14-GlcA)) and ochratoxin A (OTA and ochratoxin alpha (OTα)), by LC/MS/MS in women enrolled in the preterm birth substudy . Cryopreserved urine from the baseline visit will be used to measure intestinal fatty acid binding protein (I-FABP), a marker of small intestinal damage, and results compared to plasma levels and L:M ratio. Cryopreserved urine from a subset of 600 mothers will be used to measure PAH metabolites which are indicators of wood smoke inhalation. These samples will be shipped to Dr. James Olson at University at Buffalo for measurement as the technology is not available in Zimbabwe.

Blood: (15 ml total)

**5ml** will be collected into two endotoxin-free EDTA tubes (10ml total). Plasma will be extracted and aliquots stored in endotoxin-free tubes at -80C for subsequent analysis of CRP, AGP, sCD14, lipopolysaccharide, I-FABP, ferritin, hepcidin, sTFR, aflatoxin-lysine and IGF-1. The buffy coat layer will be removed, red cells lysed and white cells fixed for storage at -80C. Subsequent staining of thawed PBMCs with monoclonal antibodies will be undertaken to determine levels of T cell activation, proliferation, senescence, differentiation and thymic output and monocyte immunophenotype by flow cytometry. Thawed PBMCs or stored saliva can also be used to extract DNA subsequently. All enrolled women will be asked for informed consent for DNA extraction from stored samples, using **SHINE_Form9v5Specimen Storage and Shipment Consent Form**. Women providing consent will be flagged in the database so that samples can be subsequently used for DNA extraction. Women who do not provide consent will be flagged in the database and aliquots labeled with different bar-codes so that samples are not subsequently used for DNA extraction, long-term storage or shipment. DNA would be used in future studies to investigate genetic determinants of infant heath and growth.

For long-term storage plans see section 17.

**5ml** will be collected in a serum gel clot tube. Serum will be extracted and aliquots stored at -80C. For long-term storage plans see section 17.

A few drops (approx. 0.2ml) of blood will be taken from the second EDTA tube and transferred to the Hemacue cuvette for immediate hemoglobin testing and onto the HIV rapid test strip for immediate HIV testing.

Saliva:

Saliva will be collected by placing an oral swab under the tongue for 1-2 minutes. The whole swab is placed into a storage tube and frozen for subsequent analysis of salivary cortisol and cytokines (as listed under ‘blood’ above) and FUT2 ELISA to evaluate maternal capacity to fucosylate human milk oligosaccharides, which is linked to infant growth (Charbonneau *et al*., 2016). Saliva could also be used as an alternative to PBMCs for DNA extraction in women who provide consent (see above). For long-term storage plans see section 17.

Stool:

Fresh stool will be collected into a container and 3 aliquots frozen for subsequent analysis of markers of enteric inflammation (fecal neopterin, alpha-1 antitrypsin, myeloperoxidase) and analysis of the intestinal microbiota. For microbiota studies, DNA and RNA will be extracted from approximately 220 mg of stool from each sample, using standard protocols (e.g., MoBio Laboratories; PowerFecal Microbiome RNA/DNA extraction kit). Paired-end libraries will be constructed using a non-commercial protocol for the Illumina HiSeq 2000 platform. RNA samples will be depleted of rRNA prior to sequencing. Sequencing will provide information about the total microbial diversity in the intestine (e.g., species richness and abundance) and the functions (e.g. resistance to pathogen colonization) carried out by the intestinal microbiota. In 150 mother-infant pairs, we will also define the metabolic activity (metabolomics) of the fecal microbiota at 32 weeks and 1 month post-partum for women (whose infants are in the EED substudy) and at 1, 3, 6, 12 and 18 months for infants, using an untargeted metabolomics approach by nuclear magnetic resonance spectroscopy.

In addition to the 150 mother-infant pairs already included in the EED sub-study (600 stool samples), further randomly sampled stool specimens will be used to address mechanistic questions. The original 150 mother-infant paired stool samples used for metagenome sequencing will provide data on maternal and early-life microbiota composition and function. In order to examine the associations between the maternal microbiota, maternal inflammation and infant birth and growth outcomes, metagenomics sequencing of additional stool samples from mothers will be performed. Data on the composition of the maternal microbiota will be used to examine whether certain microbial taxa or functional genes are associated with infant growth faltering or poor birth outcomes (e.g., stillbirth, neonatal death or small-for-gestational-age).

Currently, there are no high throughput sequencing centers in Zimbabwe (or elsewhere in sub-Saharan Africa), or facilities to conduct nuclear magnetic resonance spectroscopy therefore stool (or the microbial DNA/RNA contained within these samples) will be transferred to Canada (University of British Columbia and Michael Smith Genome Sciences Centre, Vancouver, British Columbia, Canada) for sequencing and untargeted metabolomics. Samples transferred to Canada for sequencing and metabolomics will be identifiable by the study ID only. Chain of custody of the samples will be assured by a Zvitambo scientist who will accompany the samples to the collaborating laboratory. Samples will only be used for the analyses described in this proposal and remnants of samples will be destroyed after this work has been completed. Bioinformatics and biostatistical analysis will be carried out in conjunction with SHINE co-investigators and technical and bioinformatics training for Zimbabwean scientists will be encouraged as part of the analysis process.

Bacterial culture of stool samples may be performed to isolate microorganisms of interest that are important to the intestinal microbiome based on preliminary sequencing data. Briefly, stool samples will be re-suspended in serial dilutions in sterile buffer and added to culture media in order to culture and isolate microorganisms of interest. Whole stool or selected microbial organisms derived from the stool that demonstrate potential to influence pathways related to growth faltering or EED, may be used in animal models to identify mechanisms for such an effect. Briefly, stool samples will first be filtered to remove debris and large particles. The resulting bacterial filtrate will be re-suspended in appropriate media and transplanted into germ-free mice via oral gavage. Animals will subsequently be examined for growth and markers of EED. This analysis will be performed in the gnotobiotic facility at the Life Sciences Institute in UBC.

We will specifically evaluate the role of biofilms in the gut microbiota. Biofilms (BFs) are complex, heterogeneous assemblages of microorganisms, typically adhered to a surface. Growth in single or multispecies BFs is a strategy that can be utilized by bacteria to survive periods of nutrient starvation. Furthermore, greater than 80% of invasive human tissue infections may involve bacterial BFs, and invasive BFs are increasingly being identified in human gut pathologies (e.g. inflammatory bowel disease and colorectal cancer). Glycogen synthesis is a key adaptation for bacterial survival and persistence in conditions of fluctuating nutrient availability and facilitates BF development in response to nutrient scarcity. Likewise, ppGpp is a well-established signaling molecule that regulates bacterial metabolism during nutrient insufficiency, plays a key role in regulating BF formation, and is an important adaptation for bacterial colonization of the gut.

We propose to determine: (i) whether a mixed human gut microbiota, either from mothers who previously demonstrated an increased capacity for biofilm formation, or not, produce intestinal biofilms in germ-free (GF) mice in response to a normal diet versus a nutrient-deficient diet (high in refined carbohydrates and low in proteins and lipids); (ii) whether the nutrient-deficient diet selects for a microbiome that is enriched for biofilm-forming species and biofilm-associated bacterial metabolic functions; and (iii) whether the nutrient-deficient diet increases gut permeability and impairs weight gain. We hypothesize that a nutrient-deficient diet will induce intestinal biofilm growth in mice colonized with human gut microbiota that have an increased capacity for biofilm formation.

We will utilize fecal specimens collected during pregnancy from 10 HIV-negative pregnant women enrolled in SHINE. 200mg feces aliquots from five women with detectable levels of glycogen synthase and ppGpp biosynthesis pathway, and aliquots from five women without, will be selected based on our prior analyses of maternal fecal metagenomes from SHINE, and will be utilized to create biofilm-associated (BF+) and non-biofilm (BF-) fecal slurries to inoculate GF mice, which will be randomly assigned to a standard mouse chow or a nutrient-deficient diet. Colonic biopsies and fecal specimens from mice will be collected for sequencing. Experiments will be conducted at the Johns Hopkins University Germ-free Mouse Core Facility.

For long-term storage plans see section 17.

**Maternal clinical assessment:**

DCs will follow SHINE clinical referral guidelines for any women with abnormal clinical signs or measurements. The DC will complete a SHINE clinical referral form and ask the mother to attend the clinic either routinely or urgently, according to the guidelines.

**End of pregnancy visit**

VHWs will report whether any pregnancies have ended (including miscarriage, stillbirth, live birth). In the event of a SHINE miscarriage or stillbirth, a Research Nurse Supervisor and DC will visit the mother together, within 2 weeks of the event being reported. The timing has been chosen to accommodate the sensitivity of this visit, which will include both a verbal autopsy regarding the fetal death, and completion of an Adverse Event form (see SAE section). For infants delivered at home the DC will conduct a visit to undertake anthropometry on the infant using **SHINE_Form 25_v1_Birth Visit**. For infants delivered in health institutions the DC will undertake the routine 1 month postnatal visit, using form **SHINE_Form 14_v5_End of Pregnancy form**. Subsequently visits will be conducted at 3 months postnatal using form **SHINE_Form 20_v4_ 3 month visit**, at 6 months postnatal using form **SHINE_Form 21_v3_ 6 months visit**, at 12 months postnatal using form **SHINE_Form 22_v2_12 months visit**, and 18 months postnatal using **SHINE_Form 23_v2_18 months visit.**

Table 9 specifies the components of each post-partum research data collection visit.

**Table 9 – Postnatal data and specimen collection schedule**

|  |  |  | **Study Visit** | | | | | |  |
| --- | --- | --- | --- | --- | --- | --- | --- | --- | --- |
| **All mother-infant pairs** |  |  | **Months Post-Partum** | | | | | |  |
|  | Birth^1^ | 1 | 3 | 6 |  | 12 |  | 18 | 24 |
| Identification and Vital status of infant and mother | x | x | X | X |  | x |  | x | x |
| IYCF practice for index child |  | x | X | X |  | x |  | x |  |
| Nutributter knowledge and use (IYCF arm only) |  |  |  |  |  | x |  | x |  |
| Household sanitation and hygiene reported practices and observations |  | x | X | X |  | x |  | x |  |
| Tippy tap, soap, latrine, Water Guard Knowledge (WASH only) |  | x | x | X |  | x |  | x |  |
| Water chlorination test (WASH only) |  | x | X | X |  | X |  | x |  |
| Play Mat Use (WASH only) |  |  |  | X |  | x |  | x |  |
| IYCF and WASH Knowledge |  |  | X | X |  | x |  | x |  |
| Exposure to intervention messages |  |  |  | X |  | x |  | x |  |
| Immunizations and child health |  | x | X | X |  | x |  | x |  |
| PMTCT and infant HIV diagnosis |  | X | X | X |  | X |  | X |  |
| 7 day maternal morbidity recall |  | x | X | X |  | X |  | X |  |
| Maternal clinic visit and hospitalization |  | x | X | X |  | X |  | x |  |
| Infant 7 day morbidity history recall |  | x | X | X |  | x |  | x |  |
| Infant long-term illness recall |  | X | X | X |  | X |  | X |  |
| Infant clinical examination |  | x | X | x |  | x |  | X |  |
| Infant weight, length, head circumference and MUAC | x | X | X | X |  | X |  | X | x |
| Assessment of early child development |  |  |  |  |  |  |  |  | x |
| Infant body composition, skinfold thicknesses, mid-thigh circumference and leg length |  |  |  |  |  |  |  |  | X |
| Mother’s relationship to VHW |  |  | X |  |  | X |  | X |  |
| Woman's autonomy, agency, social support |  |  |  | X |  |  |  | x |  |
| Woman's physical and mental health |  |  |  | X |  |  |  | x |  |
| Woman mental health (Edinburgh postnatal depression scale) |  | x |  | X |  | X |  |  | x |
| Woman's mothering self-efficacy, time, and stress |  |  |  | X |  |  |  | x |  |
| Maternal weight and MUAC |  | x | x | x |  | x |  | x |  |
| Mother’s chores and employment; household socioeconomic status, income, and assets |  |  |  |  |  |  |  | x |  |
| Post-harvest storage practices |  |  |  | x |  |  |  | x |  |
| Household composition |  |  |  | x |  |  |  | x |  |
| Education, employment, child care |  |  |  | X |  |  |  | x |  |
| Market access and use of information |  |  |  | X |  |  |  | x |  |
| Household food security and diversity |  |  |  | x |  |  |  | x |  |
| Economic shocks |  |  |  | x |  |  |  | x |  |
| **Specimen Collection** |  |  |  |  |  |  |  |  |  |
| Infant blood sample^2^ |  |  |  |  |  |  |  | X^6^ | x |
| Woman’s blood sample^3^ |  |  |  |  |  |  |  | X^6^ |  |
| Paternal height^4^ |  | x | x | x |  | x |  | x |  |
| Infant saliva^5^ |  |  |  |  |  |  |  |  | x |

^1^For home deliveries only

^2^A 5.4 ml blood sample will be collected on all infants at 18 and 24 months by venipuncture, into two 2.7ml endotoxin-free EDTA tubes, using local anaesthetic cream for the blood draw if the mother wishes. A few drops of blood will be used to check hemoglobin using a Hemocue machine. If a mother is found to have become HIV-positive at the 18 month visit (ie seroconverted during the study), or already known to be HIV- positive, the infant blood will be used to test for HIV by DNA PCR and to measure CD4 count, to identify potential vertical transmission of HIV; the result of the infant HIV test will be returned to the mother by home visit. Whole blood will be stimulated with pathogen-associated molecular patterns in plates and incubated for 4-24hr. Supernatant will be removed and stored at -80C for subsequent analysis of pro- and anti-inflammatory cytokines, and cells will be fixed and stored for subsequent analysis of cellular activation, proliferation and cytokine elaboration by flow

cytometry, to gain insights into functionality of the immune system. Remaining blood samples will be spun to collect plasma, and the cell pellet will be frozen. Blood will be stored according to the section on long-term storage plans and will be used for subsequent analyses of indicators of the EE pathway, as evaluated in the EED sub-study below, and to undertake hepatitis A serology, as an indicator of feco-oral exposure. The final choice of assays will be determined by the results of this sub-study. Consent will also be sought from mothers using **SHINE_Form9v5_ Storage and Shipment Consent Form** to subsequently extract DNA from the stored cell pellet for use in future studies to investigate genetic determinants of infant health and growth. Infants for whom consent is not given will be labeled with a separate bar-code and flagged in the database so that samples are not subsequently used for DNA extraction, long-term storage or shipment. Note that if an infant is in the EED substudy, they should not have this blood sample taken, but instead should follow the schedule in Table 10.

^3^A 10 ml blood sample will be collected on all women at 18 months by venipuncture. HIV-negative women will be offered re-testing for HIV infection. We anticipate 0.8% HIV incidence per year among baseline HIV-negative women. Detection of HIV-infected women will enable timely referral for treatment, testing of infants who may be infected through breastfeeding for rapid referral to treatment, and reallocation of seroconverting women to the HIV-positive trial strata. As at previous testing opportunities, women who do not wish to learn their HIV status will be offered the option to have the HIV test conducted in the Zvitambo laboratory and not to receive the test result. Blood will also be stored according to the section on long-term storage plans and will potentially be used for subsequent analyses of indicators of the EED pathway. Woman who test HIV-positive at 18 months postnatal will have a CD4 count measured using the PIMA machine to assess their disease severity.

^4^Paternal height will be measured once during the course of the trial, whenever the father is available. He will be asked to provide consent to measure and record his height, using **SHINE_Form 40v2_Paternal Height Consent form**. This information will be used to calculate the mid-parental height, which is an important genetic determinant of growth potential for the infant.

^5^ A saliva sample will be collected from the child (at 24 months using a soft swab; samples will be stored at -80C for subsequent measurement of salivary cortisol.

^6^For 18 month visits that are conducted out of the study districts, where it will not be possible to process blood samples in the laboratory on the same day, a dried blood spot card will be collected and stored from infants instead of an EDTA blood sample. Mothers will be offered rapid HIV testing in the home but will not have an EDTA/serum blood sample collected.

^a^ Where specimens are not collected at a planned visit, either because the visit was missed or because it was not possible for another reason, those specimens will be collected as soon as possible thereafter, which may be outside the window of the original visit.

^b^Anthropometry data and hemoglobin result at 18 months (primary trial endpoints) will be checked carefully by Research Nurse Supervisors at the SHINE hub; in cases where primary endpoints are either missing or outside the stipulated plausibility ranges, a home visit will be conducted to repeat the measurement(s).

**Infant Observation**

In addition to the other data collection visits outlined above, we will conduct observation studies once on 180 mother-infant pairs during the course of the trial. These households will be selected by matching the gender and age of the youngest 90 infants in the WASH arms to 90 infants in the non-WASH arms. The purpose of these visits is to check compliance with WASH and IYCF behaviours, and therefore provide an additional in-depth measure of intervention uptake. A trained observer will conduct a 6-hour structured observation. The researcher will visit the mother and obtain consent using **SHINE_ Form 78_v1_Observation Study Consent Form**. After obtaining consent the researcher will observe the household as well as the normal daily routines of the mother using **SHINE Form 79_v1_Household and Baby Observation Form** to record specific baby activities and household observations.

**18 month visit**

An information sheet (**SHINE_Form 68v1_Specimen and Testing non EED Information Sheet**) which explains the reasons for specimen collection and haemoglobin measurement at 18 months will also be given to all participants, and, women in the SOC and IYCF arms will also be provided with a SHINE Pledge Certificate v1 which pledges that a latrine will be built at the end of the trial

In addition, informed consent will be sought to conduct an additional visit at 24mo of age using **SHINE_Form 64 _v1 _Neurodevelopment at 24 Months Visit Consent Form.** The procedures for obtaining informed consent will follow those used for the main trial (i.e. if participants require time to consider and consult, the consent form will be left with them and a return visit scheduled for a later date).

**Assessment of Early Child Development**

In the post-2015 era of the Sustainable Development Goals, there is a need to focus with renewed emphasis on whether children are thriving and whether their full human potential is being harnessed. This includes understanding whether they are developing well. The brain has the highest metabolic requirements of any organ, and stunting is closely associated with delayed developmental progression and later school outcomes. The precise links between stunting, “thrival” and neurodevelopmental outcomes have not been well explained. Assessing child development allows us to evaluate more broadly whether SHINE interventions help children to ‘thrive’, beyond measures of illness and linear growth.

The caregivers of children who reach 24 months of age from February 2016 onwards (estimated 3200 total) will be invited to have an additional home visit to assess early child development (ECD). We will undertake an assessment of general child development in the areas of gross motor, fine motor, language and social behavior skills, and will also include more specific tests that would enable us to pick up more subtle changes in child cognitive function, emotional regulation and temperament (Table 3).

In order to develop and validate appropriate tools to broadly assess ECD in rural Zimbabwe, we will undertake the following series of steps to pilot test the procedures in the study context:

**Step 1**: we will individually interview 25-30 mothers of non-SHINE children age 18 to 30 months seeking information concerning the words and grammatical markers (such as pre-fixes) their children use using **SHINE_Form 61_v1_MacArthur Bates Pilot 18-30 months Data Collection Form**.

We will seek written informed consent from caregivers at local clinics to undertake the interview (which will last 30-60 minutes), using **SHINE_Form 62_v1 _ MacArthur Bates Language Development Pilot 18-30 months Consent Form.** From these interviews, we will develop a list of at least 400 words for piloting, as well as an appropriate number of grammatical markers.

**Step 2**: We will pilot test the list of words, grammatical markers and/or word combining items, plus words that span a range from easy to more difficult, with a second cohort of 100 mothers of non-SHINE children age 18-30 months. We will also ask these mothers if their children say any words (or pre-fixes) that are not on the list. We will seek consent from caregivers at local clinics to undertake the interview (which will last 30-60 minutes), using **SHINE_Form 62_v1  MacArthur Bates Language Development Pilot 18-30 months Consent Form.**

**Step 3**: We will pilot test all the SHINE early child development tests with up to 20 children, using **SHINE_Form 63 v4_24 month visit data collection form**. These 20 children will be drawn from the SHINE cohort, but their scores will not be used in the final dataset and these children will not subsequently be included in the 24 month evaluations. Caregivers of these children will be approached to provide consent using **SHINE_Form 64 _v1 Neurodevelopment at 24 months Pilot Consent Form.**

Following these piloting steps, translated forms for the ECD tests being used will be finalized by amending **SHINE_Form 63 v4_24 month visit data collection form**. Periodically, and particularly after initial training, the administration of tests by Data Collectors will be video recorded so that an independent assessor can validate the assessments, thereby facilitating their standardization assessments. Permission for video recording will be sought from caregivers using **SHINE_ Form 66_v1  Video Neurodevelopment at 24 months Consent Form**. These permissions will include optional consent for the storage of video recordings for teaching and presentation purposes; otherwise, recordings will be destroyed after review and analysis as specified in the consent form.

The 24 month visit will be conducted by DCs using **SHINE_Form 63 v5_24 month visit data collection form**. Consent will be sought using **SHINE_Form 65 _v3 Neurodevelopment at 24 months Consent Form.** In the case of interviewing a guardian instead of a biological mother, **SHINE_Form 72_ v1__Guardian Neurodevelopment at 24 months visit Consent** will be used; this will be in situations where the mother has died or moved away and the primary caregiver is an extended family member. Consent for Shipment and Storage of samples will be sought using **SHINE_FORM 73_v1_Specimen Storage and Shipment at 24 months visit Consent**. In the case of a guardian instead of a biological mother consent will be sought using **SHINE_Form 74_v1_Guardian_Specimen Storage and Shipment at 24 months visit Consent**. The visit will be conducted among infants who have moved from their primary SHINE home but are still available to be visited within the study districts.

Caregivers of children identified as having developmental delay will be provided with some written information using SHINE_Form 67 v 1 Parent Information sheet about how to stimulate the child to encourage developmental potential. This information will be provided in training by Dr. Melissa Gladstone (trainer) to all DCs and will be based on the UNICEF/WHO Care for Child Development intervention programme (http://www.unicef.org/earlychildhood/index_68195.html). Children with substantial developmental delay (defined as delay in more than 3 items in two areas of development on the Malawi Developmental Assessment Tool) or obvious disability (as identified in the child functioning and disability module) will be referred to local health facilities using a SHINE clinical referral form. We will work with the district therapists during the piloting stages to develop appropriate referral pathways.

**Body composition at 24 month visit**

At the 24 month visit, we will also more fully evaluate the impact of the randomized trial interventions on body composition, in a subgroup of children turning 24 months from September 2016. We will undertake bioelectrical impedance vector analysis, using a BodyStat 1500MDD machine, which uses an undetectable current measured through electrodes attached to the limbs to assess lean and fat mass. We will measure skinfold thicknesses using Holtain calipers to assess subcutaneous fat, mid-calf circumference and will measure tibial length using a handheld kneemometer, which non-invasively measures the distance from knee to ankle and provides a better measure of long-term metabolic health associated with stunting. Consent will be obtained for these additional measurements using **SHINE _Form 70_v2 Neurodevelopment and Body Composition at 24 months visit Consent Form** and data collected using **SHINE _Form 71_v3 Body Composition Data Collection Form.**

***Pilot study to develop the IYCF-Plus Intervention***

The SHINE IYCF intervention provided 20 g small-quantity lipid nutrient supplement (SQ-LNS) and 5 core counselling modules promoting:

- SQ-LNS daily;
- Thick rather than thin porridge;
- Preparation of vegetables, fruits, meat, egg, insects, and legumes so that infants can masticate and swallow them;
- Responsive feeding during illness and convalescence;
- Diverse diet.

Overall, infants in the IYCF arm had significantly higher intake of macro- and micronutrients compared to the non-IYCF arm. However, the proportion of infants meeting their dietary requirements for kilocalories, zinc and folate remained low, indicating that the IYCF intervention was insufficient to close the nutrient gap.

We will pilot test an IYCF-plus intervention in a group of mother-infant pairs, which will include all of the original SHINE IYCF intervention plus three nutrient-dense foods: dehydrated egg, ground moringa leaf, and finely ground sugar bean legume. Counselling modules will promote adding these foods to children’s diets in age-specific daily amounts as shown in the table below. Achieving adequate energy intake assumes daily minimal mealie meal consumption in the amounts shown in the table.

| **Infant Age Group (mo)** | **Mealie Meal** | **Nutributter** | **Egg** | **Moringa leaf, dried and ground** | **Sugar bean legume, finely ground** |
| --- | --- | --- | --- | --- | --- |
| **6-8** | ≥ 13 g (1.5 Tbsp) | 20 g | 14 g (3 tsp) | 5 g (1 tsp) | 5 g (1 tsp) |
| **9-11** | ≥ 55 g (6 Tbsp) | 20 g | 14 g (3 tsp) | 10 g (2 tsp) | 10 g (2 tsp) |
| **12-24** | ≥ 128 g (14 Tbsp) | 20 g | 14 g (3 tsp) | 15 g (3 tsp) | 15 g (3 tsp) |

To develop and test the new IYCF-plus counselling modules, three qualitative studies will be conducted in a group of mother-infant pairs:

1. **Focus group recipe trials** among two groups of mothers of young children (6-11 months, and 12 -18 months) (total number = 12 mother infant dyads) to develop ways to prepare the three foods for complementary feeding.
2. **Trial of Improved Practices** (TIPS) with two groups of young children (6-11 months, and 12-18 months) and their mothers (total number =12) to:
   1. test and modify the recipes developed during recipe trials with different mothers and children to determine acceptability
   2. determine whether the target quantity of each of the three new foods will likely be consumed in one meal or in one day.
3. **Pilot test newly developed counselling modules** informed by the recipe trials and TIPS exercises.
4. **Focus group recipe trials**

Recipe trials will be conducted with two groups, each comprised of 6 mothers and their children (total, 12 mothers and children). Group I will be six mothers of children aged 6-11 months; Group II will be six mothers of children aged 12-18 months.

Community-based enrolment will be carried out by asking Village Health Workers (VHWS) who were in the IYCF arm during SHINE and therefore have particular IYCF expertise to identify women in their community who have children in each age group. VHWs will ask these mothers to come to a central location (e.g., a clinic) at a specified day/time. Once assembled, mothers will be told about the recipe trial and asked to provide written informed consent to participate using **SHINE_Form 86v1_Recipe TIPS Trial.** Following informed consent, a group discussion will be held, facilitated by a nutritionist. The discussion will be led using **SHINE_Form _88v1_Recipe Trial Focus Group Discussion Guide.** The goal of each recipe trial focus group will be, for each of the three new foods, to produce at least two ways of preparing it which is acceptable to mothers and infants.

1. **Trial of Improved Practices**

TIPS will be conducted with two groups each comprised of 6 mothers (total, 12 mothers and children). Group I will be six mothers of children aged 6-11 months; Group II will be six mothers of children aged 12-18 months. These will be mother-infant dyads who did not participate in the Focus Group Recipe Trials.

Community-based enrolment will be carried out by asking Village Health Workers (VHWS) who where in the IYCF arm during SHINE and therefore have particular IYCF expertise to identify women in their community who have children in each age group. VHWs will ask these mothers to come to a central location (e.g., a clinic) at a specified day/time. Once assembled, mothers will be told about the study and offered informed consent using **SHINE_Form 86v1_Recipe TIPS Trial.** Following consent, preparation of each of the two new recipes will be demonstrated for each new food (2 recipes for 3 foods, 6 demonstrations). Printed copies of each recipe will be given to the mothers. After the demonstrations, mothers will be asked to prepare one or more of the new recipes and feed the food to her child. The quantity of the new recipe consumed by the child will be recorded. Reactions from mothers and their children will be discussed and recorded. If a child rejects a new food, the recipe will be modified and offered to the child again. For example, if a child refuses porridge with moringa, porridge with a reduced amount of moringa will be prepared and offered.

Mothers will be given enough of the three foods to prepare and feed the new recipes during the following week and feed the foods to their children. One week later, the mothers will be assembled again to find out whether the mother tried the new recipes, whether the child liked the food and what quantity the child consumed. Problem recipes will be modified, prepared again, and tasted by mothers and infants. Mothers will be asked to make recommendations on which recipes they think are easiest to prepare and best accepted by their children.

1. **Counselling materials** will be prepared to teach mothers how to prepare all successful recipes for each food. These materials will include colourful photos and pamphlets. All 24 mothers who participated in the Focus Groups Recipe Trials and the TIPS will be invited to come to a central place again. The materials will be shown to the mothers. Feedback and discussion will be recorded, for subsequent transcription and analysis, to ensure that all feedback is captured; audio recordings will then be destroyed. Materials will be modified as needed based on feedback.

**School-aged SHINE follow-up visit**

A recent Lancet systematic review showed early-life nutrition interventions have benefits for neurodevelopment, but growth itself is not a reliable proxy for functional outcomes (Prado, Larson et al. 2019). The review emphasized the need for long-term follow-up of optimized complementary feeding (Ocansey, Adu-Afarwuah et al. 2019), and for WASH intervention studies that measure both growth and development. There are several reasons why further evaluations at school-age are important. Firstly, longitudinal evidence suggests that early-life interventions can alter the *trajectory* of brain and behavioral development, and differences between groups may therefore only become apparent at later ages (Martorell 2017). Secondly, neurodevelopmental evaluations of children at 2 years of age are restricted by short attention spans and the degree of engagement, and tools are therefore limited; it becomes easier at school age to undertake more sensitive and specific measures of cognitive development. Thirdly, certain domains of brain function only begin to develop from 2 years of age, including expressive language and higher cognitive functions such as socio-emotional behavior. To evaluate the effects of improved nutrition on all aspects of neurodevelopment therefore requires assessment at older ages. For example, follow-up of a similar LNS intervention delivered between 6-18 months in Ghana showed an effect on socio-emotional development at 4-6 years of age (Ocansey, Adu-Afarwuah et al. 2019), which would not be measurable at 2 years. Fourthly, neurodevelopmental scores at 2 years of age only modestly correlate with later performance. This relationship becomes stronger with age, such that school-age assessments are much more predictive of adult IQ and cognitive function. Finally, school performance is a valuable outcome that can only be evaluated at older ages.

We will pilot an additional visit to assess school-aged growth, physical and cognitive function. We will undertake an assessment of:

i) child cognitive function using the Kaufmann ABC2 (see Table 3), literacy using the Early Grade Reading Asssessment (EGRA), the MICS foundational learning module (FLM)(ZIMSTAT and UNICEF 2019), numeracy using the MICS FLM and fine motor function using rapid continuous sequential movements (RCSM) of the hands; (in addition we will pilot the feasibility of using tablet-based measures using the NIH toolbox and PLUS EF tools (see Table 3). We shall also pilot the feasibility of assessing executive function using the NEPSY animal sorting test.

ii) physical function by measuring handgrip strength, broad jump and shuttle run, including the rated perceived OMNI exertion(Utter, Robertson et al. 2002) and breathlessness(McGrath, Pianosi et al. 2005) scales to ensure appropriate effort is documented(Lelijveld, Seal et al. 2016), plus measurement of haemoglobin by Hemocue;

iii) quality of growth using both anthropometry and body composition techniques that combine bioimpedance analysis, skinfold thicknesses and leg length;

iv) child health and development, behavior, emotional regulation, homework, child discipline(ZIMSTAT and UNICEF 2019) and schooling, by caregiver questionnaire. We will also update data on food insecurity, water insecurity, socioeconomic status, water access, adversities and maternal depression (Table 3).

In order to develop and validate appropriate tools to broadly assess school-aged function in rural Zimbabwe, we will undertake the following series of steps to pilot test the procedures in the study context:

**Pilot**: We will pilot the tests and assessments in up to 80 non-SHINE children, who are aged 7 to 8 years and living in rural Shurugwi, together with their primary caregivers. We will sensitize the households of pilot study participants with the **SHINE_Form_93_v1_Community_sensitisation form.** We will seek written informed consent from caregivers at their household, local clinic or school to undertake the pilot assessment (which will last 2-3 hours), using **SHINE_Form_89_v2_ 7yr_Pilot_Caregiver_Consent.** For the child, we will also seek assent using **SHINE_Form 90_v2_7yr_Pilot_Child_Assent.** Within the consent and assent forms, we will ask additional permission to photograph the tests being conducted for training purposes. We will also ask permission to video-record the neurodevelopmental tests for internal quality assurance. This is to achieve standardized neurodevelopmental screening such as in the QualiND model (Ruisenor-Escudero, Familiar et al. 2019). To monitor the success of the tests being piloted, we will use **SHINE_Form_91_v2_7_yr_Pilot_Caregiver_Questionnaire** and **SHINE_Form_92_v2_7yr_Pilot_Child_Data_Collection**. The tests are shown and defined in **Table 3.**

Children with substantial developmental delay or obvious difficulties with hearing or vision (as identified in the child functioning and disability module) will be referred to local health facilities using a SHINE Clinical Referral Form**.** We will work with the district therapists during the piloting stages to ensure appropriate referral pathways are being maintained. Caregivers identified with depression on the Edinburgh Postnatal Depression Scale will be referred to clinic for further support.

**SHINE 7 year Follow-up visit:** We will perform an additional visit to assess school-aged health, growth, physical and cognitive function among 1300 children enrolled in SHINE. All outcomes are defined in Table 3, using the tools previously piloted to assess school-aged growth, physical and cognitive function. Among the 1300 children in the long-term follow-up visit, an estimated 1000 will be HIV-unexposed children (500 IYCF vs 500 non-IYCF; and 500 WASH vs 500 non-WASH, capitalizing on the factorial design) and 300 will be HIV-exposed. Children will i) still be living in rural Shurugwi; ii) aged 7-8 years at the time of enrolment; and iii) have a primary caregiver who is willing to provide written informed consent.

Cognitive function will be measured using the Kaufmann ABC2, fine motor, the SDQ, PLUS-EF and a short numeracy and literacy assessment. Physical function by handgrip strength, broad jump and shuttle run, including heart rate monitoring and blood pressure measurement at rest and after exertion. Quality of growth will include anthropometry, bioimpedance analysis, skinfold thickness and leg length. Child health, development, schooling and home circumstances will be updated using the same caregiver questionnaire as the pilot with addition of questions on gender norms, maternal social support and HIV medication (where relevant).

We will sensitize the households of study participants with the **SHINE_Form_98_v1_Community_sensitisation form.** We will seek written informed consent from caregivers to undertake the assessment (which will last 4 hours), using **SHINE_Form_94_v1_ 7yr _Caregiver_Consent.** For the child, we will also seek assent using **SHINE_Form 95_v1_7yr_Child_Assent.** We will also ask permission to video-record the neurodevelopmental tests for internal quality assurance. This is to achieve standardized neurodevelopmental screening such as in the QualiND model (Ruisenor-Escudero, Familiar et al. 2019).

Repeat HIV testing will be offered to all mothers who were HIV negative or HIV-unknown in SHINE, using the rapid test algorithm employed in the original trial, with appropriate pre-test and post-test counselling. HIV-positive and HIV-unknown mothers will be offered the opportunity to test their child, using a rapid test algorithm in the home, or by taking a dried blood spot sample to the laboratory for testing. Age-appropriate pre- and post-test counselling will be provided by trained nurses.

We will collect data using **SHINE_Form_96_v2_7_yr_Caregiver_Questionnaire** and **SHINE_Form_97_v2_7yr_Child_Data_Collection** forms. For HIV positive mothers, an additional questionnaire will confirm details from pregnancy, early life and any subsequent children using the **SHINE_Form_100_v1_7yr_early_life_questionnaire.**

Children with substantial developmental delay or major difficulties with hearing or vision (as identified in the child functioning and disability module) will be referred to local health facilities**.** Caregivers identified with depression on the Edinburgh Postnatal Depression Scale will be referred to clinic for further support.

***Illness monitoring***

We will undertake prospective health surveillance of the cohort of 1300 children, through monthly VHW visits to households to collect data on acute illness, clinic visits and hospitalization, by caregiver recall and review of handheld cards. Data will be collected by the VHW using **SHINE_Form_99_v2_7yr_VHW_monitoring_**form**.**

***Annual assessment***

We will develop a shorter version of the long-term follow-up assessment using factor analysis of the 7-year data to develop a tool that efficiently measures growth, and a tool that efficiently measures cognition. We will offer families an annual visit between the ages of 8 and 12 years to collect longitudinal data on child outcomes during mid-childhood. A small gift (e.g. reading book) will be given to the child at the end of each annual visit to thank them for their time in the assessment.

**Environmental Enteric Dysfunction (EED) Sub-study**

Selection: All women who enrolled in the trial from 1^st^ May 2014 onwards will be invited to join the EED substudy at the 32 week gestation visit, or as soon as possible thereafter,. All dyads where the mother is HIV-positive (approximately 600) will be invited to join to the study.

Where a mother has refused to learn her HIV test result, she will not be eligible for the EED sub-study.

Informed Consent for EED Sub-study:

At the 32 week visit, or as soon as possible thereafter, the DC will discuss the substudy with all women, and provide them with the EED Sub-Study Consent Forms **(SHINE_Form7_v8_EED Sub-study Consent Form; SHINE_Form8_v8_EED Sub-study Consent Form)**. Two consent forms are available – one for HIV-negative mothers and one for HIV-positive mothers. The only difference in procedures is that blood samples collected from HIV-exposed infants will be tested for HIV by DNA PCR for diagnostic purposes so that HIV-positive infants who become infected can be rapidly referred for treatment. NOTE: Zimbabwean government health facilities routinely test HIV-exposed infants at 4-6 weeks and again 1 month after breastfeeding ceases, so infants enrolled in the trial will receive more testing and referral services than available through the public sector health services.

Mothers who feel ready to consent to the EED substudy after reading the consent form can provide their consent at the 32 week visit; women who want more time to discuss the substudy will be given the consent form to keep and will be asked again for their consent at the 1 month postnatal visit. Women who missed the 32 week visit and who are approached to enroll in the EED substudy at a subsequent visit can provide consent at that visit if they feel ready to, or else a follow-up EED consent visit will be made once the mother has had time to consider the substudy. From mother-infant pairs enrolled in the EED Substudy detailed morbidity data will be collected. To assist in collecting these data, EED Substudy mothers will be given a cell phone and solar charger, with airtime topped up at each research visit, as described in the consent forms **(SHINE_Form7_v8_EED Sub-study Consent Form; SHINE_Form8_v8_EED Sub-study Consent Form)**.

Any HIV-positive mothers who gave birth prior to the launch of the EED substudy will be approached to give consent for the EED substudy at subsequent visits (ie beyond 32 weeks gestation) to ensure that all HIV-exposed infants are enrolled and final HIV status ascertained. This is important both for stratification of infants for the primary outcome, and to ensure that any HIV-infected infants are identified early and access immediate ART.

Data Collection for EED Sub-study: The schedule for additional data collection beyond that for the main trial is listed in Table 10. The EED substudy ends at the 18 month (primary endpoint) visit. No EED samples are collected at the 24 month visit. EED infants at the 24 month visit have data and specimens collected as outlined previously in Table 10. Mothers will be provided with an information sheet **(SHINE_Form 69_v1_Specimen and Testing EED Information Sheet**) which explains the reasons for specimen collection and haemoglobin testing at each visit.

**Table 10 – Data and sample collection from EED substudy participants**

| **Additional data for EED Sub-study Infants**  *[N=1000 non-HIV exposed (250/grp);*  *N~600 HIV-exposed)]* | **Antenatal** | **Postnatal** | | | | |
| --- | --- | --- | --- | --- | --- | --- |
|  | **30-34 weeks** | **1** | **3** | **6** | **12** | **18^1^** |
| Informed Consent for EED Sub-study | x | (x)^1^ | (x)^2^ | (x)^2^ | (x)^2^ | (x)^2^ |
| Intensive 24 hour dietary recall (50 infants per arm only) |  |  |  |  | x |  |
| Infant pre-LM single urine sample |  |  | x | x | x | x |
| Infant Lactulose:Mannitol test (2 hour urine collection) |  |  | x | x | x | x |
| Infant stool collection |  | x | x | x | x | x |
| Infant blood draw (2.5 ml in Paxgene tube, 2.7 ml in EDTA tube)^3^ |  | x | x | x | x | x |
| Infant saliva collection |  |  | x | x | x | x |
| Infant rapid hemoglobin test |  |  | x | x | x | x |
| Infant HIV testing by DNA PCR^4^ |  | x | x | x | x | x |
| Infant CD4 count^5^ |  | x | x | x | x | x |
| Infant morbidity diary (continuous) |  |  |  |  |  |  |
| Infant diarrhoeal stool collection (continuous, during diarrhoeal illness only) |  |  |  |  |  |  |
| Maternal blood sample |  | x |  |  |  |  |
| Maternal Stool sample |  | x |  |  |  |  |

^a^When an infant is in the EED substudy, specimen collection at 18 months should follow this table, and not the specimen collection schedule for infants in the main trial (Table 9)

^1^For 18 month visits that are conducted out of the study districts, where it will not be possible to process samples in the laboratory on the same day, a dried blood spot card will be collected and stored from infants instead of an EDTA blood sample. Pre-LM and LM urine, stool and saliva will not be collected from infants on long-distance visits.

^2^ If not consented at the 32 week gestation visit

^3^Local anaesthetic cream can be used prior to venipuncture if the mother wishes.

^4^Infants born to HIV-positive mothers only; a dried blood spot will be made in the field laboratory and transported to the Harare laboratory for PCR. Results will be returned to the mother by home visit. . If the sample result is indeterminate, we will conduct a home visit to give the result to the mother and request a repeat infant blood sample to clarify his/her HIV status. The repeat sample will be sent to the Harare laboratory for HIV DNA PCR and the result returned to the mother by home visit.

^5^HIV-exposed infants only

Where specimens are not collected at a planned visit, either because the visit was missed or because it was not possible for another reason, those specimens will be collected as soon as possible thereafter, which may be outside the window of the original visit.

Infant morbidity diary

Mothers of infants in the EED Sub-study will be asked to keep a daily morbidity diary (SHINE Form_26v2) using a simple calendar. For each day, the mother will be asked to place a sticker with pictures on the calendar of the symptoms experienced by the infant on that day . A laminated booklet with pictures explaining each symptom below will be left with the mother:

**Table 11: Morbidity diary for infants in EED substudy**

| **Symptom** | **Sunday** | **Monday** | **Tuesday** | **Wednesday** | **Thursday** | **Friday** | **Saturday** |
| --- | --- | --- | --- | --- | --- | --- | --- |
| Diarrhoea (3+ loose water stools per day) |  |  |  |  |  |  |  |
| Blood or mucus in stool |  |  |  |  |  |  |  |
| Hot body |  |  |  |  |  |  |  |
| Cough |  |  |  |  |  |  |  |
| Difficult or fast breathing |  |  |  |  |  |  |  |
| Inability to feed or lethargy |  |  |  |  |  |  |  |
| Took child for care |  |  |  |  |  |  |  |

To assist the mother in learning to complete the form, a SHINE field worker may make home visits to the mother during the first few months, to collect the completed diary, give a new one for the subsequent period, and encourage and thank the mother for her effort in keeping this diary. Women with adequate cellphone coverage may be supported by reminder SMS and/or phone calls to collect morbidity data instead of home visits.

To determine which supportive method (SMS, phone call or home visit) is most effective in collection of morbidity data, the first 40 women enrolled to the EED substudy will be part of a pilot evaluation: 10 women (Group 1) will be given a morbidity diary and will receive supportive home visits 2 weeks and 6 weeks after being given the diary; 10 women (Group 2) will not receive a diary but will be contacted once per week by phone to collect morbidity data and visited once at 6 weeks; 10 women (Group 3) will be given a morbidity diary and additionally receive a weekly phone call to collect morbidity data and will be visited once at 6 weeks; and 10 women (Group 4) will be given a morbidity diary and will receive supportive home visits 2 weeks and 6 weeks after being given the diary plus a weekly SMS reminder to complete the diary. At the 6 week visit, women in all groups will be asked to evaluate their experience in recalling morbidity data using **SHINE_Form_ 44v1 _Morbidity Diary Pilot Evaluation Form.**

Diarrhoea specimen collection

Women in the EED substudy enrolling from June 1st 2015 onwards will be asked to use the cellphone provided to call the SHINE hub whenever their infant develops diarrhoea (defined as >3 loose watery stools per day) or blood/mucus in the infant stools so that a Data Collector can make a home visit to collect a stool specimen. The DC will assess the infant during the home visit for dehydration and recommend a clinic visit if necessary. Stool samples will be frozen at -80C for subsequent analysis of enteric pathogens by PCR using a TaqMan array card assay at the University of Virginia, USA (Liu *et al.,* 2014), since the appropriate PCR platform is not available in Zimbabwe. Consent for this sample collection will be obtained at the 3 month postnatal visit, or as soon as possible thereafter, using **SHINE_Form 50v1 Diarrhoea Sample Collection Consent**, and when diarrhoeal specimens are collected, **SHINE_Form 51v3 Diarrhoea Sub-study Visit** will be completed. Mothers in the diarrhea substudy will receive a phone call every 2 weeks to remind them to collect a specimen if the infant develops diarrhea. Mothers will also be provided with **SHINE_Form 75_v1_Diarrhea Sample Collection Information Sheet** to provide further information about the diarrhea substudy.

Intensive dietary recall

The mothers of 50 randomly selected infants per arm (total sample size = 200) in the EED substudy will be asked a set of intensive dietary recall questions covering the prior 24 hours at the 12mo postnatal visit, using **SHINE Form 43v2 24Hr Dietary Recall Form**.

Following consent **(SHINE_Form10_v5_Specimen Storage and Shipment EED Infant Consent Form)** infant EED Sub-study biospecimens will be used for the following assays:

Urine:

Infant lactulose:mannitol test: A pre-L:M urine sample will be collected from the baby, then lactulose and mannitol sugar solution will be ingested by infants and post L:M urine collected for 2 hours. A sample of urine will then be taken from the bottle, preservative added and the sample frozen for subsequent analysis of L:M ratio by mass spectrometry at Oregon Analytics, USA and for long-term storage (see section 17). Cryopreserved urine will be used to measure intestinal fatty acid binding protein (I-FABP), a marker of small intestinal damage, and results compared to plasma levels. Urine from 6,12 and 18 mo time-points in 1600 infants enrolled in the EED substudy will be used to measure fumonisin, DON, T-2-toxin (T-2) (and its metabolites HT-2-toxin (HT-2), HT-2-toxin-4-glucuronide (HT-2-4-GlcA)), zearalenone (ZEA) (and its metabolites zearalanone (ZAN), α-zearalanol (α-ZEL), and β-zearalanol (β-ZEL) and corresponding 14-O-glucuronic acid conjugates (ZEA-14-GlcA, ZAN-14-GlcA, α/β–ZEL-14-GlcA)) and ochratoxin A (OTA and ochratoxin alpha (OTα)), by LC/MS/MS. In a group of 1000 infants (500 with ‘good growth’, defined as LAZ>-0.5 at 18 months; and 500 with ‘poor growth’, defined as LAZ<-2 at 18 months) we will send urine samples from the 12 month visit to Dr Jon Swann at Imperial College London to undertake untargeted metabolomics by NMR and/or mass spectrometry, which are not available in Zimbabwe. Metabolomics is a powerful technology for assessing metabolic pathways without a priori hypotheses, which will provide unique insights into the pathogenesis of stunting beyond the EED pathway that we are already testing. Cryopreserved urine from a subset of 600 infants at all timepoints will be used to measure PAH metabolites which are indicators of wood smoke inhalation. These samples will be shipped to Dr. James Olson at University at Buffalo for measurement as the technology is not available in Zimbabwe.

Blood (5.2ml total at each visit)

2.7 ml will be collected at each time point into an endotoxin-free EDTA tube. Plasma will be extracted and aliquots stored at -80C for subsequent analysis of CRP, sCD14, ferritin, hepcidin, sTFR, IGF-1, anti-rotavirus IgA,and, aflatoxin B1-lysine Plasma will be sent to Imperial College London to measure citrulline, kynurenine:tryptophan ratio (plus metabolites along the tryptophan/IDO1 pathway) and essential amino acids by mass spectrometry. The buffy coat layer will be removed, red cells lysed and white cells fixed for storage at -80C. Subsequent staining of thawed PBMCs with monoclonal antibodies will be undertaken to determine levels of T cell activation, proliferation, senescence, differentiation and thymic output, and monocyte immunophenotype by flow cytometry.

In a subgroup of 150 HIV-exposed and 150 HIV-unexposed infants and their mothers, the systemic inflammatory milieu will be characterized using multiplex plasma cytokine/chemokine analysis and measurement of soluble IL-6, lipopolysaccharide binding protein (LBP) and CD163 by ELISA using plasma collected at baseline for mothers and at 1mo or 3mo for their infants. This will allow us to explore the association between environmental enteric dysfunction, endotoxaemia, immune activation, HIV exposure and clinical outcomes of children.

We will undertake plasma HIV viral loads (RNA PCR) on all HIV-infected mothers with available samples at the baseline visit and the 32-week visit. This will allow us to explore the relationship between maternal HIV viraemia and outcomes of HIV-exposed children.

We will undertake plasma quantitative cytomegalovirus (CMV) DNA PCR on all HIV-infected and HIV-uninfected mothers with available samples at baseline and 32 weeks. This will allow us to determine the prevalence of CMV viremia during pregnancy; explore the associations between maternal CMV and clinical outcomes of HIV-exposed and HIV-unexposed children (survival, growth and neurodevelopment); and test the impact of the WASH intervention on reducing prevalence of maternal CMV at 32 gestational weeks.

Timing of CMV acquisition will be evaluated in HIV-exposed and HIV-unexposed children who had plasma, saliva or urine samples taken to determine age at first CMV detection. CMV DNA PCR will be undertaken on all available samples (from 1 to 18 months of age). This will allow us to explore the association between maternal CMV during pregnancy and timing of infant CMV acquisition; explore the associations between timing of infant CMV acquisition and clinical outcomes of HIV-exposed and HIV-unexposed children (survival, growth and neurodevelopment); and test the impact of the WASH intervention on delaying timing of CMV acquisition amongst HEU and HIV-unexposed children.

2.5ml will be collected at each time point into a PAXgene Blood RNA tube. This tube contains a preservative that stabilizes RNA and allows subsequent analysis of gene expression profiles, to explore mechanistic pathways underlying stunting in a non-biased way. Tubes are stored at -80C until extraction of RNA. For long-term storage plans see section 17.

At the one month visit, DCs will encourage HIV-positive mothers to have their babies tested for HIV at 6 weeks through the early infant diagnosis (EID) service of the government PMTCT program and to initiate cotrimoxazole prophylaxis. In addition, the SHINE laboratory will test all HIV-exposed infants enrolled in the trial at 1, 3, 6, 12, 18 and 24 by HIV DNA PCR on dried blood spot to ensure early diagnosis of HIV among breastfeeding infants. Any positive HIV DNA PCR results will be reported back to the family by the DC within 4 weeks, and referral to the local clinic made to access treatment. Thus, infants participating in the trial will benefit from more testing and referral opportunities than routinely available through public sector health services.

Saliva:

Saliva will be collected by placing an oral swab under the tongue for 1-2 minutes. The whole swab is placed into a storage tube and frozen for subsequent analysis of salivary cortisol and FUT2 (“secretor status”) which is important in modulating the response to epithelial injury and is a determinant of intrinsic susceptibility or resistance to certain enteric viral infections, such as norovirus and rotavirus; maternal-infant secretor status therefore an important potential effect modifier in SHINE. CMV DNA PCR will also be measured in HIV-exposed infants as outlined above.For long-term storage plans see section 17.

Stool:

An aliquot of fresh stool will be collected into a container and frozen for subsequent analysis of markers of enteric inflammation and repair (fecal neopterin, alpha-1 antitrypsin, myeloperoxidase and REG1), enteropathogen carriage by Taqman Array Card (conducted at the University of Virginia) and intestinal microbiota (as for maternal samples; see page 55 for details). We will investigate how the intestinal microbiota responds to: the WASH intervention and to the introduction of Nutributer (the intervention); EED severity; the presence of enteric pathogens (by combining data with the TaqMan Array Card); antimicrobial or other treatments; HIV and ART in the subset of children born to HIV-infected mothers; oral Rotavirus vaccine; breast-feeding practices and maternal inflammation; and how all of these may influence infant birth outcomes, growth and development.

Bacterial culture of stool samples will be performed to isolate microorganisms of interest that are important to the intestinal microbiome based on preliminary sequencing data. Briefly, stool samples will be re-suspended in serial dilutions in sterile buffer and added to culture media in order to culture and isolate microorganisms of interest. Whole stool or selected microbial organisms derived from the stool that demonstrate the potential to influence pathways related to growth faltering or environmental enteric dysfunction (EED), may be used in animal models to identify mechanisms for such an effect. Briefly, stool samples will first be filtered to remove debris and large particles. The resulting bacterial filtrate will be re-suspended in appropriate media and transplanted into germ-free mice via oral gavage. Animals will subsequently be examined for growth and markers of EED. This analysis will be performed in the gnotobiotic facility at the Life Sciences Institute in UBC.

In 150 mother-infant pairs, we will define the metabolic activity (metabolomics) of the fecal microbiota at 1, 3, 6, 12 and 18 months (as for maternal samples; see page 55 for details). The original 150 mother-infant paired stool samples used for metagenome sequencing will also provide data on maternal and early-life microbiota composition and function. In order to further examine the associations between the infant microbiota and breast-feeding practices additional stool samples from infants (1 month and 3 months) will be utilized for metagenomic sequencing. These data will allow us to examine whether differences in breast-feeding practices are associated with altered gut microbiota and whether this is associated with growth faltering. In addition, stool samples from infants (1 month and 3 months) will be utilized for metagenomic sequencing to examine whether the gut microbiota is associated with seroconversion of oral rotavirus vaccine for which data are also being collected.

Currently, there are no high throughput sequencing centers in Zimbabwe (or elsewhere in sub-Saharan Africa), or facilities to conduct nuclear magnetic resonance or mass spectroscopy therefore stool (or the microbial DNA/RNA contained within these samples) will be transferred to Canada (University of British Columbia and Michael Smith Genome Sciences Centre, Vancouver, British Columbia, Canada) for sequencing and untargeted metabolomics. Microbial metabolites from the stool may also be shared with the Metabolomics Innovation Centre in Edmonton, Alberta, Canada for testing services only.  Zvitambo scientists will be directly involved in this research and will be credited with authorship on publications arising from this work. Samples transferred to Canada for sequencing and metabolomics will be identifiable by the study ID only. Chain of custody of the samples will be assured by a Zvitambo scientist who will accompany the samples to the collaborating laboratory. Samples will only be used for the analyses described in this proposal and remnants of samples will be destroyed after this work has been completed. Bioinformatics and biostatistical analysis will be carried out in conjunction with SHINE co-investigators and technical and bioinformatics training for Zimbabwean scientists will be encouraged as part of the analysis process.

In a sub-group of 200 SHINE mother – infant pairs, microbial DNA from stool specimens will be sequenced at a deeper level in the laboratory of Dr Nicola Segata in Trento, Italy. This deeper microbial sequencing will allow us to examine the number and type of microbial organisms transferred from mother to infant at birth and during early life. These sequencing data will also contribute to the identification of microbial species and strains that are unique members of the fecal microbiota of Zimbabwean infants and adults.

For long-term storage plans see section 17.

Though the original consent form stated that infant stool would be examined for soil transmitted helminthes, subsequent information from a national survey documented that the prevalence of STH among school age children was 1.5% in Shurugwi and 0% in Chirumanzu. Thus, finding any STH infection in 18 month old children is extremely unlikely. Accordingly, the research nurse will inform the mother at the 18 month visit that we are no longer going to test the baby’s stool for helminthes because a large survey by the Ministry of Health and Child Care determined this is not a problem among children living in Shurugwi and Chirumanzu. Schistosomiasis will not be screened for routinely in children at 18mo because infection is associated with water-related activities in older children, and there are no documented safety data regarding praziquantel in children <4 years of age.

Maternal samples in EED substudy:

At 1mo postnatal, paired maternal blood and stool will be collected, to enable evaluation of mother-infant pairs soon after birth. 5ml of blood will be collected into an EDTA tube; plasma and PBMC will be extracted and stored, to enable comparison of maternal and infant biomarkers (CRP, sCD14, plasma multiplex cytokines, lipopolysaccharide, IGF-1) and flow cytometry panels. If maternal HIV status was not confirmed using samples collected at baseline and/or 32 weeks, HIV testing will be repeated using this sample to ensure final maternal HIV status has been determined, as stated in the main trial consent form. **5ml** of blood will be collected in a serum gel clot tube. Serum will be extracted and aliquots stored at -80C. For long-term storage plans see section 17. Maternal stool will be collected and aliquots stored at -20C for paired analysis of maternal and infant microbiota, using methods as described on p43.

Infant clinical assessment:

At each visit, infants will be assessed by the DC for acute illness according to IMCI (2005) guidelines. DCs will follow SHINE clinical referral guidelines for any infants with abnormal clinical signs or measurements. The DC will complete a SHINE clinical referral form and ask the mother to take the child to clinic either routinely or urgently, according to the guidelines.

**Risk Factor for Preterm Birth/SGA Sub-Study**

The mother-infant dyads selected into this case control study will be identified prospectively as infants are born. Maternal baseline samples of blood, stool, and urine will be drawn from the archive for analysis as the case and control infants are identified. The informed consent form for the overall trial includes these investigations; therefore no additional consent form will be used for this study.

**Risk Factors for Stillbirth Sub-Study**

The mother-infant dyads selected into this case control study will be identified prospectively as infants are born. Maternal baseline samples of blood, stool, and urine will be drawn from the archive for analysis as the case and control infants are identified. The informed consent form for the overall trial includes these investigations; therefore no additional consent form will be used for this study.

**Risk Factors for Miscarriage Sub-Study**

The mothers selected into this case control study will be identified prospectively as miscarriages are reported. Maternal baseline samples of blood, stool, and urine will be drawn from the archive for analysis as the cases and controls are identified. The informed consent form for the overall trial includes these investigations; therefore no additional consent form will be used for this study.

**Risk Factors for Neonatal Death Sub-Study**

The mother-infant dyads selected into this case control study will be identified prospectively as infants are born and undergo the End of Pregnancy (1mo postnatal visit). Maternal baseline samples of blood, stool, and urine will be drawn from the archive for analysis as the case and control infants are identified. The informed consent form for the overall trial includes these investigations; therefore no additional consent form will be used for this study.

***Pilot intervention for maternal depression***

Poor maternal mental health can compromise caregiving, feeding practices and parenting behaviours, thereby constraining child growth and development (Lovejoy MC, 2000). In a systematic review of studies from developing countries (Surkan PJ, 2011), children of mothers with depression had higher odds of stunting (OR 1.4, 95%CI 1.2-1.7) and underweight (OR 1.5, 95%CI 1.2-1.8). Similarly, in SHINE, maternal depression at baseline was independently associated with higher risk of child stunting at 18 months of age in the standard of care arm.

We will pilot an evidence-based community intervention for depression (*kufungisisa)*, which could form one component of a more comprehensive intervention for healthy child growth. The Friendship Bench is a brief psychological intervention that has been scaled up in urban areas of Zimbabwe because it is efficacious and cost-effective (Chibanda D, 2016). In rural areas, where access to management for common mental disorders is limited, the Friendship Bench could potentially be delivered by Village Health Workers to improve both maternal outcomes and child growth and development. We will identify 30 women with a history of depression based on an Edinburgh Postnatal Depression score >12 at any previous SHINE visit. We have designed an Introductory Script to explain to the mother our reason for visiting. We will gain written informed consent using **SHINE_ Form 80_v1 Friendship Bench Consent Form.** A Data Collector will undertake a home visit to collect data using **SHINE_ Form 81_v1 Friendship Bench Data Collection Form**. This will include details about current depression (*kufungisisa)*, anxiety and other symptoms, using validated tools, and an assessment of interaction between the mother and her child. The maternal-child interaction assessment may be video recorded so that the research team can undertake quality control and independently score the assessment.

Village Health Workers, following residential training in the Friendship Bench, will deliver six sessions of individual problem-solving therapy. A subsample of sessions will be audio-recorded and scored independently by Friendship Bench trainers, with feedback to VHWs. The mother will also be encouraged to join a local group to provide peer support and to establish income-generating activities. At the end of the intervention, a Data Collector will visit the mother at home and repeat the assessment using **SHINE_ Form 81_v1 Friendship Bench Data Collection Form**. In addition, we will conduct 10 in-depth interviews with women, using **SHINE_ Form 82_v2 Friendship Bench Maternal In-Depth Interview Guide**, to evaluate their understanding of depression and the acceptability and feasibility of the Friendship Bench in a rural setting. We will also conduct 2 focus group discussions with women (each comprising 6-8 participants) using **SHINE_ Form 83_v2 Friendship Bench Maternal Focus Group Discussion Guide.** The interview will be audio recorded if the mother provides permission.

A subgroup of 6-8 Village Health Workers will be asked to provide written informed consent to take part in a focus group discussion using **SHINE_ Form 84_v1 Friendship Bench VHW Focus Group Discussion Consent Form.** The interview will explore their experience of the Friendship Bench and will be conducted using the **SHINE_ Form 85_v2 Friendship Bench VHW Focus Group Discussion Guide.**  All audio and video recordings will be destroyed after transcription.

Women found to have clinically significant levels of depression (*kufungisisa)*, suicidal ideation or other clinical concerns will be discussed with the Nurse Intervention Manager and referred to local clinics. The study team will be able to contact the Friendship Bench trainers throughout the project, who will provide clinical advice as needed. If there are concerns that any woman is severely unwell and not able to be managed at the local clinic, a referral will be made to an appropriate health facility for further management. In this situation, the study team will provide information, a referral letter and transport costs to ensure the woman can access treatment.

Number of study contacts or visits required of participants

Research Staff: Each mother will be seen once or twice to seek consent for the trial, twice during pregnancy, 5-6 times post-partum for data collection (at birth (for home deliveries only) using the **SHINE_Form25_v1_Birth Visit** to record birth anthropometry; at 1 month using form **SHINE_Form 14_v5_End of Pregnancy form;** at 3 months postnatal using form **SHINE_Form 20_v4_ 3 month visit**, at 6 months postnatal using form **SHINE_Form 21_v3_ 6 months visit**, at 12 months postnatal using form **SHINE_Form 22_v2_12 months visit**, and 18 months postnatal using **SHINE_Form 23_v2_18 months visit**; plus 2 brief morbidity diary visits using the **SHINE_Form 26_v2_Morbidity diary** during month 2 and one extra diary visit during month 3 for a total of 11-13 contacts. All infants turning 24 months of age from February 2016 onwards will additionally have a visit using **SHINE_Form 63 v5_24 month visit data collection form**. All post-partum visits will be to the mother-infant dyad.

Village Health Workers: Each mother will be seen at least three times during pregnancy and at least monthly between delivery and 18 months post partum.

**Maternal or infant deaths**

VHWs will report maternal or infant deaths in accordance with their regular reporting. A Research Nurse Supervisor and DC will visit the mother together, within 2 weeks of being informed of the event. The timing has been chosen to accommodate the sensitivity of this visit, which will include both a verbal autopsy regarding the maternal or infant death. Maternal verbal autopsies will be conducted with a family member using SHINE Form 35v1 Maternal Verbal Autopsy; infant verbal autopsies will be conducted with the mother, or with another family member in the case of maternal deaths using SHINE Form 13v4 (End of Pregnancy Form) for infants <1 mo old at the time of death, and SHINE Form 45v1 Infant Verbal Autopsy for infants >1 mo old at the time of death. In all cases of maternal or infant death, the study team will also undertake completion of an Adverse Event form (see SAE section).

**Maternal or infant hospitalizations**

VHWs will report maternal or infant hospitalizations (defined as an overnight stay in a medical facility). This will trigger an SAE visit within 2 weeks of being informed of the event.

#### Expected duration of the study

For each mother, we expect to enroll by 24 weeks gestation and follow her until her baby is 18 months old. Thus each mother will participate for up to 25 months. The entire study (from enrollment of the first pregnant mother to the 18 month visit of the last enrolled mother) will run an estimated 3 calendar years. We anticipate at least 2 additional years for laboratory and data analysis. From February 2016, infants will be assessed during one additional visit at 24 months of age.

#### Data Analysis

***Summary of Analytic Approaches for SHINE Outcomes***

As a cluster randomized trial, SHINE outcomes, in general, will be analyzed using statistical methods that account for within-cluster correlation. With a relatively large number of clusters (212), and for some analyses, only a few events or prevalent cases per cluster, generalized estimating equations (GEE) estimation provides an ideal approach to data analysis. We will use the Stata GEE regression procedure, with exchangeable correlation structure within each cluster, for both continuous and discrete response variables. Primary analyses will contain two dummy variables representing each intervention in this 2x2 factorial study; they will be unadjusted for other covariates. A log link function will be used for prevalence outcomes, to aid in interpretability by directly producing risk ratio estimates.

Although this study is not powered to detect a statistical interaction effect between the two interventions, it will be estimated. If there is an important interaction, we will also present results using a regression model with three terms to represent the four study arms.

The case-control sub-studies will be analyzed via conditional logistic regression models. Because of the conditioning on the case-control sets, and the fact there will usually only be one case per cluster, there will be little opportunity for intra-cluster correlation to be exhibited.

#### Screening for Eligibility and Assignment to Study Groups

All women who become pregnant and are identified by the Village Health Worker during their pregnancy surveillance activities are eligible for the trial. A research nurse will visit and request her participation during the informed consent visit. Assignment to treatment group is based on the randomization of the cluster and the research nurse will have no involvement in this process.

#### Masking

Due to the types of interventions being tested in this trial, masking of either the participants or investigators is not possible. However, we have specifically chosen a cluster randomized design and a separation of the intervention and data collection teams to reduce the potential for bias as much as possible.

#### Receipt of Routine Care

All subjects will continue to receive routine care through the government or other health care system. There will be no interference with access to care. In fact, participation in this trial elevates the routine care available to all participants as we have conducted extensive health system strengthening prior to beginning this trial and research staff will promote appropriate health care attendance.

#### Use of Placebo or Non-Treatment Group

No placebo will be used for this trial, but there will be a control group assigned to neither of the intervention arms. The justification for withholding these interventions from the control group is that we still don’t fully understand the separate and combined effects of these interventions on the growth of young children. All participating households will receive a latrine or upgrades to bring a currently owned latrine to MOHCC standard, once the trial period is completed.

#### Describe what happens to participants receiving therapy when the study ends or if a subject’s participation ends prematurely

The trial will not interfere with a participant’s access to care at any time during the study or after the trial ends.

Referring subjects to care outside the study

The data collectors are qualified primary care nurses who will refer participants for care to the appropriate level of the health system based on their professional judgment at the time of each study visit. Village Health Workers are part of the health care system and will refer as well based on their training and the protocols and policies of the MOHCC.

***Loss to follow up***

For each scheduled intervention visit, 2 attempts will be made by a VHW to deliver the appropriate module; if the mother was not available on either occasion then this will be considered a missed module. Regardless of intervention delivery, the modified intent-to-treat analysis will include all infants who enrolled and provided data on length and hemoglobin at the 18 month endpoint. Data on module delivery will be collected for the per-protocol analysis, which in part will be based on successful arm-specific module delivery after 24 gestational weeks. For each research visit, the DC will make at least 2 attempts to visit the woman’s original home; if feasible, field staff will make additional attempts to make a visit within the allowable window. If data cannot be collected, it will be considered a missed visit. Women who move to a new home may be visited by a VHW at their discretion to receive modules but will not receive any intervention inputs: Nutributter (IYCF arms) or latrine, Tippy Taps, soap, Water Guard and infant play space (WASH arms) at their new home. Research visits will not be conducted on mother-infant pairs who move from their original home, except at 18 months, when a final attempt will be made to collect primary endpoint data (height and hemoglobin) in their new location. If women return to their primary home, intervention delivery will resume, and will catch up where hardware inputs (e.g. latrine, Tippy Tap) have been missed (except Nutributter). At each time point, data will be collected on time spent away from home (defined as an overnight stay) since the last DC visit and a cumulative total will be calculated over the course of the trial, to be used in per-protocol analyses.

If study subjects wish to withdraw from the trial, the SHINE Exit Form (**SHINE_Form 24_v3_Exit Form**) will be completed by the Research Nurse Supervisor. Women who withdraw from the trial will no longer receive any study inputs, including a latrine at the end of the trial; VHWs may resume routine MoHCC activities. Study participants who have withdrawn but wish to resume their participation in the study can contact the SHINE team and provide informed written “reconsent” (using intervention arm specific forms, **SHINE_Form 57, 58, 59 and 60v1_Reconsent Form**).

#### Power Calculations

See previous sections for the main trial and sub-studies for these calculations.

#### Diagnostic tests

A number of diagnostic tests will be provided to participants in this trial. This includes HIV testing for mothers and infants, hemoglobin testing for mothers and infants, urine-based assays for schistosomiasis and dip stick urinalysis in mothers, and two blood pressure measurements in mothers at baseline and 32 weeks gestation. All other tests are research oriented and do not currently have clinical usefulness in this setting.

**HIV Test:** Women and infants will be tested for HIV according to the Zimbabwean MOHCC National HIV-1 Testing Algorithm. For women, an anti-HIV antibody rapid test (Alere Determine HIV 1/2; sensitivity 99.9%, specificity 98.2%) will be undertaken in the woman’s home, or in the laboratory if she prefers. If negative, she will be considered HIV uninfected. If positive, a second rapid test (INSTI HIV-1/HIV-2 Rapid Antibody Test; sensitivity >99.8%, specificity >99.5%) will be undertaken immediately. If positive, she will be considered HIV-infected. In the case of discordant results, both rapid tests will be repeated. If both remain discordant, HIV testing will be repeated using HIV DNA PCR in the central laboratory. Infants will have blood collected onto filter paper and the dried blood spot will be tested by HIV DNA PCR at 3, 6, 12 and 18 months (Roche Amplicor HIV-1 DNA test v1.5; sensitivity 100%, specificity 100%) (Zijenah 1999).

**Hemoglobin testing:** Maternal and infant hemoglobin will be measured on whole blood using a HemoCue assay. Mothers with moderate anemia (Hb<100 g/L) will be referred to the local clinic, as per Zimbabwean antenatal guidelines. Infants with severe anemia (hemoglobin <70 g/L) or moderate anemia (hemoglobin <110 g/L) with clinical symptoms will be referred for work-up and care to the local clinic.

**Urine Schistosomiasis Assay**: The classic urinary filtration method will be used for diagnosis, as originally described by Mott(1982) and widely used by field clinicians. Presence of microhematuria by dipstick testing will also be assessed. Although there is no ‘gold standard’ test for diagnosis of urinary schistosomiasis, microscopy was found to be the best of five diagnostic methods evaluated (microscopy, urinary antigen testing, serology, ultrasound or detection of hematuria) in Ghana, with sensitivity of 82-100% and specificity of 93-98%, depending on age and geographical location (Mott 1982, Koukounari 2009). A 10ml aliquot of maternal urine will be syringe filtered through a membrane and the filter examined for eggs of *Schistosoma haematobium*. Results will be expressed as eggs per 10mL urine.

**Blood pressure (BP):** BP will be measured in pregnant women at the two antenatal visits (10-<20 weeks and 32 weeks gestation) using an ADC Advantage 6021 Automatic Electronic Blood Pressure Monitor. Women will be referred to the local clinic, as per Standard Treatment Guidelines for Zimbabwe (EDLIZ) 2011, if they have evidence of: i) a rise in diastolic blood pressure >15mmHg between visits; ii) mild pregnancy-induced hypertension (PIH), ie diastolic BP 90-100mmHg with no proteinuria; iii) moderate PIH, ie diastolic BP 100-110mmHg with no proteinuria; iv) severe PIH, ie diastolic BP >110 mmHg with no proteinuria; v) pre-eclamptic toxemia, ie any of i), ii) or iii) in the presence of proteinuria; vi) imminent eclampsia, ie proteinuria and hypertension with symptoms of visual disturbance or epigastric pain; and vii) eclampsia, ie pregnancy-induced hypertension with seizures.

**Urinalysis:** Urinalysis will be undertaken in pregnant women at the two antenatal visits (10-<24 weeks and 32 weeks gestation) using Bayer Multistix 10 SG Reagent Strips, which measure bilirubin, blood, glucose, ketones, leukocytes, nitrites, pH, protein, specific gravity and urobilinogen. If there is evidence of glycosuria, proteinuria, urinary tract infection (positive leukocytes and/or nitrites), or bilirubinuria, women will be referred to the local clinic.

**Temperature:** Axillary temperature will be measured in children using a single-use, sterile clinical thermometer (3M Tempa-DOT^TM^) according to the manufacturer’s instructions. These are widely used in routine clinical practice, have a sensitivity of 100% and 95.4%, respectively, in children and have been shown to be a reliable alternative to the mercury thermometer in validation studies (van den Bruel 2005).

### Study Procedures in Sequential Order for Village Health Workers

All Village Health Workers operational in the two districts will be approached to provide informed consent for research data collection **(SHINE_Form 11_v3_VHW_Consent_Form)**

Consenting Village Health Workers will be contacted up to 10 times for research data collection during the course of the study – over a 5-year period. On 5 of these visits (at intervals of one year, from the study’s onset) questionnaires **(SHINE_Form 12_v3_VHW_Questionnaire)** will be administered to ascertain Village Health Workers work capacity, performance as well as the motivational and supervisory characteristics of their work. Village Health Workers will also undergo semi-structured observations (shadowing) for up to 4 times during their normal activities (e.g. home visits, intervention delivery visits, report writing, and travelling) to further assess their work performance and establish the fidelity of delivery of study interventions.

## Data Security and Protection of Subject Confidentiality

Prior to data collection all Research Staff will be trained on the protection of human subjects in a 3-day course taught by the Medical Research Council of Zimbabwe staff (MRCZ, the local IRB). Key Zvitambo research staff have previously received local training, facilitated by the MRCZ, on Basic Health Research Ethics and Basic Good Clinical Practice (GCP).

### Applying for a Certificate of Confidentiality?

**No**

### Data security plan

| **Hard Copy of data collection form**: **Indicate your choice but typing an X in the appropriate box on the left:** | |
| --- | --- |
| X | Hard copies of data collection materials have identifiers and are locked in a secure cabinet or room with limited access by specified individuals. When possible, redacted (de-identified) versions of the data collection sheets will be used for coding and analysis. **NOTE:** Paper versions of all forms will be available for use when electronic devices fail for any reason. Paper forms will also be used for administrative purposes (for example, logs of data or specimens being transferred), and laboratory notebooks. |
|  | Hard copies of data collection materials include an ID code and do not have personal identifiers. However, a code linking the data to the subject’s personal information is stored separately from the data collection sheets, and is locked in a secure cabinet or room with limited access by authorized individuals. |
| X | Data are not collected on paper. Most data will be collected on Netbook Computers programmed with the questionnaires. |
|  | Other (describe): |
| **Electronic Databases: Indicate your choice but typing an X in the appropriate box on the left: :**  *Note: A de-identified version of the database should be used for data analysis except in instances in which identifying information is prerequisite for coding or analysis. Databases that retain identifying information require a higher degree of electronic security.* | |
|  | The study is minimal risk and data collected are not sensitive in nature. No personal identifiers are included in the electronic database. Any electronic documents that link IDs to identifying information are stored on a computer in accordance with JHSPH Data Security guidance. |
| X | Personal identifiers are included in the database. The data are stored on a computer that is password protected with a secure server. Transfer or storage on portable devices (e.g., laptops, flashdrives) is encrypted. The devices on which this information is stored are accessible only to individuals who need access to these data. |
|  | No personal identifiers are included in the database but linkable identifiers exist separately and the data are sensitive in nature *(e.g., substance use, mental health, genetic propensities, sexual practices or activities*) such that disclosure could provide a risk to the individual. The codes are stored on a computer that is password protected with a secure server. Transfer or storage on portable devices (e.g., laptops, flash drives) is encrypted. The devices on which this information is stored are accessible only to individuals who need access to these data. |
|  | Other (describe): |

### Description of use of participants’ personal identifiers and plans for disposing of identifiers.

Name and addresses will be collected to identify mothers for subsequent home visits. Data captured on all field computers will be encrypted with secure passwords and when data is transmitted to the Zvitambo/Harare office the transmission will be done over secure networks. Electronic databases will not include personal identifiers, but will include the study ID. A separate file linking the Study ID with the personal identifiers will be stored on main computer in the Zvitambo/ Harare office that is password protected with a secure server.

### Description of plans for destroying data

We do not plan to destroy the electronic data. Any paper forms used will be locked in a secure cabinet or room with limited access by specified individuals and destroyed after it has been computerized or is no longer required and after at least 7 years of storage.

## Recruitment Process

Please see the recruitment process described under study procedures for the main trial.

## Consent process and documentation

### Countries where the research will take place and the languages into which each consent document will be translated

The entire project will be conducted in Zimbabwe.

| **Country** | **Consent Document**  **(Indicate “All”, or specify each document when translations vary)** | **Languages** |
| --- | --- | --- |
| Zimbabwe | **All**   - Trial Consent for Standard of Care Clusters - Trial Consent for Infant Feeding clusters - Trial Consent for WASH clusters - Trial Consent for Infant Feeding + WASH clusters - Consent for specimen archival and shipping - Consent for EE Sub-study- HIV+ mothers - Consent for EE Sub-study HIV- mothers | Shona,  Ndebele, and English |

### Who will obtain informed consent from participants, how, when and where

The informed consent process will take a number of stages including a process of community engagement, followed by individual consent of eligible women.

#### Community Engagement

Three relevant groups will be engaged in order to obtain approval and assent to implement various program activities. These groups include (1) the Community Leadership, (2) Health Workers, and (3) Reproductive-aged women and their families.

(1) Community Leadership has been continuously informed about this study since planning for it began in about 2009. Investigators met with counselors (elected community representatives), traditional chiefs, district administrators, and the leadership of the Rural District Councils. Following their agreement, Project Agreements were signed between each district and Zvitambo. The project agreements detail the community based activities of the SHINE study and define the role of existing structures – the District Social Services Committee and the full council of the rural districts (the Rural District Council) in community engagement. In their roles as community representatives, members of the full council will aid towards communicating study objectives and strategies, communicating views and perceptions of the community, community engagement to maximize uptake/success and proactively dispel misconceptions in the community. Also, in their administrative roles within the Rural District Council, they will ensure the coordination of existing and new community development programs to ensure that they do not compromise the study.

Zvitambo staff have also planned and implemented the aforementioned health systems strengthening activities with district provincial and national health authorities. The Provincial Medical Director has provided assent to randomize clusters in these districts for the trial and deliver randomized program activities through the health delivery system.

(2) Health Workers: Prior to beginning activities for this trial, information sessions will be held with health workers in the districts. These sessions will provide information about the trial and discuss the roles and responsibilities of research staff and linkages with the health delivery system.

(3) Reproductive-aged women and their families: Brochures describing the study will be distributed by Village Health Workers to women of reproductive age during pregnancy surveillance, available in antenatal and well-child clinics, and available in other public places to inform the community (**SHINE Community Brochure_v5, SHINE EED Substudy Community Brochure v1** **, SHINE Early Child Development Assessment Community Brochure v1and SHINE Community Brochure_ Post Recruitment v1)**.

**Individual Consent Process**

The Village Health Worker will conduct routine surveillance for pregnancy using a system designed to detect pregnancies as early as possible in this setting. This is part of the health system strengthening process instituted prior to this trial. Once the VHW identifies a woman who is pregnant, he will ask her if a DC can visit to describe the project. If she assents the Village Health Worker will refer the mother to the DC using only the Village Health Worker ID, the mother’s line number and the date of her LMP in that Village Health Worker’s register – thus, the referral will be done without any personal identifiers. The Village Health Worker will accompany the DC within two weeks to the mother’s home and introduce them. The DC will describe the study and elicit her consent for participation using the treatment group specific consent form that corresponds to the cluster in which she resides.

In cases where the mother dies but the infant is still alive, the new guardian will be asked for consent so that the infant can remain in the trial. We define guardian as the recognized decision-maker for that baby; this person can be identified by the head of household and/or community leaders. Re-consenting will be undertaken using SHINE Form 28v2 Guardian SOC Consent Form, SHINE Form 29v2 Guardian IYCF Consent Form, SHINE Form 30 v2 Guardian WASH Consent Form, SHINE Form 31v2 Guardian WASH+IYCF Consent Form, SHINE Form 32v2 Guardian EED Sub-Study Consent Form

1. **Vulnerable population enrolled?**

Yes, pregnant women are the target group for enrollment. All are from rural Zimbabwe, a setting where poverty is highly prevalent and resources are constrained. It is in this type of setting where the interventions being tested may have the most effectiveness.

We will translate all study forms from English into Shona and Ndebele to ensure accurate understanding of the trial and the processes in place. Illiterate women can provide consent using a thumb print in the presence of a witness..

Because young women in Zimbabwe are often culturally required to discuss decisions (like participating in a trial) with their husband and other family members, we will hold many meetings with community leadership, make brochures available to the general population, and include other family members in the consent discussion if the woman wants this.

We will emphasize participation is voluntary and they are free to refuse participation in any part of the study (i.e., refuse to answer any question posed or to participate in any process requested) or quit the study all together at any time during the study.

### Waiver of consent requested?

No.

## Risks

### Description of risks

Taking blood may cause discomfort, bleeding or bruising where the needle enters the body. In rare cases, it may cause fainting. There is a small risk of infection*.*

### Frequency and severity of risks

Rare risk of fainting and infection.

### Description of measures to minimize risks

All Zvitambo research staff have been trained on the protection of human participants and Good Clinical Practice and are experienced in speaking to people in a sensitive manner and addressing any issues that arise in a positive and supportive manner.

### Description of level of research burden

For each mother, we expect to enroll her by 24 weeks gestation and follow her until her baby is 18 months old. Thus each mother will participate for up to 25 months. The entire study (from enrollment of the first pregnant mother to the 18 month visit of the last enrolled mother) will run an estimated 3 calendar years.

For a women-infant dyad who participates the entire way through the study, she/they will be visited a total of 8-10 times over this 25 month period related to research data collection with an additional 3 brief visits to collect morbidity diaries and encourage their use for dyads in the EED Sub-study. The data collection visits will occur once or twice during early pregnancy to seek consent for the trial, twice more during pregnancy and 5 times post-partum (at 1, 3, 6, 12 and 18 months) for a total of 8-10 visits plus an additional 3 visits for morbidity diaries among EED Sub-study dyads. The baseline visit and any other visit in which the lactulose:mannitol test is conducted on the mother or infant will take at least 2 hours due the length of time urine must be collected. Other data collection visits that do not require this test will typically take 1 hour. The morbidity diary visit will take 5-10 minutes.

Village Health Workers: The number of visits to mothers and infants by village health workers for women and infants enrolled in the trial will be the same as specified by the MOHCC for Village Health Worker care of pregnant women and young children: each mother will be seen at least twice during pregnancy and at least monthly between delivery and 18 months post partum. Village Health Workers will also be contacted up to 10 times for research data collection during the course of the study – over a 5-year period. On 5 of these visits questionnaires will be administered and each questionnaire takes approximately 45 minutes. Village Health Workers will also undergo semi-structured observations (shadowing) for up to 4 times during their normal activities (e.g. home visits, group education, report writing, and travelling).

### Description of how participant privacy will be protected

Privacy will be protected a number of ways for this trial. All paper forms with identifiable information will be stored in locked cabinets at field offices or study headquarters in Harare. The vast majority of data will be collected electronically via direct entry to netbook computers in the field. All data files on the netbooks will be encrypted as will all data transfers from the field to the data center in Harare. HIV tests results will be held in strictest confidence as per national policy in Zimbabwe. As much as possible, all forms or specimens will be labeled only with ID numbers and the linkage between this ID number and identifying information will be held in a secure location. Interviews conducted at the household will be done in a private location away from other adults or older children as appropriate. Maternal HIV status may be measured for all women on the baseline blood and at 32 weeks gestation, but these results will be used only during analysis after personal identifiers are stripped from the HIV result data to interpret primary outcomes by maternal HIV status. Thus, only if women have agreed to learn their status (by either agreeing to rapid testing by the DC at the baseline or 32 weeks gestation visit or having the test at the antenatal clinic which records the results on the antenatal card) will her result be made known to research staff. In addition, only women who agree to learn their status will be eligible for the EED Sub-study. We anticipate the number of women refusing to learn their status will be very low; current PMTCT program data indicates that 99% of pregnant women in the study districts know their HIV status at delivery.

In a community-based trial, people can be stigmatized even if the data and analysis are stripped of all personal identifiers if investigators name the group in reporting a sensitive finding. Recognizing risk of group harm from incidental findings, the steering committee will ensure that publications and public pronouncements of future study findings containing group associations are done with caution, respect for the group under study, and precision.

## Benefits

### Direct Benefits

There are a number of direct benefits to participants in this study. All participants will be from communities in which a systematic health system strengthening process has occurred over the past 2-3 years. In addition, all households will receive a Blair style ventilated improved pit latrine, one half during the mother’s pregnancy, and one half at the end of the trial, or upgrades to an existing latrine to bring it to MOHCC standard. All subjects will be visited regularly by a study nurse who will use his/her professional judgment regarding referral of the woman or her infant for care. At all postnatal visits, mothers will receive a small non-monetary gift to thank them and their infant for their time in participating in data collection. The total value of gifts over the course of the study will not exceed $10.

### Societal Benefits

There are significant potential societal benefits from this study. If the results demonstrate that it is critical to prevent EED from occurring in order to improve the growth of young children, this would provide significant evidence to support a much more aggressive combined sanitation-hygiene and infant nutrition approach in countries with similar settings as rural Zimbabwe.

## Payment

There is no payment for participation.

## Drug Products, Vitamins, Dietary Supplements and Devices

### Drug, Vitamin, and Dietary Supplement products

Nutributter infant diet supplement

#### Rationale:

We selected Nutributter (along with education to the mother) as the randomized nutrition intervention because it is designed to fill key nutrient gaps of breastfed infants in environments where infant malnutrition is common (http://www.nutriset.fr/en/product-range/produit-par-produit/nutributter.html). Our formative research showed that Nutributter closed all the key nutrient gaps of rural Zimbabwean breastfed infants (Paul et al. 2010) The dose of 20 g daily is delivered in a single sachet of a lipid-based fortified paste mixed into at least 2 complementary food servings.

#### Dose Decision

After the age of 6 months, breast milk alone is no longer sufficient to provide all essential nutrients to infants. In contexts like rural Zimbabwe, infant foods are typically low in numerous essential nutrients, and Nutributter is a product specifically designed to provide the daily recommended allowance of all micronutrients (Dewey et al. 2009).

**Table 12: Target nutrient composition of LNS for children (per daily ration)**

| **Nutrient** | **Nutributter -LNS-20gM** |
| --- | --- |
| Ration (g/day) | 20 |
| Total energy (kcal) | **118** |
| Protein (g) | 2.6 |
| Fat (g) | **9.6** |
| Linoleic acid (g) | **4.46** |
| α-Linolenic acid (g) | **0.58** |
| Vitamin A (μg RE) | 400 |
| Vitamin C (mg) | 30 |
| Vitamin B_1_(mg) | 0.3 |
| Vitamin B_2_ (mg) | 0.4 |
| Niacin (mg) | 4 |
| Folic acid (μg) | 80 |
| Pantothenic acid (mg) | 1.8 |
| Vitamin B_6_ (mg) | 0.3 |
| Vitamin B_12_ (μg) | 0.5 |
| Vitamin D (IU) | 200 |
| Vitamin E (mg) | 6 |
| Vitamin K (μg) | 30 |
| Iron (mg)^1^ | **6** |
| Zinc (mg) | **8** |
| Cu (mg) | **0.34** |
| Calcium (mg) | **280** |
| Phosphorus (mg) | **190** |
| Potassium (mg) | **200** |
| Magnesium (mg) | **40** |
| Selenium (μg) | **20** |
| Iodine (μg) | 90 |
| Manganese (mg) | **1.2** |

^1^Target iron content has been reduced from 9 mg in the current formulation of Nutributter to 6 mg, to reduce any potential risk associated with iron intake in malarial areas. As the daily ration will be divided into at least 2 meals during the day, the amount of iron consumed at any single meal should not exceed 3 mg. This falls within the amount of iron that a fortified processed

#### International Approvals

Nutributter is not a regulated drug product in Zimbabwe. Plumpy Nut, which has the same base as Nutributter but provides micronutrient requirements in a 500 kcal dose, is used throughout the country for community-based treatment of severe acute malnutrition.

FDA Regulated Drug Products

(including dietary supplements being used to test for a drug effect):

Nutributter is not a FDA-regulated drug product.

#### Justification and Safety Information

Nutributter formulation is based on current knowledge of infant nutrient requirements. Our formative research showed that provision of Nutributter prevented low nutrient intakes by rural Zimbabwean infants, but nutrient intakes did not exceed the Upper Limits set by the Institute of Medicine (Paul et al., 2009).

#### Responsibility for Drug Management

Nutributter sachets will be managed by the Intervention Nurses, who will distribute sachets to the Village Health Workers randomly allocated to deliver the nutrition intervention. Provision of the Nutributter sachets to VHWs and to mothers will be monitored by the trial as we monitor intervention fidelity and adherence.

### Medical Devices

**HemoCue Hb 301 machine** (HemoCue AB, Angelholm, Sweden): Hemoglobin is measured on 10uL of venous blood, as previously described (van Kempen E J and Zijlstra W G. Spectrophotometry of hemoglobin and hemoglobin derivatives. Advances in Clinical Chemistry, vol 23, 199-257 (1983)). The Hb301 haemoglobinometer has been factory calibrated against the ICSH (International Council for Standardization in Hematology) method and shown to have excellent correlation with the gold standard. The precision of the machine is excellent, with a CV <1.1%; the accuracy of the machine is within 7%, in conformity with CLIA’88 regulations (Federal register: Clinical laboratory improvement amendments of 1988; Final rules and notices. 28 February 1992, 7188-7288). In independent evaluations, it was found to have excellent linearity over a wide range (4-18 g/dL), a low level of imprecision (CV 0.4-0.7%) and an overall performance limit within 6% for 96% of samples, making it extremely reliable for clinical use (Morris et al, 2007).

**ADC Advantage 6021 Automatic Electronic Blood Pressure Monitor** (American Diagnostic Corporation, Hauppauge, NY, USA). This device is manufactured to meet the European and United States standards for non-invasive blood pressure monitors: EN-1060-1/1995, EN1060-3/1997, EN1060-4/2004 and AAMI/ANSI SP10. Clinical performance tests were carried out in the UK using the British Hypertension Society clinical protocol to measure the accuracy of this product, which was graded ‘AA’ for systolic/diastolic accuracy by independent investigators, the highest grading available for BP monitors. The measuring range (systolic/diastolic) is 30-280mmHg, measuring resolution is 1mmHg and accuracy is within ±3mmHg.

BodyStat 1500 MDD (BodyStat, Isle of Man, British Isles) is a non-invasive, portable, bioelectrical impedance analyzer which can measure reactance, resistance and phase angle to enable calculation of lean and fat mass. It works by passing a safe battery-generated signal (i.e. an undetectable electric current) through the body and measuring the impedance at two frequencies (5kHz and 50kHz) using two clips attached to the body.

Knemometer, developed in collaboration with the Danish Institute of Biomedical Engineering, resembles a pair of electronic calipers and records the knee-heel length to a resolution of 0.01mm (Michaelsen *et al*., 1991).

## Safety monitoring

### Participant safety monitoring

Participants will be visited on a regular basis in their homes by a licensed research nurse (DC). This staff member will use his/her best professional judgment regarding when to refer the mother or her infant for care. We do not anticipate any safety concerns with the interventions being tested. If an unusual situation occurs the nurse will report it to his/her supervisor and the health center for resolution, and will complete an SAE form if appropriate.

### Data Safety Monitoring Board (DSMB)

Since this is a randomized trial, we feel it is appropriate to have a DSMB.

#### DSMB membership, affiliation and expertise

The SHINE trial DSMB will be formed of:

**Professor Simon Cousens** (Professor of Statistics, London School of Hygiene and Tropical Medicine) will chair the committee.

**Dr Tariro Makadzange** **(**Lecturer, Department of Internal Medicine, University of Zimbabwe)

**Dr. Hilda Mujuru** (Sr. Lecturer, Department of Paediatrics, University of Zimbabwe).

The DSMB will meet up to three times, the final schedule will be determined in consultation with them at the first meeting.

#### The charge to the DSMB

We will draft a charge to the DSMB just prior to the first meeting and finalize it in consultation with the DSMB members. We consider the reporting responsibility be to the investigative team and not the sponsors of the trial.

#### Plans for providing DSMB reports to the IRB

All DSMB reports will be sent to the investigative team who will review it and respond to any concerns expressed in the report. Both the report and the investigative team’s response will then be forwarded to all the IBRs involved.

### Plans for interim analysis and stopping rules

Current plans call for, at most, one interim analysis when approximately one-half the mother-infant dyads have completed 18 months post-partum follow-up. However, this will be discussed with the DSMB at the first meeting to determine if they think fewer or more interim analyses are appropriate. By the time this halfway point is reached, almost all recruitment will have been completed. As a result we do not anticipate using any formal statistical stopping rules for this trial.

1. **Monitoring of trial activities**

The SHINE Monitoring Plan details all planned and systematic actions that have been established to ensure the SHINE trial is performed and the data are generated, documented, and reported in compliance with the principles of Good Clinical Practice (GCP) and applicable regulatory requirements.

## Plan for reporting unanticipated problems and adverse events.

The interventions involved in this trial including the universal strengthening of the MOHCC Village Health Worker program, the WASH interventions, and the IYCF interventions are all well accepted inputs for public health programs. We will be more aggressive about their implementation that has been done in the past, but no new separate interventions are being tested in this trial. Therefore, we do not anticipate any major adverse events that could be attributed to this study.

To minimize risk of peanut allergic reaction, Village Health Workers will be trained to test feed 1 teaspoon of Nutributter to the infant to ascertain any reaction. We do not expect to identify any cases of peanut allergy in the study population. Peanuts are a staple food in Zimbabwe yet peanut allergy is unknown. Even in the United States – where there is much attention given to peanut allergy – severe allergies to peanut are uncommon among infants under two years of age. Both the overall prevalence of allergy and the prevalence of severe allergy are very similar to those for milk (1.4-2.0% for any allergy and 0.6-0.7% for severe allergy) (Gupta *et al*, 2011). There is very little information available on peanut allergies in developing countries, but prevalence may be lower than in the U.S.(Yang *et* al, 2010) In an on-going trial giving this product to several thousand infants in Burkina Faso and Malawi, not a single reaction has been observed (iLiNS/ U California, Davis, personal communication). In addition, both the American Academy of Pediatrics and the National Institute of Allergy and Infectious Diseases advise no general restriction on introduction of potentially allergenic foods in infancy (after 6 months of age, when complementary feeding is recommended to commence) (Greer *et al*, 2008; Burks *et al*, 2011).

**Adverse Event and Serious Adverse Event reporting**

An Adverse Event (AE) is defined as any untoward medical occurrence in an enrolled study participant, regardless of its relationship to trial activities.

Serious Adverse Events (SAEs) are defined by the ICH Harmonised Tripartite Guidelines for Clinical Safety Data Management, Definitions and Standards for Expedited Reporting (1994), i.e. an incident that involved an enrolled study participant and includes one or more of the following:

1. Results in death of a study participant
2. Is life threatening to a study participant (ie at risk of death at the time of the event)
3. Requires unplanned hospitalization, OR prolongation of existing hospitalization of a study participant
4. Results in persistent or significant disability/incapacity of a study participant
5. Is a congenital anomaly or birth defect of a study baby
6. Is any other important medical event considered serious by the investigator of a study participant (eg anaphylaxis requiring emergency treatment but not resulting in hospitalization). An important medical event should carry a real, not hypothetical, risk of one of the above events (1-5) if prompt medical action were not taken.

Where the study team first learns about an AE or SAE outside of a scheduled study visit (e.g. report to the field hub by a VHW, or other staff member), a home visit will be made as soon as possible to collect information about the event. Where an AE or SAE is reported at a scheduled study visit, information will be collected during the visit.AEs and SAEs will be reported on an Event Form. The staff member reporting the event will complete part 1 of the Event Form and submit it to the Research Nurse Supervisor or Nurse Intervention Manager at the Hub the same day. The RNS/NIM will review all Event Forms within 24 hours of the form being completed, and will fill in Part 2 of the form. The RNS/NIM will pass the form to the Field Data Supervisor in the hub, who will create an electronic record of the event on the database, within 24 hours of receiving the form; this will generate an Event number.

The Compliance Team at Zvitambo will review all Event forms within 48 hours of receipt. The compliance team will adjudicate the event and check Part 1 and Part 2 of the form, then provide a summary of the event(s) and decisions made. Adverse Events and SAEs will be judged as either unlikely, uncertainly, probably or definitely related to the study interventions or procedures.

Since this is a community-based trial, we anticipate a large number of AEs and SAEs that are unrelated to trial activities. Because of the rural location and limited means of communication within the study districts, it is unlikely that the trial team will find out about most AEs and SAEs in real time.

**Reporting of events to the local IRB (MRCZ) and international IRB (JHU)**

Events will be reported to MRCZ according to the following guidelines:

1. AEs and SAEs identified **related to trial activities**:

Where AEs and SAEs are judged to be uncertainly, probably, or definitely related to the study interventions or procedures, they will bereported to MRCZ and JHU IRB under the expedited reporting time-frame (MRCZ: 3 days for SAE, 7 days for AE):JHU within 10 days of the investigators learning of the event.

The potential adverse events that may be related to our interventions (although have not been described to date by the manufacturers or distributors) are reactions to Nutributter and accidental ingestion of Waterguard. To be certain that we detect any reactions to Nutributter, we will ask a series of questions during home visits, from 6 months of age, to all mothers whose babies are randomized to receive Nutributter. Any adverse events will be followed up with a full AE/SAE narrative in the field, and expedited reporting to MRCZ, JHU IRB and the DSMB. Any cases of accidental Water Guard ingestion will similarly undergo expedited reporting to MRCZ, JHU IRB and the DSMB.

2. AEs and SAEs un**related** to trial activities:

AEs and SAEs that are judged unlikely related to study interventions and procedures will be reported annually to MRCZ and JHU IRB in a table (split by type of AE or SAE). Clinical events that led to attendance at a clinic will be reported, to capture those events that are likely to be grade 3 or 4 (serious or life threatening). Sufficient data will not be captured to enable grading of every AE symptom according to DAIDS tables.

3. Reporting of abnormal clinical investigations conducted as part of study protocol:

Abnormal results which are grade 3 or 4 adverse events according to DAIDS tables (or qualify for referral according to Zimbabwean guidelines, where not covered by DAIDS tables), will be reported to MRCZ and JHU IRB in an annual table. These will be graded as shown in Table 13 and 14 below:

**Table 13: Classification Table for Women enrolled in SHINE**

| **Parameter** | **Grade 3**  **Severe*** | **Grade 4**  **Potentially life threatening*** |
| --- | --- | --- |
| Haemoglobin (known HIV positive woman) | 6.5 – 7.4 g/dL | <6.5 g/dL |
| Haemoglobin (known HIV negative woman, or HIV status unknown) | 7.0 – 8.9 g/dL | <7.0 g/dL |
| Hypertension | >180 mmHg systolic OR  >110 mmHg diastolic | Life-threatening consequences (eg malignant hypertension) or hospitalization indicated (other than clinic visit) |
| Hypotension | Symptomatic, requiring admission for IV fluids | Shock requiring vasopressors or mechanical assistance to maintain blood pressure |
| Malnutrition | MUAC <230 mm | MUAC <190 mm OR bilateral pitting oedema |
| Proteinuria | 4+ on urinary dipstick testing | NA |
| Hematuria | Gross hematuria | Hematuria requiring transfusion |
| Maternal depression | Alteration in personality-behaviour causing inability to perform usual social and functional activities | Behaviour potentially harmful to self or others (eg suicidal and homicidal ideation or attempt, acute psychosis) OR causing inability to perform basic self-care functions. |
| New HIV infection | NA | NA |
| New urinary schistosomiasis | NA | NA |

*Grading based on Division of AIDS (DAIDS) table for the grading of adverse events (published December 2004, with clarification August 2009) where available. Malnutrition parameters based on Zimbabwe MOHCC criteria for referral for nutritional rehabilitation, taken from ‘Malnutrition Management of Acute Malnutrition in Zimbabwe’ (Version 1; June 2011).

**Table 14: Classification Table For Infants Enrolled In SHINE**

| **Parameter** | **Grade 3**  **Severe*** | **Grade 4**  **Potentially life threatening*** |
| --- | --- | --- |
| Haemoglobin (infant aged 22-35 days) | 7.0 – 7.9 g/dL | <7.0 g/dL |
| Haemoglobin (infant aged 36-56 days) | 6.0 – 6.9 g/dL | <6.0 g/dL |
| Haemoglobin (infant or child aged >57 days) | 7.0 – 8.9 g/dL | <7.0 g/dL |
| Malnutrition in a child <6 months | Weight-for-length Z-score <-3 OR MUAC <110 mm  Bilateral pedal oedema OR  Infant too weak to feed or suckle effectively | Grade 3 criteria PLUS any IMCI danger sign |
| Malnutrition in a child >6 months | Weight-for-height Z-score <-2 OR  Bilateral pedal oedema OR  MUAC <125mm | Grade 3 criteria PLUS any IMCI danger sign |
| New HIV infection | NA | NA |
|  |  |  |

*Grading based on Division of AIDS (DAIDS) table for the grading of adverse events (published December 2004, with clarification August 2009) where available. Malnutrition parameters adapted from Zimbabwe MOHCC criteria for referral for nutritional rehabilitation (Malnutrition Management of Acute Malnutrition in Zimbabwe, Version 1; June 2011) and from published data on predictive value of MUAC in infants <6 mo old (Mwangome 2012).

If any abnormal investigation also fulfils SAE criteria (eg severe anemia leading to hospitalization), it will be reported in the SAE section of the Tables.

If any mother or child enrolled in the trial is found to be acutely unwell at a scheduled home visit, they will be assessed by the study research nurse as per their training. If a mother or child needs to be referred to clinic for assessment then an AE form will be completed.

**Additional reporting to the international and local IRB**

We will also report the following to the JHU IRB and MRCZ within 10 working days of learning of the event: i) information that indicates a change to the risk:benefit ratio of the research; ii) withdrawal from marketing of a product being used (Nuributter or Waterguard); iii) changes to the protocol taken without prior IRB review to eliminate an apparent immediate hazard to a research participant; iv) incarceration of a participant; v) any event that requires prompt reporting to the sponsor; vi) complaint of a participant where the complaint indicates unexpected risks or the complaint cannot be resolved by the research team; vii) protocol violations (meaning an accidental or unintentional change to the IRB approved protocol) or breaches that placed one or more participants at increased risk, or has the potential to occur again.

**Reporting of events to the DSMB**

Any grade 3 or 4 AEs or SAEs that are related to Nutributter or Water Guard will be reported to the DSMB within 3 days of the investigators learning of the event. All other SAEs (excluding abnormal laboratory results) will be reported to the DSMB every quarter, split by trial arm.

**Reporting of events in the long-term follow-up study**

Children will be seen for a long-term follow-up visit at 7 years of age and will then have health monitoring by VHW through monthly visits. There are no trial interventions in the long-term follow-up of children in SHINE. We will report adverse events and serious adverse events that are related to any trial procedures that we undertake during the follow-up visits (e.g. venepuncture), but we will not report events that occur in the community and are identified through prospective health surveillance.

## Ethics Review Boards

The Medical Research Council of Zimbabwe (MRCZ) will oversee this study. The University of London recognizes the MRCZ as having jurisdiction over research in Zimbabwe.

## Supervision Roles and Responsibilities

**Prof Andrew Prendergast** (Clinician Scientist from University of London, resident in Zimbabwe) will be the Principal Investigator of SHINE from 2021. He was responsible for developing the human and technical capacity of the Zvitambo Laboratory to receive, process and analyze samples and oversaw the implementation of clinical assessment protocols and of field research protocols by Research Nurses during the main trial. He will lead all aspects of the ongoing SHINE trial as PI. **Naume Viola Tavengwa** (Assistant Director, Field Operations) will be responsible for overseeing the implementation of study interventions and field operations for effective module delivery by Village Health Workers in line with government policy and programming guidance and for communication between organisations and key government/community level stakeholders. She will lead a team that oversees improved research visit outcomes. **Robert Ntozini** (Associate Director IT/Data Management/Statistics) will set up the field and laboratory information systems and oversee data management processes and statistical analyses; **Batsirai Mutasa (Field Data Coordinator)** will coordinate and supervise field data management processes implemented by Field Data Supervisors at each hub;  **Florence Majo** (Research Nurse Coordinator) will coordinate and supervise all field research activities by Research Nurses and Data Collectors; **Kuda Mutasa** (Laboratory Manager) will manage the field laboratory Technicians, field laboratories and central laboratories in line with government policy guidance and oversee the implementation of all specimen processing assays; **Virginia Sauramba** (Research Administration and Compliance Manager) will manage, monitor and oversee compliance activities to ensure Good Clinical Practice procedures are followed and implemented in line with regulatory ethical guidelines and government policy guidance and also ensuring linkages with relevant Johns Hopkins University and SPH offices and department.

## External Investigator Roles and Responsibilities

Dr Mduduzi Mbuya, who was Associate Director, Implementation Science was responsible for the design of randomized interventions, the development of implementation research protocols and oversaw the delivery of study interventions now working for GAIN, USA, will continue to contribute towards SHINE manuscript writing and lead some of them. **Prof. Jean Humphrey**, Professor at Johns Hopkins University, who was former director of Zvitambo and Co PI of the SHINE trial, will remain a co-investigator on the trial in the USA.

*Ministry of Health and Child Care:*

The Ministry of Health and Child Care co-investigators include **Goldberg Mangwadu**, Director of Environmental Health who is also co- Principal Investigator of the study; **Cynthia Chasokela**, Director of Nursing Services; and **Ancikaria Chigumira**, Deputy Director Nutrition. The study team works closely with the Provincial and District Health Executives towards the planning and implementation of health systems activities. These authorities ensure the relevance as well as the consistency of these activities with prevailing policy and programming guidance

*Collaborating Universities:*

A multidisciplinary team of researchers within and outside Zimbabwe will support the Zvitambo and Ministry of Health and Child Care team at all stages of the project’s design and implementation with expertise in nutrition, epidemiology, statistics, program implementation and public health program evaluation. Co-investigators **Dr. Larry Moulton, Dr James Tielsch,** and student investigator **Tatenda Mupfudze** (PhD) from Johns Hopkins School of Public Health. Co-investigators **Dr Rebecca Stoltzfus, Dr. Andrew Jones** and student investigators **Rukundo Kambarami** (PhD), **Cynthia Matare** (PhD) and **Dadirai Fundira** (PhD) from Cornell University, Co-investigator **Laura Smith** (PhD) and student investigator Nadia Koyratty, (PhD) from University at Buffalo. Dr Joseph Piper (PhD), Dr. Ceri Evans (PhD), **Dr Christie Noble** (PhD) and **Dr Jonathan Broad** (PhD) from University of London will visit the project on a regular basis to provide planning and implementation support. Dr James Andrew Church from University of London and **Jaya Chandna** (ECD specialist from the University of Liverpool) names will remain in the study as contributors to SHINE write ups and manuscripts.

## Oversight plan for studies conducted at non-JHSPH sites

The JHBSPH PI and co-investigators are part of a study team who will manage the project along with colleagues from the Zimbabwe Ministry of Health and Child Care, Cornell University, and University of London. The project supervisory staff are experienced co-investigators and field managers. Quality control procedures are emphasized during project-specific training and this is reviewed on a regular basis by study co-investigators.

Our staff in Zimbabwe have received training in human subjects research from the Medical Research Council of Zimbabwe. Newly hired research staff for this project will undergo human subjects training in addition to project-specific task training. This will involve presentations and discussions on the importance of voluntary participation, informed consent, confidentiality, data security, and sensitivity to the participants in the research. These presentations will be reinforced with role playing and direct supervision of the process during the early stages of the study.

**Table 15: Roles and Responsibilities Matrix for IRB Application. For the following, indicate “P” for “Primary”, “S” for “Secondary” as appropriate to role and level of responsibility.) Add additional items if useful.**

|  | JHSPH | Zvitambo | Cornell Univ. | Univ. of London | Zimbabwe Ministry of Health and Child Care |
| --- | --- | --- | --- | --- | --- |
| Primary Grant Recipient | Primary scientific and administrative responsibilities |  |  |  |  |
| Subcontractor |  | Implementation of the trial | Provision of nutritional expertise | Provision of immunologic scientific expertise | Coordination of project activities with MOHCC system |

| 1 | Human subjects research ethics training for data collectors | S | P | S | S | S |
| --- | --- | --- | --- | --- | --- | --- |
| 2 | Day to day management and supervision of data collection | S | P | S | S | S |
| 3 | Reporting unanticipated problems to the JHSPH IRB/Sponsor | S | P |  |  |  |
| 4 | Hiring/supervising people obtaining informed consent and/or collecting data | S | P | S | S | S |
| 5 | Execution of plan for data security/protection of participant data confidentiality, as described in Sect. 5. | S | P |  |  |  |
| 6 | Biospecimen processing, storage, management, access, and/or future use | S | P | S | S |  |

## Creation of a biospecimen repository

### Source and purpose of the biospecimens

Aliquots of plasma, serum, PBMCs, feces, urine and saliva from each collection time point will be cryopreserved at -80°C for all women following informed consent. Aliquots of plasma, serum, cell pellets and feces from all infants at 18 mo of age will be cryopreserved at -80°C following informed consent. Aliquots of plasma, PBMCs, RNA (see below), feces, urine and saliva, collected from all infants in the EED substudy at 1, 3, 6, 12 and 18 mo of age, will be cryopreserved at -80°C following informed consent. Samples will be used to measure pre-specified analytes and will be archived for subsequent analyses of new biomarkers that will inform the aims of the trial, or future studies of maternal/child health that can be usefully addressed with this specimen archive. Specific consent will be obtained to extract DNA from stored biospecimens of women and infants using **SHINE_Form9v5_Specimen Storage and Shipment Consent Form** (see below).

### Biospecimen storage

All samples will be stored in the Zvitambo laboratory, Harare, Zimbabwe in a bank of -80C freezers that are kept in a locked store room with immediate back-up generator in case of power failure. Samples will be stored for up to 25 years from the date of collection.

We will add an archive flag to the participant IDNO to specify how specimens are to be processed.  Eg we will have IDNO=1023-0 or IDNO=1023-1 for archive and don't archive samples.  The full IDNO will be generated after the consent process when we have an indication of the method of processing based on the two options we provide to the participants during the consent process: 1) allow to store, and 2) do not allow to store. Barcode labels will incorporate the flag and the LIS can be programmed to prompt if any flagged samples are being used in assays.

### Sharing of biospecimens

Access to biospecimens by other researchers will be considered following review of a proposal by SHINE investigators and subject to approval by the Medical Research Council of Zimbabwe and/or Research Council of Zimbabwe, as appropriate. In particular, if any samples requiring export for analysis, approval will have to be granted by RCZ.

### Future specimen derivation and processing (cell lines, DNA/RNA, etc.)

Consent will be obtained to extract and store DNA from enrolled mothers and infants using **SHINE_Form9v5_Specimen Storage and Shipment Consent Form** and to extract and store RNA from infants enrolled to the EED substudy using **SHINE_Form10v5Specimen Storage and Shipment Consent Form – EED Infants**. For RNA, blood will be stored in PAXgene RNA Tubes at -80C from infants in the EED substudy at 5 time-points (1, 3, 6, 12 and 18 mo of age) to characterize gene expression profiles in stunted versus non-stunted infants; this will enable us to explore mechanistic pathways to stunting using a non-biased approach. For DNA, cell pellets from infants at the 18 mo blood draw will be stored at -80C and DNA subsequently extracted. DNA will be stored for use in future studies of genetic determinants of infant growth and health. As stated in **SHINE_Form9v5** and **SHINE_Form10v5**, maternal and infant samples for women who do not give consent for long-term storage and shipment (including extraction and storage of DNA and RNA) will be specifically identified by bar-code and not used for future studies.

### Specimen coding and identification

Samples will be bar-coded with no patient identifiers, and logged on an in-house Laboratory Information Management System that is only accessible on password-protected computers stored in locked offices.

### Certificate of Confidentiality protections

Not applicable.

### Ability of participant to withdraw consent

Participants will be required to sign a consent form to agree to storage and possible shipment of samples. The consent form states that participants are free to withdraw their consent at any time.

### Data and/or specimen use agreements

The final dataset will include maternal-reported demographic, behavioural, morbidity data, observed and maternal-reported household data, and laboratory data from urine, fecal, and blood samples collected from the infant. The dataset will be stripped of all personal identifiers prior to release for sharing. It is an expectation of the Medical Research Council of Zimbabwe (MRCZ), one of the IRB’s of record for the trial, that a Zimbabwean be a co-author on publications resulting from data collected on Zimbabwean human subjects. We will make publicly available through a website a description of the database and a description of the specimen archive that has been collected. Anyone who wants to conduct further data analyses should present a proposal of the intended use to a steering committee who will determine if the proposed study will generate important information and whether the trial data is relevant for the analysis.  If so, the committee would assign at least one member of the trial team (including a Zimbabwean) to join this secondary data analysis team.  This person would be responsible for obtaining ethical permission for the additional analyses from the committees that governed the trial - the MRCZ and the IRB of JHU, and for timely communication of findings with the MRCZ and any other relevant Zimbabwean authorities.

As noted in section 8e, in a community-based trial, people can be stigmatized even if the data and analysis are stripped of all personal identifiers if investigators name the group in reporting a sensitive finding. Recognizing risk of group harm from incidental findings, the steering committee will ensure that publications and public pronouncements of future study findings containing group associations are done with caution, respect for the group under study, and precision.

## Data Coordinating Center

Not applicable.

## 20. References

Allen HE et al.New policies for using antihelminthics in high risk groups.*Trends in Parasitology* 2002;18:381-382.

Bhutta ZA, Ahmed T, Black RE, Cousens S, Dewey K, Giugliani E, Haider BA, Kirkwood B, Morris SS, Sachdev HPS, Shekar M. Maternal and child undernutrition 3: Whats works? Interventions for maternal and child undernutrition and survival.*The Lancet* 2008; 371:417-440.

Black RE, Allen LH, Bhutta ZA, Caulfield LE, De Onis M, Ezzati M, Mathers C, Rivera J. Maternal and undernutrition: global and regional exposures and health consequences. The Lancet 2008; 371:243-260.

Briend A. Is diarrhoea a major cause of malnutrition among the under-fives in developing countries? A review of available evidence.Eur J ClinNutr 1990; 44:611-628.

Burks AW, Jones SM, Boyce JA, Sicherer SH, Wood RA, Assa'ad A, Sampson HA. NIAID-sponsored 2010 guidelines for managing food allergy: applications in the pediatric population. Pediatrics. 2011 Nov;128(5):955-65. Epub 2011 Oct 10.

Charbonneau MR, O’Donnell D, Blanton LV *et al.* Sialylated milk oligosaccharides promote microbiota-dependent growth in models of infant undernutrition. Cell 2016; 164:1-13.

Checkley W, Buckley G, Gilman RH, Assis AMO, Guerrant RL, Morris SS, Molbak K, Valentiner-Branth P, Lanata CF, Black RE, The Childhood Malnutrition and Infection Network. Multi-country analysis of the effects of diarrhoea on childhood stunting.International Journal of Epidemiology 2008; 37:816-830.

Chibanda D, Weiss HA, Verhey R, et al. Effect of a Primary Care-Based Psychological Intervention on Symptoms of Common Mental Disorders in Zimbabwe: A Randomized Clinical Trial. JAMA 2016;**316**(24):2618-26

Dewey KG, Adu-Afarwuah S. Systematic review of the efficacy and effectiveness of complementary feeding interventions in developing countries. In: Maternal and Child Nutrition. Blackwell Publishing Ltd: Program in International and Community Nutrition, University of California, Davis, California, USA, 2008; pp 24-85.

Formulations for fortified complementary foods and supplements: Review of successful products for improving the nutritional status of infants and young children, K.G. Dewey, J.Berger, J.Chen, C. Chen, S. Pee, S. Huffman, A. Lartey, C. Lutter, K.Maleta, E. Turner, M. Zeilani, S.Zlotkin, Food and Nutrition Bulletin, 2009 (vol. 30, no. 2, supplément), the United Nations University. <http://foodandnutritionbulletin.org/FNB/index.php/FNB/issue/view/165>

Dupont WD, Plummer WD: "Power and Sample Size Calculations: A Review and Computer Program", Controlled Clinical Trials 1990; 11:116-28.

Gray RH, Wawer MJ, Serwadda D, et al. Population-based study of fertility in women with HIV-1 infection in Uganda.Lancet 1998; 351:98-103.

Greer FR, Sicherer SH, Burks AW; American Academy of Pediatrics Committee on Nutrition; American Academy of Pediatrics Section on Allergy and Immunology. Effects of early nutritional interventions on the development of atopic disease in infants and children: the role of maternal dietary restriction, breastfeeding, timing of introduction of complementary foods, and hydrolyzed formulas. Pediatrics. 2008 Jan;121(1):183-91.

Gupta RS, Springston EE, Warrier MR, Smith B, Kumar R, Pongracic J, Holl JL. The prevalence, severity, and distribution of childhood food allergy in the United States. Pediatrics. 2011 Jul;128(1):e9-17. Epub 2011 Jun 20.

Hayes RJ, Bennett S. Simple sample size calculation for cluster-randomized trials. International Journal of Epidemiology 1999; 28: 319-326.

Katz J. Sample-size implications for population-based cluster surveys of nutritional status.American Journal of Clinical Nutrition 1995;61:155-60.

Keusch GT, Rosenberg IH, Denno DM, Duggan C, Guerrant RL, Lavery JV, Tarr PI, Ward HD, Black RE, Nataro JP, Ryan ET, Bhutta ZA, Coovadia H, Lima A, Ramakrishna B, Zaidi AK, Burgess DC, Brewer T. Implications of acquired environmental enteric dysfunction for growth and stunting in infants and children living in low- and middle-income countries. Food Nutr Bull 2013; 34(3): 357-64.

Koukounari A, Webster JP, Donnelly CA, Bray BC, Naples J, Bosompem K, Shiff C.

Sensitivities and specificities of diagnostic tests and infection prevalence of Schistosomahaematobium estimated from data on adults in villages northwest of Accra, Ghana. Am J Trop Med Hyg. 2009; 80(3):435-41.

Liu J, Kabir F, Manneh J *et al*. Development and assessment of molecular diagnostic tests for 15 enteropathogens causing childhood diarrhoea: a multicenter study. Lancet Infect Dis 2014; 14:716-24.

Lovejoy MC, Graczyk PA, O'Hare E, et al. Maternal depression and parenting behavior: a

meta-analytic review. Clinical psychology review 2000;**20**(5):561-92

Michaelsen KF, Skov L, Badsberg JH and Jorgensen M. Short-term measurement of linear growth in preterm infants: validation of a hand-held knemometer. Pediatric Research 1991; 30: 464-8.

Mott KE, Baltes R, Bambagha J, Baldassini B. 1982.Field studies of a reusable polyamide filter for detection of *Schistosma haematobium* eggs by urine filtration. Tropenmedlizin and Parasitologie, 3:227 – 228.

Morris LD, Osei-Bimpong A, McKeown D, Roper D, Lewis SM. Evaluation of the utility of the HemoCue 301 haemoglobinometer for blood donor screening. Vox Sanguis 2007; 93(1) 64-9.

Moulton LH. Covariate-based constrained randomization of group-randomized trials. Clinical Trials 2004; 1:297-305.

Mwangome MK, Fegan G, Fulford T, Prentice AM, Berkley JA. Mid-upper arm circumference at age of routine infant vaccination to identify infants at elevated risk of death: a retrospective cohort study in the Gambia. Bull World Health Organ 2012; 90(12): 887-94.

Olds GR. Administration of praziquantel to pregnant and lactating women.*ActaTropica* 2003, 86:185-195.

Paul, K. H., Muti, M., Chasekwa, B., Mbuya, M. N., Madzima, R. C., Humphrey, J. H. and Stoltzfus, R. J. (2010), Complementary feeding messages that target cultural barriers enhance both the use of lipid-based nutrient supplements and underlying feeding practices to improve infant diets in rural Zimbabwe. Maternal & Child Nutrition.doi: 10.1111/j.1740-8709.2010.00265.x

Savioli L, Crompton DWT, Neira M. Use of antihelminthic drugs during pregnancy.*American Journal of Obstetrics and Gynecology,* 2003, 188:5-6.

Shrimpton R, Victora CG, De Onis M, Lima RC, Blossner M, Clugston G. Worldwide timing of growth faltering: implications for nutritional interventions. Pediatrics 2001; 107(5):1-7.

Surkan PJ, Kennedy CE, Hurley KM, et al. Maternal depression and early childhood growth in developing countries: systematic review and meta-analysis. Bull World Health Organ 2011;**89**(8):608-15

Urassa EJN, Kileqo C, Mtavangu SR, et al. The role of HIV infection in pregnancy wastage in Dar es Salaam, Tanzania.JObstetGynaecol Central Afr 1992; 10:70-72.

Van den Bruel A, Aertgeerts B, De Boeck C, Buntinx F. Measuring the body temperature: how accurate is the Tempa Dot? Technol Health Care 2005;13(2):97-106.

Victora CG, Adair L, Fall C, Hallal PC, Martorell R, Richter L, Sachdev HS. Maternal and child undernutrition 2: Maternal and child undernutrition: consequences for adult health and human capital. *The Lancet* 2008; 371:340-357.

VictoraCG, De Onis M, Hallal PC, Blossner M, Shrimptom R. Worldwide timing of growth faltering: revisiting implication for intervention. Pediatrics 2010; 125: 473-480.

World Health Organization.Report of the WHO Informal COnsultatio on the use of Praziquantel during pregnancy/lactation and Albendazole/Mebendazole in children under 24 months.WHO, Geneva, 2002. Available at whqlibdoc.who.int/hq/2003/WHO_CDS_CPE_PVC_2002.4.pdf

World Health Organization.Preventive Chemotherapy in Human Helminthiasis. Coordinated use of antihelminthic drugs in control interventions: a manual for health professionals and programme managers. WHO, Geneva, 2006. Available at whqlibdoc.who.int/publications/2006/9241547103_eng.pdf

World Health Organization.Indicators for Assessing Infant and Young Child Feeding Practices.Part II Measurement.WHO 2010.

Yang Z. Are peanut allergies a concern for using peanut-based formulated foods in developing countries? Food Nutr Bull. 2010 Jun;31(2 Suppl):S147-53.

[Zijenah LS](http://www.ncbi.nlm.nih.gov.ezproxy.welch.jhmi.edu/pubmed?term=%22Zijenah%20LS%22%5BAuthor%5D), [Humphrey J](http://www.ncbi.nlm.nih.gov.ezproxy.welch.jhmi.edu/pubmed?term=%22Humphrey%20J%22%5BAuthor%5D), [Nathoo K](http://www.ncbi.nlm.nih.gov.ezproxy.welch.jhmi.edu/pubmed?term=%22Nathoo%20K%22%5BAuthor%5D), [Malaba L](http://www.ncbi.nlm.nih.gov.ezproxy.welch.jhmi.edu/pubmed?term=%22Malaba%20L%22%5BAuthor%5D), [Zvandasara P](http://www.ncbi.nlm.nih.gov.ezproxy.welch.jhmi.edu/pubmed?term=%22Zvandasara%20P%22%5BAuthor%5D), [Mahomva A](http://www.ncbi.nlm.nih.gov.ezproxy.welch.jhmi.edu/pubmed?term=%22Mahomva%20A%22%5BAuthor%5D), [Iliff P](http://www.ncbi.nlm.nih.gov.ezproxy.welch.jhmi.edu/pubmed?term=%22Iliff%20P%22%5BAuthor%5D), [Mbizvo MT](http://www.ncbi.nlm.nih.gov.ezproxy.welch.jhmi.edu/pubmed?term=%22Mbizvo%20MT%22%5BAuthor%5D) Evaluation of the prototype Roche DNA amplification kit incorporating the new SSK145 and SKCC1B primers in detection of human immunodeficiency virus type 1 DNA in Zimbabwe. J ClinMicrobiol 1999; 37(11):3569-71.

Chang, S. M., S. P. Walker, S. Grantham‐Mcgregor and C. A. Powell (2010). "Early childhood stunting and later fine motor abilities." Developmental Medicine & Child Neurology **52**(9): 831-836.

Fernald, L. C., E. L. Prado, P. Kariger and A. Raikes (2017). A Toolkit for Measuring Early Childhood Development in Low- and Middle-Income Countries. T. W. B. Strategic Impact Evaluation Fund, World Bank Group**:** 128.

Lelijveld, N., A. Seal, J. C. Wells, J. Kirkby, C. Opondo, E. Chimwezi, J. Bunn, R. Bandsma, R. S. Heyderman, M. J. Nyirenda and M. Kerac (2016). "Chronic disease outcomes after severe acute malnutrition in Malawian children (ChroSAM): a cohort study." The Lancet Global Health **4**(9): e654-e662.

Martorell, R. (2017). "Improved nutrition in the first 1000 days and adult human capital and health." American journal of human biology : the official journal of the Human Biology Council **29**(2): 10.1002/ajhb.22952.

McGrath, P. J., P. T. Pianosi, A. M. Unruh and C. P. Buckley (2005). "Dalhousie dyspnea scales: construct and content validity of pictorial scales for measuring dyspnea." BMC Pediatrics **5**(1): 33.

Obradović, J., M. J. Sulik, J. E. Finch and N. Tirado-Strayer (2018). "Assessing students' executive functions in the classroom: Validating a scalable group-based procedure." Journal of Applied Developmental Psychology **55**: 4-13.

Ocansey, M. E., S. Adu-Afarwuah, S. M. Kumordzie, H. Okronipa, R. R. Young, S. M. Tamakloe, B. M. Oaks, K. G. Dewey and E. L. Prado (2019). "Prenatal and postnatal lipid-based nutrient supplementation and cognitive, social-emotional, and motor function in preschool-aged children in Ghana: a follow-up of a randomized controlled trial." Am J Clin Nutr **109**(2): 322-334.

Prado, E. L., L. M. Larson, K. Cox, K. Bettencourt, J. N. Kubes and A. H. Shankar (2019). "Do effects of early life interventions on linear growth correspond to effects on neurobehavioural development? A systematic review and meta-analysis." The Lancet Global Health **7**(10): e1398-e1413.

Ruisenor-Escudero, H., I. Familiar, M. Nyakato, A. Kutessa, J. Namukooli, T. Ssesanga, C. Joyce, B. Laughton, J. Grab, M. Chernoff, T. Vhembo, L. Fairlie, P. Kamthunzi and M. Boivin (2019). "Building capacity in neurodevelopment assessment of children in sub-Saharan Africa: A quality assurance model to implement standardized neurodevelopment testing." Child neuropsychology : a journal on normal and abnormal development in childhood and adolescence **25**(4): 466-481.

Utter, A. C., R. J. Robertson, D. C. Nieman and J. Kang (2002). "Children's OMNI Scale of Perceived Exertion: walking/running evaluation." Med Sci Sports Exerc **34**(1): 139-144.

ZIMSTAT and UNICEF (2019). Zimbabwe Multi-Indicator Cluster Survey 2019; Snapshots of Key Findings. <https://www.unicef.org/zimbabwe/>.
