# Supplementary material for: Growth, physical, and cognitive function in children who are born HIV-free: School-age follow-up of a cluster-randomised trial in rural Zimbabwe
Source: PLoS Med. 2024 Oct 11;21(10):e1004347. doi: 10.1371/journal.pmed.1004347 (PMC11498706; doi:10.1371/journal.pmed.1004347)
Supplement: S1 Statistical Analysis Plan — (DOCX) [file pmed.1004347.s005.docx]

*Sanitation Hygiene Infant Nutrition Efficacy (SHINE) trial follow-up:*

STATISTICAL ANALYSIS PLAN

Version 2.0

Table of Contents

[1. INTRODUCTION 3](#_Toc111107100)

[2. BACKGROUND 3](#_Toc111107101)

[2.1. Overview 3](#_Toc111107102)

[3. OBJECTIVES 4](#_Toc111107103)

[4. STUDY METHODS 4](#_Toc111107104)

[4.1 Trial Design 4](#_Toc111107105)

[4.2 Randomisation into the SHINE Trial 4](#_Toc111107106)

[4.3 SHINE Follow-up Design 5](#_Toc111107107)

[4.4 Recruitment into SHINE Follow-up 5](#_Toc111107108)

[4.5 Sample Size and Power Considerations 5](#_Toc111107109)

[5. STATISTICAL METHODS 6](#_Toc111107110)

[5.1 General Considerations 6](#_Toc111107111)

[5.2 Missing data 6](#_Toc111107112)

[5.3 Primary and Secondary Analysis 6](#_Toc111107113)

[5.4 Primary modified Intention-to-Treat 7](#_Toc111107114)

[5.5 Adjusted analyses 7](#_Toc111107115)

[5.6 Subgroup analyses 8](#_Toc111107116)

[5.7 GEE Regression Model Specification 8](#_Toc111107117)

[5.8 Detailed outcomes for SHINE Follow-up at 7 years 9](#_Toc111107118)

[5.9 Effect of IYCF and WASH on outcomes (Objectives 1 & 2) 12](#_Toc111107119)

[5.10 Evaluating early-life growth and exposures on school-age function (Objectives 3a, b, c) 13](#_Toc111107120)

[5.11 CHEU and CHU comparison (Objective 3d) 13](#_Toc111107121)

[5.12 Comparison between aspects of school-age growth and function and contemporary conditions (Objective 4) 13](#_Toc111107122)

[5.13 Development of novel outcome metrics (Objective 5) 14](#_Toc111107123)

[5.14 Contemporary factors 15](#_Toc111107124)

[Appendices 16](#_Toc111107125)

[Appendix A: Definitions used in SHINE Follow-up 16](#_Toc111107126)

[Appendix B: Baseline and Follow up Summaries 18](#_Toc111107127)

[Appendix C: Contemporary summary 20](#_Toc111107128)

[References 22](#_Toc111107129)

| **Listing of Tables** | | | |
| --- | --- | --- | --- |
| **Table #** | **Table Title** | **Page** |  |
| 1 | Cognitive outcomes to be measured, including the primary outcome (MPI) and 28 secondary outcomes | 9 |  |
| 2 | Growth and body composition outcomes, totalling 20 secondary outcomes | 10 |  |
| 3 | Physical function outcomes totalling 20 secondary outcomes | 10 |  |
| 4 | Caregiver questionnaire contemporary exposures | 11 |  |

| **Listing of Figures** | | | |
| --- | --- | --- | --- |
| **Figure #** | **Figure Title** | **Page** |  |
| 1 | Conceptual framework of objectives with exposure and outcome variables | 14 |  |

# INTRODUCTION

This document contains the statistical analysis plan for the SHINE Follow-up Study. The goal is to avoid data-driven analyses at the end of the study as much as possible. Much of the content of this SAP is taken directly from the SHINE Follow-up Study Protocol.

# BACKGROUND

### 2.1. Overview

Children are defined as “stunted” when their height-for-age Z-score (HAZ) is >2 standard deviations below the WHO reference standard, but linear growth faltering also affects many children who have not yet fallen below this cutoff. Stunting is associated with increased mortality, poorer school performance, lower adult earnings and long-term chronic disease. Stunting affects 22% (149 million) of children <5 years, while up to 250 million children are at risk of not reaching their developmental potential. This makes stunting a global health priority, although its causes, longer term impact and response to interventions remain poorly understood.

Given the ongoing global burden of stunting, long-term follow-up studies reflecting contemporary conditions, geography and interventions for stunting are urgently needed to inform robust, cost-effectiveness analyses and advocacy of infant and young child feeding (IYCF) and Water, Sanitation and hygiene (WASH) policies and programming. The lack of long-term data evaluating combined neurodevelopmental, physical fitness and growth outcomes has led to a call for further studies that measure a range of outcomes. A holistic measurement is vital to understand school-age trajectories across different functional domains and the impact of risk and protective factors and potential interventions. At school-age, it also becomes easier to undertake more detailed measures of cognitive development, including school performance, executive function and socio-emotional behaviour.

The SHINE Follow-up study aims to re-enrol 1300 children born into the SHINE trial who are now aged 7 years to assess school-aged health, growth, physical and cognitive function using the School Age Health, Activity, Resilience, Anthropometry and Neurocognitive (SAHARAN) toolbox. In addition, information about the child’s prospective health will be collected as well as the derivation of metrics and the application of an annual assessment.

This statistical analysis plan (SAP) was written following the guidelines outlined by Gamble et al.(1) The SAP is designed to give transparency to possible influential statistics decisions for the SHINE follow-up study (PACTR number PACTR202201828512110). The SAP is based on protocol version 28 (October 11th 2021); other objectives outlined in the protocol will be addressed elsewhere. The study principal investigator (Andrew Prendergast) signed off this SAP version 1.0 and co-investigators (Joseph Piper, Melanie Smuk, Robert Ntozini, and Bernard Chasekwa) on 23rd July 2022. The SAP writers are aware of the 18-month outcomes from the SHINE trial(2, 3). While SHINE follow-up is an unblinded study, all contributing members were blind to the school-age participants’ treatment group allocations upon the writing of this SAP.

# OBJECTIVES

1) To evaluate the effects of the randomized IYCF and WASH interventions on school-age growth, body composition, cognitive and physical function,

2) To assess the impacts of stunting and other early-life exposures on school-age growth, body composition, cognitive and physical function, and

3) To examine the differences in school-age growth, body composition, cognitive and physical function between HIV-exposed and HIV-unexposed children.

4) To evaluate the impact of the IYCF intervention on school-age growth, health, physical and cognitive function (both for HIV-exposed and unexposed children),

4) To evaluate the impact of the WASH intervention on school-age growth, health, physical and cognitive function (both for HIV-exposed and unexposed children),

5) To evaluate the relationship between the early-life exposome during the first 1000 days and school-age growth, health, physical and cognitive function, including:

i) Early-life length-for-age Z-score, and categorical definitions of stunting (LAZ<-2) by 1 month of age (early stunting) and by 18 months of age (late stunting),

ii) Environmental factors, including socioeconomic status, household composition, demographics, maternal education, household adversities, food and water insecurity,

iii) Pregnancy exposures including maternal capabilities, maternal nutritional status and depression,

iv) HIV exposure in pregnancy.

6) To evaluate the relationship between current environmental, schooling, nurturing and care-giving practices and school-age growth, health, physical and cognitive function,

7) To develop and deploy 3 novel, holistic and succinct outcome metrics to assess school-age growth, health, physical and cognitive function.

# STUDY METHODS

### 4.1 Trial Design

The SHINE Follow-up study is an extension to the SHINE trial. In brief the SHINE trial was a proof-of-concept, 2x2 factorial, cluster-randomized, community-based trial in two rural districts of Zimbabwe that tested the independent and combined effects of protecting babies from fecal ingestion (Factor 1, operationalized through a WASH intervention) and optimizing nutritional adequacy of infant diet [Factor 2, operationalized through an IYCF intervention] on length-for-age Z score and haemoglobin concentration at 18 months of age, as a primary endpoint(2, 4). Pregnant women were enrolled and they and their infants followed until the children were 18 months of age. A total of 5280 women were enrolled in the trial. The previous objectives of the original SHINE have been published in full(4) (SHINE Trial Team, Clin Infect Dis 2015; available at <https://academic.oup.com/cid/issue/61/suppl_7>).

### 4.2 Randomisation into the SHINE Trial

The SHINE trial used a highly constrained randomisation scheme to allocate clusters to 4 intervention arms. In total 211 clusters were randomized and balanced for key variables. Participants within the trial also had to meet the strict inclusion and exclusion criteria. Detailed information about the SHINE randomisation and inclusion/exclusion criteria can be found in the trial protocol version 28.

The four trial arms were as followed:

1) Standard Care: (“Latrine Later”) (52 clusters)

2) WASH: (“Latrine Now”) (53 clusters)

3) IYCF: (“Latrine Later and Nutributter”) (53 clusters)

4) Sanitation/Hygiene AND Nutrition: (“Latrine Now and Nutributter”) (53 clusters)

### 4.3 SHINE Follow-up Design

The SHINE Follow-up study aims to revisit 1300 children born into the SHINE trial who are now aged 7-years residing within Shurugwi District only (Visits to Chriumanzu District will only be performed if there is significant under-recruitment). The children’s school-aged health, growth, physical and cognitive function will be assessed using the School Age Health, Activity, Resilience, Anthropometry and Neurocognitive (SAHARAN) toolbox. In addition, information about the child’s prospective health will be collected as well as the derivation of metrics and the application of an annual assessment. For more detail on the SHINE Follow-up design, see protocol version 28.

### 4.4 Recruitment into SHINE Follow-up

The 1300 children in Shurugwi district will be based in the following sub-cohorts:

- 1000 HIV-unexposed (CHU) children, from the 4 SHINE trial arms (approx. 250 in each arm)
- 300 children HIV-exposed and uninfected (CHEU), i.e. children born to HIV positive mothers but who remain HIV-negative. These children will be from all four arms of treatment, although it is expected there will be more children in the WASH and WASH+ IYCF treatment arms, because these arms were enrolled earlier into the original trial.

Children will be randomly selected from the SHINE trial database and will be included if they meet the following criteria:

1. Enrolled in the SHINE trial and had an 18-month endline visit;
2. Still living in rural Shurugwi (including those who have moved but stayed within Shurugwi District and also children who return to Shurugwi for school holidays);
3. Age 7 years at the time of enrolment (up to but not including 8 years);
4. Have a primary caregiver who is willing to provide written informed consent;
5. Maternal pregnancy HIV status known.

More detail on recruitment and sensitisation can be found in the study protocol version 28.

### 4.5 Sample Size and Power Considerations

The Kaufmann Assessment Battery for Children (KABC-II) is a neurocognitive test battery that provides an overall measure of cognitive processing. Subtests are stratified by age into 4-month blocks, to give an overall composite score, termed the Mental processing index (MPI). The MPI is the primary outcome for this study and forms the basis for the sample size calculation: 1000 children (500 IYCF vs 500 non-IYCF) will be assessed, providing 86% power to detect a 0.2 standard deviation difference in KABC-II total neurodevelopmental score (MPI) between combined arms, with alpha 0.05, assuming intra-cluster correlation of 0.05 and sampling from 100 clusters. An intra-cluster correlation coefficient of 0.05 will be used as SHINE follow up at 7 years is only from one of the two original districts (Shurugwi). A 0.2 SD difference in MPI will allow detection of the difference in IQ scores observed at 3-7 years of age among children followed-up in the INCAP study, and is also the approximate magnitude of socio-emotional difference recently shown with a similar SQ-LNS trial(1).

Within the randomized trial design, we aim to measure 300 CHEU across all arms, which will give us a large scope to explore intervention effects, similar to what was previously observed(2) (3).

# STATISTICAL METHODS

### 5.1 General Considerations

Except for specialized analyses, Stata 14.0 or later will be used for conducting study analyses. Reporting of results will follow the guidelines established in the extended CONSORT guidance for cluster-randomized trials(4).

### 5.2 Missing data

Observations with missing outcomes variables will be omitted from analysis. All analyses will be complete case analysis if the proportion missing in each covariate is ≤10%. Continuous covariates with >10% missing will be omitted from the analysis. A dummy category will be created for the missing values in categorical covariates with >10% missing. Multiple imputation methods will be used in sensitivity analyses.

### 5.3 Primary and Secondary Analysis

The SHINE Follow-up study has one primary and five secondary analyses. The primary analyses are to:

- Explore the difference in KABC-II neurodevelopmental score within HIV-unexposed children between the combined arms (IYCF and IYCF / WASH) versus the combined arms (SOC and WASH). We call this the IYCF v non-IYCF comparison.

The secondary analyses are to:

- Explore the difference in KABC-II neurodevelopmental score within HIV-unexposed children between the combined arms (B and D) versus the combined arms (A and C). We call this the WASH v non-WASH comparison. (Given there was no effect of WASH intervention on growth)
- Explore the difference in KABC-II neurodevelopmental score within CHEU for IYCF v non-IYCF comparison.
- Explore the difference in KABC-II neurodevelopmental score within CHEU for WASH v non-WASH comparison.
- Explore the difference in other SAHARAN tests between IYCF vs non-IYCF and WASH vs non-WASH for HIV-unexposed children
- Explore the difference in other SAHARAN tests between IYCF vs non-IYCF and WASH vs non-WASH for CHEU

### 5.4 Primary modified Intention-to-Treat

These analyses are based on children born to women who were randomized to interventions within the SHINE trial. It covers data collected from consent to participate in the study whilst pregnant, up to the child’s 7-year visit. Residence at time of consent determines study arm for these analyses. Loss to follow up, in terms of the CONSORT diagram, is anything that keeps a child’s 7-year SHINE Follow-up visit from being measured—refusal, child death, or a permanent move outside Shurugwi are expected to be among the main reasons. This analysis compares SAHARAN Toolbox outcomes across arms according to the mother’s assignment at enrollment, among all those born alive, whose mother’s pregnancy HIV status is known and who were randomized into selection. For sensitivity analyses, children born to women who were HIV-negative during pregnancy but HIV-positive at a subsequent postnatal visit (i.e. seroconverters) will be removed.

Although the study is not powered to detect a statistical interaction between the IYCF and WASH interventions, it will be estimated. For each domain, we will test for interaction with one key pre-defined outcome: (For Cognition it is MPI, which is our primary outcome, for physical function it is handgrip strength, and for growth it is Height-for-age Z-score at 7 years, HAZ). The interaction in the model will be defined as significant (p < 0.1, according to the Wald test) or with a sizeable point estimate (i.e., difference in mean score > 0.25 SD for continuous outcomes; RR > 2 or <0.5 for dichotomous outcomes). Otherwise, we will use a regression model with 2 terms to represent the treatment arms; we will estimate the effect of IYCF by comparing the 2 IYCF arms with the 2 non-IYCF arms and will estimate the effect of WASH by comparing the 2 WASH arms with the 2 non-WASH arms. If interaction is significant, we will use a regression model with 3 terms to represent the 4 intervention arms for all outcomes in that domain(5).

### 5.5 Adjusted analyses

For the primary and secondary outcomes, besides the main unadjusted analyses, we will conduct

analyses adjusted for baseline covariates (and clustering, in a GEE model as described) in order to account for residual confounding and/or improve power. The variables to be considered for inclusion are (these have data on >90%, or use a category that includes Missing):

#### Maternal

Age

Height

MUAC

Years of completed schooling

Marital status

Parity

Employment status

Religion (3 dummies for Apostolic, Other Christian, and Other religion, no religion or missing)

Maternal capabilities

Maternal hemoglobin

CD4 count and PMTCT exposure during pregnancy (HIV-infected women only)

Maternal dietary diversity score

#### Household

HH Coping Strategies Index - HH receives food aid from government or other organization

Any latrine

Improved latrine

Open defecation

Improved floor

Time to drinking water

Household dietary diversity score

Household size

Wealth score from 16 key variables, and wealth quintile (5 dummies for the 5 quintiles and Missing)

#### Infant

Low birthweight (2 dummies for <2500g, >=2500g, Missing)

Gender

Preterm

#### Study

Data collector (only for outcomes relying on measured infant length or weight or early child development assessment)

Calendar quarter of study (dummies)

### 5.6 Subgroup analyses

For the primary and secondary outcomes, a subgroup analysis will be performed by child sex. Statistical interaction terms will be evaluated to look for differential intervention impact in these subgroups.

### 5.7 GEE Regression Model Specification

All regression models will be adjusted for clustering using SHINE clusters.

Models estimating mean difference and relative risk (RR) by treatment arm - main effect WASH and IYCF on continuous outcome Y:

*xtgee Y i.wash i.iycf [covariates], family(gaussian) corr(exchangeable) vce(robust)………….…….……………………………………………………………..…(1)*

*xtgee Y i.wash i.iycf [covariates], family(log) corr(exchangeable) vce(robust) eform………..……………………………………………………………………....………(2)*

Models estimating mean difference and RR by HEU status on continuous and binary outcome Y:

*xtgee Y i.HEU [covariates], family(gaussian) corr(exchangeable) vce(robust)………….…(3)*

*xtgee Y i.HEU [covariates], family(log) corr(exchangeable) vce(robust) eform……………(4)*

Regression models testing for intervention arm WASH and IYCF interaction:

*xtgee Y i.HEU i.armwash ##i.armiycf,* *family(gaussian) corr(exchangeable) vce(robust)………..………………………………..............................................……..(5)*

*xtgee Y i.HEU i.armwash ##i.armiycf,* *family(log) corr(exchangeable) vce(robust) eform………..………………………………..............................................……….…..(6)*

Covariates used to adjust models will include trial factors, plus socioeconomic and demographic confounders, defined as baseline factors which are univariably associated with both HIV exposure (p < 0.05), univariably associated with the neurodevelopmental outcome (p < 0.05), and not likely to be on the causal pathway leading from HIV exposure to neurodevelopmental outcomes.

### 5.8 Detailed outcomes for SHINE Follow-up at 7 years

The detailed outcomes of the main 7 year follow-up using the SAHARAN toolbox are shown below. Details of analysis approach are shown in section 9. As mentioned previously, the primary outcome of the long-term follow-up study is cognitive function, assessed by the mental processing index (MPI) - the total score from 8 subtests of the Kaufmann Assessment Battery for Children 2^nd^ edition (KABC2). The subtests measure four domains of cognitive processing across learning, planning, simultaneous and sequential memory. All other outcomes are secondary outcomes and shown in Table 1.

**Table 1:** Cognitive outcomes to be measured, including the primary outcome (MPI) and 28 secondary outcomes.

|  | **MARKER** | **MEASURE** | **Outcomes** | **RATIONALE** |
| --- | --- | --- | --- | --- |
| **Cognitive**  **Function**  **(120 mins including KABC-II)** | **Kaufmann Assessment Battery for Children** | **Cognitive processing** | **Primary outcome: Mental processing index (MPI)** | **Overall measure of cognitive function** |
|  |  |  | Secondary outcomes:  KABC-II domain scores, individual subtest scores | Short & long-term memory, planning, problem-solving, sequential memory |
|  | **School Achievement Test** | Academic | Total score, Subtest scores (*numeracy, reading, writing)* | Literacy & numeracy |
|  | **Fine motor** | Shortest time to complete finger tapping sequence | Time for dominant hand, non-dominant hand, and average between both hands, | Fine motor |
|  | **Plus-EF Tablet test** | Executive Function | Overall score, individual subtest scores, reaction time | Executive function |
|  | **Child socio-emotional questionnaire** | Home support | Total score, sub-score removing food insecurity question | Child’s own perspective on home support |
|  | **Washington Group (WG) Child function module (asked in caregiver questionnaire)** | Disability screening, including vision and hearing | Overall score, Disability, learning, mental health subscale | Child functional abilities |
|  | **Strength and Difficulties Questionnaire (SDQ) (asked in caregiver questionnaire)** | Socioemotional function | SDQ total score and subtest scores | Behaviour |

**Table 2:** Growth and body composition outcomes, totalling 20 secondary outcomes.

|  | **MARKER** | **MEASURE** | **Outcomes (secondary)** | **RATIONALE** |
| --- | --- | --- | --- | --- |
| **Body composition (20 mins)** | **BIA** | Impedance of tissues | Lean mass index (LMI),  Phase angle, Impedance index (ImpI), Reactance, Resistance | Quality of growth, metabolic health |
|  | **Knee-heel length** | Tibial growth | Median Knee-heel length | Prioritization of growth |
|  | **Triceps, scapular, suprailiac, calf skinfolds** | Subcutaneous fat | Sum of skinfolds, Individual skinfolds,  Peripheral: central skinfolds, | Subcutaneous fat: peripheral c.f. central, metabolic health |
| **Anthropometry (15 mins)** | **Height,**  **weight** | Growth | Height for age Z-score (HAZ), Weight-for age Z-score (WAZ), Body mass index (BMI) | Growth, nutritional status, Metabolic health |
|  | **Head circ** | Brain volume | Head circumference (headcirc) | Prioritization of growth |
|  | **Waist circ, Hip circumference** | Abdominal size | Waist circumference, hip circumference | Nutritional status, metabolic health |
|  | **Calf circ, MUAC** | Peripheral fat & muscle | Calf circumference (calf circ), Mid upper arm circumference (MUAC) | Quality of growth |

**Table 3:** Physical function outcomes totalling 20 secondary outcomes

|  | **MARKER** | **MEASURE** | **Outcomes (secondary)** | **RATIONALE** |
| --- | --- | --- | --- | --- |
| **Physical**  **Function (30 mins)** | **Grip strength (a)** | Lean muscle both hand | Highest grip strength**,**  Standardised grip strength **(a)**  Dominant and non-dominant hand strength, | Lean muscle: hand |
|  | **Broad jump (b)** | Truncal muscles | Maximum distance,  standardised distance (**b)** | Lean muscle: leg |
|  | **20m Beep test (c)**  **(composite score = standardised a+b+c)** | Physical Fitness, | Shuttle run test level (SRT)  Standardised shuttle run test level: **(c)**  ***Composite standardised score =***  ***a+b+c***  Heart rate (HR) variability, HR: level ratio, drop in HR after 1 minute recovery, baseline HR, highest HR, final HR after test | Stamina,  Overall composite score |
|  | **Haemoglobin** | Anaemia | Hb | Physical fitness |
|  | **BP** | Fitness | Resting Systolic & diastolic bp, Pulse pressure, systolic & diastolic BP & pulse pressure 1 minute after exercise; Post-exercise difference between 1^st^ and 5^th^ BP systolic & diastolic measurement | Cardiovascular fitness |

**Table 4:** Caregiver questionnaire contemporary exposures: (EPDS: Edinburgh Postnatal Depression Score, HDDS: Household dietary diversity score, FCS: Food consumption score, H-FOOD: holistic food insecurity, HWISE: Household water insecurity experiences scale, H-WATER: holistic water insecurity). Note: WG & SDQ sections are included in cognitive outcomes.

|  | **QUESTIONNAIRE** | **DOMAIN** | **Measures** | **RATIONALE** |
| --- | --- | --- | --- | --- |
| **Caregiver questionnaire (90 mins)** | **Demographics** | Household (HH) composition | Main caregiver, caregiver years of schooling, religion, head of HH, breastfeeding duration | Nurturing, HH demographics, caregiver education |
|  | **Socioeconomic status** | SES score | Overall score | Socio-economic status |
|  | **Schooling & COVID impact** | School engagement & attendance | Years & months of schooling, Attendance,  Alternative learning (if not in school), books at home | Child Education |
|  | **Child adversity scale** | Adversities | Overall score (note different weightings may be applied) | Measure of accumulated adversities |
|  | **Child parent relationship scale** | Caregiver’s relationship with child | Overall score,  Closeness, conflict subscales | Nurturing |
|  | **MICS Child discipline score** | Caregiver’s relationship with child | Overall score,  accepting of violence (yes/no) | Nurturing |
|  | **EPDS** | Maternal depression | Overall score, ?definition of depression, | Depression |
|  | **Gender norms** | Caregiver gender norms | Overall score, sub-score on specific questions (education & violence)  (better to send a son than daughter to school) yes/no | Maternal capabilities |
|  | **Social support** | Caregiver social support | Overall score | Maternal capabilities |
|  | **HFIAS, HDDS, FCS** | Food insecurity & dietary diversity | HDDS score  HFIAS score  FCS score | Food insecurity |
|  | **H-FOOD** | Land use | Land use, food aid, other adversities | Food insecurity |
|  | **HWISE, Water access, H-WATER** | Water insecurity & access | HWISE score,  water volume, water usage | Water insecurity |
|  | **HIV** | HIV status | ARV treatment,  Date of diagnosis, |  |

### 5.9 Effect of IYCF and WASH on outcomes (Objectives 1 & 2)

We will capitalize on the 2x2 factorial trial design to evaluate the IYCF and WASH interventions as two trials run in the same population, stratified by maternal HIV status. For the analysis of IYCF as the primary outcome, we will therefore combine the two IYCF-containing trial arms (IYCF alone, and IYCF+WASH) and compare them to the two non-IYCF arms (WASH alone, and standard-of-care). For the analysis of WASH as a secondary outcome, we will combine the two WASH-containing trial arms (WASH alone, and IYCF+WASH) and compare them to the two non-WASH arms (IYCF alone, and standard-of-care). The effect of IYCF and WASH on each outcome (as defined in Tables 1 to 3) will be estimated using generalized estimating equations (GEE) with an exchangeable working correlation structure to account for within-cluster correlation, assuming no interactions between randomized interventions. All analyses will account for the nature of the distribution of the outcome and results will be presented as appropriate effects sizes (mean difference between groups and risk ratios) with a measure of precision (95% confidence intervals). Analyses will be by intention-to-treat at the child level, according to the mother’s assigned study arm based on her residence at the time of her enrolment into SHINE, regardless of subsequent moving or adherence to the interventions.

The primary analysis will be unadjusted. A secondary adjusted analysis will account for original trial stratification factors and other variables including exact decimal age, gender, socio-economic status, maternal depression score, study nurse and adversity index. Since we are performing a large number of tests, we will base any conclusions on patterns observed in outcome groupings, as opposed to relying on P-values. In addition, we will consider use of a more formal adjustment to account for the multiple testing such as Bonferroni correction. Loss to follow-up from the original trial population due to inability to trace or unwillingness to join the long-term assessment will be assessed, and if there is any evidence of loss to follow-up by the original allocation, we will consider using inverse probability weighting to account for this in the analysis.

### 5.10 Evaluating early-life growth and exposures on school-age function (Objectives 3a, b, c)

As secondary outcomes, we will explore the effects of early stunting (by 1mo of age) and late stunting (by 18mo of age) on each outcome at 7 years of age, and the effect of birthweight and LAZ as a continuous variable at 1 and 18 months. For the early-life exposome, we will follow recommended approaches for large datasets, and use principal components analyses to first reduce the number of exposure variables into a smaller set of functional biological domains that best represent the most informative combinations of exposures. We will then use appropriate techniques to quantify the effects of pregnancy and baseline exposures on school-age outcomes, exploiting the rich SHINE dataset to control for confounding by pregnancy-related factors (e.g. wealth, maternal education, haemoglobin, maternal capabilities), the trial interventions, and the time-varying nature of exposures experienced between birth and 18 months of age.

### 5.11 CHEU and CHU comparison (Objective 3d)

Baseline characteristics between CHEU and children HIV-unexposed (CHU) groups will be compared using multinomial and ordinal regression models and Somers’ D for medians, while handling within-cluster correlation with robust variance estimation. We will use GEE models to compare each functional outcome, assessed using the SAHARAN Toolbox, between CHEU and CHU groups. In addition, we will compare CHEU and CHU using the reduced school-age outcomes and early-life exposures outlined above. A secondary adjusted analysis will be performed using exact decimal age, gender, socio-economic status, maternal depression score, study nurse and adversity index. Poisson regression will be used to compare the cumulative incidence of illness episodes, clinic visits, and hospitalizations reported between groups during prospective surveillance measured by the community health worker illness questionnaire.

### 5.12 Comparison between aspects of school-age growth and function and contemporary conditions (Objective 4)

Internal consistency of outcomes between cognitive functional domains will be compared, including the primary outcome (KABC-II total score) and other cognitive measurements. Similarly, consistency of physical function measurements and their associations with growth will also be measured, as previously described(19). Univariable analysis will explore associations between school-age growth, cognitive and physical function (Tables 1 to 3) and current environmental, schooling, nurturing and care-giving exposures (Table 4). We will also undertake an exploratory factor analysis to identify the most informative combinations of measures from the SAHARAN Toolbox. To guide biological interpretation and identify relevant functional domains from the results of the data-reduction step, we will explore factor loadings using bi-plots. In the case of heavily cross-loaded results, we will consider sparse data reduction methods, such as sparse principal components analysis, to facilitate interpretation of factor loadings. We will also apply hierarchical clustering of principal component scores to group children according to their school-age outcomes. We will explore univariable distributions (central tendencies, variance, prevalence) of school-age outcome variables within each identified cluster of children to gain further insights into trade-offs and prioritisation in growth and function(6).

### 5.13 Development of novel outcome metrics (Objective 5)

Novel outcome metrics will be developed based on SAHARAN toolbox measurements conducted in 250 children in the SHINE control arm. We will use these data to undertake a factor analysis of the primary (and 28 secondary) cognitive SAHARAN variables to select the most informative sparse combination of tests for development of a cognition metric (COG-SAHARAN). We will undertake reliability and agreement analyses to compare the performance of the shortened, open-access tools with the KABC-II tool, which is a gold-standard assessment, but is costly, time-consuming, complex and proprietary. A similar factor and reliability analysis will inform the generation of the growth metric (GROW-SAHARAN) from 40 growth and physical function outcomes. GROW-SAHARAN will measure the child’s growth, body composition and physical function to holistically assess school-age children’s nutritional status. Further analyses could tailor the GROW-SAHARAN to focus on key indicators within specific areas such as physical function, body composition or chronic disease risk. This is a sufficient sample size to derive the COG-SAHARAN and GROW-SAHARAN metrics, based on previous published metrics(7, 8). As outlined above, we will employ factor analysis as a data reduction step to i) identify the underlying constructs that we are measuring, ii) evaluate which of the multiple sub-tests are driving the variability measured, and iii) determine whether all tests are required.

Standardisation exercises with the same children will allow us to test the reliability of the proposed measures using intra-cluster correlation coefficients and to measure Cronbach’s alpha. Finally, the relationship between these novel metrics will be investigated within a recognised conceptual framework that evaluates child function(9) (Figure 1). A combined child metric (“SUB-SAHARAN”) will also be developed after derivation of COG- and GROW-SAHARAN, by exploring the relationships between the two metrics. All metrics will then be operationalised to the 1000 CHU randomized to early-life IYCF or WASH interventions, and the metrics’ performance compared to the more detailed analysis outlined in previous sections.


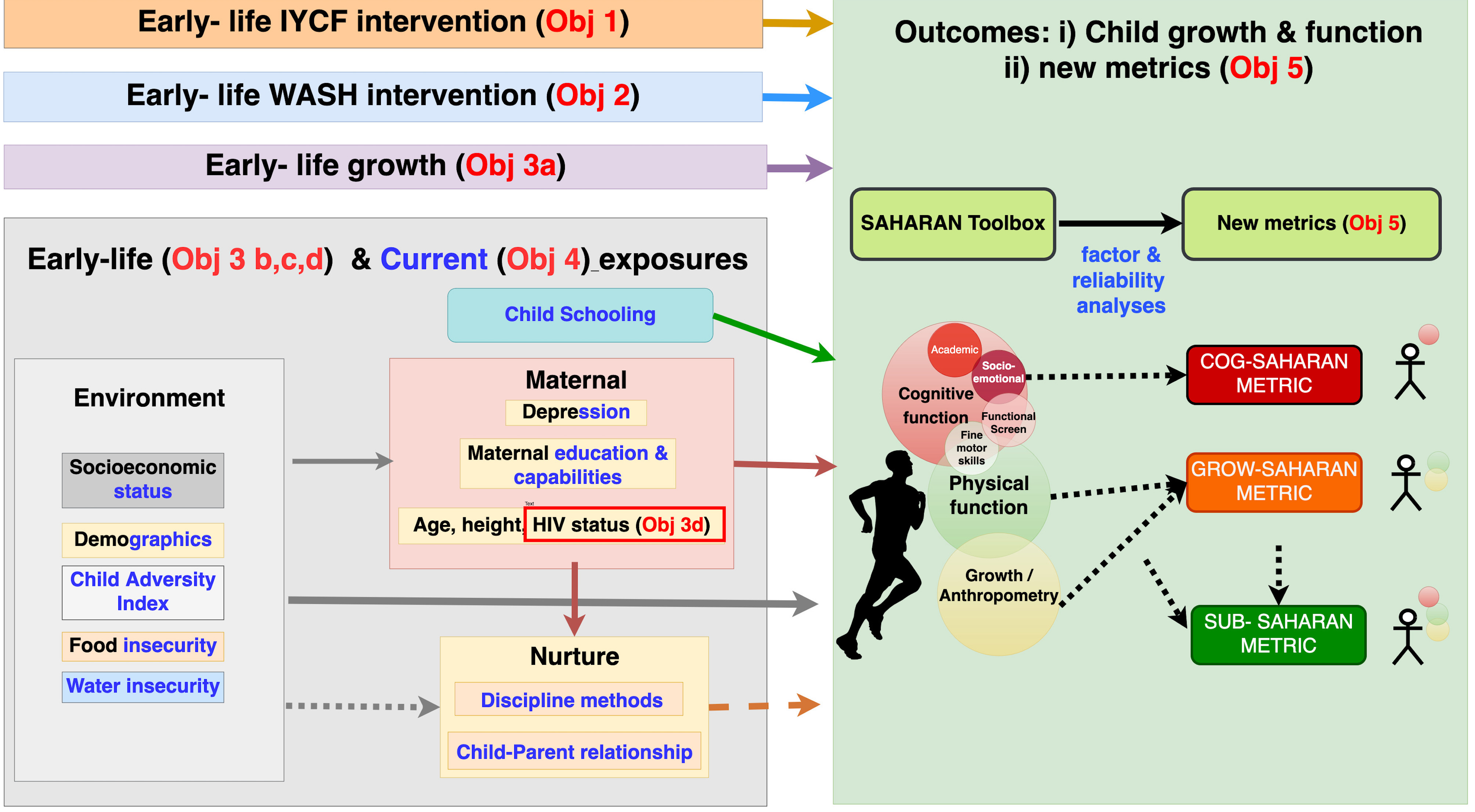


**Figure 1:** Conceptual framework of objectives with exposure and outcome variables. Exposures are split into environmental, schooling, maternal and nurturing domains. Early-life exposures are described in black and contemporary exposures in blue text. Those exposures that were measured in both early-life and contemporary are written in black and blue text. Outcomes are based on the SAHARAN toolbox to provide school-age child growth, health and function. These outcomes will also be analysed in the standard of care arm to provide new cognitive (COG-SAHARAN), Growth (GRO-SAHARAN) and overall (SUB-SAHARAN) metrics. Image from https://openclipart.org/detail/266681/jogging-man-silhouette

### 5.14 Contemporary factors

In addition, adjustment may be made if there is a significant imbalance between the intervention groups. Potential significant imbalance would be checked in contemporary environmental factors including socioeconomic status, food (HDDS, FCS, HFIAS) and water insecurity (HWISE, H-WATER) measures and maternal capabilities (gender norms and social support measures).

For each outcome, each of the above covariates will be assessed in a bivariate analysis, and those covariates that meet a minimum requirement will be entered into multivariable analyses. A minimum requirement for dichotomous outcomes is: p<0.2 or RR>2.0 or <0.5. For continuous outcomes: p<0.2 or difference >0.25 SD. For these analyses, dichotomous (cut at the median) versions of continuous covariates will be used, but then if selected, the continuous version will be used in the next stage. For covariates represented by multiple dummy variables, only the overall Wald test will be used. The exception is the Data collector (DC) variable: it will be assessed via a likelihood ratio test in a random effects model with random intercepts for cluster and for DC. If p<0.2, then a GEE model with DC represented by dummies will be run, and the DCs with the 10 furthest-outlying estimates will be entered into the next stage (to then be represented by 9 dummies).

The next stage will consist of a backward stepwise selection procedure, with p>0.2 to remove, and p<0.2 to remain.

Further exceptions may be made when there are insufficient data for stable estimation of coefficients, which may occur in subgroup analyses or for relatively rare outcomes. For continuous outcomes, we will ensure there are at least 5 outcome measures for every parameter to be estimated. For dichotomous outcomes, we will ensure there are at least 10 events (prevalent conditions) for every parameter to be estimated. If these conditions are not met, the first covariates to be dropped are DC, then calendar quarter, then the next least statistically significant covariates.

# Appendices

### Appendix A: Definitions used in SHINE Follow-up

A number of parameters will be assessed in this trial that could be measured or defined in a number of ways. Table 5 lists definitions that will be used in the study. Some of these may be subject to change if more accurate or less costly approaches to measurement become available during the study period.

| **Parameter** | **Definition/Assessment Method** |
| --- | --- |
| Child anemia | Haemoglobin < 110 g/L as assessed using the Hemocue hemoglobinometer |
| Child severe anemia | Haemoglobin < 70 g/L as assessed using the Hemocue hemoglobinometer |
| Low birth weight | Infant born weighing <2500 g within 72 hours following delivery |
| Preterm birth | Infant born prior to completing 37 weeks gestation |
| Small for gestational age | Infant with weight at birth less than the 10th percentile of Intergrowth fetal/newborn growth standards. |
| Stunted for a given age  Severely stunted for a given age | LAZ < -2 Z-score (WHO 2006)  LAZ < -3 Z-score (WHO 2006) |
| Nutributter supplementation | Distribution measures and maternal report of feeding the index child and not sharing with other children |
| Social support for mothering | Assessed using questions adapted from published instruments (Cohen 1985; Sherbourne 1991) and pilot-tested in Shona, comprising material, informational, and emotional support. |
| Maternal access/control of resources | Questions adapted from Gates-Funded Alive & Thrive Project survey, and pilot-tested in Shona |
| Maternal depression | Assessed with the Edinburgh Postnatal Depression Scale, which has been validated in Shona with cut-offs defined by Chibanda *et al.* |
| Maternal stress | Elevated salivary cortisol concentration |
| Maternal roles, priorities and time | Novel set of questions developed and pilot-tested in Shona by our team |
| Maternal perceived physical health | Assessed using an adaption of the SF-36 |
| Mothering self-efficacy | Assessed using questions adapted from published instruments (Dumka 1996; Gilmore 2009) and pilot-tested in Shona |
| Fidelity of intervention delivery | Extent to which study interventions are delivered as designed/intended. Assessed through review of supervision and CHW records to establish timing of CHW visits and adherence to implementation protocols. |
| Child functioning, disability and behavioural questionnaire | Measurement of disability and behavioural problems as provided by the Washington group – categorical – reported within domains (note those with ‘a lot of difficulty’ or ‘cannot do at all’ for vision, hearing, walking or learning will be excluded from the ECD assessment). |
| Home environment (FCI) | Family Care Inventory (continuous measure in 3 sections – Measure of the amount of stimulation the child has in the home. |
| Maternal Child Interaction | Observation of Maternal Child Interaction |
| Observation of behaviour of child and mother | Observational tool from Bayley III (continuous score) |
| School-aged cognitive function (Mental processing index) | Assessed using the Kaufmann Assessment Battery for Children, 2^nd^ Edition (KABC-II) which measures general cognition, memory, spatial ability and reasoning. Note the following 8 KABC-II subtests will be in the main study, and are highlighted in bold below: Number Recall, Word Order, Rover, Triangles, Pattern Reasoning, Story Completion, Atlantis, Atlantis delayed |
| Plus-EF (executive function) | The android based PLUS- EF tool(10) for executive function tasks measures child executive function. For 7 year olds, 3 tests are used: Multi-source interference test (MSIT), Hearts and Flowers and Flanker. [Digit Scan Backwards was found to be advanced for 7 year olds in the pilot so is omitted]. |
| School-aged numeracy and literacy | Assessed using the Word, Reading, Arithmetic test. This test gauges early numeracy skills through a 25-minute individual assessment that includes basic counting, addition, subtraction and number patterns. It also includes literacy scores by reading individual letters, syllables and words. Finally it assesses literacy through writing of letters, words and the child’s own name. |
| School-aged fine motor function | Assessed using timed sequential finger tapping, which was previously shown to be slower in children with stunting(11). |
| School-aged growth and body composition | Growth assessed by anthropometry including height, weight, head circumference, abdominal, upper arm, hip and calf circumferences. Body composition assessed by knee-heel length, skinfold thickness measurements and bioimpedance analysis (BIA). Knee-heel length measures from the knee to the bottom of the ankle as a sensitive measure of growth. Skinfold thickness measures the subcutaneous fat layer around the body. BIA measures the proportion of lean mass using an imperceptible electrical signal between the hand and foot. |
| School-aged physical function | Assessed by measuring handgrip strength, how far the children can jump in the broad jump and also how far and fast they can run in the beep test. During the beep test, their heart rate and blood pressure will also be measured and compared to resting values. |
| School-aged overall child function | Assessed using the UNICEF/Washington Group Child Functioning module, which highlights disability. |
| School-aged socio-emotional development | Assessed using the Strength and Difficulties Questionnaire (SDQ). These are caregiver questionnaires that screen for socio-emotional symptoms in children by performing a detailed behaviour assessment. |
| Schooling and homework | Assessed by a caregiver questionnaire including school enrolment, and periods of absence, available books for the child and help with homework. Further questions will also assess the impact of COVID-19 precautions on school enrolment and schooling support for children at home. |
| School-aged adversities index | Assessed by a caregiver questionnaire that asks for major life adversities associated with reduced child development since birth, including health. This includes the Child Parent Relationship scale (CPRS) which measures the caregiver’s view of their relationship with their child. |
| School-aged food and water insecurity | Assessed using the Household Food Insecurity Access Scale (HFIAS) and Household Dietary diversity score (HDDS). Further food insecurity questions will be asked using the Holistic Food insecurity scale (H-FOOD) and Food Consumption score (FCS). For water insecurity, the Household Water Insecurity Experience Scale (HWISE) will be used and also the Holistic water insecurity scale (H-WATER). |
| School-aged water access | Assessed by questionnaire on type of water point and time to walk for both drinking and household uses. |
| School-aged child discipline | Assessed by a caregiver questionnaire that asks on use of discipline. This was previously used in 2019 across Zimbabwe in the UNICEF Multi-Indicator Cluster Survey (MICS6)(12). |
| Children HIV unexposed (CHU) | Children whose mother tested HIV negative during pregnancy |
| Children HIV exposed but uninfected (CHEU) | Children whose mother tested HIV positive during pregnancy, but the child tested HIV negative |
| Seroconversion | Mother who tested HIV-negative during pregnancy and subsequently tested HIV positive |

### Appendix B: Baseline and Follow up Summaries

| **Summary: by each analysis set, where relevant, and by study arm** |
| --- |
| Number of participants enrolled |
| Status of enrolled participants—both mothers and children—at Study Visits: Birth, End of pregnancy, 3, 6, 12, 18, 24 months post-partum (status: evaluated; dropped out; lost to follow up; dead) |
| Demographics and Baseline Characteristics:  Maternal age (mean, SD)  Maternal height (mean, SD)  Maternal MUAC (mean, SD)  Maternal haemoglobin (mean, SD)  Years of schooling (median, IQR)  Maternal parity (mean, SD)  Marital status, % married  Employment status, % employed  Percentage mothers meeting minimum dietary diversity  Religion – distribution into categories: apostolic, other Christian, other religion)  Mean Edinburgh postnatal depression scale  Percentage of households meeting minimum dietary diversity score  Wealth index – distributed into quintiles  Coping strategies index, median (IQR)  Electricity in home  Improved latrine at household at baseline  Main source of household drinking water improved  Household size, median, IQR  Main caregiver, percentage mother  Infant sex, percentage female  Infant birthweight, mean (SD)  Low birth weight, percentage <2500g  Gestational age, mean (SD)  Preterm, percentage <37 weeks  Delivery place, percentage institutional delivery  Delivery mode, percentage vaginal delivery  For CHEU only:  Documented ART use during pregnancy  Maternal ARV regimen, Tenofovir disoproxil fumarate-based ART regiment  Zidovudine-based ART regimen  Documented co-trimoxazole prophylaxis during pregnancy  Maternal CD4 count (if available) |

### Appendix C: Contemporary summary

| **Summary: by each analysis set, where relevant, and by study arm** |
| --- |
| Number of participants enrolled |
|  |
| Contemporary Demographics and Characteristics:  Main caregiver, percentage mother  Primary caregiver age (mean, SD)  Primary caregiver height (mean, SD)  Years of schooling (median, IQR)  Maternal capabilities: gender norms & Social support  Religion – distribution into categories: apostolic, other Christian, other religion)  Mean Edinburgh postnatal depression scale  Percentage meeting (HDDS)  Wealth index – total, distributed into quintiles  Household Food Insecurity Assessment index (HFIAS), median (IQR)  Household Water insecurity experiences scale (HWISE)  Improved floor, percentage  Time to drinking water, median, IQR  Electricity in home  Improved latrine at household at baseline  Main source of household drinking water improved  Treat water, percentage  Household size, median, IQR  Number of children, number of adults  Household adversity scale for past 7 years  Household death, unemployment  Crop failure  Loss of land or possessions  Alcohol problems  Debt  Child Parent Relationship Scale (CPRS)  Measures of Discipline total  Years of schooling  Duration of breastfeeding  For CHEU only:  Main caregiver, percentage mother  % Currently on ARV’s  Current maternal ARV regimen |

# References

1. Ocansey ME, Adu-Afarwuah S, Kumordzie SM, Okronipa H, Young RR, Tamakloe SM, et al. Prenatal and postnatal lipid-based nutrient supplementation and cognitive, social-emotional, and motor function in preschool-aged children in Ghana: a follow-up of a randomized controlled trial. The American journal of clinical nutrition. 2019;109(2):322-34.

2. Chandna J, Ntozini R, Evans C, Kandawasvika G, Chasekwa B, Majo F, et al. Effects of improved complementary feeding and improved water, sanitation and hygiene on early child development among HIV-exposed children: substudy of a cluster randomised trial in rural Zimbabwe. BMJ Glob Health. 2020;5(1):e001718.

3. Ntozini R, Chandna J, Evans C, Kandawasvika G, Humphrey JH, Prendergast AJ. Impact of improved nutrition/sanitation on neurodevelopment of HIV-exposed children. Conference on Retroviruses and Opportunistic Infections (CROI); March 3rd-7th; Seattle2019.

4. Campbell MK, Piaggio G, Elbourne DR, Altman DG. Consort 2010 statement: extension to cluster randomised trials. BMJ : British Medical Journal. 2012;345:e5661.

5. Gladstone MJ, Chandna J, Kandawasvika G, Ntozini R, Majo FD, Tavengwa NV, et al. Independent and combined effects of improved water, sanitation, and hygiene (WASH) and improved complementary feeding on early neurodevelopment among children born to HIV-negative mothers in rural Zimbabwe: Substudy of a cluster-randomized trial. PLOS Medicine. 2019;16(3):e1002766.

6. Prendergast AJ, Szubert AJ, Berejena C, Pimundu G, Pala P, Shonhai A, et al. Baseline Inflammatory Biomarkers Identify Subgroups of HIV-Infected African Children With Differing Responses to Antiretroviral Therapy. J Infect Dis. 2016;214(2):226-36.

7. Mundfrom DJ, Shaw DG, Ke TL. Minimum Sample Size Recommendations for Conducting Factor Analyses. International Journal of Testing. 2005;5(2):159-68.

8. MacCallum RC, Widaman KF, Zhang S, Hong S. Sample size in factor analysis. Psychological Methods. 1999;4(1):84-99.

9. Prado EL, Abbeddou S, Adu-Afarwuah S, Arimond M, Ashorn P, Ashorn U, et al. Predictors and pathways of language and motor development in four prospective cohorts of young children in Ghana, Malawi, and Burkina Faso. Journal of Child Psychology and Psychiatry. 2017;58(11):1264-75.

10. Obradović J, Sulik MJ, Finch JE, Tirado-Strayer N. Assessing students' executive functions in the classroom: Validating a scalable group-based procedure. Journal of Applied Developmental Psychology. 2018;55:4-13.

11. Chang SM, Walker SP, Grantham‐Mcgregor S, Powell CA. Early childhood stunting and later fine motor abilities. Developmental Medicine & Child Neurology. 2010;52(9):831-6.

12. ZIMSTAT, UNICEF. Zimbabwe Multi-Indicator Cluster Survey 2019; Snapshots of Key Findings. <https://www.unicef.org/zimbabwe/>; 2019.
