# Supplementary material for: Growth, physical, and cognitive function in children who are born HIV-free: School-age follow-up of a cluster-randomised trial in rural Zimbabwe
Source: PLoS Med. 2024 Oct 11;21(10):e1004347. doi: 10.1371/journal.pmed.1004347 (PMC11498706; doi:10.1371/journal.pmed.1004347)
Supplement: Attachment — Submitted filename: CBHF_CHU_response to reviewers.docx [file pmed.1004347.s006.docx]

3^rd^ May 2024

Dear Dr Schaefer,

Thank you for the opportunity to respond to the reviewers’ comments on our manuscript. We are delighted to respond to them below.

ACADEMIC EDITOR COMMENTS

It is a very relevant, well-expected study. The following key issues need to be attended to specifically:

1) From Reviewer 1: Please address the reviewer's concern about the imbalance in the reporting of results (unadjusted versus adjusted "p values" - rather than odds ratios). Please tone down the claims, as the differences are real but do not involve an overwhelming number of outcome variables.

**We have now applied a Bonferroni correction to account for multiple comparisons, which has not changed the inference of our main findings: we still see evidence of reduced cognitive function in CBHF for both the primary outcome of the Mental Processing index (MPI) and the secondary outcomes of School Achievement Test (SAT) and the Plus-EF test of executive function. Using the Bonferroni correction, there is no evidence of reduced cardiovascular fitness or lower head circumference, and the text of the paper has been adjusted to reflect this. Overall, we have toned down the findings as suggested, but we believe the cognitive findings are not only plausible (since we found similar differences at 2 years of age in this cohort), but also clinically meaningful and consistent across multiple outcomes. By contrast, we have reduced emphasis on other findings and try to provide a balanced view on the results we present.**

2) From Reviewer 5: Please address the potential lack of representativeness of the sample due to loss to follow-up, etc. Also address related issues such as missing values. These have been clearly outlined by Reviewer #5 and need to be addressed.
**We have addressed all these issues. We believe the sample is representative of the overall cohort, but we are careful also to point out the limitations of a substudy. We have further emphasised the supplementary tables, which compare the baseline characteristics of those who were enrolled or not enrolled in the follow-up study (eg Table S1 for CBHF, and Table S2 for CHU). The sample at 7 years was already compared to the sample at 18 months in Table S1 and S2 for CHU and CBHF and no significant differences were observed.**

**The concern regarding missing values has been addressed. Of note, the proportion of missing follow-up observations is very low across all variables (<2.5%) so is unlikely to bias the inferences. Where there were no covariate data, these were recoded as missing to enable adjusted models to run. We have commented on this in the revised Discussion. We have responded in full to Reviewer 5 regarding attrition. In brief, the missing data mechanism was explored and predictors of missingness were included as covariate adjustments within the models. By adjusting for the predictors within the model we effectively make the missing mechanism ‘missing completely at random’ (MCAR) in terms of missing data inference bias, and hence we do not believe that bias by attrition has been introduced.**

**We appreciate there are multiple measures and outcomes, and in response to Reviewer 1, we now adjust for multiple comparisons. For clarity, a ‘summary of findings’ Table S6 has been included in the supplementary materials to assist readers.**

**Summary of Findings table:**

| Overall findings following Bonferroni correction for children born HIV-free (CBHF) compared to those unexposed to HIV (p<0.002) | | | | | | |
| --- | --- | --- | --- | --- | --- | --- |
|  | **Variable** | **Effect size (SD)**  **Unadjusted (p)** | **Adjusted for contemporary covariates (p)** | **Adjusted for baseline covariates (p)** | **Subtests & child sex** | **Interpretation** |
| **Cognition** | Mental processing index (MPI) | CBHF 0.28 SD lower, p<0.001 | <0.001 | <0.001 | CBHF scored lower in all subtests for both sexes | Evidence that CBHF have globally reduced cognitive processing across all subtests, even when adjusting for contemporary or baseline covariates |
|  | School Achievement Test (SAT) | CBHF 0.24 SD lower, p<0.001 | 0.002 | 0.001 | CBHF scored lower in all subtests for both sexes | Evidence that CBHF have globally reduced academic function across literacy and numeracy, even when adjusting for contemporary or baseline covariates |
|  | Plus EF Executive function test (Plus EF) | CBHF 0.21 SD lower, p=0.001 | 0.004 | 0.002 | CBHF scored lower except in Flanker test, for both sexes | Some evidence that CBHF have reduced executive function (significant in unadjusted models only) |
|  | Fine motor | CBHF 0.14 SD slower, p=0.022 | 0.076 | 0.11 | No overall evidence of difference | No overall evidence of difference in fine motor function following Bonferroni correction |
|  | No evidence of difference for socioemotional function measured by Strengths and difficulties Questionnaire or child socioemotional score overall. | | | | Weak evidence that CBHF boys may have worse SDQ score | No overall evidence of difference for socioemotional function. Subgroup analysis provides weak evidence that CBHF boys may score worse for SDQ. |
| **Physical** | Maximal Oxygen consumption (VO_x_max) from shuttle run test | CBHF 0.26 SD lower, p<0.001 | 0.009 | 0.015 | Weak evidence that CBHF boys may have lower VO2max | Some evidence that CBHF have reduced cardiovascular fitness (significant in unadjusted models only) |
|  | No evidence of difference for grip strength, broad jump or blood pressure | | | | | |
| **Growth** | Head circumference | CBHF 0.20 SD smaller, p=0.009 | 0.003 | 0.002 | Both sexes affected | Weak evidence that CBHF may have reduced head circumference in unadjusted models only |
|  | No evidence of difference for any other measure of growth or body composition | | | |  |  |

3) From Reviewer 3 (statistical reviewer): In addition to the issues raised by Reviewer #1, the author needs to pay attention to the statistical issues raised by Reviewer #3. They are not major, but need to be addressed to clean up the paper.

**We have addressed the issues raised by the Statistical reviewer, including changing the title of the paper to an observational substudy. References to objective 3d in the protocol paper and clarification of cluster have been provided, as well as additional explanations of our directed acyclic graphs in the supplementary materials. P-values for interaction tests have also been included as recommended.**

GENERAL COMMENTS

1) Please include page numbers and line numbers in the manuscript file. Use continuous line numbers (do not restart the numbering on each page). For review purposes, we started counting the Abstract as page 1.

**Now included**

2) Please include the study protocol document and analysis plan, with any amendments, as Supporting Information to be published with the manuscript if accepted.

**Now included as Supporting Information**

3) Please report your study according to CONSORT, but explicitly state that it is a sub-study. Please ensure that the Abstract describes the main points of the original trial (2-3 sentences, including study population, study dates, intervention and primary outcome), but that the main part of the Abstract describes the details of the follow-up study.

**Abstract updated as suggested**

4) Please cite your Supporting Information as outlined here: <https://journals.plos.org/plosmedicine/s/supporting-information>

**We have updated our citation of the Supporting Information in line with guidelines**

COMPETING INTEREST

All authors must declare their relevant competing interests per the PLOS policy, which can be seen here:
<https://journals.plos.org/plosmedicine/s/competing-interests>

For authors with ties to industry, please indicate whether any of the interests has a financial stake in the results of the current study.

**Competing interests statement now included**

DATA AVAILABILITY

The Data Availability Statement (DAS) requires revision. For each data source used in your study:
a) If the data are freely or publicly available, note this and state the location of the data: within the paper, in Supporting Information files, or in a public repository (include the DOI or accession number).
b) If the data are owned by a third party but freely available upon request, please note this and state the owner of the data set and contact information for data requests (web or email address). Note that a study author cannot be the contact person for the data.
c) If the data are not freely available, please describe briefly the ethical, legal, or contractual restriction that prevents you from sharing it. Please also include an appropriate contact (web or email address) for inquiries (again, this cannot be a study author).

**We have updated our data availability statement in line with these guidelines, and have followed option B.**

ABSTRACT

1) PLOS Medicine requests that main results are quantified with 95% CIs as well as p values. When reporting p values please report as p<0.001 and where higher as the exact p value p=0.002, for example. For the purposes of transparent data reporting, if not including the aforementioned please clearly state the reasons why not.

**All results have been updated as requested**

2) Throughout, suggest reporting statistical information as follows to improve clarity for the reader “22% (95% CI [13%,28%]; p</=)”. Please amend throughout the abstract and main manuscript. Please note the use of commas to separate upper and lower bounds, as opposed to hyphens as these can be confused with reporting of negative values.

**All results have been updated as requested**

3) When a p value is given, please specify the statistical test used to determine it.

**This has been added throughout, including using an additional column for table 1, S1a and S1b.**

4) Please ensure that all numbers presented in the abstract are present and identical to numbers presented in the main manuscript text.

**We have double checked all numbers, and they are now identical in the abstract and main text (Table 2)**

5) If not already included, please include the study design, population and setting, number of participants, years during which the study took place, length of follow up, and main outcome measures.

**Added as requested**

6) Please include the important dependent variables that are adjusted for in the analyses.

**Added as requested**

7) Please define all abbreviations including those for statistical reporting at first use.

**All have been added**

AUTHOR SUMMARY

At this stage, we ask that you include a short, non-technical Author Summary of your research to make findings accessible to a wide audience that includes both scientists and non-scientists. The authors summary should consist of 2-3 succinct bullet points under each of the following headings:

• Why Was This Study Done? Authors should reflect on what was known about the topic before the research was published and why the research was needed.

• What Did the Researchers Do and Find? Authors should briefly describe the study design that was used and the study’s major findings. Do include the headline numbers from the study, such as the sample size and key findings.

• What Do These Findings Mean? Authors should reflect on the new knowledge generated by the research and the implications for practice, research, policy, or public health. Authors should also consider how the interpretation of the study’s findings may be affected by the study limitations. In the final bullet point of ‘What Do These Findings Mean?’, please describe the main limitations of the study in non-technical language.

Author Summary should immediately follow the Abstract in your revised manuscript. This text is subject to editorial change and should be distinct from the scientific abstract. Please see our author guidelines for more information: <https://journals.plos.org/plosmedicine/s/revising-your-manuscript#loc-author-summary>

**This has been added**

INTRODUCTION

1) If there has been a systematic review of the evidence related to your study (or you have conducted one), please refer to and reference that review and indicate whether it supports the need for your study.

**We have cited a recent systematic review of neurodevelopmental outcomes in the Introduction, which focused on children below 5 years of age, and showed differences in cognition between children with and without HIV exposure. We highlight the gap in neurodevelopmental outcomes at older ages.**

2) Please ensure to define abbreviations at first use, such as “IQ”. Please revise throughout the main manuscript.

**All have been defined.**

METHODS AND RESULTS

1) PLOS Medicine requests that main results are quantified with 95% CIs as well as p values. When reporting p values please report as p<0.001 and where higher as the exact p value p=0.002, for example. For the purposes of transparent data reporting, if not including the aforementioned please clearly state the reasons why not. We suggest reporting statistical information as detailed above – see under ABSTRACT

**Now included throughout the text**

2) Please present numerators and denominators for percentages, at least in the Tables [not necessarily each time they're mentioned].

**Now included in all tables**

3) Please define HUU at first use.

**This was an error as we use the term CHU (children HIV-exposed uninfected) throughout, so we have changed the few places where HUU was erroneously used, to now read CHU**

DISCUSSION

Please present and organize the Discussion as follows: a short, clear summary of the article's findings; what the study adds to existing research and where and why the results may differ from previous research; strengths and limitations of the study; implications and next steps for research, clinical practice, and/or public policy; one-paragraph conclusion.

**We have reordered the Discussion as suggested.**

FIGURES

For all Figures, please ensure that you have complied with our figures requirements <http://journals.plos.org/plosmedicine/s/figures>.

1) Please provide titles and legends for all figures (including those in Supporting Information files).

**All now included**

2) Please in the figure legend/description, define abbreviations used in each figure (including those in Supporting Information files).

**All abbreviations are defined**

3) Figure 1: Please use “selected” instead of “randomly selected” (“1349 Randomly selected”, third box on the main CHU path).

**Updated as requested**

4) Figure 0-1/0-2: Please define the meaning of the colors used in the graphs.

**Now clarified in the legend**

TABLES

1) Please note the use of commas to separate upper and lower bounds, as opposed to hyphens as these can be confused with reporting of negative values. Suggest reporting statistical information as detailed above – see under ABSTRACT

**All are commas, and all statistical information included as suggested**

2) Please provide titles and legends for all tables (including those in Supporting Information files).

**All now included**

3) Please define all abbreviations used in the table below each table (including those in Supporting Information files). For example, for table one: SOC, IYCF, WASH, SES, SD, CBHF, CHU.

**All abbreviations are now defined**

SUPPLEMENTARY MATERIAL

1) For supplementary figures and tables, please see the general comments under TABLES and FIGURES and amend accordingly.

**All have been updated as suggested**

2) We suggest reporting statistical information as detailed above – see under ABSTRACT. Please define all numerical values.

**All have been updated as suggested**

3) As for the main manuscript, please indicate whether analyses are adjusted to help facilitate transparent data reporting please also detail the factors adjusted for and present the unadjusted analyses for comparison. If not, please clearly state the reasons why not.

**Unadjusted and adjusted data are shown, including details of all covariates used in models**

4) Please revise the references according to the comments below - see under REFERENCES.

**Revised as suggested**

REFERENCES

1) PLOS uses the numbered citation (citation-sequence) method and first six authors, et al.

**Revised as suggested**

2) Please ensure that journal name abbreviations match those found in the National Center for Biotechnology Information (NCBI) databases (<http://www.ncbi.nlm.nih.gov/nlmcatalog/journals>), and are appropriately formatted and capitalised.

**Revised as suggested**

3) Where website addresses are cited, please specify the date of access (e.g. [accessed: 12/06/2023]).

**Revised as suggested**

4) Please also see <https://journals.plos.org/plosmedicine/s/submission-guidelines#loc-references> for further details on reference formatting.

REVIEWER 1

I would recommend that the authors first add both unadjusted and corrected p-values for all estimates in Tables 2-4, and then carefully revise the abstract and Discussion sections in light of the not-so-overwhelming evidence. Simply reporting results that are aligned with the hypothesis of the authors is really not okay, and seriously undermines the scientific validity and quality of this otherwise so carefully done study.

**Thank you for the above suggestion. We have included p-values to 3 decimal places and performed a Bonferroni correction to account for multiple testing. Of note, the cognitive differences originally reported are still significant with the Bonferroni correction. However, there is only weak evidence of a difference in head circumference and cardiovascular function following Bonferroni correction, and we have adjusted the text accordingly, to focus on differences our inferences on differences in cognition in the abstract and the manuscript.**

Minor Comments
-       Abstract Methods & Findings: given the small proportion of kids selected for this study, it would be good to report attrition rates (% of targeted kids assessed) .

**Thank you for this suggestion: this has been included in the abstract as 70% of CBHF and 49% of CHU who completed the SHINE trial endpoint at age 18 months in Shurugwi.**

-       Abstract: mean difference was lower - should either be mean difference was -3, or the mean score was 3 points lower. Differences in points are not very informative if the scale is not presented - I would suggest to either report normalized z-score differences here or define the mean and SD of scales used**.**

**Thank you for this point; we have used the word ‘score’ instead. Normalised Z-score differences are difficult to compare as some cognitive tests such as the SAT were adapted for this cohort, whereas others such as the KABC-II have Z-scores based on child populations in the USA which have a very different context. Hence mean and standard deviations of the scores have been provided in the results tables, and also included in the text of the abstract, which enables readers to assess the magnitude of difference being reported.**

- Page 6: the pre-analysis plan looks very different to what is presented here - is there a separate paper looking at long term treatment effects? Please explain how this paper is related to the study described in the protocol - is this just covering hypothesis 3d? The way this is presented here and on page 9 (statistical analysis) one may think that there is a plan for this paper specifically which is quite misleading.

**We apologise for any confusion. The study protocol paper describes an overall plan for this cohort, with several objectives presented. One of the pre-specified objectives in the protocol paper is reported in the current manuscript - objective 3d: comparison of long-term growth, physical and cognitive function by HIV exposure status.**

**The pre-specified approach outlined in the protocol paper is consistent with what is presented in the current manuscript. The main outcome is cognition, as assessed using the Kaufman Assessment Battery for Children (KABC-II), with other pre-specified secondary outcomes as presented in the protocol paper. The analysis uses generalised estimating equations. Assessors were blinded to the HIV status of the caregiver until measurements were completed. Since child development is shaped by many factors (including both early-life and contemporary environments) two adjusted models are used for analysis: one which adjusts for contemporary covariates within our conceptual framework, and one which adjusts for baseline covariates within the conceptual framework. Directed acyclic graphs were created using Dagitty to select confounders: which already eliminates colliders and mediators, therefore the list of confounders should not be creating biased inferences.**

**We have updated the methods section to highlight the relevant sections of the protocol and SAP.**


6.  Page 20 - for the subgroup analyses I would add the p-value for the interaction test when they are mentioned (supporting information). At the end of the paragraph, I would recommend mentioning the number of 'other growth or body composition outcomes' that were not significant (I count 23). Put this in to discussion on multiple testing, Overall I don't think 4 out of 27 significant results at p<0.1 is beyond what one would expect by chance here, so making these changes should hopefully ensure the results are not overinterpreted.

**The P-value for the interaction tests for the subgroup analyses for child sex were included in Table S3 and are now also included in the text on page 20. We have also mentioned the number of interactions that were not significant on page 20 and included a mention of this in the discussion. Throughout the revised paper, we have tried to avoid over-interpretation. As stated above, a Bonferroni correction has been applied and our inferences now focus only on the consistent differences in cognition across multiple outcomes; by contrast, we have not focused on head circumference and cardiovascular fitness differences following the adjustment for multiple comparisons.**

Reviewer #4:

This study is commendably well-conducted, particularly regarding the comprehensive range of measurements and the meticulous application of rigorous instruments. My overall critique, however, hinges on the pivotal nature of the research question, which, in my opinion, could be further refined by leaning into the biological hypothesis more clearly. I recognize that the evidence to date is far from strong, but that is why this paper becomes more interesting if it does so.
One important issue in studies on how HIV exposure impacts child development is related to whether the associations are due to socio-economic or biological factors. From my perspective, the socio-economic hypothesis is both less compelling and less plausible. It is unlikely that HIV exposure alone significantly alters a child's social standing or existence to an extent that affects developmental outcomes. Instead, socio-economic links to development are more likely and highly plausibly confounded by factors associated with a mother's HIV status (and therefore her child's consequent exposure), and poor development. Despite efforts to measure various factors, many studies do not capture the complexity of individual circumstances or the socio-economic nuances of the mother. Thus, residual confounding or unmeasured confounding is the most probable explanation for the observed association between HIV exposure without infection and negative outcomes in children.

On the other hand, a biological hypothesis is more interesting.  There are documented potential mechanistic effects of HIV exposure on development, as noted in the discussion.  For example, the discussion of medication effects on HIV-exposed children and hypotheses around inflammation opens the door to more biologically centered hypotheses. By embracing a biological hypothesis, the evidence here could become more open to falsification but at the same time potentially more compelling.  Imagine if the hypothesis was that HIV exposure itself has developmental effects that cannot be easily explained by social and psychological factors, and therefore support clinical effects mediated by plausible biological mechanism.

**We agree with the Reviewer that our adjusted models suggest that the cognitive differences we find at age 7 years are not entirely explained by universal risk factors, since our adjusted models (which consider both antenatal and contemporary socioeconomic, environmental and psychosocial differences) still show significant differences in cognition between groups. Since the original submission of this manuscript, we have published findings from the same cohort in early infancy, which show biological differences in early life, including systemic immune activation, differences in immunophenotype within the CD8 T-cell compartment, and earlier CMV viraemia in children who are HIV-exposed (now cited). It is plausible these perturbations would affect sensitive processes like neurodevelopment and thus explain the long-term effects demonstrated here. We have tried to bring this point out more in the revised Discussion.** **However, residual confounding remains a possibility, and we have added a comment on this in the Discussion.**

Clarification is needed regarding the SHINE trial's recruitment and enrolment procedures. The significance of these findings largely depends on their capacity to represent population-level effects of HIV exposure. Selection bias could profoundly influence the observed associations. Although the follow-up observational study appears to have equitably enrolled HIV-exposed and unexposed participants, the initial trial enrolment's potential biases have not been sufficiently detailed.

**Thank you for this important point. In the original trial, contemporaneous enrolment of pregnant women occurred across the community, regardless of HIV status, through prospective pregnancy surveillance at household level. In a setting where 15% of women are living with HIV, we pre-specified that all trial outcomes would be stratified by maternal HIV status, but due to the generalised HIV epidemic that persists in rural Zimbabwe, we do not believe there was any enrolment bias by HIV status in the original trial.**

**However, re-enrolment into the long-term follow-up cohort at age 7 years could have led to some bias, since not all children were followed up. To address this, we have compared the characteristics of those who were re-enrolled or not re-enrolled in Table S1 and Table S2, and have discussed these in response to Reviewer 5 in more detail.**

An important oversight from the report is the description of the clinical significance of the observed differences. With diverse tests assessing neurocognitive and cardiovascular functions, the relevance of these disparities is unclear. Without understanding their place on a standard distribution curve, it's difficult to assess the impact of these differences on the children's life spans and regional public health.  I suspect they are large, but I am not expert in any one of these and so I am uncertain.

**Thank you for this point. As discussed above in response to Reviewer 1, our inferences in the revised manuscript are based only on the significant differences in cognition that are apparent across a range of outcomes, despite adjustment for multiple comparisons. Our revised analysis shows only weak evidence of difference in head circumference and cardiovascular fitness and so these are no longer discussed in detail in the revised paper, and are removed from the abstract.**

**Among cognitive scores, the Mental Processing Index was approximately 3 points lower in CBHF, with an average score of 45.2 (standard deviation 10.5) compared to 48.3 (11.3) in CHU, representing a Hedges’ g effect size of +0.28 SD. From other published data, 0.2 to 0.3 SD difference in cognition is clinically meaningful – for example, it is similar to the effect size reported following interventions that are designed to improve child development, such as that observed at 3-7 years of age among children followed-up in the INCAP study[1], following early-life randomisation to nutrition interventions[1]. The INCAP trial also noted a similar variance of 5% (SD 0.2) attributable to the intervention 20 years later[2]. A 0.2-0.3 standard deviation difference was also the approximate magnitude of benefit in socio-emotional scores in a previous trial of small-quantity lipid-based nutrient supplements (SQ-LNS) [3]**. **Early-life stimulation also had an impact of a similar magnitude in Jamaica [4]. On a national level, each point higher in average intelligence quotient (IQ) on a score of 100 is associated with a 0.1% increase in a nation’s GDP[5], suggesting that long-term differences of this magnitude have an impact.**

The paper's long follow-up period is a key asset, recognizing that HIV-exposed yet uninfected children face a range of adverse outcomes, including increased mortality and developmental challenges. The use of directed acyclic graphs stands out methodologically, yet the rationale behind the selection of variable groupings requires more explanation to enhance comprehension. The justification for employing three different models in the study remains somewhat opaque. While I presume all variable sets are sufficient to block all backdoor paths, the purpose of the additional adjustment sets, whether for efficiency or to address uncertainties around the minimal variable set needed to close all confounding pathways, or other rationale, is not immediately apparent.

**Thank you for this comment. We have tried to make our approach clearer in the revised paper. We used three models to consider i) unadjusted differences, ii) adjustment for baseline covariates at enrolment to the original trial, and iii) adjustment for current covariates, all of which may shape child development. Differences using generalised estimating equations without any adjustment were first explored. In all adjusted models, we also included trial factors as covariates: study arm, study nurse, exact child age, calendar month recruited, temperature and sex, since these may influence child developmental assessments. Our DAG was based on a conceptual framework of baseline and contemporary factors that might influence child development. The confounders were selected by the software (DAGitty) which already eliminates colliders and mediators, therefore the list of confounders should not be creating biased inferences. We have provided a more comprehensive footnote to each DAG in the revised submission.**

Other strengths of the paper include the impressive stability of differences after adjustment and the novel exploration of child outcomes beyond two years, a period not extensively covered in existing research. This leaves open questions about whether these disparities will resolve, persist, or widen as time progresses.

**We thank the reviewer for this comment. We agree that this is the unique strength of this cohort, since few children have been followed with a contemporaneous HIV-unexposed comparison group to 7 years of age.**

To me, the issue of multiple hypothesis testing does not diminish the study's credibility.

**We thank the reviewer for this comment, although Reviewer 1 felt strongly that the analysis should adjust for multiple comparisons and our revised analysis therefore is based on this approach. The consistent and meaningful differences in cognition between groups persist, and remains the key conclusion of this long-term follow-up cohort.**

Reviewer #5:

Thank you for the opportunity to review this manuscript. I would like to congratulate the authors on a well-executed study and clearly written manuscript. The paper examines the long-term differences in cognitive and growth outcomes between children born HIV-free and children HIV unexposed who participated in the SHINE trial. Overall, the results are interesting and fill an important gap in the literature. I have laid out a few major and some minor issues below that can help improve the paper.

MAJOR comments
*       Representativeness of the sample - the authors argue that the analytical sample is likely representative of the SHINE cohort (p23), but in my opinion they fail to show this clearly.

**Thank you for this important comment. We refer to Table S1 and S2 which describe baseline characteristics of those enrolled and not enrolled. These were broadly similar, and we therefore believe that our overall findings are likely to be representative. However, we have also discussed this further in the limitations section of the discussion.**

o       First, they only selected one district for the follow up and it's not clear why only one district was selected or how it was selected. Were there any major differences between the two districts at the start of the trial?

**Thank you for this point. One district was selected for follow-up due to logistical and financial constraints which meant we could not operate across two districts in this rural setting, where the costs of maintaining research infrastructure across such a large area is prohibitive. The two districts are contiguous within Midlands Province, and are similar in geography, demography, water and sanitation access[6], therefore there were no major differences between the districts at the start of the trial, and cluster-randomization occurred across the whole area, and not stratified by district. As noted above, the follow-up cohort are broadly similar to the whole population in the original SHINE trial.**

o       Second, sup tables 1a and 1b compare those included in the 7-year follow-up to those excluded at baseline. Many of the differences are not only significant but also quite large in terms of magnitude. In addition, the Ns reported don't match the flow chart, e.g., table s1a 267 included in follow up when flow says 264; 459 not included when flow says 421 live births. These Ns need to be reconciled.

**Thank you for this point. We have double checked all numbers: 267 mothers with HIV were included in follow-up. At the 7 year-visit, 267 included both mothers and other primary caregivers (for example if the mother had died or moved away) for the child at 7 years. 273 children were born to these mothers (including twins). From these 273 children, 5 were HIV-positive and 4 had a severe disability hence 264 were included in the analysis.**

**459 mothers with HIV were not included in follow-up across both districts. These mothers had 465 live births across both districts. (Note that since these mothers were not followed up, we do not know what proportion were being looked after either by the mother or a different primary caregiver). The comparison for table S1 and S2 is comparing those selected in the substudy with those not selected for the whole cohort across both districts, hence 459 is the number of HIV positive mothers not selected in both districts.**

**From the flow diagram, a total of 421 live births occurred among mothers with HIV in Shurugwi district only. However there were 23 neonatal deaths, 1 child exited, 13 infant deaths and 8 lost to follow-up so 376 completed the SHINE study at 18 months in Shurugwi. Of those 376 children at 18 months, 2 died, 6 were lost to follow-up, 2 declined, 6 aged out and 87 relocated out of the district, to leave 273 included within the SHINE study.**

**In terms of the baseline differences between those enrolled versus not enrolled, 42 baseline comparisons were made in Table S1. In response to Reviewer 1 comments, we have now applied a Bonferroni correction to all data in this manuscript, due to the multiple comparisons being made. With a significance threshold of p < 0.001, only 2 comparisons remain significant. The first is the median volume of water collected in the past 24 hours which was 6.7L (IQR 4.0, 10.0) among those included, and 8.6L (IQR 5.0, 13.3) among those not included. Our previous analyses have shown that the daily volume of water collected was not associated with early-life child growth or anaemia. The second difference is a slightly older mean maternal age (30.2 years (SD 6.1) for those included compared 28.6 years (SD 6.3) for those not included). Higher maternal age may be associated with higher socioeconomic status in some contexts, but this was not observed in the SHINE cohort, and we believe this difference of 1.5 years is unlikely to be clinically significant.**

**Among CHU, three baseline comparisons in Table S2 have p < 0.001. First, median per capita water volume collected was lower (6.7L, IQR 4.4, 10.0 vs 7.5L, IQR 5.0, 12.0) but – as discussed above – this variable was not associated with early-life child growth or anaemia. Second, enrolled children’s mothers had higher (and hence more progressive) median scores of baseline gender norms (2.3, IQR 1.5, 3.0 for those enrolled versus 1.7, IQR 1.5, 3.0 for those not enrolled). Baseline gender norms were associated with slight improvements in child growth in a previous published analysis from this cohort; however, each unit increase was associated with only +0.09 LAZ, so the magnitude of difference between those enrolled vs not enrolled (0.6 points) would have a very small impact on average child growth by age 18 months[7]. Third, the mean weight for height Z-score at 18 months was slightly lower among those included (-0.04, SD 1.0) compared to those not included (0.10, SD (1.1). This difference (~0.1 in WHZ) is unlikely to be clinically significant in a cohort that had very low rates of wasting.**

**Taken together, we have provided all the available information for readers to judge the generalisability of the re-enrolled cohort, and have been careful to point out the limitations of a substudy, but overall we do not believe that these baseline differences have introduced substantial bias into our sample.**

o       Third, the sample at 7y is not compared to the sample at 18 mo. Some kids were lost to follow up during the trial and others afterwards. Is the sample at 7 y representative of the cohort that completed the trial?

**Tables S1 and S2 present characteristics of enrolled and non-enrolled children at 18 months of age. This was when the primary endpoint of the original trial was evaluated, and Table S1 shows there were no significant differences in anthropometry or haemoglobin between the CHBF included or not included in the sample at 18 months. For CHU (Table S2), the only difference at 18 months was for mean weight for height Z-score at 18 months, as explained above, which we do not believe is a meaningful difference.**

*       Persistent effects of HIV exposure - the authors can make a stronger argument that the "effects" of HIV exposure persist later in life by showing what the effects were at 18 months of age. You've referenced a prior paper but the sample is different there. And as I argue in my previous point, I'm not convinced by what is shown in the current paper that the subsample followed at 7 y is representative of the full SHINE cohort. It would strengthen the paper to show results at 18 mo for the sample being analysed at 7 y.

**The effects at 18 months were already presented in supplementary Table S3 under ‘child variables’. However, the key results that show differences in 18 month growth between groups are now described in the main discussion text of the paper. Briefly, after applying a Bonferroni correction, CBHF at age 18 months had lower mean (SD) LAZ of -1.83 (1.06) compared to mean (SD) CHU of -1.50 (1.02). CBHF also had a higher proportion of underweight (16.9%) vs CHU (8.8%). Of note, CBHF also had a smaller mean (SD) head circumference at 18 months of-0.46 (1.07) compared with CHU -0.21 (0.98). These findings among the subgroup of children included in the current paper are entirely consistent with previously published comparisons from the whole cohort (Evans et al., Clin Infect Dis 2020).**

*       Related, you note that that the disparity between CBHF and CHU is in fact larger than at 2 years of age, but these results aren't shown or formally compared in the text. I think this finding needs to be highlighted a bit more as it has important programmatic implications.

**Thank you for this comment. We have now made this point in the main text of the paper. We have also included the Hedges’ g-effect size in standard deviations in the results table to aid with explanation.**

*       Attrition - overall, attrition is pretty high which is not unexpected given the long follow up. Of the 376 CBHF who completed SHINE, you enrolled 264, which is ~70%. Did you conduct any sensitivity analyses (eg IPW) to account for attrition?

**The missing data mechanism was explored and predictors of missingness were included as covariate adjustments within the models. By adjusting for the predictors within the model we effectively make the missing mechanism ‘missing completely at random’ (MCAR) in terms of missing data inference bias and hence bias by attrition has not been introduced.  Adjustment for predictors of missingness incorporates information about the missing data mechanism into our analyses and thus methods like imputation or IPW (under the MAR assumption) will unlikely provide additional benefits (as they would be based on the already adjusted predictors) and could even introduce bias if the model for estimating the probabilities of missingness is mis-specified. We therefore have not conducted sensitivity analyses for attrition under MAR as our approach will by default result in the same inferences. We have also not explored sensitivity analyses under MNAR as the dataset is rich and MNAR information is not apparent, thus any analyses are likely to be extremely limited in exploring inferential robustness.**

*       Child assessments - a bit more information in the main text would be useful, particularly around the validation/adaption of the tools (you don't note until the discussion that there was an article on developing the toolbox) and training received by the nurses.

**Thank you for this point. The SAHARAN toolbox paper was cited but this is now explicitly mentioned in the text, as this forms the set of assessments that were made in this cohort.**

It would also be helpful to describe the order in which the assessments were administered (was it always the same?) and whether they were administered at the same time of the day.

**Thank you for this. The order was always the same and this is now specified and described in detail.**

These and other administration factors can influence how children perform on the assessments. Although you do control for nurse in the regressions, you don't control for other factors that can influence performance at the time of assessment.

**We agree that these assessments can be influenced by multiple factors, and our nurses followed a consistent order and were regularly standardized. We did control for other trial factors in our models, including ambient temperature, and the time of year the measurement was conducted, in addition to study nurse.**

*       Missing data - there is no mention of how missing data on any of the variables (outcome or covariates) were handled. Some of the table notes mention missing data, but this should be consistently described in the methods.

**For missing outcome observations, the number was too small (<3%) to change our inferences and we therefore did not use any methods to account for missingness. For covariate data, missing observations were recoded as missing and included as a categorical variable in models. A limitation we acknowledge in the discussion is that this may reduce the variability in the covariate data.**

### References

1.	Martorell R. Improved Nutrition in the First 1000 Days and Adult Human Capital and Health. American journal of human biology : the official journal of the Human Biology Council. 2017;29(2). doi: 10.1002/ajhb.22952.

2.	Pollitt E, Gorman KS, Engle PL, Martorell R, Rivera J. Early supplementary feeding and cognition: effects over two decades. Monogr Soc Res Child Dev. 1993;58(7):1-99; discussion 111-8. PubMed PMID: 8272081.

3.	Ocansey ME, Adu-Afarwuah S, Kumordzie SM, Okronipa H, Young RR, Tamakloe SM, et al. Prenatal and postnatal lipid-based nutrient supplementation and cognitive, social-emotional, and motor function in preschool-aged children in Ghana: a follow-up of a randomized controlled trial. Am J Clin Nutr. 2019;109(2):322-34. Epub 2019/02/06. doi: 10.1093/ajcn/nqy303. PubMed PMID: 30721937; PubMed Central PMCID: PMCPMC6367954.

4.	Walker SP, Chang SM, Wright AS, Pinto R, Heckman JJ, Grantham-McGregor SM. Cognitive, psychosocial, and behaviour gains at age 31 years from the Jamaica early childhood stimulation trial. Journal of Child Psychology and Psychiatry. 2022;63(6):626-35. doi: <https://doi.org/10.1111/jcpp.13499>.

5.	Jones G, Schneider WJ. Intelligence, Human Capital, and Economic Growth: A Bayesian Averaging of Classical Estimates (BACE) Approach. Journal of Economic Growth. 2006;11(1):71-93.

6.	Humphrey JH, Jones AD, Manges A, Mangwadu G, Maluccio JA, Mbuya MN, et al. The Sanitation Hygiene Infant Nutrition Efficacy (SHINE) Trial: Rationale, Design, and Methods. Clin Infect Dis. 2015;61 Suppl 7:S685-702. Epub 2015/11/26. doi: 10.1093/cid/civ844. PubMed PMID: 26602296; PubMed Central PMCID: PMCPMC4657589.

7.	Tome J, Mbuya MNN, Makasi RR, Ntozini R, Prendergast AJ, Dickin KL, et al. Maternal caregiving capabilities are associated with child linear growth in rural Zimbabwe. Maternal & child nutrition. 2021;17(2):e13122. doi: <https://doi.org/10.1111/mcn.13122>.
