# Supplementary material for: Growth, physical, and cognitive function in children who are born HIV-free: School-age follow-up of a cluster-randomised trial in rural Zimbabwe
Source: PLoS Med. 2024 Oct 11;21(10):e1004347. doi: 10.1371/journal.pmed.1004347 (PMC11498706; doi:10.1371/journal.pmed.1004347)
Supplement: Attachment — Submitted filename: PLOS reply to reviewers & editor.docx [file pmed.1004347.s007.docx]

## Reply to editors and reviewers

12^th^ July 2024

**Dear PLoS Medicine,**

**Thank you for the opportunity for these minor corrections. We believe we have now answered all reviewers and editors’ requests and attach the updated manuscript with tracked changes and clean versions. We also attach the updated supporting information with tracked changes and clean version.**

**We noted a slight coding error for some of the adjusted calculations, so some of the coefficient and confidence intervals have changed slightly, but this has not altered the inferences made within the paper.**

**Please find our detailed comments below.**

**Yours sincerely**

**Dr Joe D Piper**

**BMBCh, PhD, MRCPCH, DTM&H**

Dear Dr. Piper,

Thank you very much for re-submitting your manuscript "Growth, physical and cognitive function in children who are born HIV-free: school-age follow-up of a cluster-randomized trial in rural Zimbabwe" (PMEDICINE-D-23-03875R2) for review by PLOS Medicine.

Thank you for your detailed response to the editors' and reviewers' comments. I have discussed the paper with my colleagues and the academic editor, and it has also been seen again by four of the original reviewers. The changes made to the paper were mostly satisfactory to the reviewers. As such, we intend to accept the paper for publication, pending your attention to the remaining reviewer and editorial comments below in a further revision. When submitting your revised paper, please once again include a detailed point-by-point response to the editorial comments.

The remaining issues that need to be addressed are listed at the end of this email. Any accompanying reviewer attachments can be seen via the link below. Please take these into account before resubmitting your manuscript:


In revising the manuscript for further consideration here, please ensure you address the specific points made by each reviewer and the editors. In your rebuttal letter you should indicate your response to the reviewers' and editors' comments and the changes you have made in the manuscript. Please submit a clean version of the paper as the main article file. A version with changes marked must also be uploaded as a marked up manuscript file.

Please also check the guidelines for revised papers at <http://journals.plos.org/plosmedicine/s/revising-your-manuscript> for any that apply to your paper. If you haven't already, we ask that you provide a short, non-technical Author Summary of your research to make findings accessible to a wide audience that includes both scientists and non-scientists. The Author Summary should immediately follow the Abstract in your revised manuscript. This text is subject to editorial change and should be distinct from the scientific abstract.

Please note, when your manuscript is accepted, an uncorrected proof of your manuscript will be published online ahead of the final version, unless you've already opted out via the online submission form. If, for any reason, you do not want an earlier version of your manuscript published online or are unsure if you have already indicated as such, please let the journal staff know immediately at [plosmedicine@plos.org](mailto:plosmedicine@plos.org).

We ask that you submit your revision within 1 week (Jul 01 2024). However, if this deadline is not feasible, please contact me by email, and we can discuss a suitable alternative.

Please do not hesitate to contact me directly with any questions ([atosun@plos.org](mailto:atosun@plos.org)). If you reply directly to this message, please be sure to 'Reply All' so your message comes directly to my inbox.

We look forward to receiving the revised manuscript.   

Sincerely,

Alexandra Tosun, PhD
Associate Editor 
PLOS Medicine
[plosmedicine.org](http://plosmedicine.org)

***Please note while forming your response, if your article is accepted, you may have the opportunity to make the peer review history publicly available. The record will include editor decision letters (with reviews) and your responses to reviewer comments. If eligible, we will contact you to opt in or out.***

------------------------------------------------------------

**Thank you for the opportunity to make these minor corrections. Please see our comments below to each individual point. Please note that there was a small coding error in the adjusted models for the study treatment arm, this has now been corrected. This has changed some of the confidence intervals, but has not changed the inferences made.**

Requests from Editors:

DATA AVAILABILITY STATEMENT

Please update the statement in the online submission form with the details provided in ll.273-278 and remove the data availability statement from the main text.

**This has been updated**

ABSTRACT

1) ll.3-4: “Globally, over 16 million children were exposed to HIV during pregnancy but remain HIV free at birth and throughout childhood.” – please add the time frame/point.

**This has been included: from 2022**

2) l.32, please change to: “…number of children’s books)…” **This has been updated**

3) l.38: Please write ‘SD’ and ‘CI’ in full. **This has been written in full**

4) l.45, please change to: “In this study, we found that … had …”. **This sentence has been updated as requested**

AUTHOR SUMMARY

1) l.52: “Over 16 million children globally were born HIV-free (CBHF) to mothers living with HIV.” – please add the time frame/point. **The time point has been added**

2) l.59: Please write ‘SHINE’ in full. **Sanitation, Hygiene, Infant Nutrition Efficacy trial has been included**

3) In the final bullet point of the ‘What do these findings mean’ sub-section, please include the main limitations of the study in non-technical language. **The main limitations have been included.**

INTRODUCTION

l.97: Please define ‘CMV’ at first use. **This has been defined as Cytomegalovirus**

RESULTS

1) l.282: Please define ‘IQR’ at first use. **Inter-quartile range has been included**

2) Figure 1: Please define ‘CBHF’, ‘CHU’, ‘SHINE’ and ‘SFU’. **These have been defined**

3) ll.307-308, please change to: “CBHF caregivers also had higher depression scores (4.1 vs 3.2) compared to CHU.” **This has been corrected**

4) l.325: Please define ‘SD’ at first use. **This has been included**

5) Table 1: Please define ‘CBHF’, ‘CHU’, ‘SES’, ‘SHINE’ (at first use). **This has been corrected**

6) ll.321-322, please change to: “Baseline factors measured during participation in the original trial are shown in Tables S1 and S2 [23].” **This has been corrected.**

7) ll.323-323: “All scales are explained in supplementary materials.” – please specify. **These scales are now specified in the table legend by using acronyms defined in the table**

8) l.334: Please add statistical information, e.g. “(mean (SD) -0.46 [1.07] vs -0.21 [0.98])”. **This has been corrected**

9) Table 2: Please define ‘CI’, ‘CBHF’, ‘CHU’, ‘EF’, ‘SDQ’ (or ensure to introduce the abbreviations properly). **This has been corrected**

10) l.356/367/381: Please change to ‘at 18 months’. **This has been changed for line 349 only, since the results on line 367 and 381 are for age 7 years, so ‘at 7 years’ has been added.**

11) Table 3: Please define ‘CI’, ‘CBHF’, ‘CHU’. **These have been defined**

12) Table 4: Please define ‘CI’, ‘CBHF’, ‘CHU’, ‘MUAC’. **These have been defined**

13) l.400, please change to: 'sex of children'. Please also revise Table S4 accordingly. **This has been adjusted**

14) l.405/406: “CHU boys/CBHF boys”” – please re-word and use patient-centered language, e.g. “boys in the CHU group”. Please revise accordingly through the main text. **This has been adjusted**

DISCUSSION

1) ll.421-423: “The gap in neurodevelopment of up to 0.28 SD is, if anything, greater than the disparity in neurodevelopmental scores at age 2 years of up to 0.15 SD previously reported in the same cohort [21]. – The comparison, while interesting, has its limitations due to different study cohorts being compared, which may be worth clarifying and reiterating at this point. **Thank you; this limitation has been added**

2) l.436: "(6 outside the USA)" - Given that the current study was conducted in Zimbabwe, the information "6 outside the USA" seems less helpful. Could this be further specified? **Thank you; the specific countries have been added**

3) l.443: Please ensure to introduce abbreviations, such as ‘MPI’, or write in full. **Thank you; that has been corrected.**

4) l.452: Please write ‘MRI’ in full. **Thank you; this has been corrected.**

REFERENCES

1) Where website addresses are cited, please specify the date of access using use the word “accessed” (e.g. [accessed: 10/04/2024]). **Thank you; this has been addressed.**

2) Please ensure that journal name abbreviations match those found in the National Center for Biotechnology Information (NCBI) databases (<http://www.ncbi.nlm.nih.gov/nlmcatalog/journals>), and are appropriately formatted and capitalised. For example, “Frontiers in Pediatrics” in reference [1] should be “Front Pediatr.

3) RE reference [25, 26] – it seems that the two references are identical. Please check and update the status (i.e., “[version 2; peer review: 2 approved]”). **Thank you; this has been updated**

4) Please revise the supporting information references according to the comments above. **Thank you; these have also been updated**


SUPPLEMENTARY MATERIAL

1) Please revise the supporting information, especially the definition of abbreviations. **Thank you; this has been revised**

2) S2 Figure: Please note that in the file you submitted (and in the file ‘CBHF_CHU_PlosMed_Supp_info.docx’), the graph appears to be cut off on the left and right sides. **Thank you; this has been corrected.**

3) In the published article, supporting information files are accessed only through a hyperlink attached to the captions. For this reason, you must list captions at the end of your manuscript file. You may include a caption within the supporting information file itself, as long as that caption is also provided in the manuscript file. Do not submit a separate caption file. **This has been adjusted, with S1 Supporting Information provided.**

When SI files are contained with a single file:
Please label the file as ‘S1 Supporting Information’.
Please apply alphabetical labelling to each table and figure contained within the S1 file. For example, ‘Fig A’ to ‘Fig Z’ and ‘Table A’ to ‘Table Z’.
Plain text does not need to be labelled and can just be given a title as necessary. For example, ‘Statistical Analysis Plan’.
Please cite tables/figures as ‘Fig A in S1 Supporting Information’ and/or ‘Table A in S1 Supporting Information’, for example.
Please cite plain text as, ‘Statistical Analysis Plan in S1 Supporting Information’, for example.

When SI files are uploaded as separate files:
Please label tables as ‘S1 Table’ (so on) and figures as ‘S1 Fig’ (and so on).
Any additional documents (protocols/analysis plans etc.) can be labelled as ‘S1 Protocol’, for example. Please cite items as exactly as labelled.

**All files have been labelled as requested**

SOCIAL MEDIA

To help us extend the reach of your research, please provide any X (formerly known as Twitter) handle(s) that would be appropriate to tag, including your own, your co-authors’, your institution, funder, or lab. Please enter in the submission form any handles you wish to be included when we post about this paper.

**Thank you; @ZvitamboZim has been added**

Comments from Reviewers:

Reviewer #1: I would like to thank the authors for the careful revision of the manuscript - I think the draft is much improved and much easier to follow now. I am still not fully happy with the presentation of results though:

-       Figure 1: it would be nice to also have the percentages included in each step here. I think reporting the 990 CHU as «49%» is quite misleading, considering that you selected only some children for this follow-up - I think it would be more appropriate to report this as percentage of the 1349 selected shown in the figure. If this is the correct denominator, follow-up rates would be slightly higher in the CHU group, but these differences are likely not statistically significant (would be good to test and report this).

**Thank you for this comment: we would prefer to keep the actual numbers in the CONSORT diagram to show the flow of those recruited, and we are concerned that adding percentages may confuse readers. For example, the 1349 represent those who were selected at random from the original 2016 CHU in Shurugwi; this represents 1002 selected from 2016 plus randomly selected replacements for those who declined (1), relocated (337) or aged out (1). Therefore, as children became ineligible because of these reasons, further random selections were made from the original 2016. We agree that the 49% flagged by the reviewer is misleading, so we have taken percentages out from the Abstract and this now reads: “From 5280 pregnant women originally enrolled, 376 CBHF and 2016 CHU reached the trial endpoint at 18 months in Shurugwi; of these, 264 CBHF and 990 CHU were evaluated at age 7 years using the School-Age Health, Activity, Resilience, Anthropometry and Neurocognitive (SAHARAN) toolbox.”**

-       Methods: some more details on the multiple testing corrections would be helpful here - how did you determine the 0.002 cutoff? Was this based on the 27 comparisons mentioned in the legend of the table? If yes, the correct cutoff would be 0.05/27, which would be around 0.00185. Please provide a rationale for and more detail on this in the Methods section. Given that hypotheses are likely not independent here, other types of corrections (step-down, or FDR) would likely be a bit more forgiving here. **Thank you for this point; this has been clarified in the Abstract (lines 37-38) and main manuscript (lines 279-281) which now reads: “Since there were 27 outcomes, a Bonferroni correction was applied (0.05/27) leading to a threshold of p<0.00185 being considered significant.”**

-       Tables 2-4 claim that you corrected for multiple testing, and yet, the table shows only uncorrected p-values. Please kindly indicate differences that are below this threshold with bold font or some other markers. **Thank you for this suggestion; a bold font has been applied to show results that reached the threshold of p<0.00185, with a footnote which reads: “A Bonferroni correction was applied for the 27 comparisons, with a p-value <0.00185 considered significant; results reaching this threshold are highlighted in bold.”**

**-       Abstract: you should state clearly here that 27 variables were analyzed, and that statistically different results were only found for 2 (or 3?) of these variables. Is the EF p-value below or above this cutoff? Thank you; this has been clarified in the Abstract, and we added actual numbers to clarify our inferences. This section now reads: “We applied a Bonferroni correction for the 27 comparisons (0.05/27) and considered a threshold of p<0.00185 as significant. We found strong evidence that cognitive function was lower in CBHF compared to CHU across multiple domains. The KABC-II mental processing index was 45.2 (Standard Deviation (SD) 10.5) in CBHF and 48.3 (11.3) in CHU (mean difference 3.3 points [95% Confidence Interval (95%CI) 2.0, 4.5]; P<0.001). The school achievement test score was 39.0 (SD 26.0) in CBHF and 45.7 (27.8) in CHU (mean difference 7.3 points [95%CI 3.6, 10.9]; P<0.001); differences remained significant in adjusted analyses. The Plus-EF score of executive function was 109.4 (SD 24.7) in CBHF and 114.4 (24.2) in CHU (mean difference 5.4 [95%CI 2.3, 8.5]; P=0.001); differences were not significant in adjusted analyses. We found no consistent evidence of differences in growth or physical function outcomes.”**

Reviewer #3: A detailed response to my comments was not provided. However, looking over the changes made it appears all my previous comments have been addressed. I have no further issues to raise. **Thank you**


Reviewer #4: I am satisfied with the answers the authors have provided. **Thank you**


Reviewer #5: The authors have adequately addressed all my comments. I have no further comments on the paper. **Thank you**


Any attachments provided with reviews can be seen via the following link:


------------------------------------------------------------

General Editorial Requests

1) We ask every co-author listed on the manuscript to fill in a contributing author statement. If any of the co-authors have not filled in the statement, we will remind them to do so when the paper is revised. If all statements are not completed in a timely fashion this could hold up the re-review process. Should there be a problem getting one of your co-authors to fill in a statement we will be in contact. YOU MUST NOT ADD OR REMOVE AUTHORS UNLESS YOU HAVE ALERTED THE EDITOR HANDLING THE MANUSCRIPT TO THE CHANGE AND THEY SPECIFICALLY HAVE AGREED TO IT.

2) Please ensure that the paper adheres to the PLOS Data Availability Policy (see <http://journals.plos.org/plosmedicine/s/data-availability>), which requires that all data underlying the study's findings be provided in a repository or as Supporting Information. For data residing with a third party, authors are required to provide instructions with contact information for obtaining the data. PLOS journals do not allow statements supported by "data not shown" or "unpublished results." For such statements, authors must provide supporting data or cite public sources that include it.

3) Please review your reference list to ensure that it is complete and correct. If you have cited papers that have been retracted, please include the rationale for doing so in the manuscript text, or remove these references and replace them with relevant current references. Any changes to the reference list should be mentioned in the rebuttal letter that accompanies your revised manuscript.

__________________________________________________
In compliance with data protection regulations, you may request that we remove your personal registration details at any time.  (Use the following URL: <https://www.editorialmanager.com/pmedicine/login.asp?a=r>). Please contact the publication office if you have any questions.
